# Causal relationship between gut microbiota and puerperal sepsis: a 2-sample Mendelian randomization study

**DOI:** 10.3389/fmicb.2024.1407324

**Published:** 2024-06-12

**Authors:** Liu-dan Liang, Sheng Li, Mei-jin Huang, Hui-xin Peng, Zi-jun Lu, Zhuo-hua Zhang, Li-ye Su, Suren R. Sooranna, Yan Liu, Zhao-he Huang

**Affiliations:** ^1^Department of Cardiology, The First Clinical Medical College of Jinan University, Guangzhou, China; ^2^Department of Cardiology, Affiliated Hospital of Youjiang Medical University for Nationalities, Baise, China; ^3^Department of Infectious Diseases, Affiliated Hospital of Youjiang Medical University for Nationalities, Baise, China; ^4^Atherosclerosis and Ischemic Cardiovascular Diseases Laboratory, Youjiang Medical University for Nationalities, Baise, China; ^5^Graduate School, Youjiang Medical University for Nationalities, Baise, China; ^6^Life Science and Clinical Research Center, Youjiang Medical University for Nationalities, Baise, China; ^7^Department of Surgery and Cancer, Imperial College London, Chelsea and Westminster Hospital, London, United Kingdom

**Keywords:** causal relationship, gut microbiota, *Lachnospiraceae*, puerperal sepsis, Mendelian randomization, *Ruminococcaceae*

## Abstract

**Background:**

Some recent observational studies have shown that gut microbiota composition is associated with puerperal sepsis (PS) and no causal effect have been attributed to this. The aim of this study was to determine a causal association between gut microbiota and PS by using a two-sample Mendelian randomization (MR) analysis.

**Methods:**

This study performed MR analysis on the publicly accessible genome-wide association study (GWAS) summary level data in order to explore the causal effects between gut microbiota and PS. Gut microbiota GWAS (*n* = 18,340) were obtained from the MiBioGen study and GWAS-summary-level data for PS were obtained from the UK Biobank (PS, 3,940 cases; controls, 202,267 cases). Identification of single nucleotide polymorphisms associated with each feature were identified based on a significance threshold of *p* < 1.0 × 10^–5^. The inverse variance weighted (IVW) parameter was used as the primary method for MR and it was supplemented by other methods. Additionally, a set of sensitivity analytical methods, including the MR-Egger intercept, Mendelian randomized polymorphism residual and outlier, Cochran’s Q and the leave-one-out tests were carried out to assess the robustness of our findings.

**Results:**

Our study found 3 species of gut microbiota, *Lachnospiraceae FCS020*, *Lachnospiraceae NK4A136*, and *Ruminococcaceae NK4A214,* to be associated with PS. The IVW method indicated an approximately 19% decreased risk of PS per standard deviation increase with *Lachnospiraceae FCS020* (OR = 0.81; 95% CI 0.66–1.00, *p* = 0.047). A similar trend was also found with *Lachnospiraceae NK4A136* (OR = 0.80; 95% CI 0.66–0.97, *p* = 0.024). However, *Ruminococcaceae NK4A214* was positively associated with the risk of PS (OR = 1.33, 95% CI: 1.07–1.67, *p* = 0.011).

**Conclusion:**

This two-sample MR study firstly found suggestive evidence of beneficial and detrimental causal associations of gut microbiota on the risk of PS. This may provide valuable insights into the pathogenesis of microbiota-mediated PS and potential strategies for its prevention and treatment.

## 1 Introduction

Maternal sepsis is a life-threatening condition that leads to significant maternal morbidity and mortality, posing a tremendous socio-economic and healthcare burden worldwide. Data from the World Health Organization estimate that sepsis is responsible for 11.0–30.9% of total maternal deaths, ranking fifth among the causes of maternal mortality, with puerperal sepsis (PS) accounting for a significant portion of this burden (Say et al., 2014; Chebbo et al., 2016; Ngonzi et al., 2016; Padilla and Palanisamy, 2017; Parfitt and Hering, 2018; Plante et al., 2019). Hensley et al. (2019) analyzed data from 57.8% of the U.S. population and found that the incidence rate of maternal sepsis was 0.04%. In recent years, the incidence of maternal sepsis have been increasing. Kendle et al. (2019) found an annual increase of 6.6% in the incidence rate of delivery-associated sepsis in the United States, as well as an elevated fatality rate (20.6%) (Kuriya et al., 2016; Creanga, 2018). Furthermore, PS also increased the risk of postpartum anxiety and depression in mothers, and this not only interfered with family bonding but it also had a negative impact on breastfeeding of newborn infants (Groer and Morgan, 2007; Best et al., 2019). Hence, there is an urgent need to identify therapeutic as well as preventive targets for PS.

As the largest reservoir of bacteria and endotoxins in the body, the intestine is regarded as the “engine” of sepsis and multiple organ dysfunction syndrome (MODS), as well as participating in the metabolism of matter and energy (Anand and Mande, 2022). Under normal circumstances, the gut microbiota plays a beneficial role in human physiological processes and immune homeostasis, which is crucial for maintaining human health (O'Hara and Shanahan, 2006; Clarke et al., 2014). Gut microbiota dysbiosis has been demonstrated to increase the susceptibility to sepsis and these microorganisms may be a significant conductor for the pathogenesis of sepsis (Yang et al., 2021). In particular, in critically ill patients, it has been shown that the homeostasis of gut microbiota was disrupted, leading to abnormal changes in the type, number, proportion and location of gut microbes (McDonald et al., 2016). The interaction between disease and gut microbiota disorders can lead to clinical deterioration and the development of MODS (Adelman et al., 2020). During pregnancy, the gut microbiota undergoes profound changes, and imbalances in the gut microbiota composition are often associated with adverse pregnancy outcomes (Qi et al., 2021). There is increasing evidence supporting the relationship between gut microbiome dysbiosis and PS (Winther et al., 2020). Pregnancy hormones, the immune system and gut microbiome can interact with each other and changes in bacterial composition may be conducive to maintaining homeostasis and adapting to the physiological changes that accompanies pregnancy. These changes can also make women more vulnerable during pregnancy and postpartum, especially with respect to infectious diseases (Fuhler, 2020). Therefore, a better understanding of the changes in maternal gut microbiota may help to control the risk of PS.

Randomized controlled trials (RCTs) are an ideal method for inferring causality in epidemiological studies, but due to ethical limitations, the practice of RCTs remains challenging (Hariton and Locascio, 2018). Mendelian randomization (MR) is an alternative method which can effectively apply the data obtained from existing genome-wide association studies (GWAS) and we can use the genetic variation as an instrumental variable (IV) to explore the causal relationship between risk factors and outcomes (Hemani et al., 2018). According to Mendelian laws of inheritance, parental alleles are randomly assigned to offspring, a process equivalent to randomization in RCTs. In theory, genetic variations are not affected by common confounding factors such as the environment after birth. In addition, genetic variations predate exposures and outcomes, ruling out the problem of reverse causation and therefore, their use as IVs to analyze the causal relationship between exposures and outcomes is suitable for epidemiological studies.

At present, the role of gut microbiota in the risk of PS has not been evaluated within the MR framework. Therefore, this study is the first attempt to employ a two-sample MR approach to explore the causal relationship between genetically predicted gut microbiota and PS risk using aggregated statistics from GWAS obtained data.

## 2 Patients and methods

### 2.1 Study design

**Figure 1:**
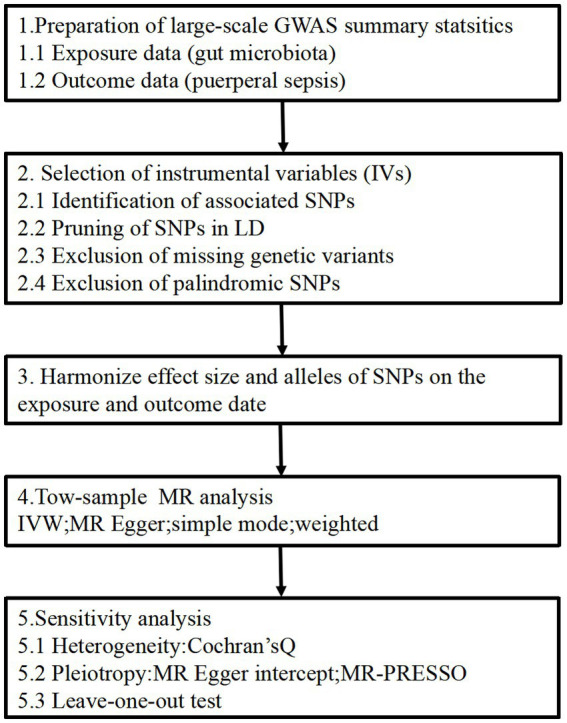
Study design of the MR study on the associations of gut microbiota and puerperal sepsis. SNPs, single nucleotide polymorphisms; LD, linkage disequilibrium; IVW, inverse variance weighted; MR, Mendelian randomization; MR-PRESSO, Mendelian randomized polymorphism residual and outlier.

The two-sample MR study design enables the estimation of the causal impacts of the exposure on the outcome by utilizing GWAS summary statistics from two distinct studies. The flowchart of our study is outlined in Figure 1. This approach relies on three core assumptions: (1) The genetic instruments should exhibit a robust correlation with the exposure, (2) the influence of the genetic instruments on the outcome should be solely mediated through the exposure and (3) the genetic instruments must be unrelated to any known factors that may confound the association between the exposure and the outcome. All methods carried out in this study were in accordance with relevant guidelines and regulations. This study has been approved by the Ethics Committee of the Affiliated Hospital of Youjiang Medical University for Nationalities (No. YFY-LL-2023-169).

### 2.2 Sources of data

The genetic summary-level data for gut microbiota was derived from the largest meta-analysis conducted by the MiBioGen consortium, which encompassed 24 cohorts and a total of 18,340 individuals. 72.3% of the participants (*n* = 13,266) in this study were of European descent (Kurilshikov et al., 2021). This research group targeted three distinct regions of the 16S rRNA gene (specifically V4, V3-V4, and V1-V2) to analyze the microbial composition and the taxonomic classification was conducted through direct taxonomic binning. During the microbiota quantitative trait loci (mbQTL) mapping analysis for each cohort, only the bacterial taxa that were detected in more than 10% of the samples were considered. This selection criterion was applied to evaluate the impact of host genetic variants on the abundance levels of these specific bacterial taxa within the gut microbiota. After excluding 12 unknown genera, a total of 119 others that passed the taxon inclusion cutoffs were included in this study. More detailed descriptions on genetic variants for gut microbiota can be found in the source publication (Kurilshikov et al., 2021).

For the risk of PS, we acquired GWAS summary data from the FinnGen study (R9) (Kurki et al., 2023). This ongoing project, integrates the germline genotype data from Finnish biobanks with health record data obtained from Finnish health registries, providing information on clinically defined outcomes. The dataset included 3,940 cases with PS and 202,267 controls for use in our analysis.

### 2.3 IVs

IVs can be used to control for confounding and measurement errors in observational studies because they allow for the possibility of making causal inferences with observed and unobserved data in epidemiological studies. A series of quality control steps was used in the selection of IVs for each feature in this study and these included: (1) Identification of associated single nucleotide polymorphisms (SNPs): SNPs associated with each feature were identified based on a significance threshold of *p* < 1.0 × 10^−5^. (2) Pruning of SNPs in linkage disequilibrium (LD): SNPs within a 500-kilobase window and with a LD threshold of *r*^2^ < 0.1 were removed. This process was based on the 1,000 Genomes Project reference panel for European populations (Phase 3). Among the remaining SNPs, those with the lowest *p*-values from the GWAS were retained. (3) Exclusion of missing genetic variants: genetic variants that were not present in the outcome of the GWAS dataset were excluded from the analysis. This was to avoid their replacement by proxy variants. (4) Exclusion of palindromic SNPs, which are SNPs with alleles consisting of a base as well as its complementary base, were excluded. This step ensured that the effects of the SNPs on the exposures corresponded to the same allele as their effects on the outcomes. We estimated the $R^{2}$ value for each MR test conducted and this represented the proportion of variance in the phenotype explained by the genetic variants. Additionally, we calculated the mean F-statistical value as a measure of the strength of the IV used (Burgess and Thompson, 2011; Papadimitriou et al., 2020; Ni et al., 2021).

### 2.4 Statistical analysis

All analyses were conducted using the TwoSampleMR package (version 0.5.6) in R, specifically version 4.2.1. Our primary analysis utilized the inverse-variance weighted (IVW) approach, which offers the highest statistical power (Burgess et al., 2016). To address potential violations of MR assumptions resulting from different pleiotropy scenarios, we performed multiple sensitivity analyses, including the MR-Egger, weighted median, simple mode and weighted mode (Hartwig et al., 2017). Previous studies have suggested that relying solely on *p*-value thresholds to determine “significance” was not ideal (Sterne and Davey, 2001; Li et al., 2017). Therefore, we employed three criteria to establish a reliable causal association: (1) The MR-IVW estimate for each feature should surpassed the *p*-value threshold, (2) The MR estimates must consistently demonstrate the same direction of effect across all sensitivity analyses, and (3) Limited evidence of horizontal pleiotropy should be observed through either the MR-Egger intercept test or the Mendelian Randomization Pleiotropy Residual Sum and Outlier (MR-PRESSO) global test (Bowden et al., 2015; Verbanck et al., 2018). Furthermore, we also assessed the heterogeneity of IVs using the Cochran’s Q-statistical test and performed a leave-one SNP-out analysis in order to ensure that the results were not biased by any individual SNP (Burgess et al., 2017).

## 3 Results

### 3.1 Selection of IVs

A total 1,269 IVs were screened for 119 bacterial genera from the MiBioGen consortium. These IVs explained 0.34–3.33% of the variance and that the F-statistics for them were all greater than 10 (ranging from 20.46 to 28.66). These parameters signified the robustness and strength of the IVs employed in this study. This indicated the absence of any significant weak instrument bias in the results we obtained, making the findings more reliable and acceptable (Supplementary Table S1).

### 3.2 Two-sample MR analysis

**Figure 2:**
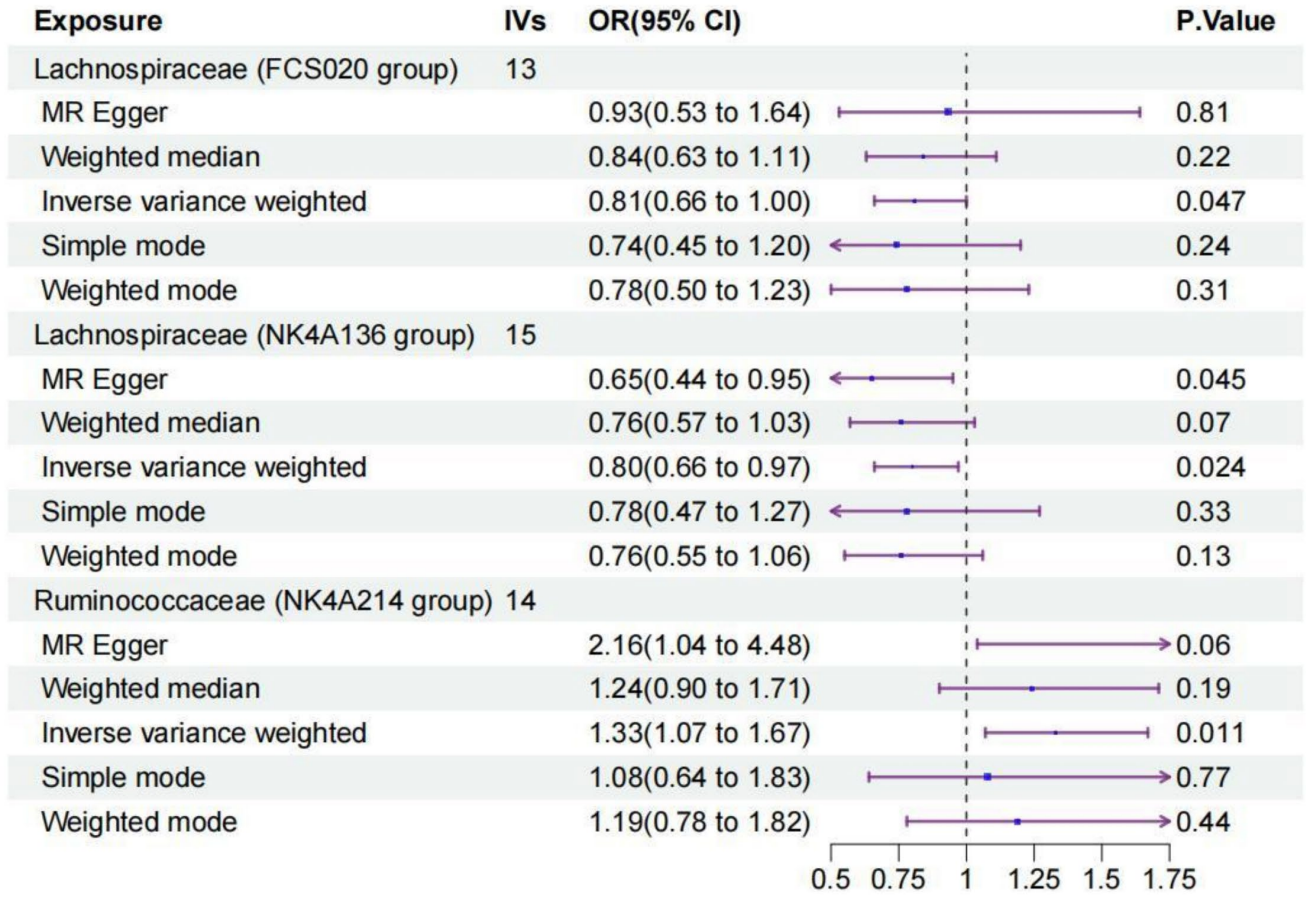
Forest plot of the causal relationships estimated for three gut microbiota and puerperal sepsis by using five MR methods including IVW, MR-Egger, weighted median, simple mode and weighted mode. The blue dots and purple bars represent the MR and the 95% confidence intervals of IMR analysis estimates, respectively. The OR > 1 indicates increased risk while <1 indicates decreased risk. MR, Mendelian randomization; CI, confidence interval; OR, odds ratio; IVW, inverse-variance weighted.

We identified three species of gut microbiota, *Lachnospiraceae FCS020*, *Lachnospiraceae NK4A136*, and *Ruminococcaceae NK4A214*, related to PS according to the criteria outlined above in which the IVW method demonstrated a significant difference (*p* < 0.05), and the other methods indicated consistent directional effects. The IVW method indicated an approximately 19% decreased risk of PS per standard deviation (SD) increase in *Lachnospiraceae FCS020* (OR = 0.81; 95% CI 0.66–1.00, *p* = 0.047). A similar trend was also found with *Lachnospiraceae NK4A136* (OR = 0.80; 95% CI 0.66–0.97, *p* = 0.024). However, the IVW method also showed that *Ruminococcaceae NK4A214* was positively associated with a risk of PS (OR = 1.33, 95% CI: 1.07–1.67, *p* = 0.011) (Table 1; Figure 2; Supplementary Table S2).

**Table 1:** MR estimates for three gut microbiota species.

Bacterial taxa (exposure)	MR method	No. of SNP	OR (95%CI)	*p*-value	FDR
Lachnospiraceae FCS020 group	MR Egger	13	0.93 (0.53–1.64)	0.81	
Lachnospiraceae FCS020 group	Weighted median	13	0.84 (0.63–1.11)	0.22	
Lachnospiraceae FCS020 group	Inverse variance weighted	13	0.81 (0.66–1.00)	4.70E-02	4.70E-02
Lachnospiraceae FCS020 group	Simple mode	13	0.74 (0.45–1.20)	0.24	
Lachnospiraceae (FCS020 group)	Weighted mode	13	0.78 (0.50–1.23)	0.31	
Lachnospiraceae NK4A136 group	MR Egger	15	0.65 (0.44–0.95)	4.50E-02	
Lachnospiraceae NK4A136 group	Weighted median	15	0.76 (0.57–1.03)	0.07	
Lachnospiraceae NK4A136 group	Inverse variance weighted	15	0.80 (0.66–0.97)	2.40E-02	3.60E-02
Lachnospiraceae NK4A136 group	Simple mode	15	0.78 (0.47–1.27)	0.33	
Lachnospiraceae NK4A136 group	Weighted mode	15	0.76 (0.55–1.06)	0.13	
Ruminococcaceae NK4A214 group	MR Egger	14	2.16 (1.04–4.48)	0.06	
Ruminococcaceae NK4A214 group	Weighted median	14	1.24 (0.90–1.71)	0.19	
Ruminococcaceae NK4A214 group	Inverse variance weighted	14	1.33 (1.07–1.67)	1.10E-02	3.30E-02
Ruminococcaceae NK4A214 group	Simple mode	14	1.08 (0.64–1.83)	0.77	
Ruminococcaceae NK4A214 group	Weighted mode	14	1.19 (0.78–1.82)	0.44	

Five MR methods including IVW, MR-Egger, weighted median, simple mode and weighted mode were used to evaluate the causal relationship between 3 gut microbiota and puerperal sepsis, and the IVW method demonstrated a significant difference between them (*p* < 0.05). Among them, gut microbiota and *Lachnospiraceae FCS020* which included a total of 13 SNPs as instrumental variables, the IVW result was OR = 0.81; 95% CI: 0.66–1.00, *p* = 0.047. For gut microbiota and *Lachnospiraceae NK4A136* which included 15 SNPs as instrumental variables, the IVW result was OR = 0.80; 95% CI: 0.66–0.97, *p* = 0.024. For gut microbiota and *Ruminococcaceae NK4A214* which included 14 SNPs as instrumental variables, the IVW result was OR = 1.33, 95% CI: 1.07–1.67, *p* = 0.011. Additionally, we used the false discovery rate (FDR) to correct our results for multiple hypothesis testing, with the significance level set at an FDR-corrected value of *p* < 0.05 (Benjamini and Hochberg, 1995).MR, Mendelian randomization; SNP, single nucleotide polymorphism; CI, confidence interval; OR, odds ratio; IVW, inverse-variance weighted; FDR, false discovery rate.

### 3.3 Sensitivity analyses and leave-one-out analysis

**Figure 3:**
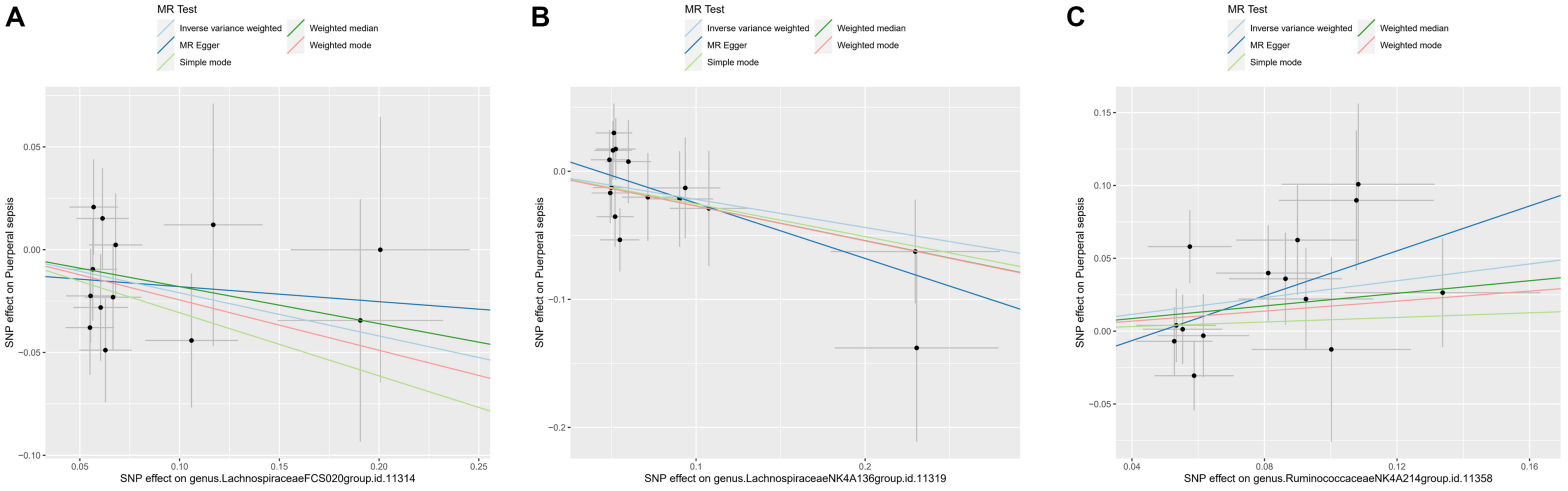
The scatter plot of MR estimates for **(A)** *Lachnospiraceae FCS020*, **(B)** Lachnospiraceae NK4A136 and **(C)** Ruminococcaceae NK4A214. The light blue, dark blue, light green, dark green, and red lines represent the association between microbiota (exposure) and puerperal sepsis (outcome) when estimated by the IVW method, MR-Egger regression, simple mode, weighted median and weighted mode, respectively. The vertical and horizontal black lines around each point represent the 95% confidence interval for each polymorphism with respect to associations with exposure and outcome, respectively. SNP, single nucleotide polymorphism; MR, Mendelian randomization; IVW, inverse-variance weighted.

**Figure 4:**
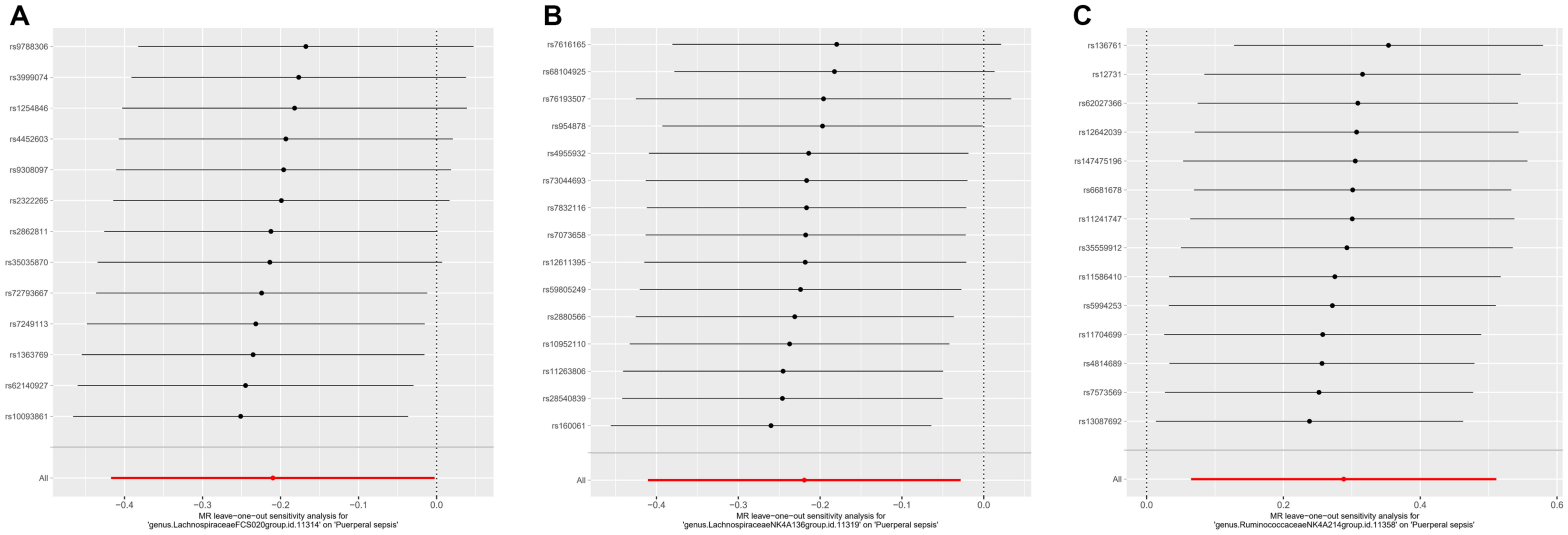
The leave one out analysis of MR estimates for **(A)** *Lachnospiraceae FCS020*, **(B)** Lachnospiraceae NK4A136 and **(C)** Ruminococcaceae NK4A214. The black dots represent the causal estimate for the association between a specific exposure and target after discarding each SNP in turn. The red dots represent the overall causal estimate by using the random-effect IVW. The horizontal lines denote 95% confidence intervals. SNP, single nucleotide polymorphism; MR, Mendelian randomization; IVW, inverse-variance weighted.

For these three gut microbiota species, no heterogeneity was found by using either the IVW method or the MR-Egger regression, and the *p*-values for Cochran’s Q test were all >0.05 (Supplementary Table S3). The MR Egger regression intercepts were near zero and these revealed little evidence of horizontal pleiotropy in the IVs (Supplementary Table S4). Using the MR-PRESSO global outlier test, no evidence for outliers was observed (Supplementary Table S5). The scatter plots did not demonstrate any potential outliers in the IVs for these three gut microbiota species (Figure 3). In the leave-one SNP-out analyses, the risk estimates of all gut microbiota genera generally remained consistent after eliminating each SNP at a time, suggesting that these were no identified leverage points with high influence (Figure 4). The full results of the MR estimates for the 119 gut microbiota genera on PS are presented in Supplementary Tables S2–S4.

## 4 Discussion

In this study, we performed a two-sample MR analysis in order to evaluate the causal relationship between gut microbiota and PS. We used the summary statistics on gut microbiota from the largest GWAS meta-analysis conducted by the MiBioGen Consortium and the summary statistics on PS from the FinnGen Consortium R9 published data. We found a causal relationship between the risk of PS and three species of gut microbiota, namely *Lachnospiraceae FCS020*, *Lachnospiraceae NK4A136*, and *Ruminococcaceae NK4A214*. The increased abundance of *Lachnospiraceae FCS020* and *Lachnospiraceae NK4A136* was negatively associated with the risk of PS, while *Ruminococcaceae NK4A214* appeared to be a risk factor for the disease.

The human intestine has a complex ecosystem of bacteria, accounting for about 80% of the total microbiota. Human gut microbiota consists of numerous microorganisms, but the most abundant species belong to the phyla, Firmicutes (synonym Bacillota) and Bacteroidetes (synonym Bacteroidota) (Ramakrishna, 2013; Akash et al., 2019; Firmino et al., 2020), fulfilling an irreplaceable role in maintaining host health. Firmicutes possess anti-inflammatory properties and can alleviate the progression of inflammatory bowel disease. Bacteroidetes perform metabolic conversions such as degradation of proteins and complex sugar polymers which are essential for the host. The perinatal period is a unique period during which a woman’s physiological status (including hormone levels, metabolism and immunity) undergoes significant alternations (Newbern and Freemark, 2011). As hormones can impact bacterial growth, changes in hormonal levels may affect gut microbiota during pregnancy (Neuman and Koren, 2017). Moreover, substantial changes in host hormones levels, metabolism and immunity before and after delivery may be accompanied by changes in gut microbiota (Salzman et al., 2010; Vijay-Kumar et al., 2010). The gut microbiome during pregnancy is characterized by a decrease in abundance and an increase in inter-subject diversity. The relative abundance of Actinobacteria, Proteobacteria, and Coprobacilli increases on average from the first to the third trimesters of pregnancy, and these effects persist until the perinatal period (Koren et al., 2012). No substantial differences in maternal gut microbiome have been found when comparing the third trimester with the first few months postpartum (Koren et al., 2012; Jost et al., 2014).

Sepsis is defined as a life-threatening organ dysfunction caused by a dysfunctional host response to infection (Singer et al., 2016). An increasing body of evidence now suggests that gut microbiota play an important role in the pathophysiology of sepsis and dysbiosis of these organisms been associated with numerous sepsis-associated pathogenic conditions (Dickson, 2016; Klingensmith and Coopersmith, 2016). The spectrum of gut microbiota can also potentially be used as a diagnostic and prognostic marker for sepsis (Liu et al., 2021). Specific physiological changes during pregnancy can alter the gut microbiome, thus increasing the risk of sepsis-like infections.

Our study identified 3 specific bacterial species having a causal relationship with the risk of PS. Among them, *Lachnospiraceae FCS020* and *Lachnospiraceae NK4A136* are important members of *Lachnospiraceae*. *Lachnospiraceae* and *Ruminococcaceae* belong to the phylum Firmicutes and are the most common bacterial groups found in the human gut. The bacteria which belong to the Lachnospiraceae family are anaerobic and they are abundant in a healthy gut. They can produce short-chain-fatty acids (SCFA) (Vacca et al., 2020) by converting primary into secondary bile acids. SCFA can enhance the integrity of the intestinal epithelial barrier and inhibit inflammation, which is important for maintaining the gastro-intestinal health (Hu et al., 2019). Sorbara et al. (2020) found a protective effect of *Lachnospiraceae* on metabolic health during sepsis. Consistent with these findings, we found an increased abundance of *Lachnospiraceae FCS020* and *Lachnospiraceae NK4A136* can reduce the risk of PS. However, Muratsu et al. (2022) showed that the Lachnospiraceae family increased in the subacute stage (day 7 after caecal ligation and puncture) in a mouse model of sepsis (Muratsu et al., 2022), which was inconsistent with our findings. A study on the component flux of the gut microbiome and the risk of gram-negative bloodstream infections found that *Ruminococcaceae* is associated with a reduced risk of gram-negative dominance (Stoma et al., 2021). The proportion of *Ruminococcaceae* in healthy patients was higher than that in sepsis patients (Antharam et al., 2013). However, we found that *Ruminococcaceae NK4A214* may be a risk factor for PS. *Ruminococcaceae* are known to have a beneficial effect on gut barrier function (Madsen, 2001). In addition, some species are also depleted in Crohn’s disease and ulcerative colitis and they have also been shown to have anti-inflammatory properties (Sokol et al., 2008).

At present, the changes in gut microbiota in PS patients have not been reported. These inconsistent results may be explained by the considerable inter-species and intra-species diversity in the gut microbiota, which may have had a significant impact on PS. Standardized and specific gut microbiota classification systems are essential for subsequent mechanistic studies and clinical guidance. Other studies that have explored the role of gut microbiota in sepsis and other related conditions. For example, a recent study looked at the correlation between gut microbiota, C- reactive protein and sepsis, and suggested that this protein has a potential role as a mediator in facilitating the impact of some bacterial species on sepsis (Zhang et al., 2023). Microbiota may also cause an imbalance in the lung and intestinal immunity by causing bacterial translocation through the TLR4 and NF-kB pathway resulting in inflammation of lung tissues (Sun et al., 2022).

The changes observed in the levels of *Lachnospiraceae FCS020*, *Lachnospiraceae NK4A136*, and *Ruminococcaceae NK4A214* in this study with PS may potentially involve interactions with the microbiomes from other epithelial sites of the body, which are known to impact human physiology, both in health and in disease (Ogunrinola et al., 2020). In particular, the microbiome directly associated with the reproductive tract (Rizzo et al., 2021), urinary tract (Kim and Lee, 2023) and skin (Byrd et al., 2018) can have more direct and localized effects on specific diseases. Lactobacilli dominate the microbiome of the human reproductive tract and the maintenance of a healthy microbiota can protect the host from pathogens, increase reproductive potential and reduce the rates of adverse pregnancy outcomes (Rizzo et al., 2021; Wang et al., 2024). These types of studies suggest that our observed changes may be due to modifications in the gut microbiome as it tries to either overcome or ameliorate the onset of PS. However, further speculations are beyond the scope of this study.

The present study has several advantages. Firstly, the causal relationship between gut microbiota and PS was determined by MR analysis, thereby eliminating confounding factors and avoiding the effect of reverse causation on possible causal inference. Secondly, the genetic variation in the gut microbiota was obtained from the largest available GWAS meta-analysis, ensuring the strength of the IVs in the MR analysis. In addition, we detected and corrected for the effect of horizontal pleiotropy by using MR-Egger regression and MR-PRESSO analyses. However, our study also had some limitations. Firstly, the SNPs based on the genome-wide statistical significance threshold (5 × 10^−8^) were too limited, so we included a genome-wide significance level (1 × 10^−5^) for this study. Secondly, the GWAS data in this study were mainly from European populations, with only a small portion of gut microbiome data from other races, thus potentially leading to partial bias. In addition, our study was based on the 16S rRNA gene and the broad classification of PS and these are accompanied by their own limitations until more detailed GWAS data become available. Thirdly, we could not rule out possible diet-gene and gene–environment interactions in our analysis and these may have influenced the observed results we obtained. We would also like to perform longitudinal observational studies in future in order to provide insights on how changes in gut microbiota over time correlate with the incidence of PS. The goal of these studies will lead to clinical trials on modifying gut microbiota (e.g., through probiotics and/or diet) to directly test the effectiveness of such interventions in future prevention and management of PS.

In this study we used two large publicly accessible genomic databases to study the link gut microbes and a type of sepsis. Based on the statistical methods used, we found evidence for some beneficial as well as detrimental effects of gut microbiota on the risk of PS. This may provide insights on the pathogenesis of microbe-mediated PS and lead to potential prevention and remedies for this condition.

## 5 Conclusion

This two-sample MR study found a causal relationship between gut microbiota and PS, providing ideas for further research on the pathogenesis of this disease. In addition, the data suggest that PS may be mediated by gut microbiota. This study provides valuable insights into the pathogenesis of microbiota-mediated PS and suggestions for potential novel targets for prevention and treatment strategies of this condition. Further RCTs are needed in order to further elucidate the role of gut microbiota in PS and the specific mechanisms involved.

## Data availability statement

The original contributions presented in the study are included in the article/Supplementary material, further inquiries can be directed to the corresponding authors.

## Ethics statement

This study was conducted after approval by the Affiliated Hospital of Youjiang Medical University for Nationalities (No. YFY-LL-2023-169). All the methods used were carried out in accordance with relevant guidelines and regulations.

## Author contributions

L-dL: Conceptualization, Data curation, Formal analysis, Writing – original draft. SL: Formal analysis, Writing – original draft. M-jH: Formal analysis, Writing – original draft. H-xP: Formal analysis, Writing – original draft. Z-jL: Formal analysis, Writing – original draft. Z-hZ: Formal analysis, Writing – original draft. L-yS: Formal analysis, Writing – original draft. SS: Conceptualization, Writing – review & editing. YL: Conceptualization, Formal analysis, Funding acquisition, Methodology, Writing – review & editing. Z-hH: Conceptualization, Funding acquisition, Supervision, Writing – review & editing.
