# Supplementary material for: Causal relationship between gut microbiota and puerperal sepsis: a 2-sample Mendelian randomization study
Source: Front Microbiol. 2024 Jun 12;15:1407324. doi: 10.3389/fmicb.2024.1407324 (PMC11203603; doi:10.3389/fmicb.2024.1407324)
Supplement: Supplementary file 1 [file Data_Sheet_1.zip › Legends to Supplementary Tables and Figures.docx]

**Legends to Supplementary Tables and Figures.**

**Supplementary Figure 1**

Association of the species with the puerperal sepsis forest. The MR effect size for three gut microbiota on puerperal sepsis. Each point represents the effect value of each SNP, and the line segment represents the 95% confidence interval. MR, Mendelian randomization; SNP, single nucleotide polymorphism.

**Supplementary Figure 2**

Association of the species with the puerperal sepsis by using a funnel plot. The horizontal coordinate represents the effect value of the instrumental variable (βIV). The ordinate represents the tool variable 1/SEIV and the black dots represent the SNP. The vertical lines represent estimates of the combined effect from the IVW and MR Egger regression methods, respectively. The vertical lines represent estimates of the combined effect from the IVW and MR-Egger regression methods, respectively. MR, Mendelian randomization; IVW, inverse-variance weighted; IV, instrumental variable.

**Supplementary Figure 3**

Preliminary MR analyses for the associations between gut microbiota and the risk of puerperal sepsis. The outer to the inner circles represent the IVW, MR-Egger, WM, simple mode and weighted mode estimates, respectively. Gut microbiota were classified in order, phylum, class, family and genus. The shades of color reflect the magnitude of the p-values as labelled inside the circle. (MR, Mendelian randomization; IVW, inverse variance-weighted; WM, weighted median).

**Table S1** The instrumental variables used in MR analysis for the association between gut microbiota and puerperal sepsis.

**Table S2** The F-statistic data for the instrumental variables.

**Table S3** The full results of MR estimates for the association between gut microbiota and puerperal sepsis.

**Table S4** The heterogeneity of gut microbiota instrumental variables.

**Table S5** The directional horizontal pleiotropy when assessed by intercept terms in MR Egger regression and MR-PRESSO between gut microbiota and puerperal sepsis.
