# Supplementary material for: Causal relationship between gut microbiota and puerperal sepsis: a 2-sample Mendelian randomization study
Source: Front Microbiol. 2024 Jun 12;15:1407324. doi: 10.3389/fmicb.2024.1407324 (PMC11203603; doi:10.3389/fmicb.2024.1407324)
Supplement: Supplementary file 1 [file Data_Sheet_1.zip › Supplemetary Tables.docx]

**Table S1** The Instrumental variables used in MR analysis for the association between gut microbiota and puerperal sepsis.

| **Bacterial taxa (exposure)** | **SNP** | | **Effect allele** | | | | **Other allele** | | | **Exposure (Bacteria) Outcome** | | | | | | | | | | | | | |  | | | | |
| --- | --- | --- | --- | --- | --- | --- | --- | --- | --- | --- | --- | --- | --- | --- | --- | --- | --- | --- | --- | --- | --- | --- | --- | --- | --- | --- | --- | --- |
|  |  |  |  |  |  |  |  |  |  | **Beta SE P-value** | | | | | | | **Beta SE** | | | **P-value** | | | |  | | |  |  |
| genus..Clostridiuminnocuumgroup.id.14397 | | rs10506058 | | A | | G | | | 0.0997048 | | | 0.022192573 | | 8.92E-06 | | 0.0123425 | | | 0.023161 | | | 0.59411 | | |  |  |  |  |
| genus..Clostridiuminnocuumgroup.id.14397 | | rs1942371 | | G | | A | | | -0.157938 | | | 0.034186957 | | 4.06E-06 | | -0.0417076 | | | 0.034981 | | | 0.233142 | | |  |  |  |  |
| genus..Clostridiuminnocuumgroup.id.14397 | | rs1948423 | | T | | A | | | -0.1088591 | | | 0.023424976 | | 3.49E-06 | | 0.0257384 | | | 0.024 | | | 0.283529 | | |  |  |  |  |
| genus..Clostridiuminnocuumgroup.id.14397 | | rs40656 | | C | | T | | | 0.1426644 | | | 0.031102116 | | 8.62E-06 | | -0.0285496 | | | 0.028576 | | | 0.317759 | | |  |  |  |  |
| genus..Clostridiuminnocuumgroup.id.14397 | | rs4869133 | | G | | A | | | -0.1805905 | | | 0.040950516 | | 7.24E-06 | | -0.0066974 | | | 0.030083 | | | 0.823821 | | |  |  |  |  |
| genus..Clostridiuminnocuumgroup.id.14397 | | rs61267978 | | T | | C | | | 0.1470795 | | | 0.032087502 | | 5.59E-06 | | 0.0007143 | | | 0.035139 | | | 0.983782 | | |  |  |  |  |
| genus..Clostridiuminnocuumgroup.id.14397 | | rs6577484 | | G | | A | | | 0.1604248 | | | 0.036085736 | | 8.41E-06 | | -0.0111348 | | | 0.037532 | | | 0.766717 | | |  |  |  |  |
| genus..Clostridiuminnocuumgroup.id.14397 | | rs6890185 | | T | | C | | | 0.1134237 | | | 0.023313729 | | 1.12E-06 | | -0.0685885 | | | 0.024553 | | | 0.00521483 | | |  |  |  |  |
| genus..Clostridiuminnocuumgroup.id.14397 | | rs71564433 | | T | | A | | | -0.1267459 | | | 0.027465666 | | 7.80E-06 | | -0.01432 | | | 0.028673 | | | 0.617482 | | |  |  |  |  |
| genus..Clostridiuminnocuumgroup.id.14397 | | rs77845139 | | A | | G | | | -0.1149929 | | | 0.02571863 | | 8.41E-06 | | -0.0204222 | | | 0.026387 | | | 0.438963 | | |  |  |  |  |
| genus..Eubacteriumbrachygroup.id.11296 | | rs112617308 | | T | | C | | | -0.1708734 | | | 0.036284397 | | 2.38E-06 | | 0.0813743 | | | 0.039639 | | | 0.0400821 | | |  |  |  |  |
| genus..Eubacteriumbrachygroup.id.11296 | | rs12151423 | | A | | G | | | 0.1013286 | | | 0.022732445 | | 9.27E-06 | | -0.0023099 | | | 0.022816 | | | 0.919362 | | |  |  |  |  |
| genus..Eubacteriumbrachygroup.id.11296 | | rs13139592 | | T | | C | | | -0.1459966 | | | 0.032719209 | | 7.97E-06 | | -0.0046811 | | | 0.033625 | | | 0.88928 | | |  |  |  |  |
| genus..Eubacteriumbrachygroup.id.11296 | | rs1384962 | | A | | G | | | 0.1208883 | | | 0.026626427 | | 6.99E-06 | | 0.0164449 | | | 0.024573 | | | 0.503351 | | |  |  |  |  |
| genus..Eubacteriumbrachygroup.id.11296 | | rs2913110 | | C | | T | | | 0.1051431 | | | 0.022941973 | | 4.56E-06 | | 0.0061291 | | | 0.023932 | | | 0.797873 | | |  |  |  |  |
| genus..Eubacteriumbrachygroup.id.11296 | | rs4862235 | | G | | A | | | 0.1048063 | | | 0.022575135 | | 3.73E-06 | | 0.0115127 | | | 0.022954 | | | 0.615986 | | |  |  |  |  |
| genus..Eubacteriumbrachygroup.id.11296 | | rs62348779 | | T | | C | | | -0.2014806 | | | 0.043286015 | | 3.78E-06 | | 0.0022921 | | | 0.042967 | | | 0.957457 | | |  |  |  |  |
| genus..Eubacteriumbrachygroup.id.11296 | | rs6591893 | | G | | A | | | 0.1082254 | | | 0.024031859 | | 7.34E-06 | | 0.016964 | | | 0.024073 | | | 0.480997 | | |  |  |  |  |
| genus..Eubacteriumbrachygroup.id.11296 | | rs720439 | | A | | G | | | -0.111927 | | | 0.025125245 | | 7.03E-06 | | -0.0052519 | | | 0.026672 | | | 0.8439 | | |  |  |  |  |
| genus..Eubacteriumbrachygroup.id.11296 | | rs73199919 | | T | | C | | | -0.2367168 | | | 0.053133485 | | 8.16E-06 | | -0.025201 | | | 0.053422 | | | 0.637118 | | |  |  |  |  |
| genus..Eubacteriumbrachygroup.id.11296 | | rs9613196 | | T | | A | | | -0.2387185 | | | 0.052856104 | | 4.99E-06 | | 0.0795236 | | | 0.057073 | | | 0.163509 | | |  |  |  |  |
| genus..Eubacteriumcoprostanoligenesgroup.id.11375 | | rs10444197 | | A | | G | | | -0.0505823 | | | 0.011345529 | | 5.98E-06 | | -0.0215807 | | | 0.02396 | | | 0.367754 | | |  |  |  |  |
| genus..Eubacteriumcoprostanoligenesgroup.id.11375 | | rs11052069 | | T | | C | | | 0.0477832 | | | 0.01078333 | | 9.38E-06 | | 0.0279757 | | | 0.023004 | | | 0.22393 | | |  |  |  |  |
| genus..Eubacteriumcoprostanoligenesgroup.id.11375 | | rs11720857 | | C | | T | | | 0.0630776 | | | 0.014448482 | | 9.26E-06 | | -0.0770358 | | | 0.02994 | | | 0.0100811 | | |  |  |  |  |
| genus..Eubacteriumcoprostanoligenesgroup.id.11375 | | rs12906958 | | C | | T | | | -0.0533159 | | | 0.011593477 | | 4.35E-06 | | -0.024743 | | | 0.025051 | | | 0.323299 | | |  |  |  |  |
| genus..Eubacteriumcoprostanoligenesgroup.id.11375 | | rs17159861 | | C | | T | | | 0.0962228 | | | 0.016838828 | | 1.04E-08 | | 0.0116598 | | | 0.036998 | | | 0.75265 | | |  |  |  |  |
| genus..Eubacteriumcoprostanoligenesgroup.id.11375 | | rs2644213 | | G | | A | | | 0.0538845 | | | 0.012124458 | | 9.86E-06 | | -0.0045375 | | | 0.024849 | | | 0.855111 | | |  |  |  |  |
| genus..Eubacteriumcoprostanoligenesgroup.id.11375 | | rs4076415 | | T | | G | | | 0.0515152 | | | 0.011029435 | | 1.99E-06 | | 0.0015336 | | | 0.023759 | | | 0.948534 | | |  |  |  |  |
| genus..Eubacteriumcoprostanoligenesgroup.id.11375 | | rs4717831 | | A | | T | | | 0.0786783 | | | 0.017403801 | | 9.18E-06 | | 0.0225457 | | | 0.032534 | | | 0.488317 | | |  |  |  |  |
| genus..Eubacteriumcoprostanoligenesgroup.id.11375 | | rs62024432 | | C | | T | | | -0.0769698 | | | 0.017210135 | | 7.50E-06 | | 0.0507579 | | | 0.038842 | | | 0.191283 | | |  |  |  |  |
| genus..Eubacteriumcoprostanoligenesgroup.id.11375 | | rs6762473 | | C | | A | | | 0.0521584 | | | 0.011235251 | | 4.26E-06 | | 0.0064076 | | | 0.024032 | | | 0.789754 | | |  |  |  |  |
| genus..Eubacteriumcoprostanoligenesgroup.id.11375 | | rs73109678 | | G | | A | | | -0.0796956 | | | 0.017227516 | | 3.66E-06 | | 0.097163 | | | 0.041898 | | | 0.0203939 | | |  |  |  |  |
| genus..Eubacteriumcoprostanoligenesgroup.id.11375 | | rs76898927 | | G | | A | | | 0.123055 | | | 0.026637491 | | 4.79E-06 | | 0.0130162 | | | 0.05024 | | | 0.795571 | | |  |  |  |  |
| genus..Eubacteriumcoprostanoligenesgroup.id.11375 | | rs9648214 | | T | | C | | | -0.0828719 | | | 0.016430987 | | 2.52E-07 | | 0.0428167 | | | 0.040493 | | | 0.290331 | | |  |  |  |  |
| genus..Eubacteriumeligensgroup.id.14372 | | rs158115 | | G | | C | | | 0.0915271 | | | 0.019061087 | | 9.74E-06 | | -0.0116338 | | | 0.034073 | | | 0.732771 | | |  |  |  |  |
| genus..Eubacteriumeligensgroup.id.14372 | | rs182318 | | G | | A | | | -0.0824941 | | | 0.019561497 | | 8.40E-06 | | -0.0189137 | | | 0.041517 | | | 0.6487 | | |  |  |  |  |
| genus..Eubacteriumeligensgroup.id.14372 | | rs2200429 | | A | | G | | | -0.0888825 | | | 0.019847898 | | 5.30E-06 | | 0.0425975 | | | 0.038409 | | | 0.267403 | | |  |  |  |  |
| genus..Eubacteriumeligensgroup.id.14372 | | rs265534 | | T | | G | | | -0.0563756 | | | 0.01201272 | | 2.27E-06 | | -0.0156206 | | | 0.02292 | | | 0.495531 | | |  |  |  |  |
| genus..Eubacteriumeligensgroup.id.14372 | | rs4583233 | | A | | C | | | 0.0670205 | | | 0.012812178 | | 2.84E-07 | | 0.0007393 | | | 0.025383 | | | 0.976764 | | |  |  |  |  |
| genus..Eubacteriumeligensgroup.id.14372 | | rs4888188 | | T | | G | | | -0.053328 | | | 0.011912579 | | 7.74E-06 | | -0.0060349 | | | 0.023216 | | | 0.794904 | | |  |  |  |  |
| genus..Eubacteriumeligensgroup.id.14372 | | rs56080211 | | C | | T | | | 0.1230927 | | | 0.028274315 | | 9.14E-06 | | 0.0525671 | | | 0.043054 | | | 0.222101 | | |  |  |  |  |
| genus..Eubacteriumeligensgroup.id.14372 | | rs6923695 | | T | | G | | | 0.1032676 | | | 0.02295245 | | 4.87E-06 | | 0.0022224 | | | 0.04585 | | | 0.961341 | | |  |  |  |  |
| genus..Eubacteriumeligensgroup.id.14372 | | rs72839198 | | C | | G | | | 0.155659 | | | 0.037561222 | | 3.14E-06 | | 0.0628651 | | | 0.039492 | | | 0.111423 | | |  |  |  |  |
| genus..Eubacteriumeligensgroup.id.14372 | | rs74606150 | | C | | G | | | -0.1964535 | | | 0.042564951 | | 4.25E-06 | | -0.0015422 | | | 0.058194 | | | 0.978857 | | |  |  |  |  |
| genus..Eubacteriumfissicatenagroup.id.14373 | | rs10147907 | | T | | G | | | 0.1722628 | | | 0.039600983 | | 8.27E-06 | | 0.0532032 | | | 0.043491 | | | 0.221213 | | |  |  |  |  |
| genus..Eubacteriumfissicatenagroup.id.14373 | | rs11818408 | | G | | A | | | 0.1058503 | | | 0.023711404 | | 8.20E-06 | | -0.0245992 | | | 0.023397 | | | 0.293089 | | |  |  |  |  |
| genus..Eubacteriumfissicatenagroup.id.14373 | | rs11876297 | | T | | C | | | 0.1314694 | | | 0.028171203 | | 2.67E-06 | | 0.0014398 | | | 0.025791 | | | 0.955481 | | |  |  |  |  |
| genus..Eubacteriumfissicatenagroup.id.14373 | | rs151257695 | | A | | G | | | 0.2095098 | | | 0.045484723 | | 3.10E-06 | | -0.0466122 | | | 0.044421 | | | 0.294026 | | |  |  |  |  |
| genus..Eubacteriumfissicatenagroup.id.14373 | | rs1768152 | | T | | C | | | 0.1394886 | | | 0.031618584 | | 8.70E-06 | | -0.0579335 | | | 0.037365 | | | 0.121026 | | |  |  |  |  |
| genus..Eubacteriumfissicatenagroup.id.14373 | | rs2733072 | | G | | A | | | 0.1096442 | | | 0.022830587 | | 1.49E-06 | | -0.0299662 | | | 0.022913 | | | 0.190936 | | |  |  |  |  |
| genus..Eubacteriumfissicatenagroup.id.14373 | | rs3771393 | | C | | T | | | 0.1308423 | | | 0.026667104 | | 7.38E-07 | | 0.0215367 | | | 0.028883 | | | 0.455875 | | |  |  |  |  |
| genus..Eubacteriumfissicatenagroup.id.14373 | | rs6934739 | | A | | G | | | 0.1114634 | | | 0.025278805 | | 9.75E-06 | | -0.0353684 | | | 0.024311 | | | 0.14572 | | |  |  |  |  |
| genus..Eubacteriumfissicatenagroup.id.14373 | | rs7104872 | | G | | A | | | 0.1386117 | | | 0.029190553 | | 2.73E-06 | | 0.0420057 | | | 0.036655 | | | 0.251809 | | |  |  |  |  |
| genus..Eubacteriumhalliigroup.id.11338 | | rs10501370 | | C | | T | | | -0.1155889 | | | 0.025248889 | | 5.42E-06 | | 0.0222129 | | | 0.046979 | | | 0.636338 | | |  |  |  |  |
| genus..Eubacteriumhalliigroup.id.11338 | | rs10798999 | | C | | T | | | 0.0601661 | | | 0.012675245 | | 2.61E-06 | | 0.0164498 | | | 0.02583 | | | 0.524229 | | |  |  |  |  |
| genus..Eubacteriumhalliigroup.id.11338 | | rs117748144 | | T | | C | | | -0.126582 | | | 0.028711955 | | 7.86E-06 | | 0.0113466 | | | 0.051696 | | | 0.826269 | | |  |  |  |  |
| genus..Eubacteriumhalliigroup.id.11338 | | rs13116360 | | T | | C | | | 0.1541244 | | | 0.029718533 | | 2.94E-07 | | 0.0260407 | | | 0.046195 | | | 0.572949 | | |  |  |  |  |
| genus..Eubacteriumhalliigroup.id.11338 | | rs17074066 | | T | | C | | | -0.0813986 | | | 0.018927232 | | 9.35E-06 | | -0.0437742 | | | 0.082063 | | | 0.59374 | | |  |  |  |  |
| genus..Eubacteriumhalliigroup.id.11338 | | rs17474256 | | G | | A | | | 0.0810805 | | | 0.018457309 | | 9.45E-06 | | 0.0167064 | | | 0.038466 | | | 0.664053 | | |  |  |  |  |
| genus..Eubacteriumhalliigroup.id.11338 | | rs281379 | | A | | G | | | -0.0499523 | | | 0.011215293 | | 9.33E-06 | | -0.0002004 | | | 0.023184 | | | 0.993104 | | |  |  |  |  |
| genus..Eubacteriumhalliigroup.id.11338 | | rs28584818 | | A | | G | | | 0.1261154 | | | 0.026862747 | | 4.43E-06 | | -0.0087892 | | | 0.042097 | | | 0.834615 | | |  |  |  |  |
| genus..Eubacteriumhalliigroup.id.11338 | | rs60254196 | | A | | G | | | -0.0522832 | | | 0.011186491 | | 2.70E-06 | | 0.003384 | | | 0.022975 | | | 0.8829 | | |  |  |  |  |
| genus..Eubacteriumhalliigroup.id.11338 | | rs630939 | | C | | T | | | -0.0508914 | | | 0.011435211 | | 9.16E-06 | | 0.0116529 | | | 0.023072 | | | 0.613518 | | |  |  |  |  |
| genus..Eubacteriumhalliigroup.id.11338 | | rs6550770 | | T | | C | | | -0.1980874 | | | 0.044354487 | | 4.82E-06 | | -0.0590903 | | | 0.0572 | | | 0.301582 | | |  |  |  |  |
| genus..Eubacteriumhalliigroup.id.11338 | | rs74018587 | | C | | T | | | 0.2089428 | | | 0.043821974 | | 3.70E-06 | | 0.055195 | | | 0.058005 | | | 0.341318 | | |  |  |  |  |
| genus..Eubacteriumhalliigroup.id.11338 | | rs78056098 | | G | | T | | | -0.0507411 | | | 0.011375822 | | 8.29E-06 | | 0.0389191 | | | 0.023793 | | | 0.101895 | | |  |  |  |  |
| genus..Eubacteriumhalliigroup.id.11338 | | rs949971 | | T | | G | | | -0.0540164 | | | 0.011610071 | | 3.29E-06 | | -0.0182885 | | | 0.024378 | | | 0.453139 | | |  |  |  |  |
| genus..Eubacteriumnodatumgroup.id.11297 | | rs10263623 | | C | | T | | | 0.1934959 | | | 0.043902338 | | 8.91E-06 | | 0.0391301 | | | 0.058928 | | | 0.506671 | | |  |  |  |  |
| genus..Eubacteriumnodatumgroup.id.11297 | | rs10458299 | | T | | C | | | -0.1877528 | | | 0.041969011 | | 8.37E-06 | | -0.0418989 | | | 0.043437 | | | 0.33475 | | |  |  |  |  |
| genus..Eubacteriumnodatumgroup.id.11297 | | rs11006576 | | A | | G | | | -0.1101671 | | | 0.024592963 | | 7.99E-06 | | -0.0002019 | | | 0.022976 | | | 0.99299 | | |  |  |  |  |
| genus..Eubacteriumnodatumgroup.id.11297 | | rs113893692 | | C | | T | | | -0.1851072 | | | 0.040357325 | | 5.76E-06 | | 0.0226715 | | | 0.034802 | | | 0.514766 | | |  |  |  |  |
| genus..Eubacteriumnodatumgroup.id.11297 | | rs34297067 | | A | | G | | | -0.1869065 | | | 0.034147492 | | 6.60E-08 | | -0.0114833 | | | 0.032332 | | | 0.722459 | | |  |  |  |  |
| genus..Eubacteriumnodatumgroup.id.11297 | | rs61841040 | | G | | T | | | 0.1606211 | | | 0.034160929 | | 3.56E-06 | | -0.0112171 | | | 0.028753 | | | 0.696448 | | |  |  |  |  |
| genus..Eubacteriumnodatumgroup.id.11297 | | rs6818880 | | A | | G | | | -0.1100806 | | | 0.024596445 | | 7.83E-06 | | -0.0168461 | | | 0.022996 | | | 0.463824 | | |  |  |  |  |
| genus..Eubacteriumnodatumgroup.id.11297 | | rs77910827 | | C | | T | | | 0.2017884 | | | 0.041356826 | | 9.05E-07 | | 0.0326301 | | | 0.036664 | | | 0.373483 | | |  |  |  |  |
| genus..Eubacteriumnodatumgroup.id.11297 | | rs7827125 | | C | | T | | | 0.1222998 | | | 0.027133143 | | 7.17E-06 | | -0.0109454 | | | 0.025487 | | | 0.667593 | | |  |  |  |  |
| genus..Eubacteriumnodatumgroup.id.11297 | | rs7880204 | | T | | C | | | -0.1254538 | | | 0.027530312 | | 6.84E-06 | | -0.023466 | | | 0.026515 | | | 0.376158 | | |  |  |  |  |
| genus..Eubacteriumnodatumgroup.id.11297 | | rs9425984 | | T | | C | | | -0.1302338 | | | 0.029234653 | | 7.21E-06 | | 0.0280448 | | | 0.027566 | | | 0.308968 | | |  |  |  |  |
| genus..Eubacteriumoxidoreducensgroup.id.11339 | | rs12129908 | | C | | A | | | 0.0893273 | | | 0.019833287 | | 5.80E-06 | | -0.0232797 | | | 0.023299 | | | 0.317718 | | |  |  |  |  |
| genus..Eubacteriumoxidoreducensgroup.id.11339 | | rs12423772 | | G | | T | | | 0.1409796 | | | 0.029502889 | | 2.63E-06 | | 0.0152318 | | | 0.032154 | | | 0.6357 | | |  |  |  |  |
| genus..Eubacteriumoxidoreducensgroup.id.11339 | | rs1425962 | | G | | C | | | 0.0906104 | | | 0.020061232 | | 7.32E-06 | | -0.0087384 | | | 0.02338 | | | 0.708592 | | |  |  |  |  |
| genus..Eubacteriumoxidoreducensgroup.id.11339 | | rs2973294 | | G | | T | | | 0.0923559 | | | 0.019543871 | | 2.39E-06 | | -0.0152911 | | | 0.023107 | | | 0.508126 | | |  |  |  |  |
| genus..Eubacteriumoxidoreducensgroup.id.11339 | | rs34561138 | | G | | A | | | 0.2161453 | | | 0.04599197 | | 2.51E-06 | | 0.0579837 | | | 0.059136 | | | 0.326833 | | |  |  |  |  |
| genus..Eubacteriumoxidoreducensgroup.id.11339 | | rs440215 | | C | | T | | | 0.0932766 | | | 0.019529391 | | 1.65E-06 | | 0.0197646 | | | 0.022996 | | | 0.390068 | | |  |  |  |  |
| genus..Eubacteriumrectalegroup.id.14374 | | rs10248854 | | C | | A | | | -0.0527827 | | | 0.01134635 | | 4.21E-06 | | 0.0203721 | | | 0.023376 | | | 0.38349 | | |  |  |  |  |
| genus..Eubacteriumrectalegroup.id.14374 | | rs10797540 | | A | | G | | | 0.0503179 | | | 0.010838946 | | 3.53E-06 | | 0.0473119 | | | 0.022987 | | | 0.0395667 | | |  |  |  |  |
| genus..Eubacteriumrectalegroup.id.14374 | | rs10892089 | | C | | G | | | -0.0635522 | | | 0.014007233 | | 6.22E-06 | | 0.0476788 | | | 0.02926 | | | 0.103212 | | |  |  |  |  |
| genus..Eubacteriumrectalegroup.id.14374 | | rs143694765 | | T | | C | | | 0.0870025 | | | 0.01977896 | | 9.75E-06 | | 0.0946076 | | | 0.037933 | | | 0.0126282 | | |  |  |  |  |
| genus..Eubacteriumrectalegroup.id.14374 | | rs16960159 | | C | | G | | | -0.156596 | | | 0.03362338 | | 4.10E-06 | | 0.0127236 | | | 0.038676 | | | 0.742173 | | |  |  |  |  |
| genus..Eubacteriumrectalegroup.id.14374 | | rs2884897 | | A | | G | | | -0.1293637 | | | 0.028902068 | | 6.44E-06 | | -0.0001878 | | | 0.062428 | | | 0.997599 | | |  |  |  |  |
| genus..Eubacteriumrectalegroup.id.14374 | | rs314726 | | T | | C | | | 0.0528824 | | | 0.010948624 | | 1.38E-06 | | -0.0268806 | | | 0.023029 | | | 0.243106 | | |  |  |  |  |
| genus..Eubacteriumrectalegroup.id.14374 | | rs35398954 | | A | | G | | | -0.0901402 | | | 0.017460171 | | 5.40E-07 | | -0.0223059 | | | 0.031178 | | | 0.474337 | | |  |  |  |  |
| genus..Eubacteriumrectalegroup.id.14374 | | rs3980709 | | A | | T | | | -0.0622701 | | | 0.014057159 | | 6.86E-06 | | 0.0055508 | | | 0.030062 | | | 0.853508 | | |  |  |  |  |
| genus..Eubacteriumrectalegroup.id.14374 | | rs58498416 | | C | | T | | | -0.0517316 | | | 0.011819644 | | 9.68E-06 | | 0.0071309 | | | 0.02387 | | | 0.765137 | | |  |  |  |  |
| genus..Eubacteriumrectalegroup.id.14374 | | rs59427698 | | A | | G | | | -0.0576035 | | | 0.013085337 | | 5.37E-06 | | -0.0236121 | | | 0.029243 | | | 0.419415 | | |  |  |  |  |
| genus..Eubacteriumrectalegroup.id.14374 | | rs62547233 | | A | | G | | | 0.0536319 | | | 0.012020063 | | 9.90E-06 | | -0.010464 | | | 0.025307 | | | 0.679254 | | |  |  |  |  |
| genus..Eubacteriumruminantiumgroup.id.11340 | | rs10131724 | | A | | C | | | -0.1998323 | | | 0.041457713 | | 2.39E-06 | | -0.0054832 | | | 0.038598 | | | 0.887033 | | |  |  |  |  |
| genus..Eubacteriumruminantiumgroup.id.11340 | | rs10923018 | | G | | A | | | 0.0726438 | | | 0.016092406 | | 6.80E-06 | | -0.0395862 | | | 0.023089 | | | 0.086441 | | |  |  |  |  |
| genus..Eubacteriumruminantiumgroup.id.11340 | | rs112375806 | | T | | A | | | 0.1431411 | | | 0.029375749 | | 5.82E-06 | | -0.0092737 | | | 0.033108 | | | 0.779397 | | |  |  |  |  |
| genus..Eubacteriumruminantiumgroup.id.11340 | | rs11637981 | | G | | T | | | -0.073258 | | | 0.016088796 | | 5.44E-06 | | 0.0056565 | | | 0.022996 | | | 0.805699 | | |  |  |  |  |
| genus..Eubacteriumruminantiumgroup.id.11340 | | rs13025464 | | T | | C | | | -0.0737077 | | | 0.016378577 | | 6.97E-06 | | -0.0258767 | | | 0.023314 | | | 0.267028 | | |  |  |  |  |
| genus..Eubacteriumruminantiumgroup.id.11340 | | rs139749 | | C | | T | | | -0.0845439 | | | 0.017179078 | | 8.59E-07 | | -0.0339818 | | | 0.024193 | | | 0.160137 | | |  |  |  |  |
| genus..Eubacteriumruminantiumgroup.id.11340 | | rs16891896 | | G | | A | | | -0.174787 | | | 0.039057268 | | 2.38E-06 | | -0.017388 | | | 0.040556 | | | 0.668112 | | |  |  |  |  |
| genus..Eubacteriumruminantiumgroup.id.11340 | | rs17519472 | | C | | T | | | 0.1078041 | | | 0.023398378 | | 4.70E-06 | | 0.0125004 | | | 0.033157 | | | 0.70617 | | |  |  |  |  |
| genus..Eubacteriumruminantiumgroup.id.11340 | | rs209813 | | G | | A | | | -0.1034881 | | | 0.023639057 | | 9.23E-06 | | 0.0137208 | | | 0.032386 | | | 0.671808 | | |  |  |  |  |
| genus..Eubacteriumruminantiumgroup.id.11340 | | rs2116427 | | A | | G | | | 0.0911461 | | | 0.018235307 | | 4.67E-07 | | -0.0081125 | | | 0.026202 | | | 0.756856 | | |  |  |  |  |
| genus..Eubacteriumruminantiumgroup.id.11340 | | rs2229917 | | A | | G | | | 0.1535379 | | | 0.032392215 | | 2.16E-06 | | -0.0665398 | | | 0.056985 | | | 0.24294 | | |  |  |  |  |
| genus..Eubacteriumruminantiumgroup.id.11340 | | rs2418654 | | C | | T | | | -0.0748879 | | | 0.016585161 | | 6.17E-06 | | -0.0229483 | | | 0.023293 | | | 0.324514 | | |  |  |  |  |
| genus..Eubacteriumruminantiumgroup.id.11340 | | rs2817174 | | C | | T | | | -0.0734306 | | | 0.016368685 | | 7.87E-06 | | -0.0081995 | | | 0.023366 | | | 0.725646 | | |  |  |  |  |
| genus..Eubacteriumruminantiumgroup.id.11340 | | rs57340348 | | T | | C | | | -0.0979429 | | | 0.021216595 | | 4.93E-06 | | -0.0150868 | | | 0.028539 | | | 0.59705 | | |  |  |  |  |
| genus..Eubacteriumruminantiumgroup.id.11340 | | rs606117 | | A | | G | | | 0.0833243 | | | 0.018055987 | | 4.82E-06 | | 0.0405289 | | | 0.025651 | | | 0.114104 | | |  |  |  |  |
| genus..Eubacteriumruminantiumgroup.id.11340 | | rs6676699 | | G | | T | | | -0.0888124 | | | 0.019644717 | | 6.38E-06 | | -0.0158333 | | | 0.024954 | | | 0.525753 | | |  |  |  |  |
| genus..Eubacteriumruminantiumgroup.id.11340 | | rs7000472 | | A | | G | | | -0.0762282 | | | 0.016523022 | | 4.07E-06 | | -0.0272267 | | | 0.023237 | | | 0.241311 | | |  |  |  |  |
| genus..Eubacteriumruminantiumgroup.id.11340 | | rs72836424 | | C | | T | | | -0.1398246 | | | 0.030068963 | | 2.62E-06 | | -0.0017354 | | | 0.036372 | | | 0.961945 | | |  |  |  |  |
| genus..Eubacteriumruminantiumgroup.id.11340 | | rs73139629 | | A | | C | | | -0.1150978 | | | 0.024790941 | | 5.36E-06 | | 0.0572633 | | | 0.039596 | | | 0.148122 | | |  |  |  |  |
| genus..Eubacteriumventriosumgroup.id.11341 | | rs11617697 | | A | | G | | | -0.1433485 | | | 0.028624073 | | 7.22E-07 | | 0.0223825 | | | 0.049942 | | | 0.654027 | | |  |  |  |  |
| genus..Eubacteriumventriosumgroup.id.11341 | | rs12964517 | | G | | A | | | 0.0587198 | | | 0.012345332 | | 2.07E-06 | | 0.0142521 | | | 0.025373 | | | 0.574324 | | |  |  |  |  |
| genus..Eubacteriumventriosumgroup.id.11341 | | rs13082419 | | C | | T | | | -0.0715522 | | | 0.016136969 | | 9.56E-06 | | 0.0084287 | | | 0.036393 | | | 0.816848 | | |  |  |  |  |
| genus..Eubacteriumventriosumgroup.id.11341 | | rs16884680 | | G | | T | | | -0.0906428 | | | 0.019190901 | | 1.74E-06 | | 0.07321 | | | 0.037835 | | | 0.0529932 | | |  |  |  |  |
| genus..Eubacteriumventriosumgroup.id.11341 | | rs35179274 | | C | | T | | | -0.0627263 | | | 0.013785975 | | 5.76E-06 | | -0.023851 | | | 0.029839 | | | 0.424097 | | |  |  |  |  |
| genus..Eubacteriumventriosumgroup.id.11341 | | rs3809430 | | T | | C | | | -0.0548171 | | | 0.011842566 | | 3.55E-06 | | -0.0082496 | | | 0.024721 | | | 0.738599 | | |  |  |  |  |
| genus..Eubacteriumventriosumgroup.id.11341 | | rs57199565 | | T | | C | | | 0.0783394 | | | 0.01602571 | | 7.97E-07 | | 0.0198264 | | | 0.029711 | | | 0.50457 | | |  |  |  |  |
| genus..Eubacteriumventriosumgroup.id.11341 | | rs6048195 | | A | | T | | | -0.0603594 | | | 0.011657994 | | 2.50E-07 | | 0.0173223 | | | 0.023722 | | | 0.465255 | | |  |  |  |  |
| genus..Eubacteriumventriosumgroup.id.11341 | | rs66746423 | | C | | T | | | 0.0751501 | | | 0.016497843 | | 6.11E-06 | | -0.0361818 | | | 0.031788 | | | 0.255027 | | |  |  |  |  |
| genus..Eubacteriumventriosumgroup.id.11341 | | rs6704822 | | A | | G | | | 0.0739179 | | | 0.016670495 | | 6.62E-06 | | 0.0165899 | | | 0.033836 | | | 0.623919 | | |  |  |  |  |
| genus..Eubacteriumventriosumgroup.id.11341 | | rs72783037 | | C | | A | | | 0.0658926 | | | 0.014374684 | | 6.55E-06 | | -0.0095306 | | | 0.028143 | | | 0.734878 | | |  |  |  |  |
| genus..Eubacteriumventriosumgroup.id.11341 | | rs73615400 | | T | | C | | | -0.0956303 | | | 0.01934276 | | 9.54E-07 | | 0.0221597 | | | 0.038464 | | | 0.564538 | | |  |  |  |  |
| genus..Eubacteriumventriosumgroup.id.11341 | | rs73849225 | | T | | C | | | 0.0975711 | | | 0.022438827 | | 5.21E-06 | | -0.0150239 | | | 0.04137 | | | 0.716486 | | |  |  |  |  |
| genus..Eubacteriumventriosumgroup.id.11341 | | rs78250280 | | G | | A | | | 0.0749762 | | | 0.016440448 | | 3.36E-06 | | -0.0321023 | | | 0.032883 | | | 0.328935 | | |  |  |  |  |
| genus..Eubacteriumventriosumgroup.id.11341 | | rs876734 | | C | | T | | | -0.0618696 | | | 0.013253231 | | 2.89E-06 | | 0.0217294 | | | 0.025542 | | | 0.394924 | | |  |  |  |  |
| genus..Eubacteriumventriosumgroup.id.11341 | | rs9316536 | | T | | G | | | -0.0817111 | | | 0.018312095 | | 7.84E-06 | | -0.0109821 | | | 0.033154 | | | 0.740462 | | |  |  |  |  |
| genus..Eubacteriumxylanophilumgroup.id.14375 | | rs10140184 | | A | | C | | | 0.0576795 | | | 0.01259899 | | 4.96E-06 | | -0.003766 | | | 0.023223 | | | 0.871172 | | |  |  |  |  |
| genus..Eubacteriumxylanophilumgroup.id.14375 | | rs10917203 | | A | | C | | | 0.0612633 | | | 0.013091688 | | 3.15E-06 | | -0.034587 | | | 0.023411 | | | 0.13957 | | |  |  |  |  |
| genus..Eubacteriumxylanophilumgroup.id.14375 | | rs112176119 | | C | | T | | | -0.1134555 | | | 0.024576196 | | 3.33E-06 | | -0.0377879 | | | 0.039235 | | | 0.33549 | | |  |  |  |  |
| genus..Eubacteriumxylanophilumgroup.id.14375 | | rs12980122 | | C | | G | | | -0.1082735 | | | 0.023825715 | | 5.02E-06 | | 0.0451025 | | | 0.044041 | | | 0.305782 | | |  |  |  |  |
| genus..Eubacteriumxylanophilumgroup.id.14375 | | rs13239072 | | G | | A | | | 0.0687427 | | | 0.014272832 | | 1.82E-06 | | 0.0182363 | | | 0.025573 | | | 0.475775 | | |  |  |  |  |
| genus..Eubacteriumxylanophilumgroup.id.14375 | | rs17830032 | | G | | A | | | -0.1605974 | | | 0.031051453 | | 2.39E-07 | | 0.0138509 | | | 0.042014 | | | 0.741645 | | |  |  |  |  |
| genus..Eubacteriumxylanophilumgroup.id.14375 | | rs1999224 | | G | | T | | | -0.0949051 | | | 0.020350281 | | 3.75E-06 | | -0.0097361 | | | 0.038311 | | | 0.799392 | | |  |  |  |  |
| genus..Eubacteriumxylanophilumgroup.id.14375 | | rs2012708 | | A | | G | | | 0.0573202 | | | 0.012690502 | | 6.53E-06 | | -0.0274851 | | | 0.024065 | | | 0.253405 | | |  |  |  |  |
| genus..Eubacteriumxylanophilumgroup.id.14375 | | rs2213117 | | T | | G | | | 0.0877797 | | | 0.018901228 | | 4.21E-06 | | 0.0106766 | | | 0.031272 | | | 0.732791 | | |  |  |  |  |
| genus..Eubacteriumxylanophilumgroup.id.14375 | | rs4654122 | | C | | G | | | 0.0553508 | | | 0.012358794 | | 7.20E-06 | | 0.0460312 | | | 0.022903 | | | 0.0444457 | | |  |  |  |  |
| genus..Eubacteriumxylanophilumgroup.id.14375 | | rs75586835 | | A | | G | | | -0.1144976 | | | 0.026345472 | | 9.39E-06 | | 0.016858 | | | 0.044703 | | | 0.706092 | | |  |  |  |  |
| genus..Eubacteriumxylanophilumgroup.id.14375 | | rs79582700 | | C | | G | | | -0.0951677 | | | 0.019996897 | | 2.41E-06 | | -0.0125358 | | | 0.034797 | | | 0.718654 | | |  |  |  |  |
| genus..Ruminococcusgauvreauiigroup.id.11342 | | rs10931481 | | G | | A | | | 0.0610096 | | | 0.013048446 | | 3.38E-06 | | -0.0183528 | | | 0.025138 | | | 0.465335 | | |  |  |  |  |
| genus..Ruminococcusgauvreauiigroup.id.11342 | | rs12079579 | | A | | G | | | 0.0955324 | | | 0.021343619 | | 5.04E-06 | | 0.014568 | | | 0.041089 | | | 0.722928 | | |  |  |  |  |
| genus..Ruminococcusgauvreauiigroup.id.11342 | | rs12539819 | | C | | T | | | 0.1106535 | | | 0.02406836 | | 4.49E-06 | | -0.0127635 | | | 0.045291 | | | 0.778089 | | |  |  |  |  |
| genus..Ruminococcusgauvreauiigroup.id.11342 | | rs13188803 | | T | | A | | | 0.0709419 | | | 0.015685519 | | 7.28E-06 | | -0.0082223 | | | 0.025617 | | | 0.748233 | | |  |  |  |  |
| genus..Ruminococcusgauvreauiigroup.id.11342 | | rs1391597 | | C | | T | | | 0.0590284 | | | 0.012487351 | | 1.86E-06 | | 0.0244496 | | | 0.023362 | | | 0.295298 | | |  |  |  |  |
| genus..Ruminococcusgauvreauiigroup.id.11342 | | rs2047242 | | A | | G | | | -0.067603 | | | 0.013373773 | | 2.46E-07 | | 0.0345698 | | | 0.027255 | | | 0.204655 | | |  |  |  |  |
| genus..Ruminococcusgauvreauiigroup.id.11342 | | rs2166943 | | A | | C | | | 0.0566974 | | | 0.012349673 | | 5.28E-06 | | 0.0059827 | | | 0.023123 | | | 0.795845 | | |  |  |  |  |
| genus..Ruminococcusgauvreauiigroup.id.11342 | | rs289410 | | G | | A | | | -0.0654926 | | | 0.013910104 | | 2.27E-06 | | -0.0313677 | | | 0.025398 | | | 0.216808 | | |  |  |  |  |
| genus..Ruminococcusgauvreauiigroup.id.11342 | | rs431418 | | A | | G | | | -0.094737 | | | 0.02101571 | | 5.54E-06 | | 0.0991989 | | | 0.039014 | | | 0.0110007 | | |  |  |  |  |
| genus..Ruminococcusgauvreauiigroup.id.11342 | | rs71386687 | | T | | G | | | 0.1210369 | | | 0.02385996 | | 2.91E-07 | | 0.0194549 | | | 0.036289 | | | 0.591882 | | |  |  |  |  |
| genus..Ruminococcusgauvreauiigroup.id.11342 | | rs73802842 | | C | | A | | | 0.0736808 | | | 0.016966478 | | 7.48E-06 | | -0.0386773 | | | 0.026892 | | | 0.150361 | | |  |  |  |  |
| genus..Ruminococcusgauvreauiigroup.id.11342 | | rs9870933 | | A | | G | | | 0.0621643 | | | 0.012607163 | | 8.49E-07 | | 0.0073174 | | | 0.023355 | | | 0.754048 | | |  |  |  |  |
| genus..Ruminococcusgnavusgroup.id.14376 | | rs11597105 | | A | | G | | | 0.1146728 | | | 0.025065269 | | 6.95E-06 | | 0.0370241 | | | 0.028786 | | | 0.198371 | | |  |  |  |  |
| genus..Ruminococcusgnavusgroup.id.14376 | | rs11864644 | | T | | C | | | -0.1398037 | | | 0.031825335 | | 5.01E-06 | | -0.0614374 | | | 0.035801 | | | 0.0861509 | | |  |  |  |  |
| genus..Ruminococcusgnavusgroup.id.14376 | | rs12136548 | | C | | T | | | 0.0901946 | | | 0.019647574 | | 3.10E-06 | | -0.0264765 | | | 0.025263 | | | 0.294612 | | |  |  |  |  |
| genus..Ruminococcusgnavusgroup.id.14376 | | rs12989336 | | G | | A | | | -0.0846861 | | | 0.01879361 | | 7.12E-06 | | 0.016084 | | | 0.025509 | | | 0.528352 | | |  |  |  |  |
| genus..Ruminococcusgnavusgroup.id.14376 | | rs13163520 | | G | | A | | | -0.1273775 | | | 0.023388325 | | 5.61E-08 | | -0.013267 | | | 0.029421 | | | 0.652037 | | |  |  |  |  |
| genus..Ruminococcusgnavusgroup.id.14376 | | rs2909242 | | C | | A | | | -0.0909965 | | | 0.018351012 | | 7.41E-07 | | 0.0018009 | | | 0.024273 | | | 0.940858 | | |  |  |  |  |
| genus..Ruminococcusgnavusgroup.id.14376 | | rs3124783 | | A | | G | | | -0.1159897 | | | 0.02490991 | | 2.67E-06 | | 0.002511 | | | 0.033987 | | | 0.941104 | | |  |  |  |  |
| genus..Ruminococcusgnavusgroup.id.14376 | | rs4388134 | | C | | T | | | -0.0904997 | | | 0.020354415 | | 9.12E-06 | | -0.0166731 | | | 0.025502 | | | 0.513249 | | |  |  |  |  |
| genus..Ruminococcusgnavusgroup.id.14376 | | rs62167033 | | T | | C | | | 0.185289 | | | 0.039629529 | | 3.50E-06 | | -0.0975092 | | | 0.057807 | | | 0.0916389 | | |  |  |  |  |
| genus..Ruminococcusgnavusgroup.id.14376 | | rs78399089 | | T | | C | | | 0.1444448 | | | 0.032661769 | | 6.63E-06 | | -0.0356145 | | | 0.035771 | | | 0.319432 | | |  |  |  |  |
| genus..Ruminococcusgnavusgroup.id.14376 | | rs934940 | | A | | C | | | -0.1050449 | | | 0.022958834 | | 2.74E-06 | | 0.0348844 | | | 0.033612 | | | 0.299339 | | |  |  |  |  |
| genus..Ruminococcustorquesgroup.id.14377 | | rs10904297 | | A | | G | | | -0.1678044 | | | 0.038981237 | | 2.69E-06 | | -0.249841 | | | 0.084845 | | | 0.00323296 | | |  |  |  |  |
| genus..Ruminococcustorquesgroup.id.14377 | | rs10967781 | | C | | A | | | 0.0507994 | | | 0.011332229 | | 8.37E-06 | | -0.0349686 | | | 0.025134 | | | 0.164136 | | |  |  |  |  |
| genus..Ruminococcustorquesgroup.id.14377 | | rs12434631 | | A | | G | | | 0.074697 | | | 0.015340053 | | 2.77E-06 | | 0.0417845 | | | 0.037123 | | | 0.26035 | | |  |  |  |  |
| genus..Ruminococcustorquesgroup.id.14377 | | rs13154778 | | T | | A | | | 0.0563358 | | | 0.012952644 | | 7.16E-06 | | -0.0307361 | | | 0.026344 | | | 0.243317 | | |  |  |  |  |
| genus..Ruminococcustorquesgroup.id.14377 | | rs1475330 | | T | | C | | | 0.0523492 | | | 0.011820049 | | 8.13E-06 | | -0.0310391 | | | 0.026853 | | | 0.247726 | | |  |  |  |  |
| genus..Ruminococcustorquesgroup.id.14377 | | rs158487 | | A | | G | | | 0.0531781 | | | 0.011634088 | | 7.52E-06 | | -0.0215217 | | | 0.025381 | | | 0.396462 | | |  |  |  |  |
| genus..Ruminococcustorquesgroup.id.14377 | | rs1972694 | | T | | A | | | -0.0614094 | | | 0.013732055 | | 8.93E-06 | | -0.0926444 | | | 0.030332 | | | 0.00225512 | | |  |  |  |  |
| genus..Ruminococcustorquesgroup.id.14377 | | rs35866622 | | T | | C | | | -0.0612024 | | | 0.010942125 | | 2.21E-08 | | 0.0060604 | | | 0.024092 | | | 0.801388 | | |  |  |  |  |
| genus..Ruminococcustorquesgroup.id.14377 | | rs4073731 | | T | | C | | | 0.0651948 | | | 0.014222118 | | 4.05E-06 | | 0.049097 | | | 0.030945 | | | 0.11261 | | |  |  |  |  |
| genus..Ruminococcustorquesgroup.id.14377 | | rs60603763 | | C | | G | | | 0.1243283 | | | 0.027528605 | | 5.87E-06 | | -0.0088359 | | | 0.041493 | | | 0.831367 | | |  |  |  |  |
| genus..Ruminococcustorquesgroup.id.14377 | | rs73130967 | | A | | T | | | 0.0770375 | | | 0.016826423 | | 3.71E-06 | | -0.0216083 | | | 0.041071 | | | 0.598806 | | |  |  |  |  |
| genus..Ruminococcustorquesgroup.id.14377 | | rs77034621 | | T | | G | | | -0.1515528 | | | 0.033587775 | | 6.07E-06 | | 0.12818 | | | 0.089472 | | | 0.151962 | | |  |  |  |  |
| genus..Ruminococcustorquesgroup.id.14377 | | rs773123 | | T | | A | | | 0.0824005 | | | 0.017386159 | | 1.59E-06 | | -0.0539038 | | | 0.034752 | | | 0.120879 | | |  |  |  |  |
| genus.Actinomyces.id.423 | | rs10787984 | | G | | C | | | 0.094316 | | | 0.021351309 | | 9.62E-06 | | 0.0259502 | | | 0.037808 | | | 0.492483 | | |  |  |  |  |
| genus.Actinomyces.id.423 | | rs2715439 | | C | | T | | | 0.0746684 | | | 0.016482184 | | 6.27E-06 | | 0.0210116 | | | 0.022848 | | | 0.357756 | | |  |  |  |  |
| genus.Actinomyces.id.423 | | rs34583783 | | G | | T | | | 0.1265958 | | | 0.026846063 | | 4.49E-06 | | -0.0863621 | | | 0.047838 | | | 0.0710281 | | |  |  |  |  |
| genus.Actinomyces.id.423 | | rs35011108 | | A | | G | | | 0.2326336 | | | 0.051204357 | | 6.34E-06 | | 0.0093052 | | | 0.045902 | | | 0.839354 | | |  |  |  |  |
| genus.Actinomyces.id.423 | | rs4073240 | | G | | A | | | 0.0749687 | | | 0.016736817 | | 7.94E-06 | | -0.009376 | | | 0.0235 | | | 0.689908 | | |  |  |  |  |
| genus.Actinomyces.id.423 | | rs4146653 | | G | | A | | | 0.0985224 | | | 0.021418242 | | 4.50E-06 | | -0.0377375 | | | 0.032778 | | | 0.249605 | | |  |  |  |  |
| genus.Actinomyces.id.423 | | rs71315246 | | A | | G | | | -0.0969809 | | | 0.021925041 | | 9.83E-06 | | -0.0190147 | | | 0.033313 | | | 0.568141 | | |  |  |  |  |
| genus.Actinomyces.id.423 | | rs7915461 | | T | | C | | | 0.1877598 | | | 0.040163611 | | 5.92E-06 | | -0.0343446 | | | 0.045356 | | | 0.448913 | | |  |  |  |  |
| genus.Adlercreutzia.id.812 | | rs1046175 | | C | | G | | | 0.1127579 | | | 0.025599407 | | 6.36E-06 | | -0.0109185 | | | 0.045107 | | | 0.808734 | | |  |  |  |  |
| genus.Adlercreutzia.id.812 | | rs11604400 | | C | | T | | | -0.1025242 | | | 0.023483368 | | 9.74E-06 | | -0.0479173 | | | 0.037194 | | | 0.197635 | | |  |  |  |  |
| genus.Adlercreutzia.id.812 | | rs12522517 | | A | | T | | | -0.1048844 | | | 0.023469475 | | 4.41E-06 | | -0.0287917 | | | 0.032414 | | | 0.374399 | | |  |  |  |  |
| genus.Adlercreutzia.id.812 | | rs13231526 | | C | | A | | | 0.1432371 | | | 0.031165443 | | 4.81E-06 | | 0.0436621 | | | 0.041836 | | | 0.296644 | | |  |  |  |  |
| genus.Adlercreutzia.id.812 | | rs2147798 | | C | | G | | | 0.0922959 | | | 0.019212124 | | 1.40E-06 | | -0.0156989 | | | 0.026384 | | | 0.551835 | | |  |  |  |  |
| genus.Adlercreutzia.id.812 | | rs2717140 | | C | | T | | | -0.1192252 | | | 0.025108114 | | 2.05E-06 | | 0.0221828 | | | 0.038139 | | | 0.560809 | | |  |  |  |  |
| genus.Adlercreutzia.id.812 | | rs55719207 | | G | | A | | | -0.0699243 | | | 0.015803609 | | 9.61E-06 | | 0.0423482 | | | 0.023432 | | | 0.0707213 | | |  |  |  |  |
| genus.Adlercreutzia.id.812 | | rs6664405 | | T | | C | | | -0.0953084 | | | 0.02107517 | | 5.23E-06 | | -0.0613026 | | | 0.032603 | | | 0.0600689 | | |  |  |  |  |
| genus.Adlercreutzia.id.812 | | rs7680684 | | C | | T | | | -0.083381 | | | 0.016890088 | | 9.77E-07 | | -0.0091384 | | | 0.024074 | | | 0.704241 | | |  |  |  |  |
| genus.Adlercreutzia.id.812 | | rs80078995 | | A | | T | | | -0.1132397 | | | 0.023258622 | | 1.57E-06 | | -0.0241309 | | | 0.032414 | | | 0.456601 | | |  |  |  |  |
| genus.Adlercreutzia.id.812 | | rs9490822 | | C | | T | | | -0.0734512 | | | 0.015578896 | | 2.54E-06 | | 0.0131064 | | | 0.022961 | | | 0.568124 | | |  |  |  |  |
| genus.Adlercreutzia.id.812 | | rs9915817 | | T | | C | | | 0.0749219 | | | 0.016833007 | | 8.22E-06 | | 0.0049651 | | | 0.025255 | | | 0.844144 | | |  |  |  |  |
| genus.Akkermansia.id.4037 | | rs11184341 | | G | | C | | | 0.0655855 | | | 0.01422364 | | 4.06E-06 | | -0.0085991 | | | 0.025614 | | | 0.737087 | | |  |  |  |  |
| genus.Akkermansia.id.4037 | | rs111862613 | | T | | C | | | 0.0911199 | | | 0.019674815 | | 3.39E-06 | | -0.0026351 | | | 0.030505 | | | 0.931162 | | |  |  |  |  |
| genus.Akkermansia.id.4037 | | rs117107102 | | A | | G | | | 0.2044062 | | | 0.043162894 | | 3.01E-06 | | -0.00267 | | | 0.054324 | | | 0.9608 | | |  |  |  |  |
| genus.Akkermansia.id.4037 | | rs11729256 | | T | | C | | | 0.0750473 | | | 0.015018395 | | 6.58E-07 | | 0.0694782 | | | 0.030455 | | | 0.0225279 | | |  |  |  |  |
| genus.Akkermansia.id.4037 | | rs12908520 | | G | | A | | | 0.061772 | | | 0.01309539 | | 2.26E-06 | | -0.0358032 | | | 0.023057 | | | 0.120472 | | |  |  |  |  |
| genus.Akkermansia.id.4037 | | rs2602429 | | C | | T | | | 0.0745352 | | | 0.015620127 | | 2.72E-06 | | -0.0046015 | | | 0.026067 | | | 0.859882 | | |  |  |  |  |
| genus.Akkermansia.id.4037 | | rs3995795 | | C | | T | | | 0.0641175 | | | 0.014099927 | | 5.22E-06 | | 0.0282219 | | | 0.023461 | | | 0.228996 | | |  |  |  |  |
| genus.Akkermansia.id.4037 | | rs4242783 | | G | | A | | | 0.0685454 | | | 0.014770062 | | 3.00E-06 | | -0.0188801 | | | 0.025441 | | | 0.458023 | | |  |  |  |  |
| genus.Akkermansia.id.4037 | | rs4936098 | | A | | G | | | 0.0649225 | | | 0.01359338 | | 1.10E-06 | | 0.0426866 | | | 0.023913 | | | 0.0742472 | | |  |  |  |  |
| genus.Akkermansia.id.4037 | | rs61779207 | | G | | A | | | -0.0760539 | | | 0.01677691 | | 6.32E-06 | | 0.0303851 | | | 0.02754 | | | 0.2699 | | |  |  |  |  |
| genus.Akkermansia.id.4037 | | rs74542928 | | T | | C | | | 0.1126227 | | | 0.023643438 | | 1.48E-06 | | -0.0190612 | | | 0.053899 | | | 0.723602 | | |  |  |  |  |
| genus.Akkermansia.id.4037 | | rs9349825 | | A | | G | | | -0.0703407 | | | 0.014713291 | | 2.60E-06 | | 0.0162513 | | | 0.029132 | | | 0.576947 | | |  |  |  |  |
| genus.Akkermansia.id.4037 | | rs941682 | | G | | A | | | -0.0632957 | | | 0.01437768 | | 9.17E-06 | | 0.0244884 | | | 0.025566 | | | 0.338136 | | |  |  |  |  |
| genus.Alistipes.id.968 | | rs1107244 | | G | | A | | | 0.0758558 | | | 0.017118221 | | 3.59E-06 | | -0.0181303 | | | 0.042629 | | | 0.670613 | | |  |  |  |  |
| genus.Alistipes.id.968 | | rs11769002 | | G | | A | | | -0.0528793 | | | 0.010938851 | | 1.45E-06 | | -0.0117427 | | | 0.023243 | | | 0.61341 | | |  |  |  |  |
| genus.Alistipes.id.968 | | rs11958296 | | A | | G | | | -0.0981222 | | | 0.021827201 | | 9.30E-06 | | 0.0436192 | | | 0.05547 | | | 0.431657 | | |  |  |  |  |
| genus.Alistipes.id.968 | | rs12990744 | | C | | T | | | -0.0776618 | | | 0.017298561 | | 8.21E-06 | | 0.008157 | | | 0.037678 | | | 0.828603 | | |  |  |  |  |
| genus.Alistipes.id.968 | | rs1689282 | | A | | C | | | -0.0520061 | | | 0.011394196 | | 5.28E-06 | | 0.0263454 | | | 0.024444 | | | 0.281127 | | |  |  |  |  |
| genus.Alistipes.id.968 | | rs2290844 | | C | | T | | | 0.0813969 | | | 0.019163185 | | 9.10E-06 | | -0.0462412 | | | 0.036373 | | | 0.203623 | | |  |  |  |  |
| genus.Alistipes.id.968 | | rs2450745 | | A | | C | | | -0.080524 | | | 0.018470178 | | 7.12E-06 | | 0.0248543 | | | 0.044604 | | | 0.577379 | | |  |  |  |  |
| genus.Alistipes.id.968 | | rs2875322 | | T | | C | | | -0.0580945 | | | 0.013143238 | | 8.78E-06 | | 0.0215617 | | | 0.030479 | | | 0.479302 | | |  |  |  |  |
| genus.Alistipes.id.968 | | rs34417064 | | A | | G | | | -0.048202 | | | 0.010686843 | | 7.01E-06 | | 0.011466 | | | 0.022966 | | | 0.617601 | | |  |  |  |  |
| genus.Alistipes.id.968 | | rs4810359 | | A | | G | | | -0.0652428 | | | 0.014615527 | | 7.50E-06 | | 0.0170893 | | | 0.034804 | | | 0.623413 | | |  |  |  |  |
| genus.Alistipes.id.968 | | rs67281112 | | G | | C | | | 0.0634575 | | | 0.013784589 | | 3.92E-06 | | -0.0236325 | | | 0.028505 | | | 0.407075 | | |  |  |  |  |
| genus.Alistipes.id.968 | | rs7129639 | | C | | A | | | -0.0524955 | | | 0.010958377 | | 1.78E-06 | | -0.0203239 | | | 0.024239 | | | 0.401764 | | |  |  |  |  |
| genus.Alistipes.id.968 | | rs8130320 | | A | | G | | | -0.049002 | | | 0.01071701 | | 4.84E-06 | | 0.0228607 | | | 0.022957 | | | 0.319342 | | |  |  |  |  |
| genus.Allisonella.id.2174 | | rs1901739 | | T | | G | | | 0.1157685 | | | 0.024862618 | | 3.59E-06 | | -0.0291968 | | | 0.022912 | | | 0.202546 | | |  |  |  |  |
| genus.Allisonella.id.2174 | | rs35110698 | | T | | C | | | -0.146322 | | | 0.032085207 | | 5.72E-06 | | 0.0051009 | | | 0.032478 | | | 0.875199 | | |  |  |  |  |
| genus.Allisonella.id.2174 | | rs35778461 | | C | | T | | | 0.1466787 | | | 0.029718557 | | 1.21E-06 | | -0.0410795 | | | 0.027196 | | | 0.130913 | | |  |  |  |  |
| genus.Allisonella.id.2174 | | rs594561 | | C | | T | | | 0.1122306 | | | 0.025168069 | | 9.41E-06 | | -0.017383 | | | 0.022826 | | | 0.446335 | | |  |  |  |  |
| genus.Allisonella.id.2174 | | rs602075 | | A | | G | | | 0.1689743 | | | 0.029697566 | | 3.57E-08 | | -0.0131684 | | | 0.025952 | | | 0.611866 | | |  |  |  |  |
| genus.Allisonella.id.2174 | | rs6742198 | | G | | A | | | 0.149152 | | | 0.031648438 | | 3.35E-06 | | -0.0388102 | | | 0.026852 | | | 0.148359 | | |  |  |  |  |
| genus.Allisonella.id.2174 | | rs685403 | | G | | C | | | -0.1752032 | | | 0.040447416 | | 4.88E-06 | | 0.0606884 | | | 0.033656 | | | 0.0713576 | | |  |  |  |  |
| genus.Allisonella.id.2174 | | rs76904847 | | G | | A | | | 0.1485235 | | | 0.033485691 | | 6.09E-06 | | 0.0163627 | | | 0.030267 | | | 0.588772 | | |  |  |  |  |
| genus.Allisonella.id.2174 | | rs7898615 | | T | | G | | | 0.1679664 | | | 0.037358631 | | 8.87E-06 | | -0.0043627 | | | 0.03325 | | | 0.895611 | | |  |  |  |  |
| genus.Alloprevotella.id.961 | | rs17380632 | | A | | T | | | 0.1255638 | | | 0.028040851 | | 9.27E-06 | | -0.0299095 | | | 0.026909 | | | 0.266357 | | |  |  |  |  |
| genus.Alloprevotella.id.961 | | rs2154444 | | T | | G | | | 0.1382343 | | | 0.030896071 | | 8.37E-06 | | -0.0422624 | | | 0.025943 | | | 0.103305 | | |  |  |  |  |
| genus.Alloprevotella.id.961 | | rs34619204 | | G | | A | | | -0.1560369 | | | 0.034439247 | | 8.84E-06 | | -0.056198 | | | 0.030207 | | | 0.0628261 | | |  |  |  |  |
| genus.Alloprevotella.id.961 | | rs4364940 | | A | | G | | | 0.1264177 | | | 0.028220735 | | 8.58E-06 | | -0.0185488 | | | 0.024696 | | | 0.452596 | | |  |  |  |  |
| genus.Alloprevotella.id.961 | | rs4680035 | | A | | G | | | -0.1195994 | | | 0.025944593 | | 4.99E-06 | | -0.0540306 | | | 0.02342 | | | 0.0210528 | | |  |  |  |  |
| genus.Alloprevotella.id.961 | | rs58212166 | | A | | G | | | -0.1615059 | | | 0.035910233 | | 7.94E-06 | | 0.0635992 | | | 0.029507 | | | 0.0311329 | | |  |  |  |  |
| genus.Anaerofilum.id.2053 | | rs10794359 | | T | | C | | | -0.0953524 | | | 0.020059979 | | 2.23E-06 | | -0.0373823 | | | 0.023135 | | | 0.106133 | | |  |  |  |  |
| genus.Anaerofilum.id.2053 | | rs1563175 | | A | | C | | | 0.0923834 | | | 0.020215708 | | 5.54E-06 | | -0.0028244 | | | 0.022882 | | | 0.901763 | | |  |  |  |  |
| genus.Anaerofilum.id.2053 | | rs17012738 | | T | | G | | | 0.0903417 | | | 0.020023943 | | 7.24E-06 | | 0.0079548 | | | 0.022855 | | | 0.727802 | | |  |  |  |  |
| genus.Anaerofilum.id.2053 | | rs17096874 | | C | | T | | | -0.1263369 | | | 0.02689189 | | 2.86E-06 | | -0.0324714 | | | 0.028033 | | | 0.246727 | | |  |  |  |  |
| genus.Anaerofilum.id.2053 | | rs17105491 | | G | | C | | | -0.1931074 | | | 0.04111866 | | 1.57E-06 | | -0.0367237 | | | 0.047233 | | | 0.436865 | | |  |  |  |  |
| genus.Anaerofilum.id.2053 | | rs4244069 | | G | | A | | | -0.1467745 | | | 0.032659424 | | 9.81E-06 | | 0.043345 | | | 0.034579 | | | 0.210018 | | |  |  |  |  |
| genus.Anaerofilum.id.2053 | | rs4506496 | | G | | A | | | 0.1030899 | | | 0.021286122 | | 1.49E-06 | | -0.0088848 | | | 0.025144 | | | 0.723821 | | |  |  |  |  |
| genus.Anaerofilum.id.2053 | | rs712981 | | A | | C | | | 0.1007596 | | | 0.020290248 | | 6.83E-07 | | 0.0369234 | | | 0.023436 | | | 0.115146 | | |  |  |  |  |
| genus.Anaerofilum.id.2053 | | rs79598899 | | C | | T | | | 0.1825858 | | | 0.035728447 | | 3.75E-07 | | -0.0106302 | | | 0.054649 | | | 0.845772 | | |  |  |  |  |
| genus.Anaerofilum.id.2053 | | rs816292 | | T | | C | | | -0.1130026 | | | 0.022040046 | | 2.64E-07 | | 0.0440428 | | | 0.025166 | | | 0.0800958 | | |  |  |  |  |
| genus.Anaerofilum.id.2053 | | rs9299345 | | T | | C | | | -0.1363995 | | | 0.030237163 | | 8.04E-06 | | -0.0174254 | | | 0.038633 | | | 0.651956 | | |  |  |  |  |
| genus.Anaerostipes.id.1991 | | rs10502061 | | A | | G | | | 0.0835752 | | | 0.019201667 | | 7.94E-06 | | -0.0365226 | | | 0.035856 | | | 0.308396 | | |  |  |  |  |
| genus.Anaerostipes.id.1991 | | rs13376554 | | A | | T | | | 0.197389 | | | 0.04586424 | | 7.72E-06 | | 0.0540815 | | | 0.073113 | | | 0.459486 | | |  |  |  |  |
| genus.Anaerostipes.id.1991 | | rs2014785 | | T | | C | | | 0.0515687 | | | 0.011219863 | | 4.68E-06 | | -0.0159922 | | | 0.023216 | | | 0.490915 | | |  |  |  |  |
| genus.Anaerostipes.id.1991 | | rs2396460 | | T | | C | | | -0.0512653 | | | 0.010955542 | | 2.91E-06 | | -0.0056569 | | | 0.022978 | | | 0.805542 | | |  |  |  |  |
| genus.Anaerostipes.id.1991 | | rs2804244 | | A | | G | | | -0.053089 | | | 0.011098265 | | 2.04E-06 | | 0.0061496 | | | 0.023459 | | | 0.793215 | | |  |  |  |  |
| genus.Anaerostipes.id.1991 | | rs3900776 | | G | | A | | | -0.1100132 | | | 0.023630171 | | 2.75E-06 | | 0.0916483 | | | 0.069249 | | | 0.185685 | | |  |  |  |  |
| genus.Anaerostipes.id.1991 | | rs60983350 | | G | | A | | | -0.0539901 | | | 0.011657526 | | 4.42E-06 | | -0.0470471 | | | 0.024383 | | | 0.0536723 | | |  |  |  |  |
| genus.Anaerostipes.id.1991 | | rs62157625 | | T | | C | | | 0.088573 | | | 0.018554786 | | 1.45E-06 | | 0.0107238 | | | 0.035312 | | | 0.761367 | | |  |  |  |  |
| genus.Anaerostipes.id.1991 | | rs62215703 | | G | | A | | | 0.0644965 | | | 0.013670195 | | 1.98E-06 | | -0.018352 | | | 0.027762 | | | 0.50858 | | |  |  |  |  |
| genus.Anaerostipes.id.1991 | | rs6474958 | | A | | G | | | -0.0500065 | | | 0.011200088 | | 6.74E-06 | | 0.0041576 | | | 0.024652 | | | 0.866067 | | |  |  |  |  |
| genus.Anaerostipes.id.1991 | | rs6726833 | | C | | A | | | -0.0877497 | | | 0.018940832 | | 3.32E-06 | | -0.0668469 | | | 0.042187 | | | 0.113068 | | |  |  |  |  |
| genus.Anaerostipes.id.1991 | | rs6854026 | | T | | C | | | -0.0508482 | | | 0.010907505 | | 3.20E-06 | | -0.0408674 | | | 0.023083 | | | 0.0766549 | | |  |  |  |  |
| genus.Anaerostipes.id.1991 | | rs7193624 | | C | | T | | | 0.0750642 | | | 0.015072346 | | 5.35E-07 | | -0.0068199 | | | 0.041687 | | | 0.870047 | | |  |  |  |  |
| genus.Anaerostipes.id.1991 | | rs7823228 | | G | | C | | | -0.0621085 | | | 0.013661403 | | 5.84E-06 | | -0.0270661 | | | 0.030423 | | | 0.373647 | | |  |  |  |  |
| genus.Anaerostipes.id.1991 | | rs78735375 | | A | | C | | | -0.1374286 | | | 0.030530436 | | 5.33E-06 | | -0.0289661 | | | 0.05821 | | | 0.618754 | | |  |  |  |  |
| genus.Anaerotruncus.id.2054 | | rs10150232 | | A | | G | | | 0.0567088 | | | 0.012487702 | | 6.68E-06 | | -0.0506961 | | | 0.028582 | | | 0.0761062 | | |  |  |  |  |
| genus.Anaerotruncus.id.2054 | | rs11018566 | | A | | G | | | -0.1564652 | | | 0.036603226 | | 6.14E-06 | | -0.105791 | | | 0.050743 | | | 0.037086 | | |  |  |  |  |
| genus.Anaerotruncus.id.2054 | | rs115414803 | | A | | C | | | -0.1443559 | | | 0.031752395 | | 6.83E-06 | | -0.034813 | | | 0.047564 | | | 0.464215 | | |  |  |  |  |
| genus.Anaerotruncus.id.2054 | | rs12056802 | | C | | G | | | 0.0774194 | | | 0.017717789 | | 6.13E-06 | | -0.0139608 | | | 0.034215 | | | 0.683246 | | |  |  |  |  |
| genus.Anaerotruncus.id.2054 | | rs1272208 | | G | | T | | | -0.0611743 | | | 0.012983073 | | 4.28E-06 | | -0.010665 | | | 0.026931 | | | 0.692092 | | |  |  |  |  |
| genus.Anaerotruncus.id.2054 | | rs1431492 | | C | | T | | | -0.0654996 | | | 0.014618811 | | 7.36E-06 | | 0.0523077 | | | 0.031405 | | | 0.095801 | | |  |  |  |  |
| genus.Anaerotruncus.id.2054 | | rs17734739 | | T | | C | | | 0.0660052 | | | 0.014908026 | | 7.43E-06 | | -0.0118084 | | | 0.032903 | | | 0.719681 | | |  |  |  |  |
| genus.Anaerotruncus.id.2054 | | rs2704155 | | T | | A | | | -0.1062347 | | | 0.023481456 | | 5.42E-06 | | -0.0029607 | | | 0.057549 | | | 0.958969 | | |  |  |  |  |
| genus.Anaerotruncus.id.2054 | | rs34449434 | | A | | C | | | -0.0497004 | | | 0.011340216 | | 9.85E-06 | | 0.0349536 | | | 0.023932 | | | 0.144136 | | |  |  |  |  |
| genus.Anaerotruncus.id.2054 | | rs4669806 | | G | | T | | | 0.0576389 | | | 0.012299403 | | 2.42E-06 | | 0.0303598 | | | 0.027825 | | | 0.275235 | | |  |  |  |  |
| genus.Anaerotruncus.id.2054 | | rs6494922 | | A | | G | | | 0.0903106 | | | 0.020225652 | | 6.62E-06 | | -0.036481 | | | 0.050573 | | | 0.470694 | | |  |  |  |  |
| genus.Anaerotruncus.id.2054 | | rs6563550 | | T | | C | | | 0.0877135 | | | 0.017674473 | | 2.35E-07 | | -0.0194642 | | | 0.04263 | | | 0.647969 | | |  |  |  |  |
| genus.Anaerotruncus.id.2054 | | rs7155595 | | C | | A | | | 0.0539336 | | | 0.011890292 | | 7.55E-06 | | 0.0048702 | | | 0.024889 | | | 0.84486 | | |  |  |  |  |
| genus.Anaerotruncus.id.2054 | | rs7675045 | | A | | T | | | -0.0497815 | | | 0.01105734 | | 6.92E-06 | | -0.0415729 | | | 0.022875 | | | 0.0691512 | | |  |  |  |  |
| genus.Anaerotruncus.id.2054 | | rs8005030 | | C | | T | | | 0.0554447 | | | 0.01178543 | | 2.28E-06 | | -0.0022423 | | | 0.02429 | | | 0.926446 | | |  |  |  |  |
| genus.Anaerotruncus.id.2054 | | rs9347879 | | T | | C | | | 0.050618 | | | 0.011049001 | | 4.22E-06 | | 0.012403 | | | 0.022817 | | | 0.586721 | | |  |  |  |  |
| genus.Bacteroides.id.918 | | rs11585893 | | A | | G | | | -0.0740746 | | | 0.014763325 | | 1.80E-06 | | -0.028401 | | | 0.026769 | | | 0.288703 | | |  |  |  |  |
| genus.Bacteroides.id.918 | | rs13207588 | | A | | G | | | -0.059205 | | | 0.013119468 | | 7.49E-06 | | -0.0092706 | | | 0.029021 | | | 0.749388 | | |  |  |  |  |
| genus.Bacteroides.id.918 | | rs1340391 | | T | | C | | | -0.0592004 | | | 0.013224416 | | 6.73E-06 | | -0.0331014 | | | 0.033391 | | | 0.321523 | | |  |  |  |  |
| genus.Bacteroides.id.918 | | rs17619981 | | T | | G | | | 0.0880978 | | | 0.018700207 | | 2.69E-06 | | 0.0136854 | | | 0.033462 | | | 0.682548 | | |  |  |  |  |
| genus.Bacteroides.id.918 | | rs2023437 | | T | | C | | | -0.0782325 | | | 0.016763399 | | 5.02E-06 | | -0.0140548 | | | 0.034754 | | | 0.685913 | | |  |  |  |  |
| genus.Bacteroides.id.918 | | rs2366421 | | T | | A | | | -0.0528153 | | | 0.011712437 | | 7.65E-06 | | 0.0679123 | | | 0.02986 | | | 0.0229446 | | |  |  |  |  |
| genus.Bacteroides.id.918 | | rs28757219 | | T | | A | | | 0.0818398 | | | 0.017031866 | | 1.29E-06 | | -0.0629617 | | | 0.034906 | | | 0.0712656 | | |  |  |  |  |
| genus.Bacteroides.id.918 | | rs495004 | | C | | G | | | -0.0607475 | | | 0.012986536 | | 3.42E-06 | | 0.038252 | | | 0.027302 | | | 0.161199 | | |  |  |  |  |
| genus.Bacteroides.id.918 | | rs66710942 | | C | | T | | | 0.0488038 | | | 0.010741209 | | 5.86E-06 | | 0.0046007 | | | 0.022898 | | | 0.840762 | | |  |  |  |  |
| genus.Bacteroides.id.918 | | rs6795673 | | C | | T | | | 0.0538565 | | | 0.010525127 | | 3.38E-07 | | 0.0102397 | | | 0.022811 | | | 0.653507 | | |  |  |  |  |
| genus.Bacteroides.id.918 | | rs9507307 | | C | | T | | | 0.0604456 | | | 0.012912793 | | 2.13E-06 | | -0.0216115 | | | 0.02669 | | | 0.418103 | | |  |  |  |  |
| genus.Barnesiella.id.944 | | rs11155559 | | T | | C | | | 0.0956023 | | | 0.021296077 | | 8.92E-06 | | 0.0201851 | | | 0.039518 | | | 0.609499 | | |  |  |  |  |
| genus.Barnesiella.id.944 | | rs12909713 | | C | | T | | | -0.0550716 | | | 0.012003894 | | 4.95E-06 | | 0.0015212 | | | 0.022835 | | | 0.946888 | | |  |  |  |  |
| genus.Barnesiella.id.944 | | rs13242616 | | T | | C | | | -0.0583747 | | | 0.012317572 | | 2.29E-06 | | -0.0053185 | | | 0.024635 | | | 0.82907 | | |  |  |  |  |
| genus.Barnesiella.id.944 | | rs199035 | | G | | A | | | 0.0559495 | | | 0.011971558 | | 3.00E-06 | | -0.0410327 | | | 0.023006 | | | 0.0744921 | | |  |  |  |  |
| genus.Barnesiella.id.944 | | rs2057922 | | G | | C | | | 0.0915241 | | | 0.019462277 | | 3.83E-06 | | -0.0158673 | | | 0.031057 | | | 0.609419 | | |  |  |  |  |
| genus.Barnesiella.id.944 | | rs2276875 | | A | | G | | | -0.0697176 | | | 0.013953704 | | 4.65E-07 | | -0.0314858 | | | 0.026374 | | | 0.232551 | | |  |  |  |  |
| genus.Barnesiella.id.944 | | rs2428166 | | G | | A | | | -0.1658587 | | | 0.033730907 | | 8.51E-07 | | -0.0023981 | | | 0.097808 | | | 0.980439 | | |  |  |  |  |
| genus.Barnesiella.id.944 | | rs28418786 | | C | | G | | | -0.0791324 | | | 0.017495701 | | 6.48E-06 | | 0.0184792 | | | 0.036586 | | | 0.613495 | | |  |  |  |  |
| genus.Barnesiella.id.944 | | rs28479800 | | A | | T | | | -0.09152 | | | 0.021000228 | | 7.93E-06 | | 0.0236825 | | | 0.036465 | | | 0.51604 | | |  |  |  |  |
| genus.Barnesiella.id.944 | | rs35177866 | | A | | G | | | 0.0917125 | | | 0.019013343 | | 2.95E-06 | | 0.021239 | | | 0.043585 | | | 0.626041 | | |  |  |  |  |
| genus.Barnesiella.id.944 | | rs62251337 | | A | | G | | | -0.0690763 | | | 0.014937412 | | 4.24E-06 | | -0.0195045 | | | 0.032734 | | | 0.551273 | | |  |  |  |  |
| genus.Barnesiella.id.944 | | rs72684847 | | T | | C | | | -0.1143693 | | | 0.025387327 | | 6.76E-06 | | 0.0265021 | | | 0.043021 | | | 0.537872 | | |  |  |  |  |
| genus.Barnesiella.id.944 | | rs76181748 | | C | | T | | | -0.0778559 | | | 0.017163996 | | 6.78E-06 | | -0.0108101 | | | 0.027267 | | | 0.69177 | | |  |  |  |  |
| genus.Barnesiella.id.944 | | rs77455852 | | T | | G | | | -0.0891491 | | | 0.019560057 | | 3.16E-06 | | -0.0589177 | | | 0.032465 | | | 0.0695553 | | |  |  |  |  |
| genus.Barnesiella.id.944 | | rs79795328 | | A | | G | | | -0.0818645 | | | 0.01764407 | | 4.23E-06 | | -0.0053086 | | | 0.032946 | | | 0.871991 | | |  |  |  |  |
| genus.Bifidobacterium.id.436 | | rs10841473 | | G | | C | | | -0.0624207 | | | 0.012943849 | | 1.65E-06 | | 0.0294895 | | | 0.027161 | | | 0.277603 | | |  |  |  |  |
| genus.Bifidobacterium.id.436 | | rs12022129 | | G | | A | | | 0.0619356 | | | 0.013893747 | | 8.00E-06 | | -0.0336653 | | | 0.025612 | | | 0.188692 | | |  |  |  |  |
| genus.Bifidobacterium.id.436 | | rs12465599 | | A | | G | | | 0.0535787 | | | 0.011821744 | | 6.27E-06 | | 0.0238213 | | | 0.022873 | | | 0.297667 | | |  |  |  |  |
| genus.Bifidobacterium.id.436 | | rs13020688 | | G | | A | | | 0.0562696 | | | 0.012261748 | | 4.07E-06 | | 0.0588948 | | | 0.024886 | | | 0.0179506 | | |  |  |  |  |
| genus.Bifidobacterium.id.436 | | rs182549 | | C | | T | | | 0.1197035 | | | 0.012729374 | | 1.28E-20 | | -0.0034943 | | | 0.02351 | | | 0.881845 | | |  |  |  |  |
| genus.Bifidobacterium.id.436 | | rs1879087 | | C | | T | | | 0.105365 | | | 0.024052943 | | 1.45E-06 | | -0.0454609 | | | 0.044965 | | | 0.312 | | |  |  |  |  |
| genus.Bifidobacterium.id.436 | | rs1961273 | | C | | T | | | 0.0674036 | | | 0.013231896 | | 3.51E-07 | | 0.0037966 | | | 0.025154 | | | 0.880025 | | |  |  |  |  |
| genus.Bifidobacterium.id.436 | | rs2491158 | | G | | A | | | 0.0712624 | | | 0.015983004 | | 8.05E-06 | | 0.0481881 | | | 0.03358 | | | 0.151282 | | |  |  |  |  |
| genus.Bifidobacterium.id.436 | | rs2686790 | | T | | C | | | 0.070741 | | | 0.015792595 | | 7.50E-06 | | 0.0264131 | | | 0.032202 | | | 0.412081 | | |  |  |  |  |
| genus.Bifidobacterium.id.436 | | rs4567981 | | T | | A | | | 0.0562084 | | | 0.011792309 | | 1.93E-06 | | 0.0444613 | | | 0.022976 | | | 0.0529761 | | |  |  |  |  |
| genus.Bifidobacterium.id.436 | | rs540489 | | T | | G | | | -0.0637641 | | | 0.013874591 | | 5.19E-06 | | 0.0053002 | | | 0.029967 | | | 0.859609 | | |  |  |  |  |
| genus.Bifidobacterium.id.436 | | rs55888705 | | A | | G | | | 0.0546319 | | | 0.012113865 | | 6.67E-06 | | 0.0032694 | | | 0.025395 | | | 0.89756 | | |  |  |  |  |
| genus.Bifidobacterium.id.436 | | rs56108664 | | T | | C | | | 0.0729971 | | | 0.015792589 | | 2.44E-06 | | -0.0081408 | | | 0.033984 | | | 0.81068 | | |  |  |  |  |
| genus.Bifidobacterium.id.436 | | rs5746486 | | T | | C | | | -0.0536216 | | | 0.012080057 | | 9.00E-06 | | -0.0373209 | | | 0.023428 | | | 0.111156 | | |  |  |  |  |
| genus.Bifidobacterium.id.436 | | rs62181700 | | G | | A | | | -0.0624643 | | | 0.013120526 | | 2.17E-06 | | -0.0237241 | | | 0.02659 | | | 0.372272 | | |  |  |  |  |
| genus.Bifidobacterium.id.436 | | rs638382 | | T | | C | | | -0.0650864 | | | 0.012300762 | | 9.45E-08 | | 0.0404807 | | | 0.023036 | | | 0.078866 | | |  |  |  |  |
| genus.Bifidobacterium.id.436 | | rs6430601 | | T | | G | | | 0.0796261 | | | 0.017031321 | | 1.09E-06 | | 0.0073997 | | | 0.035277 | | | 0.833854 | | |  |  |  |  |
| genus.Bifidobacterium.id.436 | | rs7322849 | | T | | C | | | 0.1124285 | | | 0.020181262 | | 1.08E-08 | | -0.0168992 | | | 0.039915 | | | 0.672015 | | |  |  |  |  |
| genus.Bifidobacterium.id.436 | | rs75344046 | | C | | T | | | 0.2323541 | | | 0.050597867 | | 4.86E-06 | | 0.0374115 | | | 0.054151 | | | 0.489644 | | |  |  |  |  |
| genus.Bifidobacterium.id.436 | | rs7570971 | | A | | C | | | 0.1133352 | | | 0.012574753 | | 2.01E-19 | | -0.004112 | | | 0.023421 | | | 0.860634 | | |  |  |  |  |
| genus.Bifidobacterium.id.436 | | rs76671854 | | C | | G | | | -0.0846055 | | | 0.01840032 | | 3.96E-06 | | -0.0378798 | | | 0.034685 | | | 0.274784 | | |  |  |  |  |
| genus.Bifidobacterium.id.436 | | rs857444 | | C | | T | | | 0.0558234 | | | 0.012121924 | | 3.57E-06 | | -0.000304 | | | 0.023699 | | | 0.989764 | | |  |  |  |  |
| genus.Bilophila.id.3170 | | rs11069458 | | T | | C | | | -0.0680947 | | | 0.015502996 | | 7.72E-06 | | -0.0002584 | | | 0.029534 | | | 0.993019 | | |  |  |  |  |
| genus.Bilophila.id.3170 | | rs116261629 | | G | | C | | | 0.1281123 | | | 0.026135828 | | 8.62E-07 | | 0.0185901 | | | 0.039366 | | | 0.636757 | | |  |  |  |  |
| genus.Bilophila.id.3170 | | rs1241171 | | G | | A | | | -0.0692654 | | | 0.015014825 | | 4.24E-06 | | -0.0140546 | | | 0.032463 | | | 0.665054 | | |  |  |  |  |
| genus.Bilophila.id.3170 | | rs1571225 | | C | | T | | | 0.0826826 | | | 0.017062147 | | 1.12E-06 | | 0.0062708 | | | 0.030765 | | | 0.838485 | | |  |  |  |  |
| genus.Bilophila.id.3170 | | rs1969927 | | G | | A | | | 0.0564589 | | | 0.012693476 | | 9.07E-06 | | -0.0061576 | | | 0.024213 | | | 0.799254 | | |  |  |  |  |
| genus.Bilophila.id.3170 | | rs2713349 | | A | | T | | | 0.0617228 | | | 0.014017424 | | 8.63E-06 | | 0.0379369 | | | 0.02506 | | | 0.130064 | | |  |  |  |  |
| genus.Bilophila.id.3170 | | rs2728491 | | G | | T | | | -0.0627416 | | | 0.013945646 | | 6.33E-06 | | -0.0441625 | | | 0.026734 | | | 0.098553 | | |  |  |  |  |
| genus.Bilophila.id.3170 | | rs3827020 | | C | | T | | | 0.0766479 | | | 0.016064223 | | 1.79E-06 | | -0.0209959 | | | 0.028746 | | | 0.465153 | | |  |  |  |  |
| genus.Bilophila.id.3170 | | rs4798126 | | G | | A | | | 0.073288 | | | 0.016798984 | | 7.15E-06 | | 0.0163709 | | | 0.028801 | | | 0.56976 | | |  |  |  |  |
| genus.Bilophila.id.3170 | | rs542415 | | T | | C | | | -0.0613623 | | | 0.013343594 | | 4.71E-06 | | 0.0382727 | | | 0.023424 | | | 0.102281 | | |  |  |  |  |
| genus.Bilophila.id.3170 | | rs60178956 | | G | | A | | | -0.0624818 | | | 0.014148499 | | 8.06E-06 | | 0.0067449 | | | 0.027293 | | | 0.804807 | | |  |  |  |  |
| genus.Bilophila.id.3170 | | rs6793291 | | C | | A | | | 0.112729 | | | 0.024163294 | | 3.11E-06 | | 0.039055 | | | 0.051899 | | | 0.45174 | | |  |  |  |  |
| genus.Bilophila.id.3170 | | rs72676854 | | T | | C | | | 0.1232088 | | | 0.026882038 | | 5.62E-06 | | -0.0003664 | | | 0.052047 | | | 0.994383 | | |  |  |  |  |
| genus.Bilophila.id.3170 | | rs7802841 | | C | | A | | | 0.0670097 | | | 0.013770056 | | 1.77E-06 | | -0.0214984 | | | 0.024893 | | | 0.387783 | | |  |  |  |  |
| genus.Bilophila.id.3170 | | rs8013541 | | A | | T | | | -0.0571991 | | | 0.01256729 | | 5.48E-06 | | -0.0267801 | | | 0.02303 | | | 0.244902 | | |  |  |  |  |
| genus.Bilophila.id.3170 | | rs9899990 | | A | | G | | | -0.1027023 | | | 0.023387821 | | 9.07E-06 | | 0.0397987 | | | 0.041349 | | | 0.335796 | | |  |  |  |  |
| genus.Blautia.id.1992 | | rs11149971 | | C | | T | | | 0.1176047 | | | 0.023389975 | | 1.04E-06 | | -0.0731319 | | | 0.050247 | | | 0.145541 | | |  |  |  |  |
| genus.Blautia.id.1992 | | rs115043014 | | G | | A | | | -0.2066054 | | | 0.043991974 | | 5.19E-06 | | -0.0447026 | | | 0.088429 | | | 0.613194 | | |  |  |  |  |
| genus.Blautia.id.1992 | | rs117001700 | | T | | C | | | 0.1964143 | | | 0.044112197 | | 8.84E-06 | | -0.0367774 | | | 0.097951 | | | 0.707312 | | |  |  |  |  |
| genus.Blautia.id.1992 | | rs12453000 | | C | | T | | | 0.0625335 | | | 0.012998711 | | 1.26E-06 | | 0.0108219 | | | 0.033332 | | | 0.745429 | | |  |  |  |  |
| genus.Blautia.id.1992 | | rs16892041 | | T | | C | | | -0.0622605 | | | 0.014180398 | | 8.82E-06 | | 0.0281463 | | | 0.027857 | | | 0.31231 | | |  |  |  |  |
| genus.Blautia.id.1992 | | rs2788271 | | T | | G | | | -0.0575596 | | | 0.013349169 | | 7.16E-06 | | -2.84E-05 | | | 0.030096 | | | 0.999246 | | |  |  |  |  |
| genus.Blautia.id.1992 | | rs3005511 | | A | | G | | | 0.0501085 | | | 0.01107676 | | 6.19E-06 | | -0.0473362 | | | 0.024902 | | | 0.0573113 | | |  |  |  |  |
| genus.Blautia.id.1992 | | rs4926264 | | T | | C | | | 0.0826207 | | | 0.017823907 | | 5.10E-06 | | 0.058098 | | | 0.037216 | | | 0.118499 | | |  |  |  |  |
| genus.Blautia.id.1992 | | rs67794373 | | C | | T | | | 0.0601709 | | | 0.012344281 | | 1.00E-06 | | 0.0530805 | | | 0.027389 | | | 0.0526163 | | |  |  |  |  |
| genus.Blautia.id.1992 | | rs682885 | | A | | G | | | -0.0493376 | | | 0.010735883 | | 4.49E-06 | | -0.029779 | | | 0.024442 | | | 0.223085 | | |  |  |  |  |
| genus.Blautia.id.1992 | | rs72973581 | | A | | G | | | 0.1251691 | | | 0.026540793 | | 1.74E-06 | | -0.0686516 | | | 0.049071 | | | 0.161807 | | |  |  |  |  |
| genus.Blautia.id.1992 | | rs7860714 | | A | | G | | | -0.0502178 | | | 0.010985269 | | 4.09E-06 | | 0.0112189 | | | 0.023912 | | | 0.63894 | | |  |  |  |  |
| genus.Butyricicoccus.id.2055 | | rs10084203 | | A | | G | | | 0.0549699 | | | 0.012356262 | | 8.59E-06 | | 0.0095615 | | | 0.034034 | | | 0.778759 | | |  |  |  |  |
| genus.Butyricicoccus.id.2055 | | rs12034718 | | A | | G | | | 0.0701199 | | | 0.01582135 | | 9.58E-06 | | -0.0321368 | | | 0.027446 | | | 0.241625 | | |  |  |  |  |
| genus.Butyricicoccus.id.2055 | | rs12585793 | | T | | C | | | -0.2622062 | | | 0.056472877 | | 5.79E-06 | | -0.0198186 | | | 0.068731 | | | 0.773079 | | |  |  |  |  |
| genus.Butyricicoccus.id.2055 | | rs2017189 | | G | | T | | | -0.0506956 | | | 0.011024022 | | 3.87E-06 | | 0.0254834 | | | 0.022874 | | | 0.265254 | | |  |  |  |  |
| genus.Butyricicoccus.id.2055 | | rs4962426 | | G | | T | | | 0.0614216 | | | 0.013597912 | | 7.38E-06 | | -0.0036999 | | | 0.028845 | | | 0.897936 | | |  |  |  |  |
| genus.Butyricicoccus.id.2055 | | rs56221232 | | T | | C | | | 0.0828027 | | | 0.016740066 | | 7.62E-07 | | -0.0420066 | | | 0.038056 | | | 0.26967 | | |  |  |  |  |
| genus.Butyricicoccus.id.2055 | | rs62478070 | | T | | G | | | 0.2240393 | | | 0.049495932 | | 5.94E-06 | | -0.0490611 | | | 0.072421 | | | 0.498126 | | |  |  |  |  |
| genus.Butyricicoccus.id.2055 | | rs7322368 | | T | | C | | | 0.0815733 | | | 0.018316651 | | 5.52E-06 | | 0.0373259 | | | 0.039887 | | | 0.349379 | | |  |  |  |  |
| genus.Butyricicoccus.id.2055 | | rs75238760 | | T | | A | | | 0.0619423 | | | 0.013994184 | | 6.80E-06 | | -0.0184645 | | | 0.033913 | | | 0.586115 | | |  |  |  |  |
| genus.Butyricimonas.id.945 | | rs11228830 | | A | | G | | | 0.1352649 | | | 0.029841132 | | 6.55E-06 | | -0.0686596 | | | 0.040752 | | | 0.0920216 | | |  |  |  |  |
| genus.Butyricimonas.id.945 | | rs113054641 | | G | | A | | | -0.144881 | | | 0.027457379 | | 1.74E-07 | | 0.0247345 | | | 0.055528 | | | 0.656002 | | |  |  |  |  |
| genus.Butyricimonas.id.945 | | rs12304031 | | G | | A | | | -0.086278 | | | 0.019684629 | | 6.70E-06 | | 0.0134545 | | | 0.034758 | | | 0.698692 | | |  |  |  |  |
| genus.Butyricimonas.id.945 | | rs12458763 | | A | | C | | | 0.1220366 | | | 0.026958664 | | 6.37E-06 | | -0.0254405 | | | 0.055117 | | | 0.644387 | | |  |  |  |  |
| genus.Butyricimonas.id.945 | | rs1701950 | | G | | C | | | -0.1050556 | | | 0.024104658 | | 7.68E-06 | | -0.0174693 | | | 0.039465 | | | 0.658017 | | |  |  |  |  |
| genus.Butyricimonas.id.945 | | rs1862649 | | G | | A | | | 0.113124 | | | 0.024817639 | | 4.76E-06 | | -0.0780988 | | | 0.045322 | | | 0.0848535 | | |  |  |  |  |
| genus.Butyricimonas.id.945 | | rs2114713 | | G | | T | | | 0.062721 | | | 0.013899768 | | 6.88E-06 | | -0.0268053 | | | 0.022969 | | | 0.243196 | | |  |  |  |  |
| genus.Butyricimonas.id.945 | | rs2642760 | | G | | C | | | 0.0713357 | | | 0.014539571 | | 8.58E-07 | | 0.0195608 | | | 0.02478 | | | 0.429884 | | |  |  |  |  |
| genus.Butyricimonas.id.945 | | rs270727 | | G | | C | | | -0.0694173 | | | 0.015079545 | | 5.38E-06 | | 0.0225315 | | | 0.024504 | | | 0.35783 | | |  |  |  |  |
| genus.Butyricimonas.id.945 | | rs326049 | | C | | G | | | 0.0759694 | | | 0.01676173 | | 8.19E-06 | | 0.0017386 | | | 0.025988 | | | 0.946662 | | |  |  |  |  |
| genus.Butyricimonas.id.945 | | rs62130338 | | G | | A | | | -0.0732608 | | | 0.015832388 | | 3.90E-06 | | -0.0185531 | | | 0.024082 | | | 0.441057 | | |  |  |  |  |
| genus.Butyricimonas.id.945 | | rs62390301 | | T | | C | | | -0.0872983 | | | 0.017489288 | | 7.42E-07 | | 0.0247286 | | | 0.028592 | | | 0.387098 | | |  |  |  |  |
| genus.Butyricimonas.id.945 | | rs7083431 | | A | | C | | | 0.0703539 | | | 0.014443868 | | 8.85E-07 | | -0.0067562 | | | 0.025694 | | | 0.792591 | | |  |  |  |  |
| genus.Butyricimonas.id.945 | | rs71428626 | | G | | T | | | -0.1331884 | | | 0.028990548 | | 4.80E-06 | | 0.0415536 | | | 0.064548 | | | 0.519728 | | |  |  |  |  |
| genus.Butyricimonas.id.945 | | rs72814525 | | A | | G | | | 0.0664154 | | | 0.01497662 | | 8.25E-06 | | 0.0104609 | | | 0.026662 | | | 0.6948 | | |  |  |  |  |
| genus.Butyricimonas.id.945 | | rs782080 | | T | | A | | | 0.0653488 | | | 0.01379735 | | 2.23E-06 | | -0.002731 | | | 0.023232 | | | 0.906423 | | |  |  |  |  |
| genus.Butyricimonas.id.945 | | rs78453362 | | A | | G | | | -0.1494802 | | | 0.032707281 | | 4.06E-06 | | 0.0061378 | | | 0.073508 | | | 0.933455 | | |  |  |  |  |
| genus.Butyricimonas.id.945 | | rs9657374 | | C | | T | | | 0.0680719 | | | 0.014817248 | | 4.50E-06 | | 0.0026252 | | | 0.025031 | | | 0.916471 | | |  |  |  |  |
| genus.Butyrivibrio.id.1993 | | rs1007475 | | G | | T | | | 0.1180654 | | | 0.026127086 | | 7.92E-06 | | -0.0394774 | | | 0.025548 | | | 0.122289 | | |  |  |  |  |
| genus.Butyrivibrio.id.1993 | | rs11761679 | | T | | C | | | 0.1547754 | | | 0.032153939 | | 2.20E-06 | | -0.0172632 | | | 0.032944 | | | 0.600272 | | |  |  |  |  |
| genus.Butyrivibrio.id.1993 | | rs142855850 | | A | | G | | | 0.2050132 | | | 0.045713757 | | 6.86E-06 | | -0.0362419 | | | 0.038924 | | | 0.351807 | | |  |  |  |  |
| genus.Butyrivibrio.id.1993 | | rs16934069 | | T | | C | | | -0.1337842 | | | 0.029944364 | | 8.86E-06 | | 0.0050236 | | | 0.030003 | | | 0.867024 | | |  |  |  |  |
| genus.Butyrivibrio.id.1993 | | rs16941336 | | C | | T | | | 0.1274875 | | | 0.026793591 | | 1.53E-06 | | 0.0041767 | | | 0.027237 | | | 0.878126 | | |  |  |  |  |
| genus.Butyrivibrio.id.1993 | | rs17163238 | | G | | A | | | 0.1409869 | | | 0.030911712 | | 5.51E-06 | | 0.0091853 | | | 0.02896 | | | 0.751116 | | |  |  |  |  |
| genus.Butyrivibrio.id.1993 | | rs4537857 | | T | | C | | | -0.1245897 | | | 0.026099163 | | 1.80E-06 | | 0.0222368 | | | 0.024174 | | | 0.357636 | | |  |  |  |  |
| genus.Butyrivibrio.id.1993 | | rs486484 | | A | | G | | | -0.1083487 | | | 0.024000793 | | 6.61E-06 | | -0.047173 | | | 0.023156 | | | 0.0416284 | | |  |  |  |  |
| genus.Butyrivibrio.id.1993 | | rs4928024 | | A | | G | | | -0.17471 | | | 0.038933257 | | 8.19E-06 | | 0.0347012 | | | 0.029476 | | | 0.239093 | | |  |  |  |  |
| genus.Butyrivibrio.id.1993 | | rs72723662 | | C | | T | | | 0.2240782 | | | 0.044933642 | | 7.86E-07 | | -0.0265249 | | | 0.033242 | | | 0.424914 | | |  |  |  |  |
| genus.Butyrivibrio.id.1993 | | rs7412979 | | C | | G | | | 0.1867786 | | | 0.038911205 | | 1.71E-06 | | 0.0155513 | | | 0.041787 | | | 0.709777 | | |  |  |  |  |
| genus.Butyrivibrio.id.1993 | | rs74622183 | | A | | G | | | -0.2010274 | | | 0.042820448 | | 2.46E-06 | | 0.0384091 | | | 0.0401 | | | 0.33815 | | |  |  |  |  |
| genus.Butyrivibrio.id.1993 | | rs77356209 | | T | | C | | | 0.2168745 | | | 0.04832994 | | 6.66E-06 | | 0.0234934 | | | 0.054304 | | | 0.665284 | | |  |  |  |  |
| genus.Butyrivibrio.id.1993 | | rs7752361 | | A | | G | | | -0.1192303 | | | 0.023997488 | | 7.69E-07 | | 0.0002858 | | | 0.022848 | | | 0.990019 | | |  |  |  |  |
| genus.Butyrivibrio.id.1993 | | rs7763512 | | G | | A | | | 0.1198457 | | | 0.025336711 | | 3.11E-06 | | -0.0169167 | | | 0.0231 | | | 0.463975 | | |  |  |  |  |
| genus.Butyrivibrio.id.1993 | | rs9349693 | | A | | G | | | 0.1179727 | | | 0.025986063 | | 5.55E-06 | | -0.005441 | | | 0.025122 | | | 0.828533 | | |  |  |  |  |
| genus.CandidatusSoleaferrea.id.11350 | | rs10090365 | | A | | G | | | -0.0834413 | | | 0.018098882 | | 4.17E-06 | | -0.019916 | | | 0.0228 | | | 0.382387 | | |  |  |  |  |
| genus.CandidatusSoleaferrea.id.11350 | | rs10809135 | | T | | C | | | 0.0834858 | | | 0.01824263 | | 5.47E-06 | | -0.0188434 | | | 0.023237 | | | 0.417418 | | |  |  |  |  |
| genus.CandidatusSoleaferrea.id.11350 | | rs11153159 | | G | | C | | | -0.12805 | | | 0.028544611 | | 4.42E-06 | | -0.0199957 | | | 0.032555 | | | 0.539069 | | |  |  |  |  |
| genus.CandidatusSoleaferrea.id.11350 | | rs12500231 | | A | | T | | | 0.0813853 | | | 0.018269552 | | 7.68E-06 | | 0.0137381 | | | 0.02294 | | | 0.549263 | | |  |  |  |  |
| genus.CandidatusSoleaferrea.id.11350 | | rs2193878 | | T | | A | | | 0.2282551 | | | 0.050924135 | | 9.46E-06 | | -0.0102111 | | | 0.073869 | | | 0.890056 | | |  |  |  |  |
| genus.CandidatusSoleaferrea.id.11350 | | rs36155147 | | C | | T | | | 0.1049661 | | | 0.024099527 | | 5.41E-06 | | 0.021263 | | | 0.024538 | | | 0.386189 | | |  |  |  |  |
| genus.CandidatusSoleaferrea.id.11350 | | rs386526 | | C | | G | | | 0.0818459 | | | 0.018008617 | | 8.33E-06 | | -0.0071635 | | | 0.023118 | | | 0.756662 | | |  |  |  |  |
| genus.CandidatusSoleaferrea.id.11350 | | rs4294381 | | T | | C | | | 0.1121965 | | | 0.023186007 | | 1.37E-06 | | 0.0387468 | | | 0.03131 | | | 0.215893 | | |  |  |  |  |
| genus.CandidatusSoleaferrea.id.11350 | | rs4678258 | | T | | C | | | 0.0986136 | | | 0.021564627 | | 5.53E-06 | | 0.0160888 | | | 0.026765 | | | 0.547762 | | |  |  |  |  |
| genus.CandidatusSoleaferrea.id.11350 | | rs6489992 | | A | | G | | | -0.0840437 | | | 0.018703073 | | 7.89E-06 | | -0.0022572 | | | 0.02364 | | | 0.923932 | | |  |  |  |  |
| genus.CandidatusSoleaferrea.id.11350 | | rs6494306 | | A | | G | | | -0.0969304 | | | 0.021422086 | | 5.80E-06 | | 0.0418304 | | | 0.02439 | | | 0.0863316 | | |  |  |  |  |
| genus.CandidatusSoleaferrea.id.11350 | | rs6881988 | | G | | C | | | -0.0819131 | | | 0.018185036 | | 9.23E-06 | | 0.0090863 | | | 0.022899 | | | 0.691519 | | |  |  |  |  |
| genus.CandidatusSoleaferrea.id.11350 | | rs7400877 | | T | | C | | | -0.0951085 | | | 0.021279907 | | 9.29E-06 | | 0.0350377 | | | 0.027839 | | | 0.208172 | | |  |  |  |  |
| genus.CandidatusSoleaferrea.id.11350 | | rs830149 | | C | | G | | | 0.1846466 | | | 0.039723748 | | 9.58E-06 | | 0.0093502 | | | 0.04823 | | | 0.84628 | | |  |  |  |  |
| genus.CandidatusSoleaferrea.id.11350 | | rs9973954 | | A | | G | | | 0.0892075 | | | 0.019540149 | | 5.95E-06 | | 0.0146664 | | | 0.023962 | | | 0.54049 | | |  |  |  |  |
| genus.Catenibacterium.id.2153 | | rs12404911 | | C | | T | | | 0.1407172 | | | 0.030414222 | | 2.80E-06 | | -0.0139546 | | | 0.029407 | | | 0.635114 | | |  |  |  |  |
| genus.Catenibacterium.id.2153 | | rs212393 | | G | | A | | | -0.1352539 | | | 0.02862088 | | 3.62E-06 | | 0.0126587 | | | 0.028196 | | | 0.653461 | | |  |  |  |  |
| genus.Catenibacterium.id.2153 | | rs73128290 | | A | | G | | | 0.1297262 | | | 0.02845626 | | 4.29E-06 | | 0.0053054 | | | 0.024887 | | | 0.831188 | | |  |  |  |  |
| genus.Catenibacterium.id.2153 | | rs7742829 | | C | | T | | | 0.1141101 | | | 0.025109895 | | 5.61E-06 | | 0.0109982 | | | 0.023022 | | | 0.632842 | | |  |  |  |  |
| genus.ChristensenellaceaeR.7group.id.11283 | | rs10461257 | | A | | G | | | -0.0551969 | | | 0.01220692 | | 6.51E-06 | | 0.009504 | | | 0.024206 | | | 0.694591 | | |  |  |  |  |
| genus.ChristensenellaceaeR.7group.id.11283 | | rs17081797 | | A | | G | | | -0.0904325 | | | 0.020425314 | | 3.34E-06 | | -0.0097802 | | | 0.046533 | | | 0.833528 | | |  |  |  |  |
| genus.ChristensenellaceaeR.7group.id.11283 | | rs62132810 | | A | | G | | | -0.0828853 | | | 0.017962272 | | 5.67E-06 | | -0.0581921 | | | 0.032865 | | | 0.0766231 | | |  |  |  |  |
| genus.ChristensenellaceaeR.7group.id.11283 | | rs62190261 | | A | | C | | | 0.0958394 | | | 0.021472282 | | 8.74E-06 | | -0.0243882 | | | 0.040817 | | | 0.550168 | | |  |  |  |  |
| genus.ChristensenellaceaeR.7group.id.11283 | | rs62467127 | | C | | T | | | 0.1141083 | | | 0.025198272 | | 3.25E-06 | | -0.0172766 | | | 0.073536 | | | 0.814255 | | |  |  |  |  |
| genus.ChristensenellaceaeR.7group.id.11283 | | rs73952017 | | C | | T | | | -0.0862164 | | | 0.019435393 | | 8.46E-06 | | -0.0380066 | | | 0.037544 | | | 0.31138 | | |  |  |  |  |
| genus.ChristensenellaceaeR.7group.id.11283 | | rs78521377 | | C | | T | | | 0.1249918 | | | 0.027479689 | | 5.61E-06 | | 0.0543046 | | | 0.066379 | | | 0.413301 | | |  |  |  |  |
| genus.ChristensenellaceaeR.7group.id.11283 | | rs892686 | | A | | G | | | 0.0514099 | | | 0.011135975 | | 3.97E-06 | | 0.003663 | | | 0.022867 | | | 0.872733 | | |  |  |  |  |
| genus.ChristensenellaceaeR.7group.id.11283 | | rs999354 | | T | | A | | | 0.0580248 | | | 0.011718514 | | 7.01E-07 | | 0.0191613 | | | 0.023583 | | | 0.416493 | | |  |  |  |  |
| genus.Clostridiumsensustricto1.id.1873 | | rs11264403 | | G | | A | | | -0.1390615 | | | 0.033445425 | | 7.76E-06 | | -0.0676424 | | | 0.043255 | | | 0.117863 | | |  |  |  |  |
| genus.Clostridiumsensustricto1.id.1873 | | rs11586026 | | A | | T | | | 0.1109668 | | | 0.025049788 | | 8.85E-06 | | -0.0403183 | | | 0.063614 | | | 0.526214 | | |  |  |  |  |
| genus.Clostridiumsensustricto1.id.1873 | | rs116847295 | | C | | T | | | 0.1100214 | | | 0.024603155 | | 4.58E-06 | | 0.0019275 | | | 0.03418 | | | 0.95503 | | |  |  |  |  |
| genus.Clostridiumsensustricto1.id.1873 | | rs12341505 | | G | | A | | | 0.0810718 | | | 0.018012111 | | 4.82E-06 | | 0.0019844 | | | 0.039344 | | | 0.959774 | | |  |  |  |  |
| genus.Clostridiumsensustricto1.id.1873 | | rs12490337 | | C | | G | | | -0.0616879 | | | 0.0137581 | | 7.49E-06 | | 0.0472995 | | | 0.02848 | | | 0.0967542 | | |  |  |  |  |
| genus.Clostridiumsensustricto1.id.1873 | | rs2795528 | | G | | A | | | -0.1843147 | | | 0.039220419 | | 2.72E-06 | | -0.0102137 | | | 0.049502 | | | 0.836533 | | |  |  |  |  |
| genus.Clostridiumsensustricto1.id.1873 | | rs2817172 | | C | | T | | | 0.058139 | | | 0.012449219 | | 2.77E-06 | | 0.0096393 | | | 0.023323 | | | 0.67939 | | |  |  |  |  |
| genus.Clostridiumsensustricto1.id.1873 | | rs550843 | | T | | C | | | -0.0783246 | | | 0.016920943 | | 2.05E-06 | | 0.0285299 | | | 0.025487 | | | 0.262969 | | |  |  |  |  |
| genus.Collinsella.id.815 | | rs10890671 | | T | | C | | | -0.0537401 | | | 0.011884842 | | 6.52E-06 | | 0.0073621 | | | 0.02303 | | | 0.749209 | | |  |  |  |  |
| genus.Collinsella.id.815 | | rs11597285 | | G | | T | | | -0.0537947 | | | 0.012054849 | | 9.38E-06 | | 0.0072476 | | | 0.02334 | | | 0.756162 | | |  |  |  |  |
| genus.Collinsella.id.815 | | rs12921100 | | A | | T | | | 0.0563759 | | | 0.012701892 | | 8.23E-06 | | -0.0291051 | | | 0.025733 | | | 0.258037 | | |  |  |  |  |
| genus.Collinsella.id.815 | | rs1496626 | | T | | C | | | -0.0722307 | | | 0.016160046 | | 6.78E-06 | | -0.0436528 | | | 0.033613 | | | 0.194055 | | |  |  |  |  |
| genus.Collinsella.id.815 | | rs149807560 | | C | | A | | | -0.1042642 | | | 0.023584097 | | 7.10E-06 | | 0.0270225 | | | 0.045082 | | | 0.548897 | | |  |  |  |  |
| genus.Collinsella.id.815 | | rs2103510 | | G | | A | | | 0.0786551 | | | 0.016821672 | | 2.42E-06 | | 0.0273608 | | | 0.035691 | | | 0.44331 | | |  |  |  |  |
| genus.Collinsella.id.815 | | rs2671662 | | C | | G | | | -0.0567751 | | | 0.011944997 | | 2.22E-06 | | -0.010992 | | | 0.022988 | | | 0.632541 | | |  |  |  |  |
| genus.Collinsella.id.815 | | rs59414781 | | C | | G | | | 0.0668866 | | | 0.014977291 | | 9.15E-06 | | -0.0177943 | | | 0.033552 | | | 0.595862 | | |  |  |  |  |
| genus.Collinsella.id.815 | | rs62102630 | | A | | G | | | -0.0696299 | | | 0.015332482 | | 8.31E-06 | | -0.0274396 | | | 0.033799 | | | 0.416877 | | |  |  |  |  |
| genus.Collinsella.id.815 | | rs62448871 | | C | | A | | | -0.0540309 | | | 0.012032414 | | 6.78E-06 | | 0.0133778 | | | 0.023143 | | | 0.563224 | | |  |  |  |  |
| genus.Collinsella.id.815 | | rs73052258 | | G | | A | | | 0.0930178 | | | 0.020265842 | | 1.72E-06 | | -0.0065301 | | | 0.042892 | | | 0.878992 | | |  |  |  |  |
| genus.Collinsella.id.815 | | rs75672793 | | A | | G | | | -0.108908 | | | 0.024052154 | | 6.14E-06 | | -0.122386 | | | 0.054241 | | | 0.0240503 | | |  |  |  |  |
| genus.Collinsella.id.815 | | rs9541268 | | C | | A | | | 0.09597 | | | 0.019731218 | | 8.79E-07 | | 0.0479887 | | | 0.039949 | | | 0.229659 | | |  |  |  |  |
| genus.Coprobacter.id.949 | | rs11532348 | | C | | T | | | -0.1039306 | | | 0.022682638 | | 5.71E-06 | | -0.0224301 | | | 0.032109 | | | 0.484828 | | |  |  |  |  |
| genus.Coprobacter.id.949 | | rs12684609 | | T | | C | | | 0.100827 | | | 0.022036643 | | 6.10E-06 | | -0.0124066 | | | 0.028703 | | | 0.665563 | | |  |  |  |  |
| genus.Coprobacter.id.949 | | rs12996055 | | A | | C | | | 0.0921817 | | | 0.020940499 | | 8.08E-06 | | 0.0470108 | | | 0.026004 | | | 0.0706334 | | |  |  |  |  |
| genus.Coprobacter.id.949 | | rs143662916 | | C | | T | | | 0.2532639 | | | 0.05401412 | | 3.07E-06 | | 0.070969 | | | 0.067114 | | | 0.290311 | | |  |  |  |  |
| genus.Coprobacter.id.949 | | rs189356 | | G | | A | | | 0.0781125 | | | 0.017191637 | | 6.26E-06 | | -0.0157701 | | | 0.023106 | | | 0.49491 | | |  |  |  |  |
| genus.Coprobacter.id.949 | | rs213863 | | C | | T | | | -0.0887412 | | | 0.018837727 | | 2.35E-06 | | 0.034828 | | | 0.023793 | | | 0.143248 | | |  |  |  |  |
| genus.Coprobacter.id.949 | | rs28402691 | | T | | C | | | 0.1106954 | | | 0.025119913 | | 9.56E-06 | | 0.0326817 | | | 0.032181 | | | 0.309845 | | |  |  |  |  |
| genus.Coprobacter.id.949 | | rs305411 | | A | | G | | | 0.1292185 | | | 0.026451784 | | 1.01E-06 | | -0.0400345 | | | 0.037761 | | | 0.289046 | | |  |  |  |  |
| genus.Coprobacter.id.949 | | rs3828477 | | G | | T | | | -0.091218 | | | 0.019569041 | | 2.89E-06 | | 0.0010477 | | | 0.024062 | | | 0.965271 | | |  |  |  |  |
| genus.Coprobacter.id.949 | | rs5011652 | | G | | C | | | 0.0899597 | | | 0.02000097 | | 5.51E-06 | | -0.0277343 | | | 0.025708 | | | 0.280666 | | |  |  |  |  |
| genus.Coprobacter.id.949 | | rs55672356 | | T | | A | | | -0.1933542 | | | 0.041381488 | | 2.74E-06 | | -0.0530437 | | | 0.065352 | | | 0.416986 | | |  |  |  |  |
| genus.Coprobacter.id.949 | | rs72821405 | | T | | C | | | -0.1473653 | | | 0.031997805 | | 4.76E-06 | | -0.0593049 | | | 0.040435 | | | 0.142468 | | |  |  |  |  |
| genus.Coprobacter.id.949 | | rs74919520 | | G | | A | | | 0.1257029 | | | 0.02762957 | | 5.76E-06 | | -0.0173161 | | | 0.036839 | | | 0.638319 | | |  |  |  |  |
| genus.Coprobacter.id.949 | | rs76001613 | | C | | G | | | 0.2159104 | | | 0.049301859 | | 9.13E-06 | | 0.0569525 | | | 0.045389 | | | 0.20956 | | |  |  |  |  |
| genus.Coprococcus1.id.11301 | | rs1010560 | | C | | A | | | 0.0580232 | | | 0.012272151 | | 1.96E-06 | | -0.036873 | | | 0.025789 | | | 0.152779 | | |  |  |  |  |
| genus.Coprococcus1.id.11301 | | rs12794898 | | G | | T | | | 0.0903299 | | | 0.019721215 | | 4.92E-06 | | 0.0101579 | | | 0.034299 | | | 0.767111 | | |  |  |  |  |
| genus.Coprococcus1.id.11301 | | rs12886051 | | G | | C | | | -0.0521782 | | | 0.011805063 | | 8.01E-06 | | 0.0076296 | | | 0.02507 | | | 0.760876 | | |  |  |  |  |
| genus.Coprococcus1.id.11301 | | rs1519491 | | T | | C | | | 0.0499197 | | | 0.01135578 | | 8.95E-06 | | 0.0245745 | | | 0.023333 | | | 0.292249 | | |  |  |  |  |
| genus.Coprococcus1.id.11301 | | rs1576241 | | A | | G | | | -0.0510343 | | | 0.010953456 | | 3.33E-06 | | 0.0108865 | | | 0.023555 | | | 0.643957 | | |  |  |  |  |
| genus.Coprococcus1.id.11301 | | rs1762123 | | C | | T | | | -0.0891532 | | | 0.019855409 | | 8.01E-06 | | -0.0132228 | | | 0.037818 | | | 0.726603 | | |  |  |  |  |
| genus.Coprococcus1.id.11301 | | rs2907920 | | A | | G | | | 0.0561173 | | | 0.012682384 | | 7.65E-06 | | -0.0360045 | | | 0.025401 | | | 0.156355 | | |  |  |  |  |
| genus.Coprococcus1.id.11301 | | rs4277593 | | G | | A | | | -0.0585649 | | | 0.010991363 | | 1.14E-07 | | -0.0076863 | | | 0.023274 | | | 0.741213 | | |  |  |  |  |
| genus.Coprococcus1.id.11301 | | rs56405618 | | A | | G | | | -0.08963 | | | 0.018650815 | | 1.57E-06 | | 0.08849 | | | 0.03583 | | | 0.0135204 | | |  |  |  |  |
| genus.Coprococcus1.id.11301 | | rs73031725 | | T | | C | | | 0.167591 | | | 0.035522381 | | 1.98E-06 | | -0.0512933 | | | 0.065805 | | | 0.4357 | | |  |  |  |  |
| genus.Coprococcus1.id.11301 | | rs73167075 | | T | | C | | | 0.0573137 | | | 0.01275731 | | 8.57E-06 | | -0.0279507 | | | 0.027961 | | | 0.317487 | | |  |  |  |  |
| genus.Coprococcus1.id.11301 | | rs74101919 | | T | | C | | | -0.0718989 | | | 0.014465115 | | 1.03E-06 | | 0.0227499 | | | 0.036853 | | | 0.537022 | | |  |  |  |  |
| genus.Coprococcus1.id.11301 | | rs7784490 | | C | | G | | | -0.0518543 | | | 0.011313593 | | 4.55E-06 | | -0.0262135 | | | 0.024211 | | | 0.278934 | | |  |  |  |  |
| genus.Coprococcus1.id.11301 | | rs946513 | | C | | T | | | 0.2058989 | | | 0.046001796 | | 8.62E-06 | | 0.0051843 | | | 0.055473 | | | 0.925542 | | |  |  |  |  |
| genus.Coprococcus2.id.11302 | | rs10070053 | | A | | G | | | 0.0594312 | | | 0.01353752 | | 7.65E-06 | | -0.0295821 | | | 0.023215 | | | 0.202568 | | |  |  |  |  |
| genus.Coprococcus2.id.11302 | | rs10121347 | | C | | G | | | 0.0925805 | | | 0.021945877 | | 8.31E-06 | | -0.0140414 | | | 0.035712 | | | 0.694186 | | |  |  |  |  |
| genus.Coprococcus2.id.11302 | | rs12634070 | | T | | C | | | 0.0736486 | | | 0.016492134 | | 9.95E-06 | | -0.0482528 | | | 0.02615 | | | 0.065004 | | |  |  |  |  |
| genus.Coprococcus2.id.11302 | | rs1958519 | | T | | A | | | 0.0665288 | | | 0.013855806 | | 1.58E-06 | | 0.0036483 | | | 0.022959 | | | 0.873742 | | |  |  |  |  |
| genus.Coprococcus2.id.11302 | | rs2482516 | | C | | T | | | 0.0754415 | | | 0.016461865 | | 4.72E-06 | | -0.0431974 | | | 0.027446 | | | 0.115507 | | |  |  |  |  |
| genus.Coprococcus2.id.11302 | | rs35890118 | | A | | G | | | -0.066536 | | | 0.014766106 | | 8.26E-06 | | 0.0030636 | | | 0.026302 | | | 0.907273 | | |  |  |  |  |
| genus.Coprococcus2.id.11302 | | rs59936925 | | A | | T | | | 0.1170853 | | | 0.023399473 | | 9.38E-07 | | 0.0425573 | | | 0.036606 | | | 0.245001 | | |  |  |  |  |
| genus.Coprococcus2.id.11302 | | rs61823518 | | A | | C | | | -0.0955381 | | | 0.021572431 | | 6.68E-06 | | 0.0420375 | | | 0.037117 | | | 0.257393 | | |  |  |  |  |
| genus.Coprococcus2.id.11302 | | rs6677933 | | C | | T | | | -0.0804405 | | | 0.016421626 | | 1.19E-06 | | -0.0371285 | | | 0.03237 | | | 0.251384 | | |  |  |  |  |
| genus.Coprococcus2.id.11302 | | rs6894272 | | T | | C | | | -0.1134582 | | | 0.025300319 | | 9.53E-06 | | 0.021885 | | | 0.035013 | | | 0.531939 | | |  |  |  |  |
| genus.Coprococcus2.id.11302 | | rs72680320 | | T | | C | | | -0.0649382 | | | 0.013918999 | | 2.27E-06 | | 0.0173468 | | | 0.024243 | | | 0.474275 | | |  |  |  |  |
| genus.Coprococcus2.id.11302 | | rs9426473 | | A | | G | | | 0.0727361 | | | 0.016166159 | | 6.31E-06 | | 0.0160832 | | | 0.026014 | | | 0.536415 | | |  |  |  |  |
| genus.Coprococcus3.id.11303 | | rs10810043 | | A | | G | | | 0.0515518 | | | 0.011592302 | | 9.27E-06 | | 0.0163481 | | | 0.024303 | | | 0.501148 | | |  |  |  |  |
| genus.Coprococcus3.id.11303 | | rs11077359 | | T | | C | | | -0.0645443 | | | 0.014883314 | | 9.64E-06 | | -0.0192103 | | | 0.030521 | | | 0.529077 | | |  |  |  |  |
| genus.Coprococcus3.id.11303 | | rs11080344 | | C | | T | | | 0.0516921 | | | 0.011305408 | | 4.79E-06 | | 0.0159624 | | | 0.022906 | | | 0.485884 | | |  |  |  |  |
| genus.Coprococcus3.id.11303 | | rs13247359 | | G | | A | | | 0.051211 | | | 0.011303088 | | 7.33E-06 | | -0.0321315 | | | 0.023034 | | | 0.163033 | | |  |  |  |  |
| genus.Coprococcus3.id.11303 | | rs178271 | | T | | C | | | 0.1452569 | | | 0.029423868 | | 7.81E-07 | | 0.108222 | | | 0.088618 | | | 0.222002 | | |  |  |  |  |
| genus.Coprococcus3.id.11303 | | rs34731438 | | T | | G | | | -0.0626607 | | | 0.013730465 | | 7.71E-06 | | -0.0162161 | | | 0.026424 | | | 0.539424 | | |  |  |  |  |
| genus.Coprococcus3.id.11303 | | rs4575475 | | G | | A | | | 0.0619514 | | | 0.013778722 | | 7.04E-06 | | 0.0339854 | | | 0.027271 | | | 0.212679 | | |  |  |  |  |
| genus.Coprococcus3.id.11303 | | rs62481985 | | G | | C | | | -0.0582385 | | | 0.011505221 | | 4.18E-07 | | -0.0100826 | | | 0.023206 | | | 0.663943 | | |  |  |  |  |
| genus.Coprococcus3.id.11303 | | rs6994742 | | C | | T | | | 0.0551306 | | | 0.012392938 | | 7.84E-06 | | 0.0134692 | | | 0.023442 | | | 0.56557 | | |  |  |  |  |
| genus.Coprococcus3.id.11303 | | rs7521171 | | G | | A | | | -0.0596434 | | | 0.012927857 | | 4.32E-06 | | -0.0041268 | | | 0.024792 | | | 0.867795 | | |  |  |  |  |
| genus.Coprococcus3.id.11303 | | rs8100692 | | T | | C | | | 0.0577411 | | | 0.011347595 | | 4.16E-07 | | -0.0006471 | | | 0.022955 | | | 0.97751 | | |  |  |  |  |
| genus.DefluviitaleaceaeUCG011.id.11287 | | rs112893842 | | T | | C | | | 0.1138102 | | | 0.023280562 | | 1.45E-06 | | 0.057099 | | | 0.03987 | | | 0.152105 | | |  |  |  |  |
| genus.DefluviitaleaceaeUCG011.id.11287 | | rs12122999 | | G | | T | | | -0.0827292 | | | 0.018115427 | | 4.83E-06 | | -0.0170738 | | | 0.026375 | | | 0.51741 | | |  |  |  |  |
| genus.DefluviitaleaceaeUCG011.id.11287 | | rs1582238 | | T | | C | | | 0.0805036 | | | 0.016725672 | | 1.57E-06 | | 0.0117964 | | | 0.023861 | | | 0.621041 | | |  |  |  |  |
| genus.DefluviitaleaceaeUCG011.id.11287 | | rs28696126 | | A | | T | | | -0.1065027 | | | 0.023847256 | | 6.63E-06 | | 0.0081185 | | | 0.034216 | | | 0.812447 | | |  |  |  |  |
| genus.DefluviitaleaceaeUCG011.id.11287 | | rs2892880 | | G | | A | | | 0.0817755 | | | 0.018168647 | | 6.83E-06 | | 0.0409281 | | | 0.026328 | | | 0.12006 | | |  |  |  |  |
| genus.DefluviitaleaceaeUCG011.id.11287 | | rs4344384 | | G | | T | | | 0.0715978 | | | 0.015630912 | | 4.83E-06 | | -0.0304448 | | | 0.022869 | | | 0.183107 | | |  |  |  |  |
| genus.DefluviitaleaceaeUCG011.id.11287 | | rs4677103 | | A | | G | | | 0.0977912 | | | 0.019726332 | | 9.60E-07 | | -0.0453333 | | | 0.03017 | | | 0.132947 | | |  |  |  |  |
| genus.DefluviitaleaceaeUCG011.id.11287 | | rs55658617 | | T | | C | | | 0.1743688 | | | 0.036222554 | | 2.15E-06 | | -0.012355 | | | 0.061588 | | | 0.841004 | | |  |  |  |  |
| genus.DefluviitaleaceaeUCG011.id.11287 | | rs72731813 | | C | | T | | | -0.147383 | | | 0.029379613 | | 4.33E-07 | | -0.0207884 | | | 0.053407 | | | 0.697093 | | |  |  |  |  |
| genus.DefluviitaleaceaeUCG011.id.11287 | | rs9608282 | | T | | G | | | 0.1429392 | | | 0.029979298 | | 2.52E-06 | | 0.065533 | | | 0.063702 | | | 0.303601 | | |  |  |  |  |
| genus.DefluviitaleaceaeUCG011.id.11287 | | rs9725395 | | A | | G | | | -0.1383397 | | | 0.029554002 | | 3.52E-06 | | -0.0671346 | | | 0.036082 | | | 0.0627986 | | |  |  |  |  |
| genus.Desulfovibrio.id.3173 | | rs11160353 | | T | | A | | | -0.0691756 | | | 0.014728505 | | 2.70E-06 | | 0.011492 | | | 0.023236 | | | 0.620902 | | |  |  |  |  |
| genus.Desulfovibrio.id.3173 | | rs12031543 | | T | | C | | | -0.127185 | | | 0.028188722 | | 6.55E-06 | | -0.014874 | | | 0.033223 | | | 0.654371 | | |  |  |  |  |
| genus.Desulfovibrio.id.3173 | | rs1294544 | | A | | C | | | 0.0688782 | | | 0.015363458 | | 7.78E-06 | | -0.0028748 | | | 0.025123 | | | 0.908896 | | |  |  |  |  |
| genus.Desulfovibrio.id.3173 | | rs13066142 | | G | | A | | | 0.1191406 | | | 0.025089055 | | 3.79E-06 | | 0.0201509 | | | 0.039567 | | | 0.610551 | | |  |  |  |  |
| genus.Desulfovibrio.id.3173 | | rs16863365 | | A | | G | | | 0.1094025 | | | 0.022696375 | | 1.79E-06 | | -0.033947 | | | 0.054502 | | | 0.533377 | | |  |  |  |  |
| genus.Desulfovibrio.id.3173 | | rs2032031 | | A | | G | | | -0.0654931 | | | 0.014868084 | | 9.14E-06 | | -0.020236 | | | 0.022843 | | | 0.375692 | | |  |  |  |  |
| genus.Desulfovibrio.id.3173 | | rs2590913 | | G | | A | | | 0.1544955 | | | 0.033939625 | | 6.65E-06 | | 0.0871632 | | | 0.052527 | | | 0.0970376 | | |  |  |  |  |
| genus.Desulfovibrio.id.3173 | | rs2853179 | | C | | T | | | 0.0811971 | | | 0.017423443 | | 2.42E-06 | | -0.0383682 | | | 0.027259 | | | 0.159265 | | |  |  |  |  |
| genus.Desulfovibrio.id.3173 | | rs4797774 | | G | | A | | | 0.2125819 | | | 0.04700444 | | 5.64E-06 | | 0.0492104 | | | 0.058632 | | | 0.401295 | | |  |  |  |  |
| genus.Desulfovibrio.id.3173 | | rs6580353 | | T | | C | | | 0.0770979 | | | 0.016973529 | | 4.94E-06 | | -0.0243017 | | | 0.028894 | | | 0.400318 | | |  |  |  |  |
| genus.Desulfovibrio.id.3173 | | rs72647089 | | T | | G | | | -0.1065944 | | | 0.023933668 | | 8.30E-06 | | 0.0268773 | | | 0.04202 | | | 0.522407 | | |  |  |  |  |
| genus.Desulfovibrio.id.3173 | | rs7729080 | | C | | A | | | -0.070287 | | | 0.015769108 | | 9.96E-06 | | 0.0141588 | | | 0.025066 | | | 0.572174 | | |  |  |  |  |
| genus.Dialister.id.2183 | | rs10138457 | | T | | C | | | -0.1130538 | | | 0.026193253 | | 7.88E-06 | | 0.0077961 | | | 0.039371 | | | 0.843031 | | |  |  |  |  |
| genus.Dialister.id.2183 | | rs10938938 | | G | | A | | | -0.0773514 | | | 0.017091491 | | 7.37E-06 | | 0.0495283 | | | 0.031275 | | | 0.113279 | | |  |  |  |  |
| genus.Dialister.id.2183 | | rs11071887 | | T | | C | | | 0.0662403 | | | 0.014631484 | | 5.91E-06 | | -0.0312691 | | | 0.024441 | | | 0.20077 | | |  |  |  |  |
| genus.Dialister.id.2183 | | rs11166701 | | G | | A | | | -0.0655301 | | | 0.013186 | | 5.51E-07 | | 0.0008624 | | | 0.022863 | | | 0.96991 | | |  |  |  |  |
| genus.Dialister.id.2183 | | rs2314294 | | T | | C | | | 0.0865932 | | | 0.019371895 | | 8.08E-06 | | -0.0250799 | | | 0.033381 | | | 0.452457 | | |  |  |  |  |
| genus.Dialister.id.2183 | | rs2435610 | | A | | C | | | 0.0647131 | | | 0.014331424 | | 5.93E-06 | | 0.0071358 | | | 0.02638 | | | 0.786772 | | |  |  |  |  |
| genus.Dialister.id.2183 | | rs4747450 | | C | | A | | | 0.0668509 | | | 0.014768536 | | 5.84E-06 | | 0.0044339 | | | 0.027492 | | | 0.871874 | | |  |  |  |  |
| genus.Dialister.id.2183 | | rs4753063 | | G | | A | | | -0.0596297 | | | 0.013005185 | | 4.86E-06 | | -0.0197534 | | | 0.02291 | | | 0.388575 | | |  |  |  |  |
| genus.Dialister.id.2183 | | rs517089 | | T | | A | | | 0.076227 | | | 0.017041108 | | 5.14E-06 | | 0.0434745 | | | 0.030406 | | | 0.152776 | | |  |  |  |  |
| genus.Dialister.id.2183 | | rs75416973 | | A | | G | | | 0.0727223 | | | 0.016450983 | | 9.46E-06 | | 0.0376908 | | | 0.027678 | | | 0.173277 | | |  |  |  |  |
| genus.Dialister.id.2183 | | rs764177 | | C | | A | | | -0.0601454 | | | 0.013536587 | | 9.61E-06 | | 0.0212746 | | | 0.023708 | | | 0.36953 | | |  |  |  |  |
| genus.Dialister.id.2183 | | rs76680460 | | G | | A | | | -0.1612961 | | | 0.036433211 | | 8.19E-06 | | 0.111742 | | | 0.056792 | | | 0.0491168 | | |  |  |  |  |
| genus.Dorea.id.1997 | | rs11150408 | | T | | G | | | 0.0488254 | | | 0.010906337 | | 7.06E-06 | | 0.0057471 | | | 0.022941 | | | 0.802185 | | |  |  |  |  |
| genus.Dorea.id.1997 | | rs12216169 | | T | | A | | | 0.0881722 | | | 0.019368315 | | 5.33E-06 | | 0.0443897 | | | 0.035604 | | | 0.21248 | | |  |  |  |  |
| genus.Dorea.id.1997 | | rs12537781 | | T | | C | | | -0.055544 | | | 0.012522298 | | 9.15E-06 | | -0.048717 | | | 0.026741 | | | 0.0684873 | | |  |  |  |  |
| genus.Dorea.id.1997 | | rs13279148 | | G | | A | | | 0.0715309 | | | 0.015087753 | | 2.25E-06 | | 0.001224 | | | 0.036544 | | | 0.973282 | | |  |  |  |  |
| genus.Dorea.id.1997 | | rs1899291 | | C | | T | | | 0.0697217 | | | 0.015028462 | | 4.57E-06 | | 0.0077491 | | | 0.031533 | | | 0.805878 | | |  |  |  |  |
| genus.Dorea.id.1997 | | rs3005511 | | A | | G | | | 0.0516127 | | | 0.011285226 | | 5.29E-06 | | -0.0473362 | | | 0.024902 | | | 0.0573113 | | |  |  |  |  |
| genus.Dorea.id.1997 | | rs345219 | | T | | G | | | -0.0497368 | | | 0.011262081 | | 8.80E-06 | | 0.0096048 | | | 0.023057 | | | 0.67699 | | |  |  |  |  |
| genus.Dorea.id.1997 | | rs3752849 | | G | | A | | | 0.1638459 | | | 0.036607992 | | 7.68E-06 | | 0.0021508 | | | 0.052324 | | | 0.967212 | | |  |  |  |  |
| genus.Dorea.id.1997 | | rs4793307 | | C | | T | | | 0.0574165 | | | 0.012247163 | | 4.01E-06 | | 0.0036018 | | | 0.026952 | | | 0.893691 | | |  |  |  |  |
| genus.Dorea.id.1997 | | rs62503162 | | A | | G | | | -0.0974091 | | | 0.019437924 | | 7.47E-07 | | -0.0454646 | | | 0.05643 | | | 0.420425 | | |  |  |  |  |
| genus.Dorea.id.1997 | | rs62583469 | | T | | A | | | -0.0634136 | | | 0.014222695 | | 5.78E-06 | | 0.0519673 | | | 0.033278 | | | 0.118374 | | |  |  |  |  |
| genus.Dorea.id.1997 | | rs73729431 | | C | | T | | | -0.1374503 | | | 0.029998324 | | 3.17E-06 | | -0.0144753 | | | 0.080972 | | | 0.858119 | | |  |  |  |  |
| genus.Dorea.id.1997 | | rs78772518 | | A | | C | | | 0.1382597 | | | 0.028360928 | | 3.38E-06 | | 0.0432378 | | | 0.034611 | | | 0.211567 | | |  |  |  |  |
| genus.Eggerthella.id.819 | | rs112205261 | | T | | C | | | -0.1886179 | | | 0.040361319 | | 3.35E-06 | | 0.0533236 | | | 0.04199 | | | 0.204116 | | |  |  |  |  |
| genus.Eggerthella.id.819 | | rs13070736 | | A | | C | | | -0.1213311 | | | 0.027202382 | | 7.62E-06 | | -0.0079753 | | | 0.030856 | | | 0.796044 | | |  |  |  |  |
| genus.Eggerthella.id.819 | | rs1784446 | | G | | A | | | 0.0907728 | | | 0.019809579 | | 5.23E-06 | | 0.0309772 | | | 0.022751 | | | 0.173323 | | |  |  |  |  |
| genus.Eggerthella.id.819 | | rs2223081 | | G | | A | | | 0.1025717 | | | 0.022110762 | | 3.89E-06 | | 0.0434181 | | | 0.025395 | | | 0.0873213 | | |  |  |  |  |
| genus.Eggerthella.id.819 | | rs2240838 | | A | | G | | | 0.0980582 | | | 0.01976528 | | 7.36E-07 | | -0.007521 | | | 0.023086 | | | 0.744591 | | |  |  |  |  |
| genus.Eggerthella.id.819 | | rs3851328 | | T | | G | | | -0.1077743 | | | 0.023652669 | | 4.18E-06 | | -0.0273932 | | | 0.027546 | | | 0.32 | | |  |  |  |  |
| genus.Eggerthella.id.819 | | rs6430926 | | C | | T | | | 0.0879736 | | | 0.019712748 | | 8.37E-06 | | 0.0013137 | | | 0.023017 | | | 0.954486 | | |  |  |  |  |
| genus.Eggerthella.id.819 | | rs67490567 | | T | | C | | | 0.1084948 | | | 0.024545741 | | 8.94E-06 | | 0.018551 | | | 0.026397 | | | 0.482199 | | |  |  |  |  |
| genus.Eggerthella.id.819 | | rs76663501 | | C | | T | | | 0.1753494 | | | 0.03785764 | | 4.83E-06 | | -0.0350785 | | | 0.051736 | | | 0.497754 | | |  |  |  |  |
| genus.Eisenbergiella.id.11304 | | rs11027642 | | C | | T | | | 0.1290055 | | | 0.028484392 | | 4.92E-06 | | 3.43E-05 | | | 0.032602 | | | 0.99916 | | |  |  |  |  |
| genus.Eisenbergiella.id.11304 | | rs11079158 | | T | | C | | | 0.1006268 | | | 0.022546032 | | 7.35E-06 | | -0.0444674 | | | 0.027673 | | | 0.108073 | | |  |  |  |  |
| genus.Eisenbergiella.id.11304 | | rs11938607 | | T | | C | | | 0.0978125 | | | 0.021663562 | | 8.22E-06 | | 0.0370737 | | | 0.026172 | | | 0.156609 | | |  |  |  |  |
| genus.Eisenbergiella.id.11304 | | rs12257723 | | A | | C | | | -0.0952742 | | | 0.021156892 | | 8.85E-06 | | -0.0061453 | | | 0.024332 | | | 0.800606 | | |  |  |  |  |
| genus.Eisenbergiella.id.11304 | | rs12278566 | | T | | A | | | -0.1210647 | | | 0.025165804 | | 1.65E-06 | | -0.0087744 | | | 0.025876 | | | 0.734541 | | |  |  |  |  |
| genus.Eisenbergiella.id.11304 | | rs12710729 | | C | | A | | | 0.0893384 | | | 0.019906928 | | 9.84E-06 | | 0.0276889 | | | 0.024441 | | | 0.257267 | | |  |  |  |  |
| genus.Eisenbergiella.id.11304 | | rs13258851 | | A | | G | | | 0.1370014 | | | 0.030212434 | | 7.75E-06 | | 0.0549788 | | | 0.032774 | | | 0.0934415 | | |  |  |  |  |
| genus.Eisenbergiella.id.11304 | | rs1508033 | | A | | C | | | 0.091546 | | | 0.019578652 | | 3.23E-06 | | 0.0459758 | | | 0.02481 | | | 0.0638631 | | |  |  |  |  |
| genus.Eisenbergiella.id.11304 | | rs1553971 | | T | | G | | | 0.1209645 | | | 0.026305423 | | 5.27E-06 | | -0.0101654 | | | 0.027395 | | | 0.710591 | | |  |  |  |  |
| genus.Eisenbergiella.id.11304 | | rs2683098 | | C | | T | | | 0.1073189 | | | 0.022515029 | | 2.24E-06 | | 0.0073394 | | | 0.027782 | | | 0.79164 | | |  |  |  |  |
| genus.Eisenbergiella.id.11304 | | rs3812426 | | G | | A | | | 0.1064473 | | | 0.0224159 | | 2.72E-06 | | 0.0379008 | | | 0.031491 | | | 0.228759 | | |  |  |  |  |
| genus.Eisenbergiella.id.11304 | | rs4462860 | | G | | A | | | 0.0939071 | | | 0.020110429 | | 4.16E-06 | | 0.0408857 | | | 0.023453 | | | 0.0812831 | | |  |  |  |  |
| genus.Enterorhabdus.id.820 | | rs10098492 | | T | | C | | | 0.1323231 | | | 0.029368842 | | 6.41E-06 | | -0.026744 | | | 0.048858 | | | 0.584119 | | |  |  |  |  |
| genus.Enterorhabdus.id.820 | | rs11098863 | | T | | A | | | -0.0966008 | | | 0.016267701 | | 3.06E-09 | | -0.0268394 | | | 0.022838 | | | 0.239913 | | |  |  |  |  |
| genus.Enterorhabdus.id.820 | | rs114731706 | | T | | G | | | 0.182308 | | | 0.038229783 | | 2.17E-06 | | -0.0591041 | | | 0.065401 | | | 0.366147 | | |  |  |  |  |
| genus.Enterorhabdus.id.820 | | rs2051957 | | C | | T | | | 0.0843291 | | | 0.018986405 | | 8.90E-06 | | -0.0702998 | | | 0.028668 | | | 0.0141987 | | |  |  |  |  |
| genus.Enterorhabdus.id.820 | | rs3017103 | | A | | G | | | 0.0980897 | | | 0.020899456 | | 2.94E-06 | | -0.0233676 | | | 0.02917 | | | 0.423077 | | |  |  |  |  |
| genus.Enterorhabdus.id.820 | | rs73331712 | | T | | C | | | 0.2619895 | | | 0.055123019 | | 4.85E-06 | | 0.0417039 | | | 0.05689 | | | 0.463524 | | |  |  |  |  |
| genus.Enterorhabdus.id.820 | | rs77655283 | | G | | A | | | 0.1329876 | | | 0.029830551 | | 5.88E-06 | | 0.0008107 | | | 0.044715 | | | 0.985534 | | |  |  |  |  |
| genus.Enterorhabdus.id.820 | | rs7923280 | | A | | T | | | 0.0860019 | | | 0.016914834 | | 5.24E-07 | | 0.041454 | | | 0.025467 | | | 0.103572 | | |  |  |  |  |
| genus.Enterorhabdus.id.820 | | rs9470637 | | A | | T | | | -0.0755513 | | | 0.016626296 | | 5.70E-06 | | -0.0163609 | | | 0.02291 | | | 0.475137 | | |  |  |  |  |
| genus.Erysipelatoclostridium.id.11381 | | rs1434153 | | G | | A | | | -0.0684526 | | | 0.01524432 | | 6.85E-06 | | -0.0054721 | | | 0.023008 | | | 0.812009 | | |  |  |  |  |
| genus.Erysipelatoclostridium.id.11381 | | rs16936671 | | C | | T | | | -0.0968026 | | | 0.021788273 | | 6.04E-06 | | 0.0169763 | | | 0.033132 | | | 0.608387 | | |  |  |  |  |
| genus.Erysipelatoclostridium.id.11381 | | rs17804233 | | T | | C | | | -0.0662914 | | | 0.014420319 | | 4.59E-06 | | -0.0278021 | | | 0.022864 | | | 0.223989 | | |  |  |  |  |
| genus.Erysipelatoclostridium.id.11381 | | rs2901723 | | C | | A | | | 0.0641339 | | | 0.014429456 | | 8.79E-06 | | -0.0231479 | | | 0.023079 | | | 0.315862 | | |  |  |  |  |
| genus.Erysipelatoclostridium.id.11381 | | rs340991 | | A | | G | | | -0.0740482 | | | 0.015889138 | | 3.75E-06 | | 0.0442491 | | | 0.025814 | | | 0.0865048 | | |  |  |  |  |
| genus.Erysipelatoclostridium.id.11381 | | rs34528142 | | C | | G | | | -0.0875551 | | | 0.019555627 | | 6.13E-06 | | -0.01425 | | | 0.028455 | | | 0.616516 | | |  |  |  |  |
| genus.Erysipelatoclostridium.id.11381 | | rs3804326 | | A | | G | | | 0.1414698 | | | 0.033635154 | | 9.85E-06 | | -0.0329821 | | | 0.052963 | | | 0.533455 | | |  |  |  |  |
| genus.Erysipelatoclostridium.id.11381 | | rs45480394 | | T | | G | | | -0.0687134 | | | 0.015183535 | | 7.66E-06 | | 0.0264553 | | | 0.023805 | | | 0.266415 | | |  |  |  |  |
| genus.Erysipelatoclostridium.id.11381 | | rs4697572 | | A | | G | | | -0.0810636 | | | 0.016330822 | | 7.59E-07 | | -0.0020323 | | | 0.028637 | | | 0.943423 | | |  |  |  |  |
| genus.Erysipelatoclostridium.id.11381 | | rs58236560 | | G | | T | | | -0.1111569 | | | 0.023442572 | | 2.16E-06 | | -0.037557 | | | 0.035064 | | | 0.284118 | | |  |  |  |  |
| genus.Erysipelatoclostridium.id.11381 | | rs61806970 | | C | | T | | | 0.142548 | | | 0.032068815 | | 9.09E-06 | | 0.0392093 | | | 0.045613 | | | 0.390004 | | |  |  |  |  |
| genus.Erysipelatoclostridium.id.11381 | | rs62060367 | | T | | C | | | -0.0798013 | | | 0.018135792 | | 6.70E-06 | | -0.0253779 | | | 0.032215 | | | 0.430836 | | |  |  |  |  |
| genus.Erysipelatoclostridium.id.11381 | | rs622418 | | A | | G | | | -0.0668051 | | | 0.014322449 | | 3.68E-06 | | -0.0125196 | | | 0.022818 | | | 0.583236 | | |  |  |  |  |
| genus.Erysipelatoclostridium.id.11381 | | rs6474512 | | A | | C | | | 0.067015 | | | 0.014320143 | | 3.02E-06 | | -0.0486276 | | | 0.023529 | | | 0.0387588 | | |  |  |  |  |
| genus.Erysipelatoclostridium.id.11381 | | rs710230 | | T | | C | | | 0.1433813 | | | 0.028151388 | | 6.33E-07 | | 0.112392 | | | 0.042808 | | | 0.00865207 | | |  |  |  |  |
| genus.Erysipelatoclostridium.id.11381 | | rs7221249 | | A | | G | | | 0.0839861 | | | 0.01427414 | | 4.31E-09 | | 0.0005782 | | | 0.022893 | | | 0.979849 | | |  |  |  |  |
| genus.Erysipelatoclostridium.id.11381 | | rs9590927 | | G | | A | | | -0.0646003 | | | 0.014347949 | | 6.39E-06 | | -0.0271777 | | | 0.022942 | | | 0.236174 | | |  |  |  |  |
| genus.ErysipelotrichaceaeUCG003.id.11384 | | rs10164067 | | T | | G | | | -0.1032601 | | | 0.021240502 | | 1.13E-06 | | -0.0782969 | | | 0.051454 | | | 0.12809 | | |  |  |  |  |
| genus.ErysipelotrichaceaeUCG003.id.11384 | | rs11666127 | | A | | G | | | -0.0719187 | | | 0.016137058 | | 7.90E-06 | | 0.0019573 | | | 0.030121 | | | 0.948188 | | |  |  |  |  |
| genus.ErysipelotrichaceaeUCG003.id.11384 | | rs11994308 | | C | | T | | | 0.1154174 | | | 0.024250252 | | 1.33E-06 | | -0.0072839 | | | 0.040048 | | | 0.855678 | | |  |  |  |  |
| genus.ErysipelotrichaceaeUCG003.id.11384 | | rs12251396 | | A | | G | | | -0.0705399 | | | 0.015876598 | | 9.52E-06 | | 0.0113951 | | | 0.031554 | | | 0.718001 | | |  |  |  |  |
| genus.ErysipelotrichaceaeUCG003.id.11384 | | rs17798136 | | G | | A | | | 0.1588176 | | | 0.034767431 | | 3.24E-06 | | 0.0456027 | | | 0.046639 | | | 0.328178 | | |  |  |  |  |
| genus.ErysipelotrichaceaeUCG003.id.11384 | | rs214586 | | T | | C | | | -0.1064546 | | | 0.024038036 | | 8.50E-06 | | 0.0093246 | | | 0.036818 | | | 0.800065 | | |  |  |  |  |
| genus.ErysipelotrichaceaeUCG003.id.11384 | | rs2302840 | | T | | C | | | -0.0871966 | | | 0.017687036 | | 1.29E-06 | | -0.0274123 | | | 0.030313 | | | 0.365836 | | |  |  |  |  |
| genus.ErysipelotrichaceaeUCG003.id.11384 | | rs28568391 | | A | | G | | | -0.0583911 | | | 0.011874052 | | 6.42E-07 | | -0.0180935 | | | 0.022935 | | | 0.430168 | | |  |  |  |  |
| genus.ErysipelotrichaceaeUCG003.id.11384 | | rs4758231 | | G | | T | | | -0.0551754 | | | 0.012204675 | | 6.55E-06 | | -0.0039677 | | | 0.0254 | | | 0.875867 | | |  |  |  |  |
| genus.ErysipelotrichaceaeUCG003.id.11384 | | rs59068084 | | T | | G | | | 0.0564772 | | | 0.012022387 | | 3.12E-06 | | 0.0031645 | | | 0.023535 | | | 0.893041 | | |  |  |  |  |
| genus.ErysipelotrichaceaeUCG003.id.11384 | | rs62403464 | | T | | C | | | -0.0731743 | | | 0.015651572 | | 3.44E-06 | | -0.002087 | | | 0.029694 | | | 0.943967 | | |  |  |  |  |
| genus.ErysipelotrichaceaeUCG003.id.11384 | | rs6875357 | | C | | T | | | 0.1656734 | | | 0.035383116 | | 6.70E-06 | | 0.0110469 | | | 0.059591 | | | 0.852931 | | |  |  |  |  |
| genus.ErysipelotrichaceaeUCG003.id.11384 | | rs73074432 | | C | | T | | | 0.072154 | | | 0.016437785 | | 9.99E-06 | | 0.0431383 | | | 0.036802 | | | 0.241132 | | |  |  |  |  |
| genus.ErysipelotrichaceaeUCG003.id.11384 | | rs74988980 | | G | | A | | | -0.1331414 | | | 0.034857439 | | 8.64E-06 | | -0.0756535 | | | 0.056302 | | | 0.179043 | | |  |  |  |  |
| genus.ErysipelotrichaceaeUCG003.id.11384 | | rs75949021 | | T | | C | | | -0.1696671 | | | 0.037423793 | | 3.58E-06 | | 0.0717065 | | | 0.057698 | | | 0.213944 | | |  |  |  |  |
| genus.ErysipelotrichaceaeUCG003.id.11384 | | rs76502207 | | T | | C | | | 0.1448438 | | | 0.028992913 | | 6.41E-07 | | -0.0484565 | | | 0.05792 | | | 0.40281 | | |  |  |  |  |
| genus.ErysipelotrichaceaeUCG003.id.11384 | | rs79396538 | | C | | G | | | 0.0848148 | | | 0.019114638 | | 8.63E-06 | | -0.0410601 | | | 0.0496 | | | 0.407765 | | |  |  |  |  |
| genus.ErysipelotrichaceaeUCG003.id.11384 | | rs8053479 | | A | | G | | | -0.0838212 | | | 0.018650912 | | 5.83E-06 | | 0.0090561 | | | 0.03579 | | | 0.800244 | | |  |  |  |  |
| genus.Escherichia.Shigella.id.3504 | | rs112767262 | | T | | C | | | 0.0732963 | | | 0.01635886 | | 8.21E-06 | | 0.0023843 | | | 0.027554 | | | 0.931045 | | |  |  |  |  |
| genus.Escherichia.Shigella.id.3504 | | rs113127095 | | A | | G | | | 0.1510094 | | | 0.032343436 | | 3.33E-06 | | 0.0081987 | | | 0.057813 | | | 0.887227 | | |  |  |  |  |
| genus.Escherichia.Shigella.id.3504 | | rs113513883 | | A | | G | | | 0.1722833 | | | 0.038041024 | | 5.28E-06 | | 0.107542 | | | 0.064955 | | | 0.0977958 | | |  |  |  |  |
| genus.Escherichia.Shigella.id.3504 | | rs1154904 | | A | | G | | | -0.0613376 | | | 0.01306443 | | 3.04E-06 | | 0.054437 | | | 0.022794 | | | 0.0169313 | | |  |  |  |  |
| genus.Escherichia.Shigella.id.3504 | | rs11706043 | | T | | A | | | 0.075657 | | | 0.016415509 | | 5.87E-06 | | -0.0288595 | | | 0.027693 | | | 0.29736 | | |  |  |  |  |
| genus.Escherichia.Shigella.id.3504 | | rs117092367 | | A | | T | | | 0.1173618 | | | 0.026453289 | | 9.65E-06 | | 0.0643468 | | | 0.04611 | | | 0.162866 | | |  |  |  |  |
| genus.Escherichia.Shigella.id.3504 | | rs118526 | | C | | A | | | -0.0594401 | | | 0.013598005 | | 8.00E-06 | | 0.0251474 | | | 0.024986 | | | 0.314186 | | |  |  |  |  |
| genus.Escherichia.Shigella.id.3504 | | rs2267739 | | G | | C | | | 0.1155054 | | | 0.02376277 | | 1.42E-06 | | -0.061746 | | | 0.035945 | | | 0.0858322 | | |  |  |  |  |
| genus.Escherichia.Shigella.id.3504 | | rs2798105 | | A | | G | | | -0.1008507 | | | 0.02220448 | | 8.25E-06 | | -0.036078 | | | 0.03851 | | | 0.34884 | | |  |  |  |  |
| genus.Escherichia.Shigella.id.3504 | | rs35555519 | | C | | G | | | 0.101976 | | | 0.022288306 | | 4.92E-06 | | -0.0089829 | | | 0.038233 | | | 0.814244 | | |  |  |  |  |
| genus.Escherichia.Shigella.id.3504 | | rs4731451 | | G | | A | | | -0.0609885 | | | 0.013515708 | | 7.47E-06 | | 0.021436 | | | 0.02453 | | | 0.382184 | | |  |  |  |  |
| genus.Escherichia.Shigella.id.3504 | | rs57024273 | | T | | C | | | 0.0626172 | | | 0.013991866 | | 9.70E-06 | | -0.0300299 | | | 0.02622 | | | 0.252089 | | |  |  |  |  |
| genus.Escherichia.Shigella.id.3504 | | rs592299 | | T | | C | | | -0.0592004 | | | 0.012948643 | | 4.77E-06 | | -0.0223513 | | | 0.022978 | | | 0.330688 | | |  |  |  |  |
| genus.Escherichia.Shigella.id.3504 | | rs73208162 | | A | | G | | | -0.1192956 | | | 0.024838994 | | 2.19E-06 | | -0.148052 | | | 0.064619 | | | 0.0219543 | | |  |  |  |  |
| genus.Escherichia.Shigella.id.3504 | | rs7502686 | | G | | C | | | -0.1363837 | | | 0.030257643 | | 5.90E-06 | | -0.0827942 | | | 0.047386 | | | 0.0805954 | | |  |  |  |  |
| genus.Faecalibacterium.id.2057 | | rs10927394 | | G | | T | | | -0.2322621 | | | 0.051236432 | | 7.02E-06 | | -0.0200841 | | | 0.084555 | | | 0.812248 | | |  |  |  |  |
| genus.Faecalibacterium.id.2057 | | rs114946999 | | C | | T | | | -0.0861565 | | | 0.018960043 | | 5.70E-06 | | -0.0772869 | | | 0.03443 | | | 0.0247822 | | |  |  |  |  |
| genus.Faecalibacterium.id.2057 | | rs11776390 | | T | | C | | | -0.078354 | | | 0.017183046 | | 6.40E-06 | | 0.0210742 | | | 0.045898 | | | 0.646121 | | |  |  |  |  |
| genus.Faecalibacterium.id.2057 | | rs12320842 | | C | | G | | | 0.094833 | | | 0.016396224 | | 7.57E-09 | | -0.0101627 | | | 0.033536 | | | 0.761863 | | |  |  |  |  |
| genus.Faecalibacterium.id.2057 | | rs1271565 | | C | | T | | | -0.0576251 | | | 0.011964928 | | 1.30E-06 | | 0.0391294 | | | 0.026 | | | 0.132333 | | |  |  |  |  |
| genus.Faecalibacterium.id.2057 | | rs12753492 | | A | | C | | | 0.0641305 | | | 0.014993333 | | 8.80E-06 | | -0.0253355 | | | 0.036257 | | | 0.48469 | | |  |  |  |  |
| genus.Faecalibacterium.id.2057 | | rs2835874 | | T | | C | | | -0.0866356 | | | 0.019649581 | | 7.54E-06 | | 0.0077603 | | | 0.061929 | | | 0.900279 | | |  |  |  |  |
| genus.Faecalibacterium.id.2057 | | rs28376661 | | C | | G | | | 0.0504817 | | | 0.010936797 | | 3.66E-06 | | 0.0124064 | | | 0.024883 | | | 0.618068 | | |  |  |  |  |
| genus.Faecalibacterium.id.2057 | | rs61875484 | | C | | G | | | 0.0817953 | | | 0.018369023 | | 9.18E-06 | | -0.0899583 | | | 0.039164 | | | 0.0216197 | | |  |  |  |  |
| genus.Faecalibacterium.id.2057 | | rs6910935 | | A | | G | | | 0.1348639 | | | 0.027703163 | | 1.38E-06 | | 0.0554793 | | | 0.047771 | | | 0.245495 | | |  |  |  |  |
| genus.Faecalibacterium.id.2057 | | rs75499067 | | C | | T | | | 0.2275847 | | | 0.046552213 | | 1.76E-06 | | -0.0497483 | | | 0.043787 | | | 0.255893 | | |  |  |  |  |
| genus.Faecalibacterium.id.2057 | | rs79656633 | | T | | C | | | 0.1456307 | | | 0.032301873 | | 8.14E-06 | | 0.0710098 | | | 0.03821 | | | 0.0631132 | | |  |  |  |  |
| genus.Faecalibacterium.id.2057 | | rs9536330 | | T | | C | | | -0.048317 | | | 0.01079702 | | 5.33E-06 | | 0.0162331 | | | 0.022951 | | | 0.47939 | | |  |  |  |  |
| genus.FamilyXIIIAD3011group.id.11293 | | rs11126423 | | C | | T | | | 0.0904399 | | | 0.019630134 | | 5.91E-06 | | -0.0168718 | | | 0.040106 | | | 0.673985 | | |  |  |  |  |
| genus.FamilyXIIIAD3011group.id.11293 | | rs11736617 | | G | | A | | | -0.0759493 | | | 0.017210081 | | 9.02E-06 | | 0.0563799 | | | 0.050445 | | | 0.263713 | | |  |  |  |  |
| genus.FamilyXIIIAD3011group.id.11293 | | rs12812672 | | T | | C | | | -0.096065 | | | 0.020828023 | | 2.56E-06 | | -0.0667914 | | | 0.044054 | | | 0.129491 | | |  |  |  |  |
| genus.FamilyXIIIAD3011group.id.11293 | | rs12911842 | | A | | T | | | -0.0811976 | | | 0.018333544 | | 6.91E-06 | | 0.0280936 | | | 0.041011 | | | 0.493327 | | |  |  |  |  |
| genus.FamilyXIIIAD3011group.id.11293 | | rs149302 | | T | | C | | | -0.0645635 | | | 0.014322495 | | 7.48E-06 | | -0.0248551 | | | 0.027051 | | | 0.358196 | | |  |  |  |  |
| genus.FamilyXIIIAD3011group.id.11293 | | rs16840310 | | A | | G | | | -0.0608052 | | | 0.012214901 | | 6.75E-07 | | -0.0054441 | | | 0.023181 | | | 0.814324 | | |  |  |  |  |
| genus.FamilyXIIIAD3011group.id.11293 | | rs16940167 | | C | | T | | | 0.0732552 | | | 0.015989797 | | 3.91E-06 | | -0.0218067 | | | 0.029101 | | | 0.453643 | | |  |  |  |  |
| genus.FamilyXIIIAD3011group.id.11293 | | rs17156849 | | G | | A | | | -0.1128909 | | | 0.024529236 | | 4.19E-06 | | -0.0193813 | | | 0.048405 | | | 0.688862 | | |  |  |  |  |
| genus.FamilyXIIIAD3011group.id.11293 | | rs62029761 | | A | | G | | | 0.1287527 | | | 0.027600154 | | 3.89E-06 | | 0.0467799 | | | 0.050301 | | | 0.352369 | | |  |  |  |  |
| genus.FamilyXIIIAD3011group.id.11293 | | rs62200412 | | C | | T | | | -0.0800851 | | | 0.016383437 | | 5.80E-07 | | 0.0005124 | | | 0.026242 | | | 0.984421 | | |  |  |  |  |
| genus.FamilyXIIIAD3011group.id.11293 | | rs72730932 | | C | | A | | | -0.0899559 | | | 0.017710652 | | 6.89E-07 | | 0.0349397 | | | 0.03936 | | | 0.374705 | | |  |  |  |  |
| genus.FamilyXIIIAD3011group.id.11293 | | rs739451 | | C | | T | | | 0.0649589 | | | 0.014753414 | | 7.88E-06 | | -0.0673924 | | | 0.028147 | | | 0.0166502 | | |  |  |  |  |
| genus.FamilyXIIIAD3011group.id.11293 | | rs9276029 | | A | | G | | | -0.0811384 | | | 0.018566861 | | 8.93E-06 | | -0.0390714 | | | 0.028758 | | | 0.17426 | | |  |  |  |  |
| genus.FamilyXIIIAD3011group.id.11293 | | rs9837139 | | A | | G | | | 0.1075202 | | | 0.024048177 | | 8.71E-06 | | -0.0553324 | | | 0.040987 | | | 0.17702 | | |  |  |  |  |
| genus.FamilyXIIIAD3011group.id.11293 | | rs9852893 | | C | | G | | | 0.0657834 | | | 0.012905622 | | 3.88E-07 | | -0.0319872 | | | 0.025686 | | | 0.21302 | | |  |  |  |  |
| genus.FamilyXIIIUCG001.id.11294 | | rs112362903 | | A | | G | | | -0.149047 | | | 0.033327141 | | 7.88E-06 | | -0.0930363 | | | 0.064804 | | | 0.151097 | | |  |  |  |  |
| genus.FamilyXIIIUCG001.id.11294 | | rs116979587 | | T | | A | | | -0.1216789 | | | 0.02608207 | | 3.05E-06 | | -0.009303 | | | 0.053616 | | | 0.862247 | | |  |  |  |  |
| genus.FamilyXIIIUCG001.id.11294 | | rs12049454 | | T | | C | | | -0.0647298 | | | 0.013406537 | | 1.17E-06 | | 0.0039931 | | | 0.023405 | | | 0.864531 | | |  |  |  |  |
| genus.FamilyXIIIUCG001.id.11294 | | rs1426266 | | T | | C | | | -0.0665521 | | | 0.013715073 | | 1.25E-06 | | 0.0201153 | | | 0.025733 | | | 0.434393 | | |  |  |  |  |
| genus.FamilyXIIIUCG001.id.11294 | | rs3842897 | | G | | A | | | -0.1126393 | | | 0.024275096 | | 5.20E-06 | | 0.0433567 | | | 0.040271 | | | 0.281652 | | |  |  |  |  |
| genus.FamilyXIIIUCG001.id.11294 | | rs62414802 | | C | | T | | | -0.0611904 | | | 0.013456987 | | 4.29E-06 | | 0.0094327 | | | 0.02631 | | | 0.719948 | | |  |  |  |  |
| genus.FamilyXIIIUCG001.id.11294 | | rs7119679 | | G | | A | | | -0.0809085 | | | 0.017478204 | | 3.52E-06 | | 0.0077079 | | | 0.026883 | | | 0.774323 | | |  |  |  |  |
| genus.FamilyXIIIUCG001.id.11294 | | rs76463770 | | A | | G | | | 0.1931297 | | | 0.04198659 | | 3.77E-06 | | -0.0072593 | | | 0.06641 | | | 0.912956 | | |  |  |  |  |
| genus.FamilyXIIIUCG001.id.11294 | | rs8076666 | | A | | G | | | 0.088655 | | | 0.019809411 | | 8.02E-06 | | 0.0471126 | | | 0.035026 | | | 0.178604 | | |  |  |  |  |
| genus.Flavonifractor.id.2059 | | rs114873521 | | C | | T | | | -0.1300666 | | | 0.029408684 | | 7.13E-06 | | 0.0068488 | | | 0.044564 | | | 0.877857 | | |  |  |  |  |
| genus.Flavonifractor.id.2059 | | rs11642826 | | G | | C | | | 0.1468605 | | | 0.032519094 | | 6.65E-06 | | -0.0696278 | | | 0.039514 | | | 0.0780531 | | |  |  |  |  |
| genus.Flavonifractor.id.2059 | | rs11811696 | | T | | C | | | -0.1160627 | | | 0.024101172 | | 2.07E-06 | | 0.0552506 | | | 0.041678 | | | 0.184953 | | |  |  |  |  |
| genus.Flavonifractor.id.2059 | | rs12030302 | | A | | G | | | -0.0692276 | | | 0.013746695 | | 5.61E-07 | | -0.0275767 | | | 0.022865 | | | 0.227796 | | |  |  |  |  |
| genus.Flavonifractor.id.2059 | | rs12038887 | | C | | G | | | 0.0942719 | | | 0.021164606 | | 9.37E-06 | | 0.0300764 | | | 0.033647 | | | 0.371385 | | |  |  |  |  |
| genus.Flavonifractor.id.2059 | | rs34066017 | | A | | G | | | 0.0764294 | | | 0.015978927 | | 1.52E-06 | | 0.0001194 | | | 0.028325 | | | 0.996636 | | |  |  |  |  |
| genus.Flavonifractor.id.2059 | | rs4949766 | | T | | C | | | -0.0687466 | | | 0.015166112 | | 4.83E-06 | | 0.0218366 | | | 0.027645 | | | 0.429584 | | |  |  |  |  |
| genus.Flavonifractor.id.2059 | | rs6761463 | | G | | C | | | -0.083392 | | | 0.018459615 | | 8.11E-06 | | 0.0082905 | | | 0.033234 | | | 0.803007 | | |  |  |  |  |
| genus.Flavonifractor.id.2059 | | rs798674 | | G | | C | | | 0.0637125 | | | 0.014160814 | | 7.02E-06 | | 0.0143889 | | | 0.023453 | | | 0.539538 | | |  |  |  |  |
| genus.Flavonifractor.id.2059 | | rs806808 | | T | | C | | | 0.0667272 | | | 0.013655748 | | 1.18E-06 | | -0.0103165 | | | 0.023086 | | | 0.654959 | | |  |  |  |  |
| genus.Fusicatenibacter.id.11305 | | rs10439674 | | A | | G | | | -0.0572129 | | | 0.013000675 | | 7.68E-06 | | 0.0323844 | | | 0.028372 | | | 0.253692 | | |  |  |  |  |
| genus.Fusicatenibacter.id.11305 | | rs167879 | | C | | T | | | -0.0659543 | | | 0.014876261 | | 5.87E-06 | | -0.0506637 | | | 0.032143 | | | 0.114981 | | |  |  |  |  |
| genus.Fusicatenibacter.id.11305 | | rs1864685 | | A | | C | | | -0.0494799 | | | 0.010810494 | | 4.96E-06 | | -0.0011196 | | | 0.023251 | | | 0.961595 | | |  |  |  |  |
| genus.Fusicatenibacter.id.11305 | | rs2025938 | | G | | A | | | -0.0967298 | | | 0.020542557 | | 2.99E-06 | | 0.0100452 | | | 0.046376 | | | 0.828516 | | |  |  |  |  |
| genus.Fusicatenibacter.id.11305 | | rs2039204 | | T | | A | | | -0.0497415 | | | 0.010794137 | | 3.94E-06 | | 0.0125346 | | | 0.023825 | | | 0.598804 | | |  |  |  |  |
| genus.Fusicatenibacter.id.11305 | | rs206581 | | A | | G | | | -0.0568339 | | | 0.012789397 | | 8.96E-06 | | -0.0044869 | | | 0.027722 | | | 0.871422 | | |  |  |  |  |
| genus.Fusicatenibacter.id.11305 | | rs2132128 | | G | | A | | | -0.0771914 | | | 0.016034448 | | 1.08E-06 | | 0.0070103 | | | 0.037958 | | | 0.853475 | | |  |  |  |  |
| genus.Fusicatenibacter.id.11305 | | rs3303 | | T | | C | | | -0.0953657 | | | 0.020406992 | | 3.94E-06 | | -0.0748912 | | | 0.046822 | | | 0.109711 | | |  |  |  |  |
| genus.Fusicatenibacter.id.11305 | | rs4378146 | | A | | C | | | -0.0616691 | | | 0.012525884 | | 7.20E-07 | | -0.0005599 | | | 0.026199 | | | 0.982948 | | |  |  |  |  |
| genus.Fusicatenibacter.id.11305 | | rs60254196 | | A | | G | | | -0.0492427 | | | 0.010936491 | | 5.47E-06 | | 0.003384 | | | 0.022975 | | | 0.8829 | | |  |  |  |  |
| genus.Fusicatenibacter.id.11305 | | rs62187631 | | T | | C | | | -0.0710571 | | | 0.015924014 | | 4.55E-06 | | 0.019614 | | | 0.028986 | | | 0.498616 | | |  |  |  |  |
| genus.Fusicatenibacter.id.11305 | | rs62353480 | | A | | G | | | -0.0701397 | | | 0.014558884 | | 1.57E-06 | | 0.0354903 | | | 0.030586 | | | 0.245915 | | |  |  |  |  |
| genus.Fusicatenibacter.id.11305 | | rs6515626 | | G | | A | | | 0.141575 | | | 0.031355872 | | 7.29E-06 | | -0.0411225 | | | 0.045566 | | | 0.366799 | | |  |  |  |  |
| genus.Fusicatenibacter.id.11305 | | rs704418 | | T | | C | | | 0.0739124 | | | 0.015107283 | | 7.77E-07 | | -0.0085703 | | | 0.034692 | | | 0.804879 | | |  |  |  |  |
| genus.Fusicatenibacter.id.11305 | | rs7069626 | | T | | C | | | 0.0509014 | | | 0.011138084 | | 5.42E-06 | | 0.0074974 | | | 0.024324 | | | 0.757905 | | |  |  |  |  |
| genus.Fusicatenibacter.id.11305 | | rs73103914 | | A | | G | | | -0.0597324 | | | 0.013445773 | | 8.30E-06 | | 0.0150807 | | | 0.031944 | | | 0.636857 | | |  |  |  |  |
| genus.Fusicatenibacter.id.11305 | | rs792108 | | T | | C | | | -0.0508345 | | | 0.011384326 | | 8.50E-06 | | 0.0651933 | | | 0.023102 | | | 0.00477254 | | |  |  |  |  |
| genus.Fusicatenibacter.id.11305 | | rs8028026 | | A | | G | | | -0.0792146 | | | 0.018050473 | | 8.06E-06 | | 0.0298983 | | | 0.039509 | | | 0.449206 | | |  |  |  |  |
| genus.Fusicatenibacter.id.11305 | | rs8063430 | | T | | C | | | -0.1040265 | | | 0.022216952 | | 4.93E-06 | | -0.0142998 | | | 0.052123 | | | 0.783816 | | |  |  |  |  |
| genus.Fusicatenibacter.id.11305 | | rs9905659 | | G | | A | | | -0.061617 | | | 0.013657585 | | 7.31E-06 | | 0.0292228 | | | 0.029615 | | | 0.323763 | | |  |  |  |  |
| genus.Gordonibacter.id.821 | | rs117347059 | | G | | C | | | -0.128261 | | | 0.028512264 | | 9.17E-06 | | 0.0372606 | | | 0.029294 | | | 0.203389 | | |  |  |  |  |
| genus.Gordonibacter.id.821 | | rs13412653 | | A | | C | | | 0.1075974 | | | 0.023922776 | | 8.61E-06 | | 0.0079134 | | | 0.023583 | | | 0.737203 | | |  |  |  |  |
| genus.Gordonibacter.id.821 | | rs16955299 | | G | | A | | | -0.1964229 | | | 0.043353771 | | 6.37E-06 | | 0.10195 | | | 0.03717 | | | 0.00609214 | | |  |  |  |  |
| genus.Gordonibacter.id.821 | | rs322296 | | G | | A | | | 0.1786895 | | | 0.037721957 | | 4.02E-06 | | -0.0036709 | | | 0.043659 | | | 0.932992 | | |  |  |  |  |
| genus.Gordonibacter.id.821 | | rs35042269 | | C | | A | | | -0.1802927 | | | 0.040329634 | | 8.11E-06 | | 0.0241323 | | | 0.036112 | | | 0.503967 | | |  |  |  |  |
| genus.Gordonibacter.id.821 | | rs3765837 | | T | | G | | | -0.1907285 | | | 0.043360843 | | 7.17E-06 | | -0.0479571 | | | 0.04426 | | | 0.278574 | | |  |  |  |  |
| genus.Gordonibacter.id.821 | | rs4596722 | | A | | G | | | 0.1029083 | | | 0.023157103 | | 9.06E-06 | | -0.0088826 | | | 0.022828 | | | 0.697193 | | |  |  |  |  |
| genus.Gordonibacter.id.821 | | rs71545975 | | A | | G | | | -0.153972 | | | 0.033891756 | | 7.04E-06 | | -0.0347672 | | | 0.030252 | | | 0.250445 | | |  |  |  |  |
| genus.Gordonibacter.id.821 | | rs7220558 | | A | | T | | | 0.1168841 | | | 0.02348337 | | 6.71E-07 | | 0.0040832 | | | 0.02324 | | | 0.86053 | | |  |  |  |  |
| genus.Gordonibacter.id.821 | | rs72714787 | | C | | A | | | 0.1814048 | | | 0.037710172 | | 1.43E-06 | | 0.0019954 | | | 0.033612 | | | 0.95266 | | |  |  |  |  |
| genus.Gordonibacter.id.821 | | rs72939513 | | A | | G | | | -0.2139896 | | | 0.049059194 | | 7.98E-06 | | 0.0529185 | | | 0.052048 | | | 0.309284 | | |  |  |  |  |
| genus.Gordonibacter.id.821 | | rs7294633 | | C | | T | | | 0.12867 | | | 0.024994637 | | 3.44E-07 | | -0.0057694 | | | 0.025645 | | | 0.822002 | | |  |  |  |  |
| genus.Gordonibacter.id.821 | | rs76287110 | | A | | T | | | -0.2429649 | | | 0.046617731 | | 1.67E-07 | | 0.0432082 | | | 0.041855 | | | 0.301911 | | |  |  |  |  |
| genus.Gordonibacter.id.821 | | rs768830 | | G | | A | | | 0.1498524 | | | 0.033331454 | | 7.76E-06 | | -0.0153785 | | | 0.031489 | | | 0.625286 | | |  |  |  |  |
| genus.Haemophilus.id.3698 | | rs10781340 | | G | | A | | | 0.0948915 | | | 0.020322072 | | 4.32E-06 | | 0.0216024 | | | 0.034619 | | | 0.532622 | | |  |  |  |  |
| genus.Haemophilus.id.3698 | | rs10840326 | | C | | G | | | -0.0677384 | | | 0.015146258 | | 7.37E-06 | | 0.0174239 | | | 0.023778 | | | 0.463691 | | |  |  |  |  |
| genus.Haemophilus.id.3698 | | rs111582866 | | G | | A | | | -0.1242654 | | | 0.026016016 | | 1.27E-06 | | 0.0114829 | | | 0.040586 | | | 0.777233 | | |  |  |  |  |
| genus.Haemophilus.id.3698 | | rs12191680 | | C | | G | | | 0.1065443 | | | 0.020032321 | | 1.47E-07 | | -0.0219853 | | | 0.037942 | | | 0.562292 | | |  |  |  |  |
| genus.Haemophilus.id.3698 | | rs12876183 | | T | | A | | | 0.0749201 | | | 0.016710973 | | 9.62E-06 | | -0.0281011 | | | 0.024115 | | | 0.243889 | | |  |  |  |  |
| genus.Haemophilus.id.3698 | | rs35509 | | G | | A | | | 0.128249 | | | 0.026877911 | | 2.01E-06 | | -0.0455716 | | | 0.057175 | | | 0.42542 | | |  |  |  |  |
| genus.Haemophilus.id.3698 | | rs4822728 | | T | | C | | | 0.0705861 | | | 0.015138761 | | 3.48E-06 | | 0.0149561 | | | 0.022802 | | | 0.511873 | | |  |  |  |  |
| genus.Haemophilus.id.3698 | | rs56310940 | | G | | C | | | -0.1083933 | | | 0.024705186 | | 7.23E-06 | | 0.037558 | | | 0.03487 | | | 0.281447 | | |  |  |  |  |
| genus.Haemophilus.id.3698 | | rs76022354 | | C | | T | | | 0.2446376 | | | 0.050550437 | | 1.83E-06 | | 0.0256444 | | | 0.052476 | | | 0.625059 | | |  |  |  |  |
| genus.Haemophilus.id.3698 | | rs78909003 | | T | | C | | | -0.2462557 | | | 0.050392389 | | 1.67E-06 | | -0.010464 | | | 0.050245 | | | 0.835027 | | |  |  |  |  |
| genus.Haemophilus.id.3698 | | rs9328464 | | T | | C | | | 0.0723095 | | | 0.014879949 | | 1.42E-06 | | -0.0194025 | | | 0.022909 | | | 0.397018 | | |  |  |  |  |
| genus.Haemophilus.id.3698 | | rs9382510 | | C | | T | | | -0.093521 | | | 0.017264852 | | 7.12E-08 | | 0.0356173 | | | 0.026184 | | | 0.173739 | | |  |  |  |  |
| genus.Haemophilus.id.3698 | | rs9574096 | | A | | T | | | -0.0736375 | | | 0.015522726 | | 2.18E-06 | | -0.0013854 | | | 0.023638 | | | 0.953265 | | |  |  |  |  |
| genus.Haemophilus.id.3698 | | rs9895850 | | T | | C | | | -0.1929569 | | | 0.041675519 | | 2.14E-06 | | 0.0160318 | | | 0.055788 | | | 0.773829 | | |  |  |  |  |
| genus.Holdemanella.id.11393 | | rs12415649 | | G | | C | | | 0.0839384 | | | 0.019056694 | | 7.88E-06 | | 0.0330662 | | | 0.027523 | | | 0.229591 | | |  |  |  |  |
| genus.Holdemanella.id.11393 | | rs12513188 | | G | | A | | | 0.0903906 | | | 0.019527621 | | 4.65E-06 | | 0.0156007 | | | 0.025808 | | | 0.545514 | | |  |  |  |  |
| genus.Holdemanella.id.11393 | | rs17586763 | | T | | C | | | -0.2272845 | | | 0.051013457 | | 7.72E-06 | | 0.0486122 | | | 0.052131 | | | 0.351075 | | |  |  |  |  |
| genus.Holdemanella.id.11393 | | rs1830029 | | C | | G | | | -0.0954096 | | | 0.021093039 | | 5.35E-06 | | -0.0486446 | | | 0.027373 | | | 0.0755475 | | |  |  |  |  |
| genus.Holdemanella.id.11393 | | rs1926302 | | G | | A | | | -0.1079718 | | | 0.023142056 | | 7.50E-06 | | 0.0368372 | | | 0.027444 | | | 0.179514 | | |  |  |  |  |
| genus.Holdemanella.id.11393 | | rs34187114 | | C | | A | | | -0.1045052 | | | 0.022600956 | | 5.13E-06 | | -0.0295513 | | | 0.036266 | | | 0.415158 | | |  |  |  |  |
| genus.Holdemanella.id.11393 | | rs35228298 | | G | | A | | | 0.0934832 | | | 0.020288588 | | 7.30E-06 | | -0.0049616 | | | 0.031326 | | | 0.874152 | | |  |  |  |  |
| genus.Holdemanella.id.11393 | | rs4541991 | | T | | C | | | -0.0927334 | | | 0.019443459 | | 2.10E-06 | | 0.0064396 | | | 0.024393 | | | 0.791786 | | |  |  |  |  |
| genus.Holdemanella.id.11393 | | rs607782 | | T | | C | | | -0.0854214 | | | 0.017251511 | | 7.19E-07 | | -0.007612 | | | 0.02368 | | | 0.74787 | | |  |  |  |  |
| genus.Holdemanella.id.11393 | | rs62113381 | | T | | C | | | -0.1054472 | | | 0.023202912 | | 5.54E-06 | | -0.0268142 | | | 0.033882 | | | 0.428707 | | |  |  |  |  |
| genus.Holdemanella.id.11393 | | rs73011279 | | T | | C | | | -0.0961669 | | | 0.019933645 | | 1.36E-06 | | -0.0059205 | | | 0.027253 | | | 0.828017 | | |  |  |  |  |
| genus.Holdemanella.id.11393 | | rs75764681 | | T | | C | | | -0.2831026 | | | 0.059899873 | | 1.94E-06 | | -0.0946872 | | | 0.055823 | | | 0.0898463 | | |  |  |  |  |
| genus.Holdemanella.id.11393 | | rs761624 | | C | | G | | | 0.0959801 | | | 0.017953732 | | 1.38E-07 | | -0.0137807 | | | 0.024812 | | | 0.578611 | | |  |  |  |  |
| genus.Holdemanella.id.11393 | | rs8113760 | | G | | A | | | 0.078999 | | | 0.017340046 | | 4.62E-06 | | 0.0161956 | | | 0.024715 | | | 0.512272 | | |  |  |  |  |
| genus.Holdemania.id.2157 | | rs10885477 | | T | | C | | | -0.1351366 | | | 0.030189444 | | 8.60E-06 | | 0.0268644 | | | 0.052403 | | | 0.608192 | | |  |  |  |  |
| genus.Holdemania.id.2157 | | rs11080063 | | G | | A | | | -0.0665238 | | | 0.014983728 | | 6.67E-06 | | 0.0134139 | | | 0.023213 | | | 0.563356 | | |  |  |  |  |
| genus.Holdemania.id.2157 | | rs111745969 | | A | | G | | | 0.1206769 | | | 0.026578103 | | 3.71E-06 | | -0.0172166 | | | 0.033404 | | | 0.606268 | | |  |  |  |  |
| genus.Holdemania.id.2157 | | rs113593397 | | A | | G | | | -0.1289347 | | | 0.028252607 | | 9.36E-06 | | -0.0186442 | | | 0.038662 | | | 0.62964 | | |  |  |  |  |
| genus.Holdemania.id.2157 | | rs116500994 | | G | | T | | | -0.1375505 | | | 0.029338596 | | 2.34E-06 | | -0.0508762 | | | 0.054385 | | | 0.349541 | | |  |  |  |  |
| genus.Holdemania.id.2157 | | rs12701617 | | A | | G | | | -0.0660617 | | | 0.014942681 | | 9.52E-06 | | -0.0156716 | | | 0.022895 | | | 0.493658 | | |  |  |  |  |
| genus.Holdemania.id.2157 | | rs150096134 | | T | | A | | | 0.1621393 | | | 0.033208945 | | 2.38E-06 | | -0.0161139 | | | 0.038202 | | | 0.673164 | | |  |  |  |  |
| genus.Holdemania.id.2157 | | rs1867876 | | T | | C | | | 0.0842919 | | | 0.016219181 | | 2.74E-07 | | 0.0271392 | | | 0.024938 | | | 0.276477 | | |  |  |  |  |
| genus.Holdemania.id.2157 | | rs41438744 | | C | | G | | | -0.1248705 | | | 0.02701295 | | 2.44E-06 | | 0.0008362 | | | 0.041986 | | | 0.984111 | | |  |  |  |  |
| genus.Holdemania.id.2157 | | rs4146507 | | C | | T | | | 0.079488 | | | 0.017700231 | | 7.23E-06 | | -0.042125 | | | 0.026713 | | | 0.114802 | | |  |  |  |  |
| genus.Holdemania.id.2157 | | rs55888180 | | C | | G | | | 0.128671 | | | 0.028279464 | | 5.89E-06 | | 0.0620167 | | | 0.050738 | | | 0.221597 | | |  |  |  |  |
| genus.Holdemania.id.2157 | | rs73139538 | | G | | A | | | -0.1485936 | | | 0.032746911 | | 7.77E-06 | | 0.0077709 | | | 0.067692 | | | 0.908605 | | |  |  |  |  |
| genus.Holdemania.id.2157 | | rs77293403 | | A | | G | | | 0.1645565 | | | 0.034177476 | | 1.77E-06 | | 0.0424715 | | | 0.061499 | | | 0.489817 | | |  |  |  |  |
| genus.Holdemania.id.2157 | | rs80149660 | | C | | T | | | -0.2329874 | | | 0.051918311 | | 6.04E-06 | | 0.0289131 | | | 0.05598 | | | 0.605511 | | |  |  |  |  |
| genus.Holdemania.id.2157 | | rs9500080 | | C | | T | | | 0.0926764 | | | 0.01788862 | | 4.09E-07 | | 0.0406975 | | | 0.030285 | | | 0.179011 | | |  |  |  |  |
| genus.Holdemania.id.2157 | | rs9529719 | | T | | C | | | 0.074038 | | | 0.016049433 | | 5.97E-06 | | 0.0198556 | | | 0.024465 | | | 0.417018 | | |  |  |  |  |
| genus.Holdemania.id.2157 | | rs967319 | | T | | C | | | 0.0788638 | | | 0.017674402 | | 8.38E-06 | | -0.001774 | | | 0.026677 | | | 0.946979 | | |  |  |  |  |
| genus.Howardella.id.2000 | | rs10048062 | | C | | T | | | -0.1473522 | | | 0.033653121 | | 8.59E-06 | | -0.0518669 | | | 0.040102 | | | 0.195884 | | |  |  |  |  |
| genus.Howardella.id.2000 | | rs12452946 | | A | | G | | | -0.1058331 | | | 0.022894084 | | 3.80E-06 | | -0.0455022 | | | 0.022814 | | | 0.046102 | | |  |  |  |  |
| genus.Howardella.id.2000 | | rs1484873 | | A | | G | | | -0.2278312 | | | 0.046335026 | | 2.56E-06 | | 0.0132587 | | | 0.03186 | | | 0.677292 | | |  |  |  |  |
| genus.Howardella.id.2000 | | rs17167098 | | G | | A | | | -0.1693657 | | | 0.035206781 | | 1.12E-06 | | 0.0407953 | | | 0.033224 | | | 0.219494 | | |  |  |  |  |
| genus.Howardella.id.2000 | | rs2154047 | | C | | A | | | -0.192557 | | | 0.042009328 | | 9.97E-06 | | -0.0471694 | | | 0.04014 | | | 0.23994 | | |  |  |  |  |
| genus.Howardella.id.2000 | | rs36081916 | | T | | C | | | -0.1812297 | | | 0.040298718 | | 4.70E-06 | | -0.0540226 | | | 0.040702 | | | 0.184416 | | |  |  |  |  |
| genus.Howardella.id.2000 | | rs3791893 | | A | | G | | | 0.1470353 | | | 0.034023014 | | 9.50E-06 | | -0.114976 | | | 0.03353 | | | 0.00060566 | | |  |  |  |  |
| genus.Howardella.id.2000 | | rs609430 | | T | | G | | | -0.1120454 | | | 0.023932786 | | 3.34E-06 | | -0.0006408 | | | 0.023895 | | | 0.978606 | | |  |  |  |  |
| genus.Howardella.id.2000 | | rs61771805 | | A | | T | | | -0.1367787 | | | 0.029681112 | | 4.03E-06 | | -0.000328 | | | 0.035659 | | | 0.99266 | | |  |  |  |  |
| genus.Howardella.id.2000 | | rs672217 | | G | | A | | | 0.1641457 | | | 0.034999266 | | 3.52E-06 | | 0.0355186 | | | 0.028694 | | | 0.215775 | | |  |  |  |  |
| genus.Hungatella.id.11306 | | rs10044993 | | C | | A | | | 0.139547 | | | 0.031674777 | | 8.07E-06 | | -0.0095781 | | | 0.040928 | | | 0.814965 | | |  |  |  |  |
| genus.Hungatella.id.11306 | | rs13128780 | | T | | C | | | -0.1497253 | | | 0.031277743 | | 1.75E-06 | | 0.0311899 | | | 0.029182 | | | 0.285158 | | |  |  |  |  |
| genus.Hungatella.id.11306 | | rs13249325 | | T | | G | | | -0.1000226 | | | 0.022588331 | | 9.69E-06 | | -0.0288344 | | | 0.022887 | | | 0.207727 | | |  |  |  |  |
| genus.Hungatella.id.11306 | | rs17092615 | | G | | A | | | 0.1522347 | | | 0.033786729 | | 7.38E-06 | | -0.0431567 | | | 0.033145 | | | 0.192891 | | |  |  |  |  |
| genus.Hungatella.id.11306 | | rs72759041 | | G | | T | | | -0.126025 | | | 0.028224238 | | 3.86E-06 | | -0.0022002 | | | 0.028186 | | | 0.937779 | | |  |  |  |  |
| genus.Intestinibacter.id.11345 | | rs10805326 | | G | | A | | | 0.0775151 | | | 0.013966468 | | 3.55E-08 | | 0.0100763 | | | 0.025064 | | | 0.687671 | | |  |  |  |  |
| genus.Intestinibacter.id.11345 | | rs11109097 | | C | | T | | | 0.0624272 | | | 0.013853923 | | 5.49E-06 | | 0.0251743 | | | 0.023018 | | | 0.274101 | | |  |  |  |  |
| genus.Intestinibacter.id.11345 | | rs118030283 | | G | | A | | | -0.151829 | | | 0.032447117 | | 2.67E-06 | | -0.0233804 | | | 0.054826 | | | 0.669781 | | |  |  |  |  |
| genus.Intestinibacter.id.11345 | | rs16938435 | | T | | C | | | -0.1121885 | | | 0.023543887 | | 1.80E-06 | | -0.0255198 | | | 0.038651 | | | 0.509088 | | |  |  |  |  |
| genus.Intestinibacter.id.11345 | | rs2098844 | | C | | T | | | -0.0575295 | | | 0.012841562 | | 6.79E-06 | | 0.0038691 | | | 0.023699 | | | 0.870314 | | |  |  |  |  |
| genus.Intestinibacter.id.11345 | | rs2702387 | | A | | G | | | 0.0608559 | | | 0.013214549 | | 4.26E-06 | | -0.0118428 | | | 0.023174 | | | 0.609328 | | |  |  |  |  |
| genus.Intestinibacter.id.11345 | | rs4327025 | | G | | A | | | -0.0810346 | | | 0.015439666 | | 1.64E-07 | | -0.0185485 | | | 0.029299 | | | 0.526686 | | |  |  |  |  |
| genus.Intestinibacter.id.11345 | | rs447950 | | A | | G | | | 0.0628342 | | | 0.01366523 | | 5.64E-06 | | -0.0143226 | | | 0.023615 | | | 0.544178 | | |  |  |  |  |
| genus.Intestinibacter.id.11345 | | rs478972 | | T | | C | | | -0.142681 | | | 0.02971163 | | 1.82E-06 | | -0.0531716 | | | 0.039529 | | | 0.17858 | | |  |  |  |  |
| genus.Intestinibacter.id.11345 | | rs6062862 | | A | | G | | | 0.0924508 | | | 0.020465817 | | 6.68E-06 | | 0.0488183 | | | 0.041483 | | | 0.23926 | | |  |  |  |  |
| genus.Intestinibacter.id.11345 | | rs62430350 | | T | | C | | | 0.1510957 | | | 0.035147611 | | 6.84E-06 | | 0.0161814 | | | 0.06061 | | | 0.789487 | | |  |  |  |  |
| genus.Intestinibacter.id.11345 | | rs68093214 | | C | | T | | | 0.0662258 | | | 0.014987729 | | 9.26E-06 | | 0.0124515 | | | 0.026641 | | | 0.64023 | | |  |  |  |  |
| genus.Intestinibacter.id.11345 | | rs6875660 | | C | | T | | | 0.0890455 | | | 0.01939052 | | 3.06E-06 | | 0.0282585 | | | 0.048189 | | | 0.557599 | | |  |  |  |  |
| genus.Intestinibacter.id.11345 | | rs893394 | | G | | A | | | 0.0583322 | | | 0.013072938 | | 7.85E-06 | | -0.0344223 | | | 0.02322 | | | 0.138225 | | |  |  |  |  |
| genus.Intestinibacter.id.11345 | | rs9348442 | | C | | T | | | 0.099082 | | | 0.022162526 | | 6.26E-06 | | 0.0508803 | | | 0.034583 | | | 0.141219 | | |  |  |  |  |
| genus.Intestinimonas.id.2062 | | rs1000888 | | C | | G | | | -0.0585038 | | | 0.01321901 | | 9.71E-06 | | 0.0429309 | | | 0.024195 | | | 0.0760064 | | |  |  |  |  |
| genus.Intestinimonas.id.2062 | | rs10262702 | | T | | C | | | 0.0918017 | | | 0.019488468 | | 2.06E-06 | | 0.0095741 | | | 0.03471 | | | 0.78268 | | |  |  |  |  |
| genus.Intestinimonas.id.2062 | | rs11258178 | | A | | G | | | 0.0660742 | | | 0.013413886 | | 6.98E-07 | | -0.0247013 | | | 0.022881 | | | 0.280341 | | |  |  |  |  |
| genus.Intestinimonas.id.2062 | | rs11928843 | | G | | A | | | -0.0607331 | | | 0.013203515 | | 4.21E-06 | | -0.032086 | | | 0.022935 | | | 0.161814 | | |  |  |  |  |
| genus.Intestinimonas.id.2062 | | rs12226153 | | A | | G | | | -0.1511425 | | | 0.030692229 | | 5.12E-07 | | -0.076278 | | | 0.097971 | | | 0.436229 | | |  |  |  |  |
| genus.Intestinimonas.id.2062 | | rs12566247 | | T | | A | | | 0.0637084 | | | 0.013535548 | | 2.19E-06 | | -0.0470269 | | | 0.023103 | | | 0.0417965 | | |  |  |  |  |
| genus.Intestinimonas.id.2062 | | rs17067892 | | C | | T | | | 0.1071938 | | | 0.025001344 | | 6.38E-06 | | 0.0080684 | | | 0.040347 | | | 0.8415 | | |  |  |  |  |
| genus.Intestinimonas.id.2062 | | rs1859797 | | G | | A | | | 0.060368 | | | 0.013179253 | | 4.12E-06 | | 0.0226135 | | | 0.022937 | | | 0.324184 | | |  |  |  |  |
| genus.Intestinimonas.id.2062 | | rs2276760 | | A | | G | | | -0.0685234 | | | 0.015254563 | | 7.84E-06 | | -0.0128626 | | | 0.026727 | | | 0.630336 | | |  |  |  |  |
| genus.Intestinimonas.id.2062 | | rs2731794 | | C | | T | | | 0.1206321 | | | 0.025752925 | | 1.92E-06 | | -0.0419394 | | | 0.06241 | | | 0.501581 | | |  |  |  |  |
| genus.Intestinimonas.id.2062 | | rs2930225 | | G | | T | | | 0.0729524 | | | 0.015294848 | | 1.35E-06 | | 0.0256017 | | | 0.027091 | | | 0.344653 | | |  |  |  |  |
| genus.Intestinimonas.id.2062 | | rs4113676 | | A | | C | | | -0.2185069 | | | 0.049015185 | | 7.42E-06 | | 0.0999653 | | | 0.10029 | | | 0.31888 | | |  |  |  |  |
| genus.Intestinimonas.id.2062 | | rs4784055 | | T | | C | | | -0.175324 | | | 0.038599023 | | 8.72E-07 | | -0.154142 | | | 0.054853 | | | 0.00495268 | | |  |  |  |  |
| genus.Intestinimonas.id.2062 | | rs62240188 | | G | | A | | | 0.1300736 | | | 0.026717884 | | 2.20E-06 | | 0.0558456 | | | 0.039529 | | | 0.157722 | | |  |  |  |  |
| genus.Intestinimonas.id.2062 | | rs6934519 | | C | | T | | | 0.0692373 | | | 0.015115352 | | 8.57E-06 | | -0.0181347 | | | 0.026061 | | | 0.486515 | | |  |  |  |  |
| genus.Intestinimonas.id.2062 | | rs716604 | | A | | G | | | 0.0818068 | | | 0.016599238 | | 8.57E-07 | | -0.0141634 | | | 0.027178 | | | 0.602278 | | |  |  |  |  |
| genus.Intestinimonas.id.2062 | | rs7170984 | | T | | C | | | -0.0658116 | | | 0.014076733 | | 2.98E-06 | | 0.0047219 | | | 0.025437 | | | 0.852734 | | |  |  |  |  |
| genus.Intestinimonas.id.2062 | | rs72982915 | | C | | T | | | 0.1831579 | | | 0.040274272 | | 4.91E-06 | | 0.0799988 | | | 0.049615 | | | 0.106874 | | |  |  |  |  |
| genus.Intestinimonas.id.2062 | | rs994794 | | G | | C | | | -0.1419048 | | | 0.031507362 | | 7.31E-06 | | -0.0057873 | | | 0.065709 | | | 0.929817 | | |  |  |  |  |
| genus.Lachnoclostridium.id.11308 | | rs1031599 | | G | T | | | -0.078627 | | | 0.017564435 | | 6.31E-06 | | 0.0772038 | | | 0.046835 | | | 0.0992636 | |  |  |  |  |  |  |
| genus.Lachnoclostridium.id.11308 | | rs12566975 | | T | C | | | -0.0468097 | | | 0.010578675 | | 9.57E-06 | | -0.0169068 | | | 0.022915 | | | 0.460631 | |  |  |  |  |  |  |
| genus.Lachnoclostridium.id.11308 | | rs1528479 | | G | A | | | -0.0497798 | | | 0.011191926 | | 9.64E-06 | | -0.0082899 | | | 0.023636 | | | 0.725785 | |  |  |  |  |  |  |
| genus.Lachnoclostridium.id.11308 | | rs1997204 | | T | C | | | -0.1080748 | | | 0.024202203 | | 5.97E-06 | | -0.0101221 | | | 0.055401 | | | 0.855029 | |  |  |  |  |  |  |
| genus.Lachnoclostridium.id.11308 | | rs2385421 | | A | G | | | 0.0746186 | | | 0.018073408 | | 7.14E-06 | | 0.0502604 | | | 0.035314 | | | 0.154666 | |  |  |  |  |  |  |
| genus.Lachnoclostridium.id.11308 | | rs3821998 | | C | A | | | -0.0864066 | | | 0.019251946 | | 6.72E-06 | | -0.031704 | | | 0.037407 | | | 0.39669 | |  |  |  |  |  |  |
| genus.Lachnoclostridium.id.11308 | | rs4738679 | | G | A | | | -0.0520267 | | | 0.011404049 | | 4.42E-06 | | 0.029051 | | | 0.023477 | | | 0.215937 | |  |  |  |  |  |  |
| genus.Lachnoclostridium.id.11308 | | rs6112314 | | A | C | | | -0.0561715 | | | 0.010817419 | | 2.43E-07 | | 0.0232383 | | | 0.024219 | | | 0.3373 | |  |  |  |  |  |  |
| genus.Lachnoclostridium.id.11308 | | rs615997 | | T | C | | | 0.0511752 | | | 0.010649056 | | 2.03E-06 | | -0.0178418 | | | 0.022735 | | | 0.43258 | |  |  |  |  |  |  |
| genus.Lachnoclostridium.id.11308 | | rs61915992 | | A | T | | | 0.0803876 | | | 0.01722087 | | 2.67E-06 | | -0.0007512 | | | 0.03242 | | | 0.981514 | |  |  |  |  |  |  |
| genus.Lachnoclostridium.id.11308 | | rs62028349 | | G | C | | | 0.0469989 | | | 0.010597086 | | 9.17E-06 | | -0.034666 | | | 0.023049 | | | 0.13258 | |  |  |  |  |  |  |
| genus.Lachnoclostridium.id.11308 | | rs62285313 | | A | G | | | 0.0864203 | | | 0.018156544 | | 1.58E-06 | | -0.10799 | | | 0.039741 | | | 0.00658112 | |  |  |  |  |  |  |
| genus.Lachnoclostridium.id.11308 | | rs72829893 | | G | T | | | 0.1174724 | | | 0.026810315 | | 5.58E-06 | | 0.042834 | | | 0.037363 | | | 0.251622 | |  |  |  |  |  |  |
| genus.Lachnoclostridium.id.11308 | | rs78068103 | | A | G | | | 0.0886199 | | | 0.019424795 | | 3.67E-06 | | -0.0492556 | | | 0.035652 | | | 0.167102 | |  |  |  |  |  |  |
| genus.Lachnoclostridium.id.11308 | | rs789029 | | C | T | | | -0.0641288 | | | 0.013797406 | | 3.75E-06 | | 0.0339802 | | | 0.032791 | | | 0.300074 | |  |  |  |  |  |  |
| genus.Lachnospira.id.2004 | | rs13157098 | | A | G | | | -0.0768058 | | | 0.015531203 | | 5.99E-07 | | 0.052224 | | | 0.030946 | | | 0.091485 | |  |  |  |  |  |  |
| genus.Lachnospira.id.2004 | | rs159484 | | G | A | | | 0.0794668 | | | 0.017699872 | | 6.68E-06 | | -0.0338022 | | | 0.043769 | | | 0.439946 | |  |  |  |  |  |  |
| genus.Lachnospira.id.2004 | | rs2326833 | | C | G | | | -0.078495 | | | 0.017109015 | | 4.60E-06 | | 0.0454326 | | | 0.031361 | | | 0.147424 | |  |  |  |  |  |  |
| genus.Lachnospira.id.2004 | | rs2520509 | | A | G | | | 0.0519063 | | | 0.011577959 | | 7.42E-06 | | 0.0111832 | | | 0.025114 | | | 0.656104 | |  |  |  |  |  |  |
| genus.Lachnospira.id.2004 | | rs4686798 | | T | C | | | 0.0531826 | | | 0.011374743 | | 2.74E-06 | | -0.0200347 | | | 0.023755 | | | 0.39902 | |  |  |  |  |  |  |
| genus.Lachnospira.id.2004 | | rs4923324 | | G | A | | | -0.0617321 | | | 0.013338657 | | 2.44E-06 | | 0.0007413 | | | 0.030984 | | | 0.980912 | |  |  |  |  |  |  |
| genus.Lachnospira.id.2004 | | rs56791201 | | T | C | | | 0.051823 | | | 0.011069673 | | 2.93E-06 | | 0.0018286 | | | 0.023752 | | | 0.938634 | |  |  |  |  |  |  |
| genus.LachnospiraceaeFCS020group.id.11314 | | rs10093861 | | G | A | | | -0.0568869 | | | 0.012115188 | | 3.06E-06 | | -0.0207319 | | | 0.023229 | | | 0.372124 | |  |  |  |  |  |  |
| genus.LachnospiraceaeFCS020group.id.11314 | | rs113859143 | | G | C | | | -0.1089063 | | | 0.024205472 | | 2.55E-06 | | 0.0227575 | | | 0.049395 | | | 0.644997 | |  |  |  |  |  |  |
| genus.LachnospiraceaeFCS020group.id.11314 | | rs12078956 | | C | G | | | 0.106101 | | | 0.022290421 | | 2.15E-06 | | 0.0557953 | | | 0.036832 | | | 0.129803 | |  |  |  |  |  |  |
| genus.LachnospiraceaeFCS020group.id.11314 | | rs1254846 | | G | A | | | 0.1059626 | | | 0.023250118 | | 5.60E-06 | | -0.0441658 | | | 0.032576 | | | 0.175172 | |  |  |  |  |  |  |
| genus.LachnospiraceaeFCS020group.id.11314 | | rs1363769 | | T | C | | | -0.2006284 | | | 0.044937217 | | 1.58E-06 | | 5.18E-05 | | | 0.064619 | | | 0.999361 | |  |  |  |  |  |  |
| genus.LachnospiraceaeFCS020group.id.11314 | | rs2322265 | | C | T | | | -0.0666305 | | | 0.014157654 | | 5.21E-06 | | 0.023102 | | | 0.025967 | | | 0.373644 | |  |  |  |  |  |  |
| genus.LachnospiraceaeFCS020group.id.11314 | | rs2862811 | | T | C | | | 0.0564927 | | | 0.012173604 | | 3.92E-06 | | -0.0095247 | | | 0.024933 | | | 0.702448 | |  |  |  |  |  |  |
| genus.LachnospiraceaeFCS020group.id.11314 | | rs35035870 | | T | C | | | -0.1906153 | | | 0.041440269 | | 2.62E-06 | | 0.0344176 | | | 0.058876 | | | 0.55883 | |  |  |  |  |  |  |
| genus.LachnospiraceaeFCS020group.id.11314 | | rs369444 | | C | G | | | 0.1254813 | | | 0.025900786 | | 3.15E-06 | | -0.0101829 | | | 0.044149 | | | 0.817587 | |  |  |  |  |  |  |
| genus.LachnospiraceaeFCS020group.id.11314 | | rs3999074 | | G | T | | | -0.0550587 | | | 0.012184677 | | 6.55E-06 | | 0.0379153 | | | 0.022896 | | | 0.0977327 | |  |  |  |  |  |  |
| genus.LachnospiraceaeFCS020group.id.11314 | | rs4452603 | | T | G | | | 0.0604208 | | | 0.013596453 | | 8.98E-06 | | -0.028134 | | | 0.025867 | | | 0.276754 | |  |  |  |  |  |  |
| genus.LachnospiraceaeFCS020group.id.11314 | | rs62140927 | | C | T | | | 0.0613709 | | | 0.013105468 | | 2.38E-06 | | 0.0152083 | | | 0.024348 | | | 0.532211 | |  |  |  |  |  |  |
| genus.LachnospiraceaeFCS020group.id.11314 | | rs7249113 | | G | A | | | 0.0679483 | | | 0.013349587 | | 3.72E-07 | | 0.0023026 | | | 0.025051 | | | 0.926762 | |  |  |  |  |  |  |
| genus.LachnospiraceaeFCS020group.id.11314 | | rs72793667 | | A | G | | | -0.1168808 | | | 0.024658906 | | 1.63E-06 | | -0.012092 | | | 0.058894 | | | 0.837324 | |  |  |  |  |  |  |
| genus.LachnospiraceaeFCS020group.id.11314 | | rs9308097 | | A | G | | | 0.055381 | | | 0.012365944 | | 7.47E-06 | | -0.022493 | | | 0.022878 | | | 0.325513 | |  |  |  |  |  |  |
| genus.LachnospiraceaeFCS020group.id.11314 | | rs9788306 | | C | T | | | -0.062804 | | | 0.013074422 | | 1.39E-06 | | 0.0488792 | | | 0.025258 | | | 0.0529615 | |  |  |  |  |  |  |
| genus.LachnospiraceaeFCS020group.id.11314 | | rs9919338 | | G | C | | | -0.0553339 | | | 0.012062109 | | 4.91E-06 | | -0.0167619 | | | 0.022968 | | | 0.465515 | |  |  |  |  |  |  |
| genus.LachnospiraceaeNC2004group.id.11316 | | rs117467633 | | T | C | | | -0.1696875 | | | 0.038316857 | | 9.13E-06 | | 0.0720379 | | | 0.059013 | | | 0.222191 | |  |  |  |  |  |  |
| genus.LachnospiraceaeNC2004group.id.11316 | | rs12127733 | | G | A | | | 0.1151815 | | | 0.024600625 | | 3.11E-06 | | -0.0275836 | | | 0.029717 | | | 0.353306 | |  |  |  |  |  |  |
| genus.LachnospiraceaeNC2004group.id.11316 | | rs12208226 | | C | A | | | -0.1547391 | | | 0.034037233 | | 9.75E-06 | | 0.0489888 | | | 0.037051 | | | 0.186101 | |  |  |  |  |  |  |
| genus.LachnospiraceaeNC2004group.id.11316 | | rs12863463 | | G | A | | | -0.1563649 | | | 0.03453716 | | 6.04E-06 | | -0.0070961 | | | 0.042811 | | | 0.868351 | |  |  |  |  |  |  |
| genus.LachnospiraceaeNC2004group.id.11316 | | rs1331592 | | C | G | | | 0.0948806 | | | 0.02083688 | | 5.34E-06 | | -0.0362011 | | | 0.026875 | | | 0.177975 | |  |  |  |  |  |  |
| genus.LachnospiraceaeNC2004group.id.11316 | | rs17067076 | | G | A | | | -0.1546314 | | | 0.035219374 | | 5.61E-06 | | 0.0270255 | | | 0.035693 | | | 0.448945 | |  |  |  |  |  |  |
| genus.LachnospiraceaeNC2004group.id.11316 | | rs1928659 | | T | C | | | 0.102522 | | | 0.022644542 | | 6.17E-06 | | 0.0018761 | | | 0.028481 | | | 0.94748 | |  |  |  |  |  |  |
| genus.LachnospiraceaeNC2004group.id.11316 | | rs1929743 | | T | C | | | 0.0837206 | | | 0.019031847 | | 9.06E-06 | | 0.006952 | | | 0.024163 | | | 0.773567 | |  |  |  |  |  |  |
| genus.LachnospiraceaeNC2004group.id.11316 | | rs3756315 | | A | G | | | -0.0883469 | | | 0.01883968 | | 3.33E-06 | | 0.0036356 | | | 0.02498 | | | 0.884283 | |  |  |  |  |  |  |
| genus.LachnospiraceaeNC2004group.id.11316 | | rs6116753 | | G | A | | | 0.0994748 | | | 0.020913933 | | 2.92E-06 | | 0.0030737 | | | 0.029792 | | | 0.917826 | |  |  |  |  |  |  |
| genus.LachnospiraceaeND3007group.id.11317 | | rs2861203 | | G | A | | | 0.0572304 | | | 0.012727879 | | 7.37E-06 | | 0.0025924 | | | 0.025049 | | | 0.917571 | |  |  |  |  |  |  |
| genus.LachnospiraceaeND3007group.id.11317 | | rs72776675 | | T | C | | | -0.0647067 | | | 0.014797022 | | 8.72E-06 | | -0.0222651 | | | 0.030371 | | | 0.463492 | |  |  |  |  |  |  |
| genus.LachnospiraceaeND3007group.id.11317 | | rs9932954 | | A | G | | | -0.0561864 | | | 0.011598415 | | 1.25E-06 | | -0.0233356 | | | 0.024157 | | | 0.334035 | |  |  |  |  |  |  |
| genus.LachnospiraceaeNK4A136group.id.11319 | | rs10952110 | | G | T | | | 0.0487709 | | | 0.010961435 | | 9.08E-06 | | 0.0089914 | | | 0.023055 | | | 0.69654 | |  |  |  |  |  |  |
| genus.LachnospiraceaeNK4A136group.id.11319 | | rs11263806 | | A | G | | | -0.0524615 | | | 0.011675801 | | 5.07E-06 | | -0.0173955 | | | 0.024109 | | | 0.470573 | |  |  |  |  |  |  |
| genus.LachnospiraceaeNK4A136group.id.11319 | | rs12362320 | | G | C | | | 0.0573168 | | | 0.011546623 | | 8.04E-07 | | 0.0021828 | | | 0.0232 | | | 0.925043 | |  |  |  |  |  |  |
| genus.LachnospiraceaeNK4A136group.id.11319 | | rs12611395 | | A | G | | | -0.0902532 | | | 0.019965236 | | 5.83E-06 | | 0.0216143 | | | 0.037309 | | | 0.562361 | |  |  |  |  |  |  |
| genus.LachnospiraceaeNK4A136group.id.11319 | | rs160061 | | A | G | | | 0.0513831 | | | 0.010809407 | | 2.12E-06 | | 0.0299694 | | | 0.022811 | | | 0.188903 | |  |  |  |  |  |  |
| genus.LachnospiraceaeNK4A136group.id.11319 | | rs28540839 | | A | C | | | 0.0508285 | | | 0.011059105 | | 9.34E-06 | | 0.0163791 | | | 0.022844 | | | 0.473381 | |  |  |  |  |  |  |
| genus.LachnospiraceaeNK4A136group.id.11319 | | rs2880566 | | T | C | | | 0.0599578 | | | 0.013469261 | | 5.61E-06 | | 0.0075639 | | | 0.032357 | | | 0.815166 | |  |  |  |  |  |  |
| genus.LachnospiraceaeNK4A136group.id.11319 | | rs4955932 | | T | C | | | -0.0492283 | | | 0.010938782 | | 7.05E-06 | | 0.0169127 | | | 0.023549 | | | 0.472638 | |  |  |  |  |  |  |
| genus.LachnospiraceaeNK4A136group.id.11319 | | rs59805249 | | T | C | | | 0.0936162 | | | 0.020798243 | | 9.45E-06 | | -0.0129732 | | | 0.039405 | | | 0.741981 | |  |  |  |  |  |  |
| genus.LachnospiraceaeNK4A136group.id.11319 | | rs68104925 | | T | C | | | -0.0549104 | | | 0.011538497 | | 2.37E-06 | | 0.0535343 | | | 0.02482 | | | 0.0310127 | |  |  |  |  |  |  |
| genus.LachnospiraceaeNK4A136group.id.11319 | | rs7073658 | | T | G | | | -0.0499621 | | | 0.010969284 | | 5.27E-06 | | 0.0127406 | | | 0.022914 | | | 0.5782 | |  |  |  |  |  |  |
| genus.LachnospiraceaeNK4A136group.id.11319 | | rs73044693 | | A | G | | | -0.1075796 | | | 0.022988374 | | 3.57E-06 | | 0.0289447 | | | 0.044938 | | | 0.519511 | |  |  |  |  |  |  |
| genus.LachnospiraceaeNK4A136group.id.11319 | | rs7616165 | | G | T | | | -0.230542 | | | 0.048346456 | | 2.77E-06 | | 0.137922 | | | 0.073069 | | | 0.0590854 | |  |  |  |  |  |  |
| genus.LachnospiraceaeNK4A136group.id.11319 | | rs76193507 | | A | G | | | -0.2297302 | | | 0.049977561 | | 2.93E-06 | | 0.062669 | | | 0.040516 | | | 0.121918 | |  |  |  |  |  |  |
| genus.LachnospiraceaeNK4A136group.id.11319 | | rs7832116 | | A | G | | | -0.0714756 | | | 0.015170307 | | 3.57E-06 | | 0.0202253 | | | 0.034317 | | | 0.555612 | |  |  |  |  |  |  |
| genus.LachnospiraceaeNK4A136group.id.11319 | | rs954878 | | A | G | | | -0.0520656 | | | 0.010908221 | | 1.78E-06 | | 0.0353842 | | | 0.023628 | | | 0.134241 | |  |  |  |  |  |  |
| genus.LachnospiraceaeUCG001.id.11321 | | rs10815577 | | C | G | | | -0.0683706 | | | 0.014369051 | | 1.72E-06 | | 0.0138731 | | | 0.023091 | | | 0.54798 | |  |  |  |  |  |  |
| genus.LachnospiraceaeUCG001.id.11321 | | rs12131224 | | C | T | | | 0.1170954 | | | 0.025909955 | | 7.40E-06 | | -0.0227846 | | | 0.036607 | | | 0.533671 | |  |  |  |  |  |  |
| genus.LachnospiraceaeUCG001.id.11321 | | rs2050911 | | G | A | | | 0.0751466 | | | 0.015393431 | | 1.11E-06 | | 0.0150756 | | | 0.024117 | | | 0.53191 | |  |  |  |  |  |  |
| genus.LachnospiraceaeUCG001.id.11321 | | rs2371284 | | T | C | | | -0.0761893 | | | 0.017012675 | | 7.56E-06 | | -0.0090112 | | | 0.027152 | | | 0.73998 | |  |  |  |  |  |  |
| genus.LachnospiraceaeUCG001.id.11321 | | rs437876 | | T | C | | | 0.0784637 | | | 0.014476767 | | 7.17E-08 | | -0.012911 | | | 0.023955 | | | 0.589916 | |  |  |  |  |  |  |
| genus.LachnospiraceaeUCG001.id.11321 | | rs4981345 | | T | C | | | -0.0681973 | | | 0.014983138 | | 6.09E-06 | | -0.0588512 | | | 0.024307 | | | 0.0154696 | |  |  |  |  |  |  |
| genus.LachnospiraceaeUCG001.id.11321 | | rs573933 | | T | C | | | -0.1078985 | | | 0.023234631 | | 3.11E-06 | | -0.0214282 | | | 0.037137 | | | 0.563935 | |  |  |  |  |  |  |
| genus.LachnospiraceaeUCG001.id.11321 | | rs62496417 | | T | G | | | -0.0748504 | | | 0.016568056 | | 5.88E-06 | | 0.0219266 | | | 0.027787 | | | 0.43006 | |  |  |  |  |  |  |
| genus.LachnospiraceaeUCG001.id.11321 | | rs7213933 | | T | A | | | -0.0816269 | | | 0.018380006 | | 9.02E-06 | | 0.0125405 | | | 0.031387 | | | 0.689488 | |  |  |  |  |  |  |
| genus.LachnospiraceaeUCG001.id.11321 | | rs7341608 | | T | C | | | -0.0784757 | | | 0.017764768 | | 9.48E-06 | | 0.0642794 | | | 0.033539 | | | 0.0552968 | |  |  |  |  |  |  |
| genus.LachnospiraceaeUCG001.id.11321 | | rs74034332 | | G | A | | | 0.1680455 | | | 0.038263689 | | 3.33E-06 | | 0.0323312 | | | 0.047126 | | | 0.492678 | |  |  |  |  |  |  |
| genus.LachnospiraceaeUCG001.id.11321 | | rs78848836 | | A | G | | | -0.118868 | | | 0.025975321 | | 3.38E-06 | | 0.0138349 | | | 0.037487 | | | 0.712082 | |  |  |  |  |  |  |
| genus.LachnospiraceaeUCG001.id.11321 | | rs79476906 | | T | A | | | -0.087088 | | | 0.019700842 | | 8.27E-06 | | 0.0023566 | | | 0.031785 | | | 0.940897 | |  |  |  |  |  |  |
| genus.LachnospiraceaeUCG001.id.11321 | | rs8104225 | | A | G | | | 0.0892098 | | | 0.019766483 | | 8.04E-06 | | 0.0367418 | | | 0.027743 | | | 0.185385 | |  |  |  |  |  |  |
| genus.LachnospiraceaeUCG001.id.11321 | | rs9403580 | | C | T | | | 0.1078005 | | | 0.022977722 | | 3.47E-06 | | 0.022781 | | | 0.033812 | | | 0.50047 | |  |  |  |  |  |  |
| genus.LachnospiraceaeUCG001.id.11321 | | rs985416 | | C | T | | | 0.0970258 | | | 0.018180694 | | 1.46E-07 | | 0.0226103 | | | 0.029731 | | | 0.446957 | |  |  |  |  |  |  |
| genus.LachnospiraceaeUCG004.id.11324 | | rs11128180 | | A | G | | | 0.0648288 | | | 0.014012621 | | 4.52E-06 | | 0.0266507 | | | 0.026988 | | | 0.323402 | |  |  |  |  |  |  |
| genus.LachnospiraceaeUCG004.id.11324 | | rs12072562 | | T | C | | | 0.1331037 | | | 0.030356565 | | 7.07E-06 | | 0.0266127 | | | 0.05891 | | | 0.65145 | |  |  |  |  |  |  |
| genus.LachnospiraceaeUCG004.id.11324 | | rs12673420 | | G | A | | | 0.0554351 | | | 0.011837515 | | 2.98E-06 | | -0.0022731 | | | 0.022824 | | | 0.920669 | |  |  |  |  |  |  |
| genus.LachnospiraceaeUCG004.id.11324 | | rs12747809 | | G | A | | | -0.062197 | | | 0.012568463 | | 8.65E-07 | | 0.0652651 | | | 0.025409 | | | 0.0102122 | |  |  |  |  |  |  |
| genus.LachnospiraceaeUCG004.id.11324 | | rs12894272 | | A | G | | | 0.0579792 | | | 0.012522898 | | 4.34E-06 | | -0.0158424 | | | 0.024125 | | | 0.511377 | |  |  |  |  |  |  |
| genus.LachnospiraceaeUCG004.id.11324 | | rs233486 | | A | G | | | -0.0799088 | | | 0.017765984 | | 6.28E-06 | | -0.0279338 | | | 0.032938 | | | 0.396392 | |  |  |  |  |  |  |
| genus.LachnospiraceaeUCG004.id.11324 | | rs2444793 | | C | T | | | -0.0542433 | | | 0.011818754 | | 4.77E-06 | | 0.0179244 | | | 0.023437 | | | 0.444391 | |  |  |  |  |  |  |
| genus.LachnospiraceaeUCG004.id.11324 | | rs2706242 | | G | C | | | -0.0901399 | | | 0.019971219 | | 9.84E-06 | | 0.0044867 | | | 0.040816 | | | 0.912469 | |  |  |  |  |  |  |
| genus.LachnospiraceaeUCG004.id.11324 | | rs2726805 | | A | G | | | 0.0548397 | | | 0.01208444 | | 6.30E-06 | | -0.0288408 | | | 0.023101 | | | 0.211854 | |  |  |  |  |  |  |
| genus.LachnospiraceaeUCG004.id.11324 | | rs2882478 | | G | A | | | -0.0577091 | | | 0.011833839 | | 1.21E-06 | | 0.0436549 | | | 0.022978 | | | 0.0574513 | |  |  |  |  |  |  |
| genus.LachnospiraceaeUCG004.id.11324 | | rs35182105 | | A | G | | | -0.1097243 | | | 0.024221006 | | 4.87E-06 | | -0.0569101 | | | 0.050552 | | | 0.260258 | |  |  |  |  |  |  |
| genus.LachnospiraceaeUCG004.id.11324 | | rs62256516 | | G | A | | | 0.0592366 | | | 0.013222215 | | 9.85E-06 | | 0.0221988 | | | 0.026612 | | | 0.404184 | |  |  |  |  |  |  |
| genus.LachnospiraceaeUCG004.id.11324 | | rs6656451 | | C | T | | | -0.05436 | | | 0.01194699 | | 5.57E-06 | | 0.0230772 | | | 0.022835 | | | 0.312213 | |  |  |  |  |  |  |
| genus.LachnospiraceaeUCG004.id.11324 | | rs7629954 | | A | G | | | 0.1084409 | | | 0.023824987 | | 5.77E-06 | | -0.0890345 | | | 0.055282 | | | 0.107276 | |  |  |  |  |  |  |
| genus.LachnospiraceaeUCG008.id.11328 | | rs10741777 | | T | C | | | -0.0973842 | | | 0.019479136 | | 7.69E-07 | | -0.0042707 | | | 0.024699 | | | 0.862724 | |  |  |  |  |  |  |
| genus.LachnospiraceaeUCG008.id.11328 | | rs10751237 | | A | C | | | -0.082075 | | | 0.017437723 | | 3.17E-06 | | 0.0056019 | | | 0.024803 | | | 0.821313 | |  |  |  |  |  |  |
| genus.LachnospiraceaeUCG008.id.11328 | | rs10793103 | | C | T | | | 0.0974344 | | | 0.018127922 | | 9.35E-08 | | -0.0165976 | | | 0.022956 | | | 0.469666 | |  |  |  |  |  |  |
| genus.LachnospiraceaeUCG008.id.11328 | | rs10801803 | | G | A | | | -0.1170291 | | | 0.024308454 | | 1.40E-06 | | -0.0336029 | | | 0.032581 | | | 0.302366 | |  |  |  |  |  |  |
| genus.LachnospiraceaeUCG008.id.11328 | | rs11236216 | | T | C | | | 0.083842 | | | 0.017670871 | | 2.35E-06 | | -0.0035816 | | | 0.023119 | | | 0.876884 | |  |  |  |  |  |  |
| genus.LachnospiraceaeUCG008.id.11328 | | rs13024781 | | T | C | | | -0.0798769 | | | 0.016884555 | | 2.29E-06 | | -0.0317134 | | | 0.02292 | | | 0.166466 | |  |  |  |  |  |  |
| genus.LachnospiraceaeUCG008.id.11328 | | rs57091572 | | A | G | | | -0.1104427 | | | 0.023580145 | | 2.86E-06 | | 0.0118399 | | | 0.033676 | | | 0.725147 | |  |  |  |  |  |  |
| genus.LachnospiraceaeUCG008.id.11328 | | rs61944774 | | A | G | | | 0.1798381 | | | 0.039379746 | | 6.34E-06 | | -0.0407736 | | | 0.051353 | | | 0.427199 | |  |  |  |  |  |  |
| genus.LachnospiraceaeUCG008.id.11328 | | rs62277846 | | C | T | | | 0.1022925 | | | 0.021231902 | | 1.59E-06 | | -0.0006334 | | | 0.029005 | | | 0.982578 | |  |  |  |  |  |  |
| genus.LachnospiraceaeUCG008.id.11328 | | rs67078837 | | T | C | | | -0.0845848 | | | 0.017069269 | | 7.68E-07 | | -0.0087094 | | | 0.023147 | | | 0.706719 | |  |  |  |  |  |  |
| genus.LachnospiraceaeUCG008.id.11328 | | rs75356640 | | G | A | | | 0.1365235 | | | 0.030312828 | | 9.83E-06 | | -0.0053231 | | | 0.0349 | | | 0.878773 | |  |  |  |  |  |  |
| genus.LachnospiraceaeUCG008.id.11328 | | rs955844 | | A | C | | | 0.1120642 | | | 0.022836934 | | 1.81E-06 | | 0.0117295 | | | 0.032927 | | | 0.721674 | |  |  |  |  |  |  |
| genus.LachnospiraceaeUCG008.id.11328 | | rs9873555 | | G | C | | | -0.1211618 | | | 0.023346742 | | 2.41E-07 | | -0.0027254 | | | 0.035629 | | | 0.939026 | |  |  |  |  |  |  |
| genus.LachnospiraceaeUCG010.id.11330 | | rs10414815 | | T | C | | | 0.1045296 | | | 0.023025257 | | 4.24E-06 | | 0.0312139 | | | 0.055235 | | | 0.571997 | |  |  |  |  |  |  |
| genus.LachnospiraceaeUCG010.id.11330 | | rs11192447 | | A | G | | | 0.1265921 | | | 0.024345002 | | 4.69E-07 | | -0.0499079 | | | 0.052336 | | | 0.340284 | |  |  |  |  |  |  |
| genus.LachnospiraceaeUCG010.id.11330 | | rs12346653 | | C | T | | | 0.0657686 | | | 0.013956927 | | 2.70E-06 | | 0.0154472 | | | 0.028097 | | | 0.582467 | |  |  |  |  |  |  |
| genus.LachnospiraceaeUCG010.id.11330 | | rs17730011 | | G | A | | | -0.0702357 | | | 0.015706022 | | 7.85E-06 | | 0.0260752 | | | 0.027947 | | | 0.350813 | |  |  |  |  |  |  |
| genus.LachnospiraceaeUCG010.id.11330 | | rs2153460 | | A | T | | | -0.0684038 | | | 0.015656159 | | 9.17E-06 | | -0.0075506 | | | 0.030726 | | | 0.805882 | |  |  |  |  |  |  |
| genus.LachnospiraceaeUCG010.id.11330 | | rs2833528 | | C | T | | | -0.0562148 | | | 0.012776299 | | 9.92E-06 | | -0.0212983 | | | 0.023679 | | | 0.368413 | |  |  |  |  |  |  |
| genus.LachnospiraceaeUCG010.id.11330 | | rs336138 | | G | T | | | 0.0779543 | | | 0.017186539 | | 7.48E-06 | | -0.0429841 | | | 0.0357 | | | 0.228578 | |  |  |  |  |  |  |
| genus.LachnospiraceaeUCG010.id.11330 | | rs4576377 | | A | C | | | -0.0572001 | | | 0.012704307 | | 7.63E-06 | | 0.0104809 | | | 0.023707 | | | 0.658411 | |  |  |  |  |  |  |
| genus.LachnospiraceaeUCG010.id.11330 | | rs72761829 | | A | T | | | 0.1118836 | | | 0.023889286 | | 2.58E-06 | | 0.044556 | | | 0.040283 | | | 0.2687 | |  |  |  |  |  |  |
| genus.LachnospiraceaeUCG010.id.11330 | | rs72894957 | | G | A | | | 0.222263 | | | 0.048642511 | | 5.68E-06 | | -0.11217 | | | 0.07654 | | | 0.142781 | |  |  |  |  |  |  |
| genus.LachnospiraceaeUCG010.id.11330 | | rs74315802 | | G | T | | | 0.0867262 | | | 0.018347717 | | 3.19E-06 | | -0.0092432 | | | 0.029748 | | | 0.756014 | |  |  |  |  |  |  |
| genus.LachnospiraceaeUCG010.id.11330 | | rs9981767 | | A | C | | | 0.0655062 | | | 0.01319942 | | 9.96E-07 | | -0.0424114 | | | 0.02629 | | | 0.106699 | |  |  |  |  |  |  |
| genus.Lactobacillus.id.1837 | | rs12693845 | | C | T | | | -0.0805447 | | | 0.017742876 | | 8.96E-06 | | 0.022916 | | | 0.023551 | | | 0.330531 | |  |  |  |  |  |  |
| genus.Lactobacillus.id.1837 | | rs1530559 | | G | A | | | 0.0804001 | | | 0.017820739 | | 4.93E-06 | | 0.0073463 | | | 0.023054 | | | 0.749987 | |  |  |  |  |  |  |
| genus.Lactobacillus.id.1837 | | rs16861661 | | G | A | | | -0.183147 | | | 0.038148013 | | 1.28E-06 | | -0.0230186 | | | 0.046429 | | | 0.62005 | |  |  |  |  |  |  |
| genus.Lactobacillus.id.1837 | | rs328312 | | T | A | | | 0.0815092 | | | 0.016944121 | | 1.41E-06 | | -0.0008074 | | | 0.022892 | | | 0.971863 | |  |  |  |  |  |  |
| genus.Lactobacillus.id.1837 | | rs6092149 | | A | T | | | -0.0801344 | | | 0.01714896 | | 3.29E-06 | | -0.0003525 | | | 0.023135 | | | 0.987845 | |  |  |  |  |  |  |
| genus.Lactobacillus.id.1837 | | rs62314653 | | C | A | | | 0.1876919 | | | 0.039458456 | | 2.24E-06 | | 0.0523748 | | | 0.048457 | | | 0.279762 | |  |  |  |  |  |  |
| genus.Lactobacillus.id.1837 | | rs7399658 | | G | A | | | -0.1071337 | | | 0.022188263 | | 3.12E-06 | | 0.0175721 | | | 0.029805 | | | 0.555472 | |  |  |  |  |  |  |
| genus.Lactobacillus.id.1837 | | rs768253 | | T | G | | | -0.079195 | | | 0.017179101 | | 4.25E-06 | | -0.0185045 | | | 0.022938 | | | 0.419825 | |  |  |  |  |  |  |
| genus.Lactobacillus.id.1837 | | rs77478751 | | A | G | | | -0.2198858 | | | 0.047575911 | | 7.33E-06 | | -0.0043283 | | | 0.035588 | | | 0.903198 | |  |  |  |  |  |  |
| genus.Lactobacillus.id.1837 | | rs921925 | | A | C | | | 0.0985077 | | | 0.020322897 | | 9.72E-07 | | -0.0395908 | | | 0.027917 | | | 0.156149 | |  |  |  |  |  |  |
| genus.Lactococcus.id.1851 | | rs10417872 | | T | G | | | 0.1183058 | | | 0.024521975 | | 1.29E-06 | | 0.0389511 | | | 0.025256 | | | 0.123005 | |  |  |  |  |  |  |
| genus.Lactococcus.id.1851 | | rs123059 | | T | C | | | -0.1367098 | | | 0.02746936 | | 1.27E-06 | | 0.0074803 | | | 0.027693 | | | 0.787071 | |  |  |  |  |  |  |
| genus.Lactococcus.id.1851 | | rs12621813 | | G | A | | | 0.1084228 | | | 0.023997845 | | 6.61E-06 | | 0.0253642 | | | 0.025943 | | | 0.328224 | |  |  |  |  |  |  |
| genus.Lactococcus.id.1851 | | rs17168302 | | G | A | | | 0.1918565 | | | 0.042476093 | | 6.29E-06 | | -0.0324648 | | | 0.037076 | | | 0.381233 | |  |  |  |  |  |  |
| genus.Lactococcus.id.1851 | | rs2293361 | | C | T | | | -0.1992211 | | | 0.043096892 | | 1.40E-06 | | -0.0203532 | | | 0.050873 | | | 0.6891 | |  |  |  |  |  |  |
| genus.Lactococcus.id.1851 | | rs34757988 | | G | C | | | 0.1222939 | | | 0.022897605 | | 8.95E-08 | | -0.0240873 | | | 0.023464 | | | 0.304623 | |  |  |  |  |  |  |
| genus.Lactococcus.id.1851 | | rs4766997 | | C | T | | | 0.1145992 | | | 0.023839024 | | 2.06E-06 | | 0.0372665 | | | 0.023062 | | | 0.106107 | |  |  |  |  |  |  |
| genus.Lactococcus.id.1851 | | rs55910161 | | C | T | | | 0.1464259 | | | 0.030736694 | | 2.36E-06 | | 0.0430263 | | | 0.036328 | | | 0.236254 | |  |  |  |  |  |  |
| genus.Lactococcus.id.1851 | | rs6674304 | | C | T | | | 0.2007577 | | | 0.044212138 | | 6.18E-06 | | 0.0203364 | | | 0.059398 | | | 0.732069 | |  |  |  |  |  |  |
| genus.Lactococcus.id.1851 | | rs757872 | | G | C | | | 0.1407991 | | | 0.027581978 | | 4.37E-07 | | -0.0018256 | | | 0.030588 | | | 0.952407 | |  |  |  |  |  |  |
| genus.Marvinbryantia.id.2005 | | rs11620597 | | T | C | | | 0.1194793 | | | 0.027168773 | | 7.80E-06 | | 0.0236314 | | | 0.074054 | | | 0.749642 | |  |  |  |  |  |  |
| genus.Marvinbryantia.id.2005 | | rs11645029 | | G | C | | | -0.060618 | | | 0.013155125 | | 4.15E-06 | | -0.0319113 | | | 0.022888 | | | 0.163239 | |  |  |  |  |  |  |
| genus.Marvinbryantia.id.2005 | | rs1187983 | | C | T | | | -0.0935456 | | | 0.019317443 | | 2.02E-06 | | -0.012474 | | | 0.037438 | | | 0.738992 | |  |  |  |  |  |  |
| genus.Marvinbryantia.id.2005 | | rs146541147 | | G | A | | | 0.1188454 | | | 0.02684231 | | 6.86E-06 | | -0.026647 | | | 0.064086 | | | 0.677556 | |  |  |  |  |  |  |
| genus.Marvinbryantia.id.2005 | | rs2724813 | | A | G | | | -0.0840769 | | | 0.01675508 | | 6.28E-07 | | -0.0373665 | | | 0.026873 | | | 0.164377 | |  |  |  |  |  |  |
| genus.Marvinbryantia.id.2005 | | rs2842896 | | C | T | | | -0.0649396 | | | 0.013114646 | | 7.25E-07 | | -0.0226574 | | | 0.023296 | | | 0.330758 | |  |  |  |  |  |  |
| genus.Marvinbryantia.id.2005 | | rs2863363 | | A | G | | | 0.0634861 | | | 0.013632279 | | 3.11E-06 | | -0.0016356 | | | 0.026545 | | | 0.950867 | |  |  |  |  |  |  |
| genus.Marvinbryantia.id.2005 | | rs3125832 | | A | C | | | 0.0679323 | | | 0.015011987 | | 5.03E-06 | | 0.0596116 | | | 0.027355 | | | 0.0293184 | |  |  |  |  |  |  |
| genus.Marvinbryantia.id.2005 | | rs3991048 | | G | T | | | 0.0591462 | | | 0.013253586 | | 7.09E-06 | | 0.0696172 | | | 0.023626 | | | 0.00321299 | |  |  |  |  |  |  |
| genus.Marvinbryantia.id.2005 | | rs61884471 | | G | A | | | 0.1244263 | | | 0.024843116 | | 1.01E-06 | | 0.0581165 | | | 0.036546 | | | 0.111782 | |  |  |  |  |  |  |
| genus.Marvinbryantia.id.2005 | | rs72948274 | | A | C | | | -0.126354 | | | 0.02722118 | | 3.26E-06 | | 0.0672248 | | | 0.046692 | | | 0.149939 | |  |  |  |  |  |  |
| genus.Marvinbryantia.id.2005 | | rs8006832 | | G | T | | | -0.0952415 | | | 0.021669867 | | 6.58E-06 | | -0.0302656 | | | 0.039557 | | | 0.444201 | |  |  |  |  |  |  |
| genus.Methanobrevibacter.id.123 | | rs10202904 | | T | G | | | -0.1128111 | | | 0.023910624 | | 3.09E-06 | | 0.0090214 | | | 0.023319 | | | 0.698848 | |  |  |  |  |  |  |
| genus.Methanobrevibacter.id.123 | | rs11018665 | | A | T | | | 0.113013 | | | 0.025434251 | | 7.03E-06 | | -0.0440645 | | | 0.026723 | | | 0.0991631 | |  |  |  |  |  |  |
| genus.Methanobrevibacter.id.123 | | rs1334944 | | T | C | | | 0.1151965 | | | 0.025549236 | | 7.61E-06 | | 0.0072183 | | | 0.025399 | | | 0.776256 | |  |  |  |  |  |  |
| genus.Methanobrevibacter.id.123 | | rs4779844 | | G | C | | | 0.109639 | | | 0.024797947 | | 9.28E-06 | | 0.0690305 | | | 0.023796 | | | 0.00372015 | |  |  |  |  |  |  |
| genus.Methanobrevibacter.id.123 | | rs4802933 | | A | G | | | -0.1356284 | | | 0.030813872 | | 9.74E-06 | | -0.0323178 | | | 0.027093 | | | 0.23293 | |  |  |  |  |  |  |
| genus.Methanobrevibacter.id.123 | | rs6776814 | | T | C | | | -0.1889563 | | | 0.041990543 | | 8.05E-06 | | 0.0046405 | | | 0.081581 | | | 0.954639 | |  |  |  |  |  |  |
| genus.Methanobrevibacter.id.123 | | rs76029318 | | T | C | | | 0.2228489 | | | 0.045431939 | | 1.08E-06 | | 0.0856394 | | | 0.047118 | | | 0.0691353 | |  |  |  |  |  |  |
| genus.Methanobrevibacter.id.123 | | rs894996 | | C | A | | | 0.2142126 | | | 0.045604463 | | 3.82E-06 | | -0.0173694 | | | 0.044584 | | | 0.696841 | |  |  |  |  |  |  |
| genus.Odoribacter.id.952 | | rs10093869 | | A | G | | | -0.0577795 | | | 0.012538929 | | 3.67E-06 | | -0.0191078 | | | 0.023113 | | | 0.408403 | |  |  |  |  |  |  |
| genus.Odoribacter.id.952 | | rs10423795 | | C | T | | | 0.0550695 | | | 0.012116411 | | 6.58E-06 | | 0.0059217 | | | 0.023553 | | | 0.801489 | |  |  |  |  |  |  |
| genus.Odoribacter.id.952 | | rs16918425 | | A | T | | | 0.0999781 | | | 0.022378451 | | 8.85E-06 | | -0.0256758 | | | 0.053945 | | | 0.6341 | |  |  |  |  |  |  |
| genus.Odoribacter.id.952 | | rs28417404 | | A | G | | | -0.0726896 | | | 0.016137195 | | 3.68E-06 | | 0.0860005 | | | 0.038575 | | | 0.0257858 | |  |  |  |  |  |  |
| genus.Odoribacter.id.952 | | rs4793970 | | A | G | | | -0.0576329 | | | 0.012915412 | | 6.03E-06 | | 0.0446156 | | | 0.023815 | | | 0.0610085 | |  |  |  |  |  |  |
| genus.Odoribacter.id.952 | | rs503751 | | C | G | | | 0.0618294 | | | 0.011888659 | | 2.08E-07 | | -0.0158689 | | | 0.022846 | | | 0.487306 | |  |  |  |  |  |  |
| genus.Odoribacter.id.952 | | rs6856150 | | G | A | | | 0.0881922 | | | 0.019414623 | | 6.06E-06 | | 0.016476 | | | 0.034736 | | | 0.635269 | |  |  |  |  |  |  |
| genus.Odoribacter.id.952 | | rs74553962 | | T | G | | | 0.1214488 | | | 0.026410563 | | 9.49E-06 | | -0.0297723 | | | 0.043399 | | | 0.492706 | |  |  |  |  |  |  |
| genus.Odoribacter.id.952 | | rs77779484 | | G | A | | | -0.1334894 | | | 0.026852607 | | 6.56E-07 | | -0.0730171 | | | 0.047965 | | | 0.127938 | |  |  |  |  |  |  |
| genus.Olsenella.id.822 | | rs1035588 | | A | G | | | -0.1081481 | | | 0.023684753 | | 4.86E-06 | | -0.0260598 | | | 0.023688 | | | 0.271282 | |  |  |  |  |  |  |
| genus.Olsenella.id.822 | | rs17148768 | | G | A | | | 0.1404342 | | | 0.029560175 | | 2.20E-06 | | 0.0041218 | | | 0.030741 | | | 0.893339 | |  |  |  |  |  |  |
| genus.Olsenella.id.822 | | rs2759329 | | G | A | | | -0.1111314 | | | 0.02372184 | | 3.43E-06 | | 0.0015036 | | | 0.023657 | | | 0.94932 | |  |  |  |  |  |  |
| genus.Olsenella.id.822 | | rs35225860 | | A | G | | | -0.2236037 | | | 0.048239131 | | 3.87E-06 | | 0.131641 | | | 0.058333 | | | 0.0240259 | |  |  |  |  |  |  |
| genus.Olsenella.id.822 | | rs61090148 | | A | G | | | -0.1047827 | | | 0.023134258 | | 6.44E-06 | | -0.0046919 | | | 0.023135 | | | 0.839283 | |  |  |  |  |  |  |
| genus.Olsenella.id.822 | | rs62112538 | | C | T | | | -0.1994303 | | | 0.040703259 | | 1.19E-06 | | 0.0132891 | | | 0.036898 | | | 0.718729 | |  |  |  |  |  |  |
| genus.Olsenella.id.822 | | rs72691585 | | C | A | | | -0.2490789 | | | 0.052081416 | | 2.95E-06 | | 0.0195073 | | | 0.033521 | | | 0.560604 | |  |  |  |  |  |  |
| genus.Olsenella.id.822 | | rs7540303 | | C | T | | | 0.1080438 | | | 0.023637952 | | 5.32E-06 | | 0.0042039 | | | 0.023622 | | | 0.85875 | |  |  |  |  |  |  |
| genus.Olsenella.id.822 | | rs8066522 | | G | A | | | -0.1065287 | | | 0.02403789 | | 9.70E-06 | | 0.010753 | | | 0.024362 | | | 0.658928 | |  |  |  |  |  |  |
| genus.Olsenella.id.822 | | rs9460691 | | C | A | | | 0.1199658 | | | 0.026864494 | | 7.28E-06 | | 0.0064691 | | | 0.029132 | | | 0.824262 | |  |  |  |  |  |  |
| genus.Oscillibacter.id.2063 | | rs11627628 | | T | C | | | 0.1439607 | | | 0.029022314 | | 1.01E-06 | | -0.0605526 | | | 0.043233 | | | 0.161333 | |  |  |  |  |  |  |
| genus.Oscillibacter.id.2063 | | rs11990279 | | T | C | | | -0.0824934 | | | 0.018045748 | | 4.94E-06 | | 0.0039427 | | | 0.028512 | | | 0.890016 | |  |  |  |  |  |  |
| genus.Oscillibacter.id.2063 | | rs12417956 | | C | G | | | 0.0785431 | | | 0.017407454 | | 6.03E-06 | | -0.044904 | | | 0.025482 | | | 0.0780369 | |  |  |  |  |  |  |
| genus.Oscillibacter.id.2063 | | rs12649930 | | T | G | | | 0.1215887 | | | 0.025961173 | | 4.09E-06 | | 0.0150807 | | | 0.037787 | | | 0.689822 | |  |  |  |  |  |  |
| genus.Oscillibacter.id.2063 | | rs133832 | | A | C | | | -0.0795527 | | | 0.016241059 | | 1.15E-06 | | -0.0136485 | | | 0.025426 | | | 0.591412 | |  |  |  |  |  |  |
| genus.Oscillibacter.id.2063 | | rs137917150 | | T | A | | | -0.1749922 | | | 0.038839403 | | 4.62E-06 | | -0.0159612 | | | 0.03997 | | | 0.689652 | |  |  |  |  |  |  |
| genus.Oscillibacter.id.2063 | | rs16866406 | | A | G | | | 0.0988765 | | | 0.02087954 | | 3.08E-06 | | 0.0016755 | | | 0.03165 | | | 0.95778 | |  |  |  |  |  |  |
| genus.Oscillibacter.id.2063 | | rs16934185 | | A | G | | | -0.1295675 | | | 0.028156999 | | 4.38E-06 | | 0.019173 | | | 0.037534 | | | 0.609481 | |  |  |  |  |  |  |
| genus.Oscillibacter.id.2063 | | rs234108 | | A | G | | | 0.0749553 | | | 0.015263201 | | 9.16E-07 | | -0.0062566 | | | 0.023251 | | | 0.787865 | |  |  |  |  |  |  |
| genus.Oscillibacter.id.2063 | | rs36095275 | | C | T | | | -0.0752368 | | | 0.015686113 | | 1.40E-06 | | 0.0172232 | | | 0.023294 | | | 0.459677 | |  |  |  |  |  |  |
| genus.Oscillibacter.id.2063 | | rs4506202 | | A | G | | | -0.0711323 | | | 0.01522611 | | 3.21E-06 | | 0.0232907 | | | 0.02294 | | | 0.30996 | |  |  |  |  |  |  |
| genus.Oscillibacter.id.2063 | | rs61883564 | | A | G | | | -0.1013514 | | | 0.022101565 | | 3.39E-06 | | 0.066058 | | | 0.033192 | | | 0.0465715 | |  |  |  |  |  |  |
| genus.Oscillibacter.id.2063 | | rs6901560 | | C | G | | | 0.0855032 | | | 0.018693038 | | 6.21E-06 | | 0.0169216 | | | 0.029342 | | | 0.564145 | |  |  |  |  |  |  |
| genus.Oscillibacter.id.2063 | | rs75453768 | | G | T | | | 0.1221201 | | | 0.026862946 | | 5.35E-06 | | 0.0086738 | | | 0.038566 | | | 0.822051 | |  |  |  |  |  |  |
| genus.Oscillibacter.id.2063 | | rs761240 | | T | G | | | -0.1766396 | | | 0.038881196 | | 2.04E-06 | | -0.0613974 | | | 0.053877 | | | 0.254458 | |  |  |  |  |  |  |
| genus.Oscillibacter.id.2063 | | rs9393920 | | A | G | | | -0.0744662 | | | 0.015108116 | | 9.92E-07 | | -0.0197328 | | | 0.023454 | | | 0.400149 | |  |  |  |  |  |  |
| genus.Oscillospira.id.2064 | | rs12206468 | | G | A | | | -0.1330158 | | | 0.026973335 | | 1.04E-06 | | -0.0586557 | | | 0.042991 | | | 0.17245 | |  |  |  |  |  |  |
| genus.Oscillospira.id.2064 | | rs12925026 | | T | C | | | 0.1355901 | | | 0.030677984 | | 9.31E-06 | | 0.0239953 | | | 0.049548 | | | 0.628181 | |  |  |  |  |  |  |
| genus.Oscillospira.id.2064 | | rs1954532 | | T | C | | | -0.0826246 | | | 0.017525166 | | 2.27E-06 | | -0.0156067 | | | 0.028662 | | | 0.586095 | |  |  |  |  |  |  |
| genus.Oscillospira.id.2064 | | rs2211563 | | C | A | | | 0.0819177 | | | 0.018107866 | | 9.12E-06 | | 0.0010814 | | | 0.025261 | | | 0.965854 | |  |  |  |  |  |  |
| genus.Oscillospira.id.2064 | | rs28889936 | | A | C | | | 0.1140509 | | | 0.025283847 | | 3.37E-06 | | -0.0416346 | | | 0.038691 | | | 0.281894 | |  |  |  |  |  |  |
| genus.Oscillospira.id.2064 | | rs62422654 | | C | T | | | 0.0898456 | | | 0.019808815 | | 6.47E-06 | | 0.0015782 | | | 0.028093 | | | 0.955201 | |  |  |  |  |  |  |
| genus.Oscillospira.id.2064 | | rs72866977 | | A | C | | | -0.130569 | | | 0.028167414 | | 5.63E-06 | | 0.0816326 | | | 0.04268 | | | 0.0557918 | |  |  |  |  |  |  |
| genus.Oscillospira.id.2064 | | rs73038677 | | T | A | | | -0.08321 | | | 0.016866557 | | 1.09E-06 | | 0.0252923 | | | 0.033181 | | | 0.445904 | |  |  |  |  |  |  |
| genus.Oscillospira.id.2064 | | rs751183 | | T | C | | | -0.0774322 | | | 0.017221553 | | 6.85E-06 | | 0.0031468 | | | 0.029529 | | | 0.915133 | |  |  |  |  |  |  |
| genus.Oscillospira.id.2064 | | rs8076323 | | A | G | | | 0.0715392 | | | 0.015653872 | | 5.61E-06 | | -0.0574457 | | | 0.024305 | | | 0.0181038 | |  |  |  |  |  |  |
| genus.Oxalobacter.id.2978 | | rs10464997 | | G | A | | | 0.1376909 | | | 0.029480352 | | 3.30E-06 | | 0.0347721 | | | 0.029374 | | | 0.236496 | |  |  |  |  |  |  |
| genus.Oxalobacter.id.2978 | | rs11108500 | | A | G | | | -0.1990991 | | | 0.04273266 | | 3.74E-06 | | -0.0394182 | | | 0.04076 | | | 0.333499 | |  |  |  |  |  |  |
| genus.Oxalobacter.id.2978 | | rs111966731 | | T | C | | | 0.2131145 | | | 0.047162014 | | 7.30E-06 | | 0.0316009 | | | 0.040836 | | | 0.43902 | |  |  |  |  |  |  |
| genus.Oxalobacter.id.2978 | | rs12002250 | | A | C | | | 0.2171224 | | | 0.046631729 | | 1.42E-06 | | 0.0390839 | | | 0.056838 | | | 0.491678 | |  |  |  |  |  |  |
| genus.Oxalobacter.id.2978 | | rs1569853 | | T | C | | | -0.1380775 | | | 0.029698126 | | 3.65E-06 | | 0.0199521 | | | 0.035032 | | | 0.568993 | |  |  |  |  |  |  |
| genus.Oxalobacter.id.2978 | | rs36057338 | | G | T | | | 0.207847 | | | 0.042143922 | | 8.80E-07 | | -0.0831055 | | | 0.063965 | | | 0.193867 | |  |  |  |  |  |  |
| genus.Oxalobacter.id.2978 | | rs3862635 | | C | T | | | -0.172142 | | | 0.039402609 | | 9.19E-06 | | -0.0576237 | | | 0.039749 | | | 0.147146 | |  |  |  |  |  |  |
| genus.Oxalobacter.id.2978 | | rs4428215 | | G | A | | | 0.1302926 | | | 0.0242237 | | 7.51E-08 | | -0.000186 | | | 0.026221 | | | 0.994339 | |  |  |  |  |  |  |
| genus.Oxalobacter.id.2978 | | rs6000536 | | C | T | | | -0.1309917 | | | 0.025380356 | | 2.06E-07 | | 0.0242384 | | | 0.030641 | | | 0.428913 | |  |  |  |  |  |  |
| genus.Oxalobacter.id.2978 | | rs6071435 | | T | A | | | -0.1055123 | | | 0.021488964 | | 1.07E-06 | | -0.0011039 | | | 0.023493 | | | 0.962523 | |  |  |  |  |  |  |
| genus.Oxalobacter.id.2978 | | rs6993398 | | G | A | | | 0.1272171 | | | 0.027885461 | | 7.13E-06 | | 0.0094465 | | | 0.029428 | | | 0.748204 | |  |  |  |  |  |  |
| genus.Oxalobacter.id.2978 | | rs736744 | | C | T | | | 0.1178821 | | | 0.021126239 | | 2.57E-08 | | 0.0184775 | | | 0.023013 | | | 0.422025 | |  |  |  |  |  |  |
| genus.Parabacteroides.id.954 | | rs115602804 | | G | A | | | 0.1030801 | | | 0.02227358 | | 1.93E-06 | | -0.0493017 | | | 0.037165 | | | 0.184649 | |  |  |  |  |  |  |
| genus.Parabacteroides.id.954 | | rs11965579 | | G | C | | | 0.1628239 | | | 0.038186321 | | 8.87E-06 | | -0.0644342 | | | 0.078693 | | | 0.412894 | |  |  |  |  |  |  |
| genus.Parabacteroides.id.954 | | rs3860755 | | G | C | | | 0.0558784 | | | 0.011662454 | | 1.71E-06 | | 0.0253959 | | | 0.024513 | | | 0.300195 | |  |  |  |  |  |  |
| genus.Parabacteroides.id.954 | | rs4236095 | | G | A | | | 0.0761961 | | | 0.015704151 | | 1.93E-06 | | -0.0400514 | | | 0.037846 | | | 0.289931 | |  |  |  |  |  |  |
| genus.Parabacteroides.id.954 | | rs60884758 | | C | T | | | -0.0702667 | | | 0.014224884 | | 5.71E-07 | | 0.0020609 | | | 0.029861 | | | 0.944977 | |  |  |  |  |  |  |
| genus.Parabacteroides.id.954 | | rs6657302 | | T | C | | | -0.1045197 | | | 0.022552116 | | 9.76E-06 | | 0.0140054 | | | 0.046967 | | | 0.765551 | |  |  |  |  |  |  |
| genus.Parabacteroides.id.954 | | rs72893646 | | A | T | | | -0.0715594 | | | 0.015666777 | | 8.83E-06 | | 0.0355494 | | | 0.034027 | | | 0.296137 | |  |  |  |  |  |  |
| genus.Parabacteroides.id.954 | | rs7298818 | | C | T | | | 0.0888832 | | | 0.02009019 | | 8.54E-06 | | -0.0536956 | | | 0.037566 | | | 0.152901 | |  |  |  |  |  |  |
| genus.Paraprevotella.id.962 | | rs10842464 | | T | C | | | -0.0758215 | | | 0.017256778 | | 6.60E-06 | | 0.019254 | | | 0.024931 | | | 0.439935 | |  |  |  |  |  |  |
| genus.Paraprevotella.id.962 | | rs140997932 | | T | C | | | -0.1623756 | | | 0.035417814 | | 2.11E-06 | | -0.0126707 | | | 0.049843 | | | 0.799331 | |  |  |  |  |  |  |
| genus.Paraprevotella.id.962 | | rs145020347 | | A | G | | | -0.12465 | | | 0.026232336 | | 4.03E-06 | | -0.0451426 | | | 0.032357 | | | 0.162971 | |  |  |  |  |  |  |
| genus.Paraprevotella.id.962 | | rs17109926 | | A | G | | | -0.0988335 | | | 0.021617178 | | 6.75E-06 | | -0.015131 | | | 0.025682 | | | 0.555755 | |  |  |  |  |  |  |
| genus.Paraprevotella.id.962 | | rs17785622 | | A | G | | | 0.2480654 | | | 0.052430396 | | 1.93E-06 | | -0.0198193 | | | 0.059415 | | | 0.7387 | |  |  |  |  |  |  |
| genus.Paraprevotella.id.962 | | rs2081023 | | A | G | | | -0.1225607 | | | 0.023650724 | | 2.64E-07 | | 0.0314385 | | | 0.032901 | | | 0.339306 | |  |  |  |  |  |  |
| genus.Paraprevotella.id.962 | | rs3008582 | | T | C | | | 0.1057201 | | | 0.022724571 | | 4.36E-06 | | -0.0030407 | | | 0.028996 | | | 0.916484 | |  |  |  |  |  |  |
| genus.Paraprevotella.id.962 | | rs3801748 | | G | A | | | 0.0779721 | | | 0.01716912 | | 5.20E-06 | | 0.0184296 | | | 0.023774 | | | 0.438227 | |  |  |  |  |  |  |
| genus.Paraprevotella.id.962 | | rs4756632 | | G | T | | | -0.1389055 | | | 0.028989683 | | 3.82E-06 | | 0.0439473 | | | 0.033806 | | | 0.193604 | |  |  |  |  |  |  |
| genus.Paraprevotella.id.962 | | rs4767113 | | C | T | | | 0.0882466 | | | 0.018381962 | | 2.14E-06 | | 0.0063608 | | | 0.024406 | | | 0.794379 | |  |  |  |  |  |  |
| genus.Paraprevotella.id.962 | | rs7240324 | | T | G | | | -0.1022904 | | | 0.022694788 | | 5.96E-06 | | -0.0485112 | | | 0.026517 | | | 0.0673302 | |  |  |  |  |  |  |
| genus.Paraprevotella.id.962 | | rs9602779 | | A | C | | | -0.1066918 | | | 0.02202635 | | 6.93E-07 | | -0.0270352 | | | 0.026562 | | | 0.308771 | |  |  |  |  |  |  |
| genus.Paraprevotella.id.962 | | rs9900242 | | A | G | | | -0.0852967 | | | 0.017521167 | | 1.14E-06 | | -0.0166366 | | | 0.02403 | | | 0.488732 | |  |  |  |  |  |  |
| genus.Parasutterella.id.2892 | | rs10899911 | | A | G | | | -0.0717102 | | | 0.014815144 | | 1.15E-06 | | -0.0234829 | | | 0.026912 | | | 0.382886 | |  |  |  |  |  |  |
| genus.Parasutterella.id.2892 | | rs11715853 | | G | A | | | -0.0662952 | | | 0.014611398 | | 6.23E-06 | | -0.0178196 | | | 0.025049 | | | 0.47684 | |  |  |  |  |  |  |
| genus.Parasutterella.id.2892 | | rs1403396 | | A | T | | | -0.0756729 | | | 0.015903462 | | 2.76E-06 | | 0.0208702 | | | 0.027916 | | | 0.454699 | |  |  |  |  |  |  |
| genus.Parasutterella.id.2892 | | rs2090816 | | A | C | | | 0.0840967 | | | 0.017731494 | | 2.90E-06 | | -0.0271762 | | | 0.029691 | | | 0.360035 | |  |  |  |  |  |  |
| genus.Parasutterella.id.2892 | | rs35055552 | | T | C | | | 0.1095542 | | | 0.023543268 | | 3.35E-06 | | 0.0074911 | | | 0.033168 | | | 0.821317 | |  |  |  |  |  |  |
| genus.Parasutterella.id.2892 | | rs35414597 | | T | A | | | -0.0684946 | | | 0.01421433 | | 1.51E-06 | | -0.0237723 | | | 0.025 | | | 0.341655 | |  |  |  |  |  |  |
| genus.Parasutterella.id.2892 | | rs55877868 | | A | C | | | -0.1044578 | | | 0.022808932 | | 2.87E-06 | | 0.0004224 | | | 0.037913 | | | 0.99111 | |  |  |  |  |  |  |
| genus.Parasutterella.id.2892 | | rs62273907 | | A | G | | | 0.2294681 | | | 0.050226185 | | 5.88E-06 | | -0.0249622 | | | 0.045597 | | | 0.584067 | |  |  |  |  |  |  |
| genus.Parasutterella.id.2892 | | rs6809952 | | G | A | | | -0.0684957 | | | 0.015089156 | | 8.13E-06 | | 0.0093493 | | | 0.025957 | | | 0.718711 | |  |  |  |  |  |  |
| genus.Parasutterella.id.2892 | | rs6828768 | | C | T | | | 0.0636852 | | | 0.013264516 | | 1.78E-06 | | -0.0309181 | | | 0.022859 | | | 0.176204 | |  |  |  |  |  |  |
| genus.Parasutterella.id.2892 | | rs7303158 | | C | T | | | 0.0646859 | | | 0.013425592 | | 1.33E-06 | | 0.0181873 | | | 0.022984 | | | 0.428763 | |  |  |  |  |  |  |
| genus.Parasutterella.id.2892 | | rs7311004 | | T | C | | | -0.0617522 | | | 0.01364384 | | 5.92E-06 | | -0.0073879 | | | 0.022985 | | | 0.747885 | |  |  |  |  |  |  |
| genus.Parasutterella.id.2892 | | rs7572229 | | G | A | | | 0.066273 | | | 0.013273581 | | 6.32E-07 | | 0.0012373 | | | 0.022903 | | | 0.956915 | |  |  |  |  |  |  |
| genus.Parasutterella.id.2892 | | rs78383039 | | T | C | | | -0.1463146 | | | 0.029711928 | | 1.57E-06 | | 0.0119964 | | | 0.058141 | | | 0.836531 | |  |  |  |  |  |  |
| genus.Parasutterella.id.2892 | | rs8039785 | | T | G | | | 0.0618345 | | | 0.013300041 | | 3.62E-06 | | 0.0306675 | | | 0.022838 | | | 0.179329 | |  |  |  |  |  |  |
| genus.Parasutterella.id.2892 | | rs823424 | | G | A | | | -0.0713448 | | | 0.015695821 | | 4.95E-06 | | -0.0295059 | | | 0.026213 | | | 0.260329 | |  |  |  |  |  |  |
| genus.Peptococcus.id.2037 | | rs10031059 | | T | C | | | -0.1211665 | | | 0.022584403 | | 1.24E-07 | | -0.0046579 | | | 0.02684 | | | 0.862228 | |  |  |  |  |  |  |
| genus.Peptococcus.id.2037 | | rs11001941 | | G | A | | | -0.1956111 | | | 0.039221681 | | 1.33E-06 | | 0.039074 | | | 0.040505 | | | 0.334714 | |  |  |  |  |  |  |
| genus.Peptococcus.id.2037 | | rs11030569 | | A | T | | | -0.1740201 | | | 0.037413336 | | 3.13E-06 | | -0.0150279 | | | 0.034439 | | | 0.662572 | |  |  |  |  |  |  |
| genus.Peptococcus.id.2037 | | rs12069354 | | C | T | | | 0.1676298 | | | 0.037950049 | | 9.28E-06 | | -0.0261094 | | | 0.047224 | | | 0.580345 | |  |  |  |  |  |  |
| genus.Peptococcus.id.2037 | | rs2054133 | | G | A | | | 0.0895433 | | | 0.018833153 | | 2.14E-06 | | -0.0032331 | | | 0.024026 | | | 0.892954 | |  |  |  |  |  |  |
| genus.Peptococcus.id.2037 | | rs34282744 | | G | C | | | 0.1917974 | | | 0.03998324 | | 1.84E-06 | | -0.0245546 | | | 0.041906 | | | 0.557907 | |  |  |  |  |  |  |
| genus.Peptococcus.id.2037 | | rs36121075 | | A | G | | | -0.1406701 | | | 0.030627993 | | 6.99E-06 | | 0.0280928 | | | 0.030553 | | | 0.357841 | |  |  |  |  |  |  |
| genus.Peptococcus.id.2037 | | rs413827 | | G | A | | | 0.1102295 | | | 0.023752348 | | 3.30E-06 | | -0.0410458 | | | 0.02669 | | | 0.124082 | |  |  |  |  |  |  |
| genus.Peptococcus.id.2037 | | rs5770862 | | T | C | | | 0.1620178 | | | 0.035681339 | | 3.22E-06 | | 0.0155643 | | | 0.039327 | | | 0.692272 | |  |  |  |  |  |  |
| genus.Peptococcus.id.2037 | | rs6918730 | | G | A | | | 0.1353106 | | | 0.028974201 | | 1.15E-06 | | 0.0603451 | | | 0.047961 | | | 0.208311 | |  |  |  |  |  |  |
| genus.Peptococcus.id.2037 | | rs7033353 | | T | G | | | 0.090152 | | | 0.018995009 | | 2.22E-06 | | -0.0359074 | | | 0.022893 | | | 0.116767 | |  |  |  |  |  |  |
| genus.Peptococcus.id.2037 | | rs72850165 | | T | C | | | -0.134304 | | | 0.030042318 | | 5.74E-06 | | 0.0348443 | | | 0.042865 | | | 0.416284 | |  |  |  |  |  |  |
| genus.Peptococcus.id.2037 | | rs74592222 | | G | A | | | 0.137957 | | | 0.030296488 | | 8.55E-06 | | 0.0389069 | | | 0.03587 | | | 0.278075 | |  |  |  |  |  |  |
| genus.Peptococcus.id.2037 | | rs75754569 | | C | G | | | 0.1814341 | | | 0.031942874 | | 1.10E-08 | | 0.0528111 | | | 0.039501 | | | 0.181232 | |  |  |  |  |  |  |
| genus.Peptococcus.id.2037 | | rs76697713 | | C | A | | | 0.1463024 | | | 0.032234478 | | 8.05E-06 | | 0.0187505 | | | 0.047489 | | | 0.692958 | |  |  |  |  |  |  |
| genus.Peptococcus.id.2037 | | rs77614201 | | T | C | | | -0.1758638 | | | 0.03875232 | | 5.68E-06 | | -0.0477467 | | | 0.051001 | | | 0.349172 | |  |  |  |  |  |  |
| genus.Peptococcus.id.2037 | | rs7766680 | | G | C | | | 0.0976832 | | | 0.021415492 | | 3.51E-06 | | -0.0147859 | | | 0.027248 | | | 0.587377 | |  |  |  |  |  |  |
| genus.Peptococcus.id.2037 | | rs77681628 | | C | T | | | 0.2003072 | | | 0.038732756 | | 2.69E-07 | | 0.0216189 | | | 0.041941 | | | 0.606227 | |  |  |  |  |  |  |
| genus.Phascolarctobacterium.id.2168 | | rs12618201 | | A | G | | | 0.0641657 | | | 0.013820192 | | 3.38E-06 | | 0.022468 | | | 0.022941 | | | 0.327399 | |  |  |  |  |  |  |
| genus.Phascolarctobacterium.id.2168 | | rs1264476 | | T | G | | | 0.0767335 | | | 0.016604148 | | 4.30E-06 | | -0.0058549 | | | 0.029245 | | | 0.841323 | |  |  |  |  |  |  |
| genus.Phascolarctobacterium.id.2168 | | rs130483 | | A | G | | | 0.0655056 | | | 0.014359557 | | 6.79E-06 | | 0.031648 | | | 0.023289 | | | 0.174168 | |  |  |  |  |  |  |
| genus.Phascolarctobacterium.id.2168 | | rs28525131 | | G | A | | | -0.1186572 | | | 0.026904831 | | 8.23E-06 | | 0.014855 | | | 0.053399 | | | 0.780867 | |  |  |  |  |  |  |
| genus.Phascolarctobacterium.id.2168 | | rs56069061 | | G | A | | | -0.1113058 | | | 0.02306943 | | 1.87E-06 | | 0.0169193 | | | 0.04609 | | | 0.713551 | |  |  |  |  |  |  |
| genus.Phascolarctobacterium.id.2168 | | rs56157888 | | A | C | | | 0.0954864 | | | 0.019397634 | | 1.09E-06 | | -0.017095 | | | 0.027614 | | | 0.535874 | |  |  |  |  |  |  |
| genus.Phascolarctobacterium.id.2168 | | rs6427992 | | G | C | | | -0.0652458 | | | 0.013750826 | | 2.09E-06 | | 0.0238728 | | | 0.023643 | | | 0.312635 | |  |  |  |  |  |  |
| genus.Phascolarctobacterium.id.2168 | | rs74540770 | | G | A | | | -0.1210144 | | | 0.025862072 | | 3.60E-06 | | -0.02647 | | | 0.042212 | | | 0.530612 | |  |  |  |  |  |  |
| genus.Phascolarctobacterium.id.2168 | | rs75882962 | | T | C | | | 0.0968661 | | | 0.019057501 | | 3.19E-07 | | 0.0175034 | | | 0.034548 | | | 0.612411 | |  |  |  |  |  |  |
| genus.Phascolarctobacterium.id.2168 | | rs76124218 | | C | G | | | -0.1593608 | | | 0.03445316 | | 2.67E-06 | | 0.104673 | | | 0.060794 | | | 0.0851138 | |  |  |  |  |  |  |
| genus.Phascolarctobacterium.id.2168 | | rs7982713 | | G | A | | | 0.0726922 | | | 0.016320086 | | 9.72E-06 | | -0.0203026 | | | 0.025475 | | | 0.425471 | |  |  |  |  |  |  |
| genus.Prevotella7.id.11182 | | rs118038478 | | A | G | | | 0.2056756 | | | 0.046891721 | | 7.85E-06 | | -0.0520171 | | | 0.045297 | | | 0.250822 | |  |  |  |  |  |  |
| genus.Prevotella7.id.11182 | | rs12124567 | | A | G | | | -0.1212813 | | | 0.027501731 | | 9.49E-06 | | -0.0313595 | | | 0.028651 | | | 0.273713 | |  |  |  |  |  |  |
| genus.Prevotella7.id.11182 | | rs12195431 | | T | C | | | 0.1965278 | | | 0.044232188 | | 8.73E-06 | | 0.002763 | | | 0.038645 | | | 0.943002 | |  |  |  |  |  |  |
| genus.Prevotella7.id.11182 | | rs16937247 | | G | C | | | 0.1460979 | | | 0.035152923 | | 9.64E-06 | | 0.0105437 | | | 0.028582 | | | 0.712202 | |  |  |  |  |  |  |
| genus.Prevotella7.id.11182 | | rs2240542 | | C | T | | | 0.1208499 | | | 0.026177616 | | 4.84E-06 | | 0.001126 | | | 0.025827 | | | 0.965225 | |  |  |  |  |  |  |
| genus.Prevotella7.id.11182 | | rs2918132 | | C | T | | | -0.1146484 | | | 0.025491556 | | 6.42E-06 | | 0.017977 | | | 0.02363 | | | 0.446786 | |  |  |  |  |  |  |
| genus.Prevotella7.id.11182 | | rs430270 | | A | C | | | 0.1391552 | | | 0.029705527 | | 2.87E-06 | | -0.0293686 | | | 0.029131 | | | 0.313373 | |  |  |  |  |  |  |
| genus.Prevotella7.id.11182 | | rs57404562 | | C | A | | | 0.1554865 | | | 0.031605691 | | 6.22E-07 | | 0.0130515 | | | 0.034067 | | | 0.701636 | |  |  |  |  |  |  |
| genus.Prevotella7.id.11182 | | rs79263163 | | A | C | | | -0.143982 | | | 0.031500978 | | 7.51E-06 | | 0.0132591 | | | 0.028395 | | | 0.640532 | |  |  |  |  |  |  |
| genus.Prevotella7.id.11182 | | rs9426434 | | T | C | | | -0.1236572 | | | 0.027851446 | | 9.72E-06 | | 0.0319086 | | | 0.024077 | | | 0.185081 | |  |  |  |  |  |  |
| genus.Prevotella7.id.11182 | | rs9608249 | | A | G | | | -0.1582133 | | | 0.033631703 | | 2.07E-06 | | -0.0191165 | | | 0.036362 | | | 0.599073 | |  |  |  |  |  |  |
| genus.Prevotella7.id.11182 | | rs9959718 | | G | A | | | 0.133012 | | | 0.027536133 | | 1.90E-06 | | -0.0273236 | | | 0.028372 | | | 0.335517 | |  |  |  |  |  |  |
| genus.Prevotella9.id.11183 | | rs10512344 | | C | G | | | 0.2473728 | | | 0.054408651 | | 3.19E-06 | | -0.0232038 | | | 0.082545 | | | 0.77863 | |  |  |  |  |  |  |
| genus.Prevotella9.id.11183 | | rs111509883 | | T | C | | | 0.1711306 | | | 0.03476241 | | 1.24E-06 | | -0.0182883 | | | 0.036728 | | | 0.618527 | |  |  |  |  |  |  |
| genus.Prevotella9.id.11183 | | rs11199734 | | A | T | | | 0.0770209 | | | 0.016946218 | | 7.00E-06 | | -0.002578 | | | 0.027657 | | | 0.925734 | |  |  |  |  |  |  |
| genus.Prevotella9.id.11183 | | rs11685699 | | C | T | | | -0.1413574 | | | 0.029566474 | | 2.03E-06 | | -0.0188092 | | | 0.04301 | | | 0.661881 | |  |  |  |  |  |  |
| genus.Prevotella9.id.11183 | | rs117271932 | | A | G | | | 0.2080886 | | | 0.044039277 | | 2.82E-06 | | -0.0950533 | | | 0.04957 | | | 0.0551696 | |  |  |  |  |  |  |
| genus.Prevotella9.id.11183 | | rs12648235 | | T | C | | | 0.0785902 | | | 0.017767553 | | 7.39E-06 | | 0.0140968 | | | 0.027122 | | | 0.603227 | |  |  |  |  |  |  |
| genus.Prevotella9.id.11183 | | rs1304512 | | G | A | | | 0.0761593 | | | 0.016588486 | | 5.29E-06 | | -0.0621796 | | | 0.025738 | | | 0.0156975 | |  |  |  |  |  |  |
| genus.Prevotella9.id.11183 | | rs2104588 | | T | C | | | 0.1055585 | | | 0.023773023 | | 8.13E-06 | | 0.0618695 | | | 0.049763 | | | 0.213766 | |  |  |  |  |  |  |
| genus.Prevotella9.id.11183 | | rs2495052 | | A | G | | | 0.0838978 | | | 0.018852315 | | 8.97E-06 | | -0.0216034 | | | 0.032189 | | | 0.502125 | |  |  |  |  |  |  |
| genus.Prevotella9.id.11183 | | rs2683313 | | A | G | | | -0.0724576 | | | 0.015160279 | | 1.69E-06 | | 0.0018857 | | | 0.024553 | | | 0.938782 | |  |  |  |  |  |  |
| genus.Prevotella9.id.11183 | | rs4821647 | | G | C | | | 0.0636957 | | | 0.014398271 | | 9.95E-06 | | 0.0063656 | | | 0.023902 | | | 0.789993 | |  |  |  |  |  |  |
| genus.Prevotella9.id.11183 | | rs4968431 | | G | T | | | 0.0640113 | | | 0.014416226 | | 8.58E-06 | | 0.0091744 | | | 0.023823 | | | 0.700161 | |  |  |  |  |  |  |
| genus.Prevotella9.id.11183 | | rs7232121 | | G | C | | | 0.0671292 | | | 0.014422959 | | 3.76E-06 | | 0.0050987 | | | 0.02305 | | | 0.82494 | |  |  |  |  |  |  |
| genus.Prevotella9.id.11183 | | rs7237249 | | C | T | | | -0.0823764 | | | 0.018212594 | | 8.93E-06 | | -0.0098775 | | | 0.029151 | | | 0.734734 | |  |  |  |  |  |  |
| genus.Prevotella9.id.11183 | | rs72815774 | | T | C | | | -0.1761858 | | | 0.039306766 | | 8.78E-06 | | 0.0203851 | | | 0.048919 | | | 0.676888 | |  |  |  |  |  |  |
| genus.Prevotella9.id.11183 | | rs746764 | | T | C | | | -0.0915772 | | | 0.019324484 | | 2.04E-06 | | -0.0250619 | | | 0.02781 | | | 0.367484 | |  |  |  |  |  |  |
| genus.Prevotella9.id.11183 | | rs7976209 | | T | C | | | -0.0870786 | | | 0.019795052 | | 7.28E-06 | | -0.0199681 | | | 0.031558 | | | 0.526906 | |  |  |  |  |  |  |
| genus.Prevotella9.id.11183 | | rs9428102 | | A | G | | | -0.0778845 | | | 0.017606807 | | 4.62E-06 | | 0.0117082 | | | 0.027693 | | | 0.672448 | |  |  |  |  |  |  |
| genus.Prevotella9.id.11183 | | rs9613013 | | G | A | | | 0.0917591 | | | 0.020269664 | | 6.10E-06 | | 0.0103969 | | | 0.034905 | | | 0.765808 | |  |  |  |  |  |  |
| genus.RikenellaceaeRC9gutgroup.id.11191 | | rs11597218 | | T | C | | | 0.1135691 | | | 0.025164508 | | 5.63E-06 | | 0.0218508 | | | 0.023176 | | | 0.34577 | |  |  |  |  |  |  |
| genus.RikenellaceaeRC9gutgroup.id.11191 | | rs12501673 | | A | G | | | 0.1163954 | | | 0.026225344 | | 6.29E-06 | | 0.0227896 | | | 0.025872 | | | 0.378398 | |  |  |  |  |  |  |
| genus.RikenellaceaeRC9gutgroup.id.11191 | | rs17032291 | | T | C | | | -0.1695786 | | | 0.036825126 | | 6.61E-06 | | -0.012817 | | | 0.034754 | | | 0.71228 | |  |  |  |  |  |  |
| genus.RikenellaceaeRC9gutgroup.id.11191 | | rs17582787 | | A | G | | | -0.1577394 | | | 0.033992444 | | 3.55E-06 | | -0.0413113 | | | 0.030677 | | | 0.178093 | |  |  |  |  |  |  |
| genus.RikenellaceaeRC9gutgroup.id.11191 | | rs2074881 | | T | C | | | -0.142236 | | | 0.032390968 | | 9.45E-06 | | 0.0305898 | | | 0.033999 | | | 0.368259 | |  |  |  |  |  |  |
| genus.RikenellaceaeRC9gutgroup.id.11191 | | rs2900503 | | G | T | | | -0.1723292 | | | 0.032667127 | | 1.55E-07 | | 0.022721 | | | 0.030963 | | | 0.463058 | |  |  |  |  |  |  |
| genus.RikenellaceaeRC9gutgroup.id.11191 | | rs2998141 | | T | C | | | -0.1363464 | | | 0.029307262 | | 4.42E-06 | | -0.0232022 | | | 0.027043 | | | 0.390914 | |  |  |  |  |  |  |
| genus.RikenellaceaeRC9gutgroup.id.11191 | | rs4270579 | | G | A | | | -0.1180213 | | | 0.027104126 | | 5.46E-06 | | -0.0068268 | | | 0.024402 | | | 0.779658 | |  |  |  |  |  |  |
| genus.RikenellaceaeRC9gutgroup.id.11191 | | rs4717843 | | G | T | | | -0.1193761 | | | 0.026054834 | | 4.72E-06 | | -0.0054053 | | | 0.022928 | | | 0.813625 | |  |  |  |  |  |  |
| genus.RikenellaceaeRC9gutgroup.id.11191 | | rs7113155 | | G | C | | | 0.1144226 | | | 0.024779825 | | 5.26E-06 | | -0.0065607 | | | 0.023728 | | | 0.782164 | |  |  |  |  |  |  |
| genus.RikenellaceaeRC9gutgroup.id.11191 | | rs7193937 | | G | C | | | 0.1241533 | | | 0.027741031 | | 6.19E-06 | | 0.0259943 | | | 0.023952 | | | 0.277802 | |  |  |  |  |  |  |
| genus.RikenellaceaeRC9gutgroup.id.11191 | | rs7712231 | | A | G | | | 0.1562838 | | | 0.035046199 | | 7.97E-06 | | -0.0357204 | | | 0.033579 | | | 0.287426 | |  |  |  |  |  |  |
| genus.RikenellaceaeRC9gutgroup.id.11191 | | rs80309088 | | G | A | | | 0.17405 | | | 0.038342318 | | 4.56E-06 | | 0.0214635 | | | 0.03514 | | | 0.541335 | |  |  |  |  |  |  |
| genus.RikenellaceaeRC9gutgroup.id.11191 | | rs9887954 | | G | A | | | -0.1148494 | | | 0.024901293 | | 4.81E-06 | | 0.0012452 | | | 0.023241 | | | 0.957272 | |  |  |  |  |  |  |
| genus.Romboutsia.id.11347 | | rs10091895 | | T | C | | | -0.0649918 | | | 0.014550099 | | 6.62E-06 | | -0.0381397 | | | 0.027065 | | | 0.158779 | |  |  |  |  |  |  |
| genus.Romboutsia.id.11347 | | rs10279978 | | A | G | | | -0.0622202 | | | 0.012767535 | | 1.17E-06 | | 0.038053 | | | 0.024597 | | | 0.121853 | |  |  |  |  |  |  |
| genus.Romboutsia.id.11347 | | rs11221428 | | T | C | | | -0.0726779 | | | 0.015826331 | | 6.49E-06 | | 0.0106095 | | | 0.027179 | | | 0.696277 | |  |  |  |  |  |  |
| genus.Romboutsia.id.11347 | | rs114398731 | | G | C | | | -0.1310386 | | | 0.029381546 | | 7.96E-06 | | -0.0321196 | | | 0.050341 | | | 0.523443 | |  |  |  |  |  |  |
| genus.Romboutsia.id.11347 | | rs16843578 | | C | T | | | -0.0875276 | | | 0.019677862 | | 5.08E-06 | | -0.0171191 | | | 0.051097 | | | 0.7376 | |  |  |  |  |  |  |
| genus.Romboutsia.id.11347 | | rs28603357 | | T | C | | | -0.214911 | | | 0.047473928 | | 8.52E-06 | | -0.044251 | | | 0.081551 | | | 0.587392 | |  |  |  |  |  |  |
| genus.Romboutsia.id.11347 | | rs34302036 | | A | G | | | 0.0550261 | | | 0.012083475 | | 5.88E-06 | | -0.0424691 | | | 0.023155 | | | 0.0666408 | |  |  |  |  |  |  |
| genus.Romboutsia.id.11347 | | rs61841503 | | G | A | | | 0.092888 | | | 0.017145406 | | 4.00E-08 | | -0.0803932 | | | 0.033972 | | | 0.0179606 | |  |  |  |  |  |  |
| genus.Romboutsia.id.11347 | | rs62504452 | | A | G | | | -0.0710048 | | | 0.015665426 | | 4.66E-06 | | -0.0242852 | | | 0.032969 | | | 0.461356 | |  |  |  |  |  |  |
| genus.Romboutsia.id.11347 | | rs7109293 | | A | G | | | 0.0919112 | | | 0.020573834 | | 6.98E-06 | | 0.0825162 | | | 0.035829 | | | 0.0212745 | |  |  |  |  |  |  |
| genus.Romboutsia.id.11347 | | rs75200530 | | T | G | | | -0.1906444 | | | 0.042106493 | | 5.07E-06 | | 0.0026381 | | | 0.066896 | | | 0.968543 | |  |  |  |  |  |  |
| genus.Romboutsia.id.11347 | | rs75987356 | | G | A | | | -0.1295784 | | | 0.028045444 | | 6.71E-06 | | 0.0592272 | | | 0.042576 | | | 0.164198 | |  |  |  |  |  |  |
| genus.Romboutsia.id.11347 | | rs77702691 | | A | G | | | -0.094406 | | | 0.020854443 | | 7.37E-06 | | 0.0023031 | | | 0.040274 | | | 0.954396 | |  |  |  |  |  |  |
| genus.Romboutsia.id.11347 | | rs9389266 | | T | G | | | 0.0722815 | | | 0.016235341 | | 9.38E-06 | | 0.0490078 | | | 0.030014 | | | 0.102507 | |  |  |  |  |  |  |
| genus.Romboutsia.id.11347 | | rs9567264 | | C | T | | | 0.0580018 | | | 0.01274947 | | 5.76E-06 | | -0.0344328 | | | 0.024169 | | | 0.154252 | |  |  |  |  |  |  |
| genus.Roseburia.id.2012 | | rs116270582 | | T | A | | | -0.1538439 | | | 0.032932399 | | 1.20E-06 | | 0.0778235 | | | 0.056353 | | | 0.167275 | |  |  |  |  |  |  |
| genus.Roseburia.id.2012 | | rs12740451 | | T | C | | | 0.0697527 | | | 0.015360737 | | 7.34E-06 | | -0.0127005 | | | 0.033484 | | | 0.704464 | |  |  |  |  |  |  |
| genus.Roseburia.id.2012 | | rs147990086 | | A | G | | | -0.0578936 | | | 0.013239999 | | 8.93E-06 | | -0.0100786 | | | 0.029967 | | | 0.736631 | |  |  |  |  |  |  |
| genus.Roseburia.id.2012 | | rs16910295 | | T | C | | | -0.0980434 | | | 0.020956968 | | 2.91E-06 | | -0.0096376 | | | 0.050601 | | | 0.848948 | |  |  |  |  |  |  |
| genus.Roseburia.id.2012 | | rs2034589 | | G | C | | | 0.0629765 | | | 0.012348898 | | 5.01E-07 | | 0.0279991 | | | 0.02992 | | | 0.349379 | |  |  |  |  |  |  |
| genus.Roseburia.id.2012 | | rs2160994 | | T | C | | | 0.0550687 | | | 0.011248106 | | 9.70E-07 | | -0.0101615 | | | 0.024132 | | | 0.673698 | |  |  |  |  |  |  |
| genus.Roseburia.id.2012 | | rs28040 | | G | C | | | 0.0565693 | | | 0.012679199 | | 9.26E-06 | | -0.0131322 | | | 0.028605 | | | 0.646175 | |  |  |  |  |  |  |
| genus.Roseburia.id.2012 | | rs2943022 | | T | C | | | 0.0493786 | | | 0.010676336 | | 4.11E-06 | | 0.0029799 | | | 0.023179 | | | 0.897708 | |  |  |  |  |  |  |
| genus.Roseburia.id.2012 | | rs302266 | | T | C | | | -0.0777312 | | | 0.01729852 | | 8.13E-06 | | -0.0506175 | | | 0.034351 | | | 0.140604 | |  |  |  |  |  |  |
| genus.Roseburia.id.2012 | | rs329182 | | T | C | | | 0.0690327 | | | 0.015288408 | | 5.90E-06 | | -0.0075756 | | | 0.031147 | | | 0.807833 | |  |  |  |  |  |  |
| genus.Roseburia.id.2012 | | rs4748237 | | G | C | | | 0.0488326 | | | 0.010640433 | | 4.67E-06 | | 0.0171288 | | | 0.023008 | | | 0.456586 | |  |  |  |  |  |  |
| genus.Roseburia.id.2012 | | rs55858165 | | A | C | | | 0.1792844 | | | 0.040495413 | | 9.99E-06 | | -0.0422511 | | | 0.06014 | | | 0.482339 | |  |  |  |  |  |  |
| genus.Roseburia.id.2012 | | rs57466170 | | C | T | | | 0.0741405 | | | 0.017160095 | | 8.30E-06 | | -0.0276135 | | | 0.043389 | | | 0.524501 | |  |  |  |  |  |  |
| genus.Roseburia.id.2012 | | rs6445851 | | G | A | | | -0.0497336 | | | 0.010815826 | | 3.53E-06 | | -0.0720741 | | | 0.023625 | | | 0.00228276 | |  |  |  |  |  |  |
| genus.Roseburia.id.2012 | | rs6930661 | | C | T | | | -0.0961587 | | | 0.020497475 | | 2.48E-06 | | 0.0366213 | | | 0.047849 | | | 0.444066 | |  |  |  |  |  |  |
| genus.Roseburia.id.2012 | | rs75326254 | | C | T | | | -0.1046278 | | | 0.023089702 | | 7.50E-06 | | -0.0062227 | | | 0.048344 | | | 0.897583 | |  |  |  |  |  |  |
| genus.Roseburia.id.2012 | | rs78753150 | | A | C | | | 0.0968739 | | | 0.021406676 | | 9.98E-06 | | 0.0267648 | | | 0.038086 | | | 0.482213 | |  |  |  |  |  |  |
| genus.Roseburia.id.2012 | | rs9300744 | | C | T | | | -0.0588452 | | | 0.012622789 | | 4.75E-06 | | 0.0085071 | | | 0.029799 | | | 0.77527 | |  |  |  |  |  |  |
| genus.Ruminiclostridium5.id.11355 | | rs10827477 | | A | G | | | -0.0547387 | | | 0.011516344 | | 2.19E-06 | | 0.0388211 | | | 0.023907 | | | 0.104413 | |  |  |  |  |  |  |
| genus.Ruminiclostridium5.id.11355 | | rs113753996 | | T | C | | | 0.0820689 | | | 0.017446254 | | 3.99E-06 | | -0.0196167 | | | 0.029575 | | | 0.507147 | |  |  |  |  |  |  |
| genus.Ruminiclostridium5.id.11355 | | rs1223978 | | T | C | | | 0.0484169 | | | 0.010825421 | | 8.16E-06 | | 0.0349716 | | | 0.022983 | | | 0.1281 | |  |  |  |  |  |  |
| genus.Ruminiclostridium5.id.11355 | | rs1492620 | | T | C | | | -0.0830505 | | | 0.018007297 | | 3.53E-06 | | 0.0203634 | | | 0.034368 | | | 0.553509 | |  |  |  |  |  |  |
| genus.Ruminiclostridium5.id.11355 | | rs2286384 | | G | C | | | -0.0518814 | | | 0.010748225 | | 1.44E-06 | | -0.0103965 | | | 0.022768 | | | 0.647936 | |  |  |  |  |  |  |
| genus.Ruminiclostridium5.id.11355 | | rs243585 | | C | G | | | -0.0585939 | | | 0.012101692 | | 1.33E-06 | | 0.0218551 | | | 0.025656 | | | 0.394289 | |  |  |  |  |  |  |
| genus.Ruminiclostridium5.id.11355 | | rs2482038 | | C | A | | | 0.0518991 | | | 0.010877683 | | 1.70E-06 | | -0.0092464 | | | 0.023286 | | | 0.691305 | |  |  |  |  |  |  |
| genus.Ruminiclostridium5.id.11355 | | rs2791343 | | T | C | | | 0.051717 | | | 0.011336879 | | 5.54E-06 | | -0.0137001 | | | 0.023331 | | | 0.55706 | |  |  |  |  |  |  |
| genus.Ruminiclostridium5.id.11355 | | rs2801960 | | C | G | | | 0.0520975 | | | 0.011511124 | | 6.21E-06 | | -0.0023327 | | | 0.025027 | | | 0.925739 | |  |  |  |  |  |  |
| genus.Ruminiclostridium5.id.11355 | | rs2833828 | | G | A | | | 0.0489617 | | | 0.010870209 | | 6.82E-06 | | 0.0077698 | | | 0.023205 | | | 0.737753 | |  |  |  |  |  |  |
| genus.Ruminiclostridium5.id.11355 | | rs4955951 | | A | G | | | -0.0713749 | | | 0.01658264 | | 9.96E-06 | | 0.0125119 | | | 0.035471 | | | 0.724289 | |  |  |  |  |  |  |
| genus.Ruminiclostridium5.id.11355 | | rs6121460 | | G | A | | | 0.0932955 | | | 0.019920597 | | 2.64E-06 | | 0.0428805 | | | 0.041878 | | | 0.305865 | |  |  |  |  |  |  |
| genus.Ruminiclostridium5.id.11355 | | rs73002572 | | G | C | | | 0.1815475 | | | 0.041407845 | | 8.82E-06 | | -0.0025127 | | | 0.035766 | | | 0.943991 | |  |  |  |  |  |  |
| genus.Ruminiclostridium5.id.11355 | | rs79968837 | | A | G | | | -0.0950311 | | | 0.019350746 | | 1.15E-06 | | 0.0789075 | | | 0.051608 | | | 0.126267 | |  |  |  |  |  |  |
| genus.Ruminiclostridium5.id.11355 | | rs8053158 | | A | G | | | -0.0740543 | | | 0.015915101 | | 5.90E-06 | | 0.0127803 | | | 0.03496 | | | 0.714687 | |  |  |  |  |  |  |
| genus.Ruminiclostridium6.id.11356 | | rs10829821 | | T | C | | | -0.097606 | | | 0.021607241 | | 3.47E-06 | | 0.000195 | | | 0.039681 | | | 0.996079 | |  |  |  |  |  |  |
| genus.Ruminiclostridium6.id.11356 | | rs116969552 | | A | G | | | -0.1668225 | | | 0.037668056 | | 9.16E-06 | | -0.0401428 | | | 0.068365 | | | 0.557083 | |  |  |  |  |  |  |
| genus.Ruminiclostridium6.id.11356 | | rs11992182 | | A | C | | | 0.0625299 | | | 0.013787562 | | 4.65E-06 | | -0.0109125 | | | 0.027447 | | | 0.690933 | |  |  |  |  |  |  |
| genus.Ruminiclostridium6.id.11356 | | rs1871858 | | C | G | | | -0.1054215 | | | 0.023727442 | | 9.12E-06 | | 0.0100927 | | | 0.036134 | | | 0.780004 | |  |  |  |  |  |  |
| genus.Ruminiclostridium6.id.11356 | | rs2548459 | | C | T | | | 0.0554866 | | | 0.012275456 | | 6.40E-06 | | 0.0026928 | | | 0.023159 | | | 0.907437 | |  |  |  |  |  |  |
| genus.Ruminiclostridium6.id.11356 | | rs35362464 | | C | A | | | 0.0720077 | | | 0.016538673 | | 8.99E-06 | | -0.0015078 | | | 0.032926 | | | 0.963475 | |  |  |  |  |  |  |
| genus.Ruminiclostridium6.id.11356 | | rs61060922 | | T | G | | | 0.1591297 | | | 0.032228344 | | 1.09E-06 | | -0.0320519 | | | 0.064262 | | | 0.617942 | |  |  |  |  |  |  |
| genus.Ruminiclostridium6.id.11356 | | rs663262 | | T | C | | | -0.1349804 | | | 0.03107145 | | 3.39E-06 | | -0.0558449 | | | 0.064511 | | | 0.386671 | |  |  |  |  |  |  |
| genus.Ruminiclostridium6.id.11356 | | rs67479537 | | T | C | | | 0.1189942 | | | 0.026487011 | | 9.30E-06 | | 0.0116769 | | | 0.053523 | | | 0.827298 | |  |  |  |  |  |  |
| genus.Ruminiclostridium6.id.11356 | | rs71414120 | | T | G | | | 0.2008941 | | | 0.040643398 | | 1.08E-06 | | -0.0183762 | | | 0.052028 | | | 0.723941 | |  |  |  |  |  |  |
| genus.Ruminiclostridium6.id.11356 | | rs72991535 | | T | G | | | 0.1356039 | | | 0.029517443 | | 4.95E-06 | | 0.0206363 | | | 0.062768 | | | 0.74233 | |  |  |  |  |  |  |
| genus.Ruminiclostridium6.id.11356 | | rs73176030 | | T | C | | | 0.058665 | | | 0.013207973 | | 7.29E-06 | | 0.005432 | | | 0.025644 | | | 0.832248 | |  |  |  |  |  |  |
| genus.Ruminiclostridium6.id.11356 | | rs77193512 | | A | G | | | 0.0736533 | | | 0.015316349 | | 1.30E-06 | | -0.0373462 | | | 0.026472 | | | 0.158303 | |  |  |  |  |  |  |
| genus.Ruminiclostridium6.id.11356 | | rs792058 | | G | A | | | 0.0554234 | | | 0.012542318 | | 8.58E-06 | | 0.0360363 | | | 0.02323 | | | 0.12084 | |  |  |  |  |  |  |
| genus.Ruminiclostridium6.id.11356 | | rs79968172 | | G | A | | | 0.1161396 | | | 0.02430834 | | 1.66E-06 | | 0.0255778 | | | 0.048641 | | | 0.598995 | |  |  |  |  |  |  |
| genus.Ruminiclostridium6.id.11356 | | rs9555756 | | A | C | | | -0.0804138 | | | 0.017669194 | | 7.10E-06 | | -0.0336487 | | | 0.041132 | | | 0.413322 | |  |  |  |  |  |  |
| genus.Ruminiclostridium9.id.11357 | | rs113048721 | | C | G | | | 0.0600735 | | | 0.013273731 | | 4.10E-06 | | -0.0556564 | | | 0.032255 | | | 0.0844306 | |  |  |  |  |  |  |
| genus.Ruminiclostridium9.id.11357 | | rs12040548 | | G | T | | | 0.0570422 | | | 0.012235891 | | 3.15E-06 | | 0.0068917 | | | 0.025675 | | | 0.788372 | |  |  |  |  |  |  |
| genus.Ruminiclostridium9.id.11357 | | rs12419854 | | T | A | | | -0.0727975 | | | 0.015559424 | | 3.18E-06 | | -0.0499341 | | | 0.025851 | | | 0.0534035 | |  |  |  |  |  |  |
| genus.Ruminiclostridium9.id.11357 | | rs13033315 | | T | A | | | 0.0511195 | | | 0.011141773 | | 5.68E-06 | | 0.0085305 | | | 0.024445 | | | 0.727113 | |  |  |  |  |  |  |
| genus.Ruminiclostridium9.id.11357 | | rs2615052 | | T | G | | | 0.0577608 | | | 0.012057751 | | 1.75E-06 | | -0.0134126 | | | 0.025543 | | | 0.599512 | |  |  |  |  |  |  |
| genus.Ruminiclostridium9.id.11357 | | rs55875763 | | G | C | | | -0.0545331 | | | 0.01129737 | | 1.60E-06 | | -0.0164719 | | | 0.023253 | | | 0.478704 | |  |  |  |  |  |  |
| genus.Ruminiclostridium9.id.11357 | | rs57665991 | | C | G | | | -0.0641507 | | | 0.012333861 | | 2.07E-07 | | -0.0608306 | | | 0.0265 | | | 0.021706 | |  |  |  |  |  |  |
| genus.Ruminiclostridium9.id.11357 | | rs6082461 | | A | C | | | 0.0586425 | | | 0.013100394 | | 4.87E-06 | | 0.0053715 | | | 0.028365 | | | 0.849801 | |  |  |  |  |  |  |
| genus.Ruminiclostridium9.id.11357 | | rs7137760 | | C | T | | | 0.050797 | | | 0.011218236 | | 7.07E-06 | | 0.0182943 | | | 0.022932 | | | 0.425008 | |  |  |  |  |  |  |
| genus.Ruminiclostridium9.id.11357 | | rs73592673 | | A | T | | | -0.081629 | | | 0.016951466 | | 2.14E-06 | | -0.0440068 | | | 0.033901 | | | 0.194252 | |  |  |  |  |  |  |
| genus.Ruminiclostridium9.id.11357 | | rs74303178 | | T | C | | | 0.0532742 | | | 0.011927361 | | 7.92E-06 | | 0.0074014 | | | 0.024548 | | | 0.763022 | |  |  |  |  |  |  |
| genus.Ruminiclostridium9.id.11357 | | rs78191726 | | T | C | | | 0.0944998 | | | 0.02102058 | | 7.58E-06 | | -0.0141195 | | | 0.043876 | | | 0.747599 | |  |  |  |  |  |  |
| genus.Ruminiclostridium9.id.11357 | | rs79082720 | | C | G | | | 0.0928576 | | | 0.020487143 | | 6.47E-06 | | -0.0499219 | | | 0.052951 | | | 0.345789 | |  |  |  |  |  |  |
| genus.Ruminiclostridium9.id.11357 | | rs918449 | | A | G | | | -0.0950908 | | | 0.019717682 | | 2.56E-06 | | -0.0124987 | | | 0.044249 | | | 0.777589 | |  |  |  |  |  |  |
| genus.Ruminiclostridium9.id.11357 | | rs9522712 | | T | C | | | 0.0699728 | | | 0.015493905 | | 4.66E-06 | | 0.0408466 | | | 0.032281 | | | 0.205749 | |  |  |  |  |  |  |
| genus.Ruminiclostridium9.id.11357 | | rs9809789 | | C | T | | | -0.0717454 | | | 0.015967315 | | 8.72E-06 | | 0.0163837 | | | 0.029584 | | | 0.579712 | |  |  |  |  |  |  |
| genus.RuminococcaceaeNK4A214group.id.11358 | | rs11241747 | | C | T | | | 0.0533858 | | | 0.012003462 | | 6.59E-06 | | 0.0039811 | | | 0.025141 | | | 0.87418 | |  |  |  |  |  |  |
| genus.RuminococcaceaeNK4A214group.id.11358 | | rs114244418 | | C | G | | | -0.1752662 | | | 0.037277927 | | 3.59E-06 | | -0.0400483 | | | 0.057903 | | | 0.489158 | |  |  |  |  |  |  |
| genus.RuminococcaceaeNK4A214group.id.11358 | | rs11586410 | | G | A | | | -0.0863422 | | | 0.016993683 | | 3.66E-07 | | -0.0359617 | | | 0.031623 | | | 0.255453 | |  |  |  |  |  |  |
| genus.RuminococcaceaeNK4A214group.id.11358 | | rs11704699 | | G | T | | | -0.0899695 | | | 0.018475237 | | 8.71E-07 | | -0.062516 | | | 0.037616 | | | 0.0965228 | |  |  |  |  |  |  |
| genus.RuminococcaceaeNK4A214group.id.11358 | | rs12642039 | | T | C | | | -0.0553003 | | | 0.011939746 | | 3.43E-06 | | -0.0012928 | | | 0.023763 | | | 0.956614 | |  |  |  |  |  |  |
| genus.RuminococcaceaeNK4A214group.id.11358 | | rs12731 | | A | G | | | -0.0527684 | | | 0.01150534 | | 4.87E-06 | | 0.0069331 | | | 0.023549 | | | 0.768438 | |  |  |  |  |  |  |
| genus.RuminococcaceaeNK4A214group.id.11358 | | rs13087692 | | T | G | | | 0.0574623 | | | 0.012593957 | | 8.69E-06 | | 0.0580143 | | | 0.024987 | | | 0.0202423 | |  |  |  |  |  |  |
| genus.RuminococcaceaeNK4A214group.id.11358 | | rs136761 | | G | A | | | -0.0587521 | | | 0.01191547 | | 8.15E-07 | | 0.0305568 | | | 0.023763 | | | 0.198475 | |  |  |  |  |  |  |
| genus.RuminococcaceaeNK4A214group.id.11358 | | rs147475196 | | A | G | | | -0.1337686 | | | 0.029519585 | | 4.72E-06 | | -0.0263793 | | | 0.037386 | | | 0.480437 | |  |  |  |  |  |  |
| genus.RuminococcaceaeNK4A214group.id.11358 | | rs34576931 | | G | C | | | -0.0873312 | | | 0.019488596 | | 4.72E-06 | | -0.037285 | | | 0.044992 | | | 0.407277 | |  |  |  |  |  |  |
| genus.RuminococcaceaeNK4A214group.id.11358 | | rs35559912 | | T | C | | | -0.0925071 | | | 0.020367383 | | 4.89E-06 | | -0.0220558 | | | 0.035043 | | | 0.529093 | |  |  |  |  |  |  |
| genus.RuminococcaceaeNK4A214group.id.11358 | | rs4814689 | | C | T | | | -0.1083003 | | | 0.023070832 | | 4.55E-06 | | -0.100792 | | | 0.055275 | | | 0.0682354 | |  |  |  |  |  |  |
| genus.RuminococcaceaeNK4A214group.id.11358 | | rs5994253 | | A | G | | | -0.0811302 | | | 0.015762089 | | 2.35E-07 | | -0.0398726 | | | 0.032714 | | | 0.222904 | |  |  |  |  |  |  |
| genus.RuminococcaceaeNK4A214group.id.11358 | | rs62027366 | | T | C | | | 0.0615389 | | | 0.013761184 | | 6.58E-06 | | -0.0031089 | | | 0.028404 | | | 0.912843 | |  |  |  |  |  |  |
| genus.RuminococcaceaeNK4A214group.id.11358 | | rs6681678 | | C | T | | | -0.1001742 | | | 0.024000023 | | 9.05E-06 | | 0.0125219 | | | 0.063193 | | | 0.842924 | |  |  |  |  |  |  |
| genus.RuminococcaceaeNK4A214group.id.11358 | | rs73158814 | | C | G | | | -0.1092573 | | | 0.022745845 | | 2.20E-06 | | 0.0027312 | | | 0.062727 | | | 0.96527 | |  |  |  |  |  |  |
| genus.RuminococcaceaeNK4A214group.id.11358 | | rs7573569 | | T | C | | | 0.1077385 | | | 0.023363816 | | 3.23E-06 | | 0.0897675 | | | 0.047845 | | | 0.0606262 | |  |  |  |  |  |  |
| genus.RuminococcaceaeUCG002.id.11360 | | rs10916131 | | C | T | | | -0.069333 | | | 0.014675325 | | 2.87E-06 | | 0.0215511 | | | 0.031108 | | | 0.488443 | |  |  |  |  |  |  |
| genus.RuminococcaceaeUCG002.id.11360 | | rs10927423 | | C | A | | | -0.0713617 | | | 0.014771224 | | 8.50E-07 | | -0.0403497 | | | 0.029908 | | | 0.177303 | |  |  |  |  |  |  |
| genus.RuminococcaceaeUCG002.id.11360 | | rs10964441 | | G | A | | | -0.1490601 | | | 0.034485997 | | 7.45E-06 | | -0.0209621 | | | 0.03756 | | | 0.576774 | |  |  |  |  |  |  |
| genus.RuminococcaceaeUCG002.id.11360 | | rs113147300 | | A | G | | | -0.075842 | | | 0.016456212 | | 7.69E-06 | | -0.0261488 | | | 0.033269 | | | 0.431875 | |  |  |  |  |  |  |
| genus.RuminococcaceaeUCG002.id.11360 | | rs11607472 | | A | G | | | -0.0780229 | | | 0.017632484 | | 7.19E-06 | | -0.0630141 | | | 0.045419 | | | 0.165318 | |  |  |  |  |  |  |
| genus.RuminococcaceaeUCG002.id.11360 | | rs116974815 | | C | A | | | -0.1897305 | | | 0.039656625 | | 2.03E-06 | | -0.0495034 | | | 0.044965 | | | 0.270922 | |  |  |  |  |  |  |
| genus.RuminococcaceaeUCG002.id.11360 | | rs11750293 | | G | T | | | -0.0578304 | | | 0.012051229 | | 1.76E-06 | | -0.0033521 | | | 0.023749 | | | 0.887757 | |  |  |  |  |  |  |
| genus.RuminococcaceaeUCG002.id.11360 | | rs12463378 | | A | G | | | -0.0522061 | | | 0.011208649 | | 2.96E-06 | | -0.0230999 | | | 0.024872 | | | 0.353016 | |  |  |  |  |  |  |
| genus.RuminococcaceaeUCG002.id.11360 | | rs15256 | | C | T | | | 0.0732376 | | | 0.016833997 | | 9.46E-06 | | -0.0315447 | | | 0.035317 | | | 0.371757 | |  |  |  |  |  |  |
| genus.RuminococcaceaeUCG002.id.11360 | | rs2265670 | | C | G | | | -0.0512404 | | | 0.010928696 | | 2.99E-06 | | -0.0359415 | | | 0.022869 | | | 0.116034 | |  |  |  |  |  |  |
| genus.RuminococcaceaeUCG002.id.11360 | | rs362417 | | G | C | | | -0.054863 | | | 0.01209839 | | 7.80E-06 | | 0.0114946 | | | 0.027897 | | | 0.680307 | |  |  |  |  |  |  |
| genus.RuminococcaceaeUCG002.id.11360 | | rs55793120 | | T | C | | | 0.1373961 | | | 0.027413962 | | 4.81E-07 | | -0.0916309 | | | 0.048326 | | | 0.0579455 | |  |  |  |  |  |  |
| genus.RuminococcaceaeUCG002.id.11360 | | rs57079348 | | T | G | | | -0.076573 | | | 0.01728195 | | 7.22E-06 | | -0.0084307 | | | 0.0494 | | | 0.864488 | |  |  |  |  |  |  |
| genus.RuminococcaceaeUCG002.id.11360 | | rs6542556 | | A | G | | | 0.050974 | | | 0.011406021 | | 7.86E-06 | | 0.0340419 | | | 0.023509 | | | 0.147614 | |  |  |  |  |  |  |
| genus.RuminococcaceaeUCG002.id.11360 | | rs67746927 | | C | G | | | -0.054237 | | | 0.011035718 | | 9.17E-07 | | 0.0040084 | | | 0.023159 | | | 0.862585 | |  |  |  |  |  |  |
| genus.RuminococcaceaeUCG002.id.11360 | | rs6793778 | | C | T | | | -0.0558706 | | | 0.012525794 | | 9.81E-06 | | 0.0337458 | | | 0.026022 | | | 0.194695 | |  |  |  |  |  |  |
| genus.RuminococcaceaeUCG002.id.11360 | | rs7120052 | | A | C | | | 0.0624796 | | | 0.013552314 | | 1.97E-06 | | -0.0322986 | | | 0.029143 | | | 0.267742 | |  |  |  |  |  |  |
| genus.RuminococcaceaeUCG002.id.11360 | | rs7155595 | | C | A | | | 0.0569929 | | | 0.011698636 | | 1.15E-06 | | 0.0048702 | | | 0.024889 | | | 0.84486 | |  |  |  |  |  |  |
| genus.RuminococcaceaeUCG002.id.11360 | | rs7249614 | | A | G | | | -0.0492778 | | | 0.011080679 | | 9.07E-06 | | -0.0256069 | | | 0.023618 | | | 0.278268 | |  |  |  |  |  |  |
| genus.RuminococcaceaeUCG002.id.11360 | | rs72874194 | | G | C | | | -0.0770289 | | | 0.016724954 | | 3.47E-06 | | 0.0670157 | | | 0.033199 | | | 0.0435281 | |  |  |  |  |  |  |
| genus.RuminococcaceaeUCG002.id.11360 | | rs76847269 | | A | G | | | 0.163508 | | | 0.035615077 | | 5.17E-06 | | 0.072847 | | | 0.072791 | | | 0.316936 | |  |  |  |  |  |  |
| genus.RuminococcaceaeUCG002.id.11360 | | rs77564310 | | A | C | | | -0.0713093 | | | 0.014085525 | | 3.29E-07 | | -0.0035053 | | | 0.028219 | | | 0.901145 | |  |  |  |  |  |  |
| genus.RuminococcaceaeUCG002.id.11360 | | rs79016051 | | C | T | | | -0.0887747 | | | 0.018942419 | | 2.34E-06 | | 0.0571506 | | | 0.033848 | | | 0.0913293 | |  |  |  |  |  |  |
| genus.RuminococcaceaeUCG002.id.11360 | | rs882348 | | A | G | | | -0.0799946 | | | 0.017861803 | | 5.45E-06 | | -0.0333571 | | | 0.03554 | | | 0.347941 | |  |  |  |  |  |  |
| genus.RuminococcaceaeUCG003.id.11361 | | rs10490280 | | C | T | | | -0.0672094 | | | 0.014330937 | | 4.16E-06 | | -0.0029114 | | | 0.02921 | | | 0.920607 | |  |  |  |  |  |  |
| genus.RuminococcaceaeUCG003.id.11361 | | rs11243416 | | T | C | | | -0.0925653 | | | 0.019120311 | | 1.67E-06 | | 0.0084961 | | | 0.045246 | | | 0.851052 | |  |  |  |  |  |  |
| genus.RuminococcaceaeUCG003.id.11361 | | rs11613919 | | G | T | | | 0.0727604 | | | 0.015571648 | | 1.63E-06 | | 0.0065638 | | | 0.027495 | | | 0.811318 | |  |  |  |  |  |  |
| genus.RuminococcaceaeUCG003.id.11361 | | rs139730 | | G | C | | | -0.0577655 | | | 0.013064482 | | 9.72E-06 | | -0.0192212 | | | 0.026472 | | | 0.467777 | |  |  |  |  |  |  |
| genus.RuminococcaceaeUCG003.id.11361 | | rs16959793 | | A | C | | | -0.062527 | | | 0.013125865 | | 2.22E-06 | | 0.00892 | | | 0.023176 | | | 0.700332 | |  |  |  |  |  |  |
| genus.RuminococcaceaeUCG003.id.11361 | | rs2523124 | | T | C | | | -0.0546625 | | | 0.012082341 | | 5.78E-06 | | -0.0189548 | | | 0.023307 | | | 0.416062 | |  |  |  |  |  |  |
| genus.RuminococcaceaeUCG003.id.11361 | | rs3013089 | | G | A | | | -0.0551473 | | | 0.012034599 | | 4.38E-06 | | -0.0112545 | | | 0.023562 | | | 0.632898 | |  |  |  |  |  |  |
| genus.RuminococcaceaeUCG003.id.11361 | | rs4452755 | | A | C | | | -0.0634463 | | | 0.01347408 | | 3.29E-06 | | -0.036478 | | | 0.024172 | | | 0.13128 | |  |  |  |  |  |  |
| genus.RuminococcaceaeUCG003.id.11361 | | rs4532474 | | G | A | | | 0.0769231 | | | 0.017044961 | | 4.82E-06 | | 0.0022955 | | | 0.030915 | | | 0.94081 | |  |  |  |  |  |  |
| genus.RuminococcaceaeUCG003.id.11361 | | rs4629039 | | T | A | | | 0.0553742 | | | 0.012265944 | | 6.54E-06 | | -0.0137559 | | | 0.0235 | | | 0.558309 | |  |  |  |  |  |  |
| genus.RuminococcaceaeUCG003.id.11361 | | rs646327 | | G | A | | | 0.0586696 | | | 0.01183695 | | 7.83E-07 | | 0.0003271 | | | 0.023164 | | | 0.988734 | |  |  |  |  |  |  |
| genus.RuminococcaceaeUCG003.id.11361 | | rs6759615 | | A | G | | | 0.1025228 | | | 0.020020394 | | 7.86E-07 | | -0.0008068 | | | 0.038209 | | | 0.983153 | |  |  |  |  |  |  |
| genus.RuminococcaceaeUCG003.id.11361 | | rs73341549 | | T | C | | | -0.1698572 | | | 0.031876785 | | 1.51E-07 | | -0.0380886 | | | 0.049027 | | | 0.437222 | |  |  |  |  |  |  |
| genus.RuminococcaceaeUCG003.id.11361 | | rs78720113 | | A | G | | | -0.1153484 | | | 0.02496006 | | 7.59E-06 | | 0.0264709 | | | 0.042678 | | | 0.535098 | |  |  |  |  |  |  |
| genus.RuminococcaceaeUCG004.id.11362 | | rs10976229 | | T | G | | | 0.095976 | | | 0.021455386 | | 7.04E-06 | | -0.0221314 | | | 0.034011 | | | 0.515226 | |  |  |  |  |  |  |
| genus.RuminococcaceaeUCG004.id.11362 | | rs11961899 | | G | A | | | -0.0707825 | | | 0.01613814 | | 9.18E-06 | | -0.005829 | | | 0.025479 | | | 0.819045 | |  |  |  |  |  |  |
| genus.RuminococcaceaeUCG004.id.11362 | | rs12125734 | | G | T | | | 0.1339716 | | | 0.02574165 | | 2.09E-07 | | 0.0368549 | | | 0.039476 | | | 0.350515 | |  |  |  |  |  |  |
| genus.RuminococcaceaeUCG004.id.11362 | | rs2248146 | | T | C | | | 0.0689452 | | | 0.015364919 | | 8.20E-06 | | 0.0001208 | | | 0.024031 | | | 0.995991 | |  |  |  |  |  |  |
| genus.RuminococcaceaeUCG004.id.11362 | | rs3800154 | | A | C | | | -0.0797959 | | | 0.017763932 | | 6.12E-06 | | -0.0286789 | | | 0.02604 | | | 0.270749 | |  |  |  |  |  |  |
| genus.RuminococcaceaeUCG004.id.11362 | | rs511258 | | G | A | | | -0.0757474 | | | 0.016247466 | | 4.52E-06 | | -0.0515252 | | | 0.029308 | | | 0.0787336 | |  |  |  |  |  |  |
| genus.RuminococcaceaeUCG004.id.11362 | | rs550351 | | A | C | | | 0.078587 | | | 0.018003041 | | 9.43E-06 | | -0.0021498 | | | 0.023063 | | | 0.925733 | |  |  |  |  |  |  |
| genus.RuminococcaceaeUCG004.id.11362 | | rs6769553 | | A | G | | | 0.0849743 | | | 0.015745058 | | 7.91E-08 | | 0.019547 | | | 0.025956 | | | 0.451396 | |  |  |  |  |  |  |
| genus.RuminococcaceaeUCG004.id.11362 | | rs7123615 | | C | G | | | -0.0786157 | | | 0.017959154 | | 7.09E-06 | | 0.0445704 | | | 0.028866 | | | 0.122572 | |  |  |  |  |  |  |
| genus.RuminococcaceaeUCG004.id.11362 | | rs7569771 | | A | G | | | -0.075914 | | | 0.01703081 | | 8.12E-06 | | 0.026165 | | | 0.026488 | | | 0.323251 | |  |  |  |  |  |  |
| genus.RuminococcaceaeUCG004.id.11362 | | rs872501 | | G | A | | | 0.1161247 | | | 0.025946001 | | 5.81E-06 | | 0.0325665 | | | 0.042453 | | | 0.443004 | |  |  |  |  |  |  |
| genus.RuminococcaceaeUCG004.id.11362 | | rs9818949 | | G | T | | | 0.0859927 | | | 0.018867146 | | 5.39E-06 | | 0.0076987 | | | 0.028808 | | | 0.789283 | |  |  |  |  |  |  |
| genus.RuminococcaceaeUCG005.id.11363 | | rs10873449 | | T | C | | | 0.0654843 | | | 0.01439735 | | 4.11E-06 | | 0.0111218 | | | 0.028931 | | | 0.700666 | |  |  |  |  |  |  |
| genus.RuminococcaceaeUCG005.id.11363 | | rs10937802 | | G | A | | | 0.0755811 | | | 0.016822622 | | 8.17E-06 | | 0.0420611 | | | 0.035066 | | | 0.230344 | |  |  |  |  |  |  |
| genus.RuminococcaceaeUCG005.id.11363 | | rs10950694 | | T | C | | | 0.0577628 | | | 0.011412014 | | 4.30E-07 | | -0.0121356 | | | 0.023832 | | | 0.610597 | |  |  |  |  |  |  |
| genus.RuminococcaceaeUCG005.id.11363 | | rs114279581 | | A | G | | | -0.1466042 | | | 0.031603425 | | 3.22E-06 | | 0.0498027 | | | 0.043043 | | | 0.247253 | |  |  |  |  |  |  |
| genus.RuminococcaceaeUCG005.id.11363 | | rs12288512 | | A | G | | | 0.066635 | | | 0.014435565 | | 3.10E-06 | | 0.0571097 | | | 0.026757 | | | 0.0328118 | |  |  |  |  |  |  |
| genus.RuminococcaceaeUCG005.id.11363 | | rs12458218 | | T | C | | | 0.0677322 | | | 0.014452491 | | 2.41E-06 | | 0.0074719 | | | 0.029652 | | | 0.801049 | |  |  |  |  |  |  |
| genus.RuminococcaceaeUCG005.id.11363 | | rs2893871 | | G | A | | | -0.073644 | | | 0.015547986 | | 3.54E-06 | | 0.010782 | | | 0.036466 | | | 0.767478 | |  |  |  |  |  |  |
| genus.RuminococcaceaeUCG005.id.11363 | | rs34781347 | | G | A | | | 0.1886848 | | | 0.03864836 | | 6.05E-07 | | 0.0278707 | | | 0.044353 | | | 0.529755 | |  |  |  |  |  |  |
| genus.RuminococcaceaeUCG005.id.11363 | | rs35166120 | | C | G | | | -0.0686153 | | | 0.014625203 | | 3.75E-06 | | -0.0634456 | | | 0.027555 | | | 0.0213049 | |  |  |  |  |  |  |
| genus.RuminococcaceaeUCG005.id.11363 | | rs394449 | | A | T | | | 0.0692909 | | | 0.014871206 | | 2.60E-06 | | -0.0548456 | | | 0.034009 | | | 0.106809 | |  |  |  |  |  |  |
| genus.RuminococcaceaeUCG005.id.11363 | | rs55793120 | | T | C | | | 0.1215477 | | | 0.0279546 | | 7.37E-06 | | -0.0916309 | | | 0.048326 | | | 0.0579455 | |  |  |  |  |  |  |
| genus.RuminococcaceaeUCG005.id.11363 | | rs60081663 | | C | G | | | 0.1580815 | | | 0.031958601 | | 9.28E-07 | | -0.0124669 | | | 0.06565 | | | 0.849388 | |  |  |  |  |  |  |
| genus.RuminococcaceaeUCG005.id.11363 | | rs72776570 | | C | A | | | 0.087067 | | | 0.019717899 | | 5.36E-06 | | 0.0538704 | | | 0.03857 | | | 0.162511 | |  |  |  |  |  |  |
| genus.RuminococcaceaeUCG005.id.11363 | | rs7449320 | | C | A | | | 0.0599163 | | | 0.013083635 | | 4.81E-06 | | 0.0033305 | | | 0.027018 | | | 0.901894 | |  |  |  |  |  |  |
| genus.RuminococcaceaeUCG005.id.11363 | | rs7555878 | | A | G | | | 0.0586677 | | | 0.012527736 | | 2.81E-06 | | -0.0127826 | | | 0.026437 | | | 0.628737 | |  |  |  |  |  |  |
| genus.RuminococcaceaeUCG005.id.11363 | | rs7586445 | | G | A | | | 0.0782349 | | | 0.017645763 | | 8.81E-06 | | -0.0073003 | | | 0.034067 | | | 0.830319 | |  |  |  |  |  |  |
| genus.RuminococcaceaeUCG005.id.11363 | | rs898577 | | T | C | | | -0.1230122 | | | 0.028667054 | | 7.46E-06 | | 0.0462723 | | | 0.049246 | | | 0.347415 | |  |  |  |  |  |  |
| genus.RuminococcaceaeUCG009.id.11366 | | rs12508214 | | C | T | | | -0.0774649 | | | 0.016893699 | | 4.75E-06 | | -0.0075526 | | | 0.02434 | | | 0.756336 | |  |  |  |  |  |  |
| genus.RuminococcaceaeUCG009.id.11366 | | rs138460696 | | A | G | | | 0.1392743 | | | 0.031579435 | | 9.81E-06 | | 0.0486599 | | | 0.043512 | | | 0.263435 | |  |  |  |  |  |  |
| genus.RuminococcaceaeUCG009.id.11366 | | rs1550196 | | G | A | | | 0.1308415 | | | 0.026247787 | | 1.13E-06 | | 0.0151537 | | | 0.038187 | | | 0.691489 | |  |  |  |  |  |  |
| genus.RuminococcaceaeUCG009.id.11366 | | rs2058609 | | A | G | | | 0.0816464 | | | 0.017471971 | | 3.12E-06 | | 0.0152849 | | | 0.025566 | | | 0.549931 | |  |  |  |  |  |  |
| genus.RuminococcaceaeUCG009.id.11366 | | rs2192926 | | A | G | | | -0.089048 | | | 0.019298901 | | 4.88E-06 | | 0.002827 | | | 0.024258 | | | 0.907224 | |  |  |  |  |  |  |
| genus.RuminococcaceaeUCG009.id.11366 | | rs4079028 | | C | T | | | 0.0915623 | | | 0.019936034 | | 3.28E-06 | | 0.0341205 | | | 0.026685 | | | 0.201021 | |  |  |  |  |  |  |
| genus.RuminococcaceaeUCG009.id.11366 | | rs4708333 | | T | G | | | -0.084033 | | | 0.017475702 | | 1.56E-06 | | -0.0019907 | | | 0.024096 | | | 0.934158 | |  |  |  |  |  |  |
| genus.RuminococcaceaeUCG009.id.11366 | | rs61779334 | | G | C | | | -0.1381372 | | | 0.029207242 | | 1.94E-06 | | 0.0223688 | | | 0.037912 | | | 0.55518 | |  |  |  |  |  |  |
| genus.RuminococcaceaeUCG009.id.11366 | | rs6952765 | | G | A | | | 0.0732099 | | | 0.016677682 | | 8.13E-06 | | -0.0379451 | | | 0.02455 | | | 0.122197 | |  |  |  |  |  |  |
| genus.RuminococcaceaeUCG009.id.11366 | | rs758191 | | T | G | | | 0.1770195 | | | 0.037511304 | | 9.01E-06 | | -0.0295115 | | | 0.038536 | | | 0.443789 | |  |  |  |  |  |  |
| genus.RuminococcaceaeUCG009.id.11366 | | rs78410648 | | A | G | | | 0.1209889 | | | 0.027731677 | | 9.67E-06 | | 0.0145003 | | | 0.036629 | | | 0.692204 | |  |  |  |  |  |  |
| genus.RuminococcaceaeUCG009.id.11366 | | rs8009993 | | G | C | | | -0.1359362 | | | 0.024485659 | | 4.42E-08 | | 0.0044851 | | | 0.03187 | | | 0.88808 | |  |  |  |  |  |  |
| genus.RuminococcaceaeUCG009.id.11366 | | rs9558661 | | T | C | | | -0.0897823 | | | 0.020073862 | | 7.01E-06 | | -0.0376931 | | | 0.028509 | | | 0.186117 | |  |  |  |  |  |  |
| genus.RuminococcaceaeUCG010.id.11367 | | rs12597105 | | G | A | | | 0.0670855 | | | 0.014441482 | | 4.87E-06 | | -0.055056 | | | 0.029199 | | | 0.0593581 | |  |  |  |  |  |  |
| genus.RuminococcaceaeUCG010.id.11367 | | rs2820282 | | A | C | | | -0.0592304 | | | 0.012591704 | | 2.85E-06 | | 0.0085047 | | | 0.023387 | | | 0.716117 | |  |  |  |  |  |  |
| genus.RuminococcaceaeUCG010.id.11367 | | rs35506912 | | G | C | | | -0.0693743 | | | 0.014792554 | | 3.21E-06 | | 0.0259664 | | | 0.028411 | | | 0.360744 | |  |  |  |  |  |  |
| genus.RuminococcaceaeUCG010.id.11367 | | rs682403 | | A | G | | | -0.0588161 | | | 0.01246713 | | 2.37E-06 | | -0.0190477 | | | 0.022925 | | | 0.406054 | |  |  |  |  |  |  |
| genus.RuminococcaceaeUCG010.id.11367 | | rs6958419 | | C | T | | | -0.0585718 | | | 0.012499376 | | 2.84E-06 | | -0.0196509 | | | 0.022895 | | | 0.390732 | |  |  |  |  |  |  |
| genus.RuminococcaceaeUCG010.id.11367 | | rs73218807 | | G | A | | | -0.1662107 | | | 0.036793834 | | 6.43E-06 | | -0.0160463 | | | 0.041778 | | | 0.700918 | |  |  |  |  |  |  |
| genus.RuminococcaceaeUCG010.id.11367 | | rs7441445 | | C | T | | | -0.05695 | | | 0.012650232 | | 6.80E-06 | | -0.0334132 | | | 0.022903 | | | 0.144591 | |  |  |  |  |  |  |
| genus.RuminococcaceaeUCG010.id.11367 | | rs7935775 | | A | T | | | -0.0631351 | | | 0.013795347 | | 4.99E-06 | | -0.0093258 | | | 0.026029 | | | 0.720132 | |  |  |  |  |  |  |
| genus.RuminococcaceaeUCG011.id.11368 | | rs10274562 | | C | T | | | 0.1109172 | | | 0.024455551 | | 6.50E-06 | | 0.0094442 | | | 0.023478 | | | 0.687489 | |  |  |  |  |  |  |
| genus.RuminococcaceaeUCG011.id.11368 | | rs12636310 | | G | A | | | 0.1327254 | | | 0.028203654 | | 2.81E-06 | | 0.0376069 | | | 0.026352 | | | 0.153543 | |  |  |  |  |  |  |
| genus.RuminococcaceaeUCG011.id.11368 | | rs12724320 | | C | T | | | -0.1208807 | | | 0.024922917 | | 1.52E-06 | | 0.0196214 | | | 0.023439 | | | 0.402512 | |  |  |  |  |  |  |
| genus.RuminococcaceaeUCG011.id.11368 | | rs1416041 | | A | C | | | -0.1823392 | | | 0.033989816 | | 7.04E-08 | | 0.0401266 | | | 0.028129 | | | 0.153715 | |  |  |  |  |  |  |
| genus.RuminococcaceaeUCG011.id.11368 | | rs2729556 | | C | T | | | -0.109097 | | | 0.023369889 | | 3.19E-06 | | -0.0405464 | | | 0.022954 | | | 0.077325 | |  |  |  |  |  |  |
| genus.RuminococcaceaeUCG011.id.11368 | | rs4490371 | | T | C | | | -0.1118163 | | | 0.024896269 | | 7.75E-06 | | -0.0188849 | | | 0.023265 | | | 0.416952 | |  |  |  |  |  |  |
| genus.RuminococcaceaeUCG011.id.11368 | | rs79113084 | | C | T | | | -0.1521655 | | | 0.031751932 | | 2.06E-06 | | 0.0227393 | | | 0.037185 | | | 0.540856 | |  |  |  |  |  |  |
| genus.RuminococcaceaeUCG011.id.11368 | | rs9729514 | | A | G | | | 0.1849335 | | | 0.039461636 | | 2.37E-06 | | 0.0754101 | | | 0.03941 | | | 0.0556839 | |  |  |  |  |  |  |
| genus.RuminococcaceaeUCG013.id.11370 | | rs11581881 | | C | T | | | 0.0661211 | | | 0.014473649 | | 4.73E-06 | | -0.0071717 | | | 0.027215 | | | 0.792146 | |  |  |  |  |  |  |
| genus.RuminococcaceaeUCG013.id.11370 | | rs12189346 | | G | A | | | 0.0684961 | | | 0.014558037 | | 1.68E-06 | | -0.0408355 | | | 0.029173 | | | 0.161584 | |  |  |  |  |  |  |
| genus.RuminococcaceaeUCG013.id.11370 | | rs12336782 | | T | C | | | -0.0856047 | | | 0.018931084 | | 8.60E-06 | | 0.0146607 | | | 0.042621 | | | 0.730863 | |  |  |  |  |  |  |
| genus.RuminococcaceaeUCG013.id.11370 | | rs12485353 | | G | A | | | -0.0607903 | | | 0.013084699 | | 4.19E-06 | | 0.0042925 | | | 0.026303 | | | 0.870367 | |  |  |  |  |  |  |
| genus.RuminococcaceaeUCG013.id.11370 | | rs12781711 | | C | T | | | -0.0656129 | | | 0.011747657 | | 2.55E-08 | | -0.0486923 | | | 0.026387 | | | 0.064986 | |  |  |  |  |  |  |
| genus.RuminococcaceaeUCG013.id.11370 | | rs16918863 | | A | C | | | 0.1114912 | | | 0.024015687 | | 4.16E-06 | | -0.0358388 | | | 0.046877 | | | 0.444552 | |  |  |  |  |  |  |
| genus.RuminococcaceaeUCG013.id.11370 | | rs1729063 | | G | C | | | -0.0533411 | | | 0.01207565 | | 9.64E-06 | | -0.0213283 | | | 0.023959 | | | 0.373358 | |  |  |  |  |  |  |
| genus.RuminococcaceaeUCG013.id.11370 | | rs2428106 | | C | G | | | -0.0490863 | | | 0.010990264 | | 8.38E-06 | | -0.0190541 | | | 0.023126 | | | 0.409977 | |  |  |  |  |  |  |
| genus.RuminococcaceaeUCG013.id.11370 | | rs2730183 | | G | A | | | -0.0488692 | | | 0.010990742 | | 8.44E-06 | | 0.0169077 | | | 0.02332 | | | 0.468425 | |  |  |  |  |  |  |
| genus.RuminococcaceaeUCG013.id.11370 | | rs4385846 | | G | T | | | 0.0598404 | | | 0.013180685 | | 6.46E-06 | | 0.0172081 | | | 0.028946 | | | 0.552183 | |  |  |  |  |  |  |
| genus.RuminococcaceaeUCG013.id.11370 | | rs75088940 | | T | C | | | -0.0942968 | | | 0.02007125 | | 2.55E-06 | | 0.0248654 | | | 0.044745 | | | 0.578409 | |  |  |  |  |  |  |
| genus.RuminococcaceaeUCG013.id.11370 | | rs76973485 | | G | T | | | 0.1949757 | | | 0.041821342 | | 3.35E-06 | | -0.105893 | | | 0.057859 | | | 0.0672202 | |  |  |  |  |  |  |
| genus.RuminococcaceaeUCG013.id.11370 | | rs9313055 | | T | C | | | 0.1050872 | | | 0.023446005 | | 9.55E-06 | | -0.0014968 | | | 0.041563 | | | 0.971271 | |  |  |  |  |  |  |
| genus.RuminococcaceaeUCG013.id.11370 | | rs9565219 | | T | A | | | -0.0524592 | | | 0.011770144 | | 8.73E-06 | | 0.0445907 | | | 0.023213 | | | 0.0547394 | |  |  |  |  |  |  |
| genus.RuminococcaceaeUCG014.id.11371 | | rs10495392 | | C | T | | | -0.082486 | | | 0.018719441 | | 9.96E-06 | | -0.078564 | | | 0.044232 | | | 0.0757042 | |  |  |  |  |  |  |
| genus.RuminococcaceaeUCG014.id.11371 | | rs10791168 | | A | G | | | -0.0664624 | | | 0.015006876 | | 9.76E-06 | | -0.0038189 | | | 0.029633 | | | 0.897457 | |  |  |  |  |  |  |
| genus.RuminococcaceaeUCG014.id.11371 | | rs10941294 | | C | T | | | -0.1220574 | | | 0.026001669 | | 2.40E-06 | | 0.117266 | | | 0.050821 | | | 0.0210296 | |  |  |  |  |  |  |
| genus.RuminococcaceaeUCG014.id.11371 | | rs115777838 | | T | C | | | -0.1883495 | | | 0.038664295 | | 4.62E-07 | | 0.0315586 | | | 0.036348 | | | 0.385261 | |  |  |  |  |  |  |
| genus.RuminococcaceaeUCG014.id.11371 | | rs12638134 | | T | G | | | 0.0582548 | | | 0.011965732 | | 1.21E-06 | | -0.0536566 | | | 0.023075 | | | 0.0200549 | |  |  |  |  |  |  |
| genus.RuminococcaceaeUCG014.id.11371 | | rs17296933 | | C | G | | | -0.0829544 | | | 0.018505763 | | 7.34E-06 | | 0.0559935 | | | 0.036071 | | | 0.120587 | |  |  |  |  |  |  |
| genus.RuminococcaceaeUCG014.id.11371 | | rs34402072 | | C | T | | | -0.0688023 | | | 0.015608285 | | 9.80E-06 | | 0.0496953 | | | 0.032352 | | | 0.124522 | |  |  |  |  |  |  |
| genus.RuminococcaceaeUCG014.id.11371 | | rs439810 | | G | C | | | -0.0577127 | | | 0.012667538 | | 7.04E-06 | | 0.0202334 | | | 0.024253 | | | 0.404122 | |  |  |  |  |  |  |
| genus.RuminococcaceaeUCG014.id.11371 | | rs56105232 | | G | A | | | 0.1392757 | | | 0.029913177 | | 2.91E-06 | | 0.0884526 | | | 0.047859 | | | 0.0645743 | |  |  |  |  |  |  |
| genus.RuminococcaceaeUCG014.id.11371 | | rs61898819 | | A | T | | | 0.0607865 | | | 0.01387607 | | 9.92E-06 | | 0.0447827 | | | 0.028915 | | | 0.121435 | |  |  |  |  |  |  |
| genus.RuminococcaceaeUCG014.id.11371 | | rs62478832 | | T | A | | | -0.058129 | | | 0.012903031 | | 6.04E-06 | | 0.0270713 | | | 0.024054 | | | 0.260401 | |  |  |  |  |  |  |
| genus.RuminococcaceaeUCG014.id.11371 | | rs72809222 | | T | C | | | 0.0671775 | | | 0.013983796 | | 2.41E-06 | | 0.0409928 | | | 0.028043 | | | 0.143805 | |  |  |  |  |  |  |
| genus.RuminococcaceaeUCG014.id.11371 | | rs74060145 | | C | G | | | -0.1157668 | | | 0.025419855 | | 8.71E-06 | | -0.0223988 | | | 0.042444 | | | 0.597689 | |  |  |  |  |  |  |
| genus.RuminococcaceaeUCG014.id.11371 | | rs79640386 | | T | A | | | -0.1108635 | | | 0.024809787 | | 8.74E-06 | | 0.0549369 | | | 0.033711 | | | 0.103174 | |  |  |  |  |  |  |
| genus.RuminococcaceaeUCG014.id.11371 | | rs853612 | | A | G | | | -0.0528257 | | | 0.011936668 | | 9.75E-06 | | 0.0026688 | | | 0.023367 | | | 0.909071 | |  |  |  |  |  |  |
| genus.RuminococcaceaeUCG014.id.11371 | | rs995642 | | C | T | | | 0.060048 | | | 0.012641687 | | 1.90E-06 | | 0.0021928 | | | 0.026759 | | | 0.93469 | |  |  |  |  |  |  |
| genus.Ruminococcus1.id.11373 | | rs10167839 | | A | G | | | 0.0519509 | | | 0.011630648 | | 8.09E-06 | | -0.0170572 | | | 0.02383 | | | 0.474119 | |  |  |  |  |  |  |
| genus.Ruminococcus1.id.11373 | | rs10769159 | | G | C | | | -0.0640277 | | | 0.011005922 | | 5.29E-09 | | -0.0186709 | | | 0.023307 | | | 0.423091 | |  |  |  |  |  |  |
| genus.Ruminococcus1.id.11373 | | rs10995816 | | C | G | | | -0.0762002 | | | 0.017468886 | | 8.38E-06 | | 0.0520374 | | | 0.036237 | | | 0.150991 | |  |  |  |  |  |  |
| genus.Ruminococcus1.id.11373 | | rs11783695 | | G | T | | | -0.0734114 | | | 0.016139812 | | 4.73E-06 | | -0.0130206 | | | 0.031235 | | | 0.676779 | |  |  |  |  |  |  |
| genus.Ruminococcus1.id.11373 | | rs17781867 | | C | T | | | 0.0999271 | | | 0.021172816 | | 1.96E-06 | | 0.02425 | | | 0.045113 | | | 0.590895 | |  |  |  |  |  |  |
| genus.Ruminococcus1.id.11373 | | rs3000856 | | T | A | | | -0.0709997 | | | 0.016276599 | | 9.28E-06 | | -0.0137473 | | | 0.030991 | | | 0.657338 | |  |  |  |  |  |  |
| genus.Ruminococcus1.id.11373 | | rs3819978 | | C | T | | | -0.1150398 | | | 0.026013681 | | 8.74E-06 | | -0.0434805 | | | 0.043161 | | | 0.313742 | |  |  |  |  |  |  |
| genus.Ruminococcus1.id.11373 | | rs4849717 | | T | A | | | 0.1327638 | | | 0.029981613 | | 8.83E-06 | | 0.0599683 | | | 0.04363 | | | 0.169298 | |  |  |  |  |  |  |
| genus.Ruminococcus1.id.11373 | | rs6105066 | | T | C | | | -0.0607052 | | | 0.01342499 | | 5.06E-06 | | -0.001462 | | | 0.025713 | | | 0.954657 | |  |  |  |  |  |  |
| genus.Ruminococcus1.id.11373 | | rs6493760 | | C | T | | | 0.0535262 | | | 0.011588457 | | 3.38E-06 | | -0.0440257 | | | 0.023898 | | | 0.065438 | |  |  |  |  |  |  |
| genus.Ruminococcus1.id.11373 | | rs7117576 | | A | G | | | 0.0829505 | | | 0.017089133 | | 6.48E-07 | | -0.0553703 | | | 0.039886 | | | 0.165071 | |  |  |  |  |  |  |
| genus.Ruminococcus1.id.11373 | | rs7583465 | | C | T | | | 0.0527654 | | | 0.011261892 | | 2.56E-06 | | -0.0102767 | | | 0.022936 | | | 0.654103 | |  |  |  |  |  |  |
| genus.Ruminococcus1.id.11373 | | rs78572139 | | G | A | | | 0.1250377 | | | 0.027941129 | | 5.23E-06 | | 0.0191409 | | | 0.037879 | | | 0.613333 | |  |  |  |  |  |  |
| genus.Ruminococcus1.id.11373 | | rs78613526 | | G | A | | | 0.1674966 | | | 0.036763917 | | 5.11E-06 | | 0.002621 | | | 0.051928 | | | 0.959746 | |  |  |  |  |  |  |
| genus.Ruminococcus2.id.11374 | | rs12406309 | | A | C | | | -0.063193 | | | 0.014227548 | | 9.79E-06 | | -0.0470016 | | | 0.02757 | | | 0.0882267 | |  |  |  |  |  |  |
| genus.Ruminococcus2.id.11374 | | rs12986628 | | C | T | | | 0.0665937 | | | 0.014016808 | | 2.14E-06 | | 0.0528732 | | | 0.028913 | | | 0.0674404 | |  |  |  |  |  |  |
| genus.Ruminococcus2.id.11374 | | rs1819812 | | G | T | | | 0.0842356 | | | 0.018489341 | | 5.28E-06 | | 0.0169564 | | | 0.050763 | | | 0.738355 | |  |  |  |  |  |  |
| genus.Ruminococcus2.id.11374 | | rs2368224 | | T | G | | | 0.1996476 | | | 0.043845344 | | 3.63E-06 | | 0.0050823 | | | 0.052663 | | | 0.923118 | |  |  |  |  |  |  |
| genus.Ruminococcus2.id.11374 | | rs2846589 | | G | T | | | 0.0522093 | | | 0.011638161 | | 7.59E-06 | | -0.0300981 | | | 0.023348 | | | 0.197358 | |  |  |  |  |  |  |
| genus.Ruminococcus2.id.11374 | | rs2997412 | | A | G | | | -0.0568439 | | | 0.012232461 | | 4.22E-06 | | -0.0093782 | | | 0.025579 | | | 0.713891 | |  |  |  |  |  |  |
| genus.Ruminococcus2.id.11374 | | rs4400279 | | A | G | | | 0.0546114 | | | 0.012007747 | | 5.80E-06 | | -0.0226898 | | | 0.024365 | | | 0.351718 | |  |  |  |  |  |  |
| genus.Ruminococcus2.id.11374 | | rs4799823 | | C | T | | | 0.0837001 | | | 0.018226258 | | 5.40E-06 | | 0.0184567 | | | 0.029642 | | | 0.533508 | |  |  |  |  |  |  |
| genus.Ruminococcus2.id.11374 | | rs55707116 | | C | A | | | 0.0865521 | | | 0.01891409 | | 8.01E-06 | | -0.0180231 | | | 0.043547 | | | 0.678961 | |  |  |  |  |  |  |
| genus.Ruminococcus2.id.11374 | | rs58681734 | | A | G | | | 0.0723875 | | | 0.016078702 | | 4.18E-06 | | -0.0167278 | | | 0.028038 | | | 0.55077 | |  |  |  |  |  |  |
| genus.Ruminococcus2.id.11374 | | rs61791565 | | T | C | | | -0.0523503 | | | 0.011711882 | | 6.79E-06 | | 0.0023895 | | | 0.022887 | | | 0.916848 | |  |  |  |  |  |  |
| genus.Ruminococcus2.id.11374 | | rs75140805 | | T | G | | | 0.0836825 | | | 0.017644509 | | 3.95E-06 | | -0.0220327 | | | 0.030006 | | | 0.462779 | |  |  |  |  |  |  |
| genus.Ruminococcus2.id.11374 | | rs7635831 | | G | A | | | 0.0618344 | | | 0.012903108 | | 1.98E-06 | | -0.0220296 | | | 0.023762 | | | 0.353885 | |  |  |  |  |  |  |
| genus.Ruminococcus2.id.11374 | | rs7693984 | | G | A | | | -0.1028218 | | | 0.023508663 | | 9.42E-06 | | 0.0831624 | | | 0.055714 | | | 0.135526 | |  |  |  |  |  |  |
| genus.Ruminococcus2.id.11374 | | rs78120384 | | A | G | | | -0.1928155 | | | 0.039204507 | | 3.31E-07 | | 0.0288606 | | | 0.039347 | | | 0.463262 | |  |  |  |  |  |  |
| genus.Sellimonas.id.14369 | | rs113379006 | | T | C | | | -0.1627588 | | | 0.035702495 | | 7.21E-06 | | -0.0143964 | | | 0.030065 | | | 0.632048 | |  |  |  |  |  |  |
| genus.Sellimonas.id.14369 | | rs13417181 | | T | C | | | 0.1665093 | | | 0.033752138 | | 7.62E-07 | | -0.0455866 | | | 0.026961 | | | 0.0908657 | |  |  |  |  |  |  |
| genus.Sellimonas.id.14369 | | rs17657328 | | C | T | | | 0.1145714 | | | 0.025361755 | | 8.25E-06 | | -0.0155527 | | | 0.023396 | | | 0.506211 | |  |  |  |  |  |  |
| genus.Sellimonas.id.14369 | | rs2016057 | | A | C | | | -0.1258775 | | | 0.02561532 | | 1.03E-06 | | 0.0269096 | | | 0.023367 | | | 0.24949 | |  |  |  |  |  |  |
| genus.Sellimonas.id.14369 | | rs2187447 | | A | C | | | 0.2434571 | | | 0.052769543 | | 3.98E-06 | | -0.0460553 | | | 0.047903 | | | 0.336332 | |  |  |  |  |  |  |
| genus.Sellimonas.id.14369 | | rs2371572 | | A | C | | | 0.1273489 | | | 0.025086238 | | 4.46E-07 | | -0.0100527 | | | 0.02297 | | | 0.66164 | |  |  |  |  |  |  |
| genus.Sellimonas.id.14369 | | rs41816 | | A | G | | | 0.1322012 | | | 0.029108726 | | 8.39E-06 | | -0.013298 | | | 0.024907 | | | 0.593413 | |  |  |  |  |  |  |
| genus.Sellimonas.id.14369 | | rs553697 | | T | C | | | -0.1537673 | | | 0.033909892 | | 6.13E-06 | | 0.0219809 | | | 0.029486 | | | 0.45599 | |  |  |  |  |  |  |
| genus.Sellimonas.id.14369 | | rs56203279 | | T | C | | | -0.124045 | | | 0.026911497 | | 3.72E-06 | | -0.0188905 | | | 0.024081 | | | 0.432765 | |  |  |  |  |  |  |
| genus.Sellimonas.id.14369 | | rs72553859 | | G | C | | | -0.1504581 | | | 0.033262641 | | 5.38E-06 | | 0.0159873 | | | 0.031252 | | | 0.608955 | |  |  |  |  |  |  |
| genus.Senegalimassilia.id.11160 | | rs10036909 | | C | T | | | 0.1855194 | | | 0.040088092 | | 8.05E-06 | | -0.0073337 | | | 0.057738 | | | 0.898928 | |  |  |  |  |  |  |
| genus.Senegalimassilia.id.11160 | | rs11787826 | | C | A | | | 0.081327 | | | 0.017115415 | | 2.63E-06 | | 0.0385359 | | | 0.023166 | | | 0.096221 | |  |  |  |  |  |  |
| genus.Senegalimassilia.id.11160 | | rs13383270 | | G | C | | | 0.0774679 | | | 0.017084039 | | 6.04E-06 | | 0.0042541 | | | 0.022897 | | | 0.852606 | |  |  |  |  |  |  |
| genus.Senegalimassilia.id.11160 | | rs1990708 | | A | C | | | -0.1096195 | | | 0.024778524 | | 8.91E-06 | | 0.012394 | | | 0.042657 | | | 0.771394 | |  |  |  |  |  |  |
| genus.Senegalimassilia.id.11160 | | rs2017373 | | C | T | | | 0.0782258 | | | 0.017684094 | | 9.50E-06 | | -0.0346992 | | | 0.023833 | | | 0.145409 | |  |  |  |  |  |  |
| genus.Senegalimassilia.id.11160 | | rs57512504 | | T | A | | | 0.0819494 | | | 0.017224097 | | 2.03E-06 | | 0.0260062 | | | 0.023011 | | | 0.258416 | |  |  |  |  |  |  |
| genus.Senegalimassilia.id.11160 | | rs7225245 | | G | A | | | 0.079173 | | | 0.017042101 | | 4.18E-06 | | 0.0044941 | | | 0.023147 | | | 0.846052 | |  |  |  |  |  |  |
| genus.Senegalimassilia.id.11160 | | rs72887800 | | T | A | | | -0.0822462 | | | 0.017572066 | | 2.42E-06 | | -0.0120859 | | | 0.023268 | | | 0.603473 | |  |  |  |  |  |  |
| genus.Slackia.id.825 | | rs10409783 | | A | G | | | 0.0950828 | | | 0.021123946 | | 7.70E-06 | | 0.0090494 | | | 0.025326 | | | 0.720855 | |  |  |  |  |  |  |
| genus.Slackia.id.825 | | rs112764253 | | T | A | | | 0.1947135 | | | 0.041159784 | | 3.40E-06 | | -0.0451753 | | | 0.067254 | | | 0.501767 | |  |  |  |  |  |  |
| genus.Slackia.id.825 | | rs12440440 | | A | G | | | 0.0901934 | | | 0.019058248 | | 2.63E-06 | | -0.0498555 | | | 0.024051 | | | 0.0381768 | |  |  |  |  |  |  |
| genus.Slackia.id.825 | | rs13339230 | | C | G | | | 0.1470548 | | | 0.033063509 | | 7.42E-06 | | 0.0344521 | | | 0.040858 | | | 0.399104 | |  |  |  |  |  |  |
| genus.Slackia.id.825 | | rs16894137 | | C | T | | | -0.1227921 | | | 0.026304664 | | 2.71E-06 | | 0.0079656 | | | 0.033856 | | | 0.813991 | |  |  |  |  |  |  |
| genus.Slackia.id.825 | | rs35156985 | | T | C | | | -0.1557089 | | | 0.034808468 | | 8.06E-06 | | -0.0505015 | | | 0.057547 | | | 0.380178 | |  |  |  |  |  |  |
| genus.Slackia.id.825 | | rs4492265 | | A | G | | | -0.0905757 | | | 0.019165844 | | 2.41E-06 | | -0.0495756 | | | 0.0249 | | | 0.0464826 | |  |  |  |  |  |  |
| genus.Slackia.id.825 | | rs58767323 | | G | C | | | -0.1028334 | | | 0.022707713 | | 4.60E-06 | | -0.0150199 | | | 0.029882 | | | 0.615217 | |  |  |  |  |  |  |
| genus.Slackia.id.825 | | rs8901 | | C | T | | | 0.0934594 | | | 0.018681296 | | 6.07E-07 | | 0.0034783 | | | 0.025117 | | | 0.889858 | |  |  |  |  |  |  |
| genus.Streptococcus.id.1853 | | rs10028567 | | C | T | | | -0.0921167 | | | 0.019188114 | | 7.30E-06 | | 0.0062635 | | | 0.035001 | | | 0.857978 | |  |  |  |  |  |  |
| genus.Streptococcus.id.1853 | | rs10448310 | | A | G | | | -0.0517935 | | | 0.011132356 | | 3.31E-06 | | 0.0342484 | | | 0.023815 | | | 0.150403 | |  |  |  |  |  |  |
| genus.Streptococcus.id.1853 | | rs11110281 | | T | C | | | -0.1375189 | | | 0.022739814 | | 2.58E-09 | | -0.0599131 | | | 0.053089 | | | 0.259088 | |  |  |  |  |  |  |
| genus.Streptococcus.id.1853 | | rs11720390 | | G | A | | | 0.1070239 | | | 0.022812145 | | 3.59E-06 | | -0.0643003 | | | 0.047354 | | | 0.174502 | |  |  |  |  |  |  |
| genus.Streptococcus.id.1853 | | rs11764382 | | A | G | | | -0.0695345 | | | 0.014367132 | | 1.29E-06 | | -0.0177913 | | | 0.032839 | | | 0.587979 | |  |  |  |  |  |  |
| genus.Streptococcus.id.1853 | | rs17708276 | | A | G | | | -0.0793955 | | | 0.017062753 | | 3.04E-06 | | -0.026953 | | | 0.036889 | | | 0.464987 | |  |  |  |  |  |  |
| genus.Streptococcus.id.1853 | | rs1918540 | | G | A | | | 0.059639 | | | 0.012814811 | | 2.44E-06 | | -0.0062515 | | | 0.029709 | | | 0.833338 | |  |  |  |  |  |  |
| genus.Streptococcus.id.1853 | | rs2370083 | | G | T | | | -0.0816836 | | | 0.018585095 | | 9.75E-06 | | 0.0399429 | | | 0.046885 | | | 0.394249 | |  |  |  |  |  |  |
| genus.Streptococcus.id.1853 | | rs395407 | | G | C | | | -0.0792781 | | | 0.01736973 | | 4.37E-06 | | 0.070784 | | | 0.03153 | | | 0.0247697 | |  |  |  |  |  |  |
| genus.Streptococcus.id.1853 | | rs57646748 | | G | A | | | -0.0907696 | | | 0.020034438 | | 5.48E-06 | | -0.0328985 | | | 0.059249 | | | 0.578719 | |  |  |  |  |  |  |
| genus.Streptococcus.id.1853 | | rs6563952 | | G | C | | | 0.0827344 | | | 0.018003533 | | 5.82E-06 | | 0.0095327 | | | 0.039564 | | | 0.8096 | |  |  |  |  |  |  |
| genus.Streptococcus.id.1853 | | rs6806351 | | T | C | | | -0.0633829 | | | 0.013664741 | | 4.94E-06 | | -0.0373989 | | | 0.027587 | | | 0.175196 | |  |  |  |  |  |  |
| genus.Streptococcus.id.1853 | | rs71481756 | | T | G | | | 0.0931048 | | | 0.020794918 | | 6.51E-06 | | -0.063833 | | | 0.046462 | | | 0.169484 | |  |  |  |  |  |  |
| genus.Streptococcus.id.1853 | | rs77558518 | | A | G | | | -0.1039992 | | | 0.022971364 | | 4.71E-06 | | -0.0163393 | | | 0.038801 | | | 0.673682 | |  |  |  |  |  |  |
| genus.Streptococcus.id.1853 | | rs7916711 | | A | G | | | 0.1028909 | | | 0.021736227 | | 2.72E-06 | | 0.0679582 | | | 0.032859 | | | 0.0386225 | |  |  |  |  |  |  |
| genus.Streptococcus.id.1853 | | rs8191642 | | G | A | | | -0.0604189 | | | 0.013670956 | | 8.09E-06 | | 0.0059406 | | | 0.03143 | | | 0.850086 | |  |  |  |  |  |  |
| genus.Subdoligranulum.id.2070 | | rs10065321 | | T | C | | | -0.0512828 | | | 0.010810523 | | 2.10E-06 | | 0.0020837 | | | 0.023135 | | | 0.928233 | |  |  |  |  |  |  |
| genus.Subdoligranulum.id.2070 | | rs10497836 | | C | T | | | -0.0524065 | | | 0.011869733 | | 8.38E-06 | | 0.0192658 | | | 0.027931 | | | 0.49035 | |  |  |  |  |  |  |
| genus.Subdoligranulum.id.2070 | | rs12638227 | | G | C | | | -0.0557288 | | | 0.010855202 | | 2.48E-07 | | 0.037454 | | | 0.02328 | | | 0.107644 | |  |  |  |  |  |  |
| genus.Subdoligranulum.id.2070 | | rs1667315 | | G | A | | | 0.0485017 | | | 0.010745207 | | 6.72E-06 | | 0.0312811 | | | 0.023374 | | | 0.180796 | |  |  |  |  |  |  |
| genus.Subdoligranulum.id.2070 | | rs16962433 | | A | T | | | 0.0855999 | | | 0.018921315 | | 7.65E-06 | | 0.0003056 | | | 0.047119 | | | 0.994826 | |  |  |  |  |  |  |
| genus.Subdoligranulum.id.2070 | | rs2114677 | | C | T | | | -0.1041763 | | | 0.023082878 | | 2.72E-06 | | -0.0152493 | | | 0.035744 | | | 0.66965 | |  |  |  |  |  |  |
| genus.Subdoligranulum.id.2070 | | rs2171249 | | C | T | | | 0.1067437 | | | 0.023321252 | | 4.51E-06 | | -0.0149449 | | | 0.044457 | | | 0.736745 | |  |  |  |  |  |  |
| genus.Subdoligranulum.id.2070 | | rs35940633 | | G | A | | | -0.0511008 | | | 0.011001029 | | 4.22E-06 | | 0.0098515 | | | 0.024323 | | | 0.685455 | |  |  |  |  |  |  |
| genus.Subdoligranulum.id.2070 | | rs3761728 | | T | G | | | -0.0543458 | | | 0.011886608 | | 3.87E-06 | | -0.0144024 | | | 0.026314 | | | 0.584154 | |  |  |  |  |  |  |
| genus.Subdoligranulum.id.2070 | | rs4347804 | | A | G | | | 0.1660632 | | | 0.035748326 | | 2.18E-06 | | 0.13667 | | | 0.064159 | | | 0.0331558 | |  |  |  |  |  |  |
| genus.Subdoligranulum.id.2070 | | rs6555306 | | T | C | | | -0.0740777 | | | 0.015546287 | | 2.81E-06 | | 0.0228035 | | | 0.032669 | | | 0.485161 | |  |  |  |  |  |  |
| genus.Subdoligranulum.id.2070 | | rs75158211 | | T | C | | | -0.072343 | | | 0.015933044 | | 7.52E-06 | | 0.0420345 | | | 0.03224 | | | 0.192306 | |  |  |  |  |  |  |
| genus.Subdoligranulum.id.2070 | | rs76528319 | | G | T | | | -0.1432786 | | | 0.031066225 | | 7.41E-06 | | 0.050946 | | | 0.041336 | | | 0.217763 | |  |  |  |  |  |  |
| genus.Subdoligranulum.id.2070 | | rs76664262 | | T | A | | | 0.0834078 | | | 0.018535669 | | 4.87E-06 | | -0.0116042 | | | 0.036446 | | | 0.750184 | |  |  |  |  |  |  |
| genus.Sutterella.id.2896 | | rs1145877 | | A | G | | | -0.073548 | | | 0.016241333 | | 7.20E-06 | | 0.0108806 | | | 0.033188 | | | 0.743029 | |  |  |  |  |  |  |
| genus.Sutterella.id.2896 | | rs11591622 | | T | G | | | -0.0688382 | | | 0.015137492 | | 6.50E-06 | | 0.0143024 | | | 0.031179 | | | 0.646434 | |  |  |  |  |  |  |
| genus.Sutterella.id.2896 | | rs13173038 | | A | G | | | -0.0718076 | | | 0.015162698 | | 2.73E-06 | | -0.0101981 | | | 0.026385 | | | 0.699116 | |  |  |  |  |  |  |
| genus.Sutterella.id.2896 | | rs143438747 | | T | C | | | -0.1457927 | | | 0.030686706 | | 3.28E-06 | | 0.0114482 | | | 0.043026 | | | 0.790182 | |  |  |  |  |  |  |
| genus.Sutterella.id.2896 | | rs2050185 | | G | A | | | 0.0575138 | | | 0.012876636 | | 7.97E-06 | | -0.0302782 | | | 0.023634 | | | 0.200156 | |  |  |  |  |  |  |
| genus.Sutterella.id.2896 | | rs2321387 | | G | A | | | -0.0592876 | | | 0.012450922 | | 1.87E-06 | | 0.005982 | | | 0.023064 | | | 0.795351 | |  |  |  |  |  |  |
| genus.Sutterella.id.2896 | | rs2613606 | | C | T | | | -0.0556794 | | | 0.012411747 | | 7.20E-06 | | -0.0374331 | | | 0.023242 | | | 0.107271 | |  |  |  |  |  |  |
| genus.Sutterella.id.2896 | | rs607327 | | C | T | | | 0.0578334 | | | 0.012905264 | | 6.63E-06 | | 0.0216179 | | | 0.023518 | | | 0.357994 | |  |  |  |  |  |  |
| genus.Sutterella.id.2896 | | rs62501473 | | G | A | | | 0.0694233 | | | 0.014941649 | | 5.52E-06 | | 0.0035696 | | | 0.026007 | | | 0.890829 | |  |  |  |  |  |  |
| genus.Sutterella.id.2896 | | rs7499539 | | A | G | | | 0.0617478 | | | 0.013099845 | | 2.36E-06 | | 0.0031557 | | | 0.025904 | | | 0.90304 | |  |  |  |  |  |  |
| genus.Sutterella.id.2896 | | rs7638039 | | T | C | | | 0.0645624 | | | 0.014388651 | | 8.66E-06 | | 0.026145 | | | 0.02654 | | | 0.32457 | |  |  |  |  |  |  |
| genus.Sutterella.id.2896 | | rs9350083 | | T | G | | | -0.0593069 | | | 0.013392944 | | 8.23E-06 | | -0.0192179 | | | 0.023844 | | | 0.420253 | |  |  |  |  |  |  |
| genus.Terrisporobacter.id.11348 | | rs1883097 | | C | T | | | 0.2263988 | | | 0.045464179 | | 4.16E-07 | | 0.0311059 | | | 0.05988 | | | 0.603433 | |  |  |  |  |  |  |
| genus.Terrisporobacter.id.11348 | | rs2569953 | | A | C | | | -0.0775591 | | | 0.01746408 | | 8.95E-06 | | -0.0107325 | | | 0.023127 | | | 0.642592 | |  |  |  |  |  |  |
| genus.Terrisporobacter.id.11348 | | rs2872237 | | C | A | | | -0.0814505 | | | 0.017594312 | | 3.97E-06 | | 0.0798185 | | | 0.023114 | | | 0.00055376 | |  |  |  |  |  |  |
| genus.Terrisporobacter.id.11348 | | rs58405430 | | G | T | | | 0.1346172 | | | 0.030117736 | | 7.94E-06 | | 0.0415537 | | | 0.048144 | | | 0.388071 | |  |  |  |  |  |  |
| genus.Terrisporobacter.id.11348 | | rs7034891 | | G | C | | | -0.0799209 | | | 0.01737936 | | 4.54E-06 | | -0.0396428 | | | 0.023373 | | | 0.0898669 | |  |  |  |  |  |  |
| genus.Terrisporobacter.id.11348 | | rs7184125 | | T | C | | | 0.091256 | | | 0.020549332 | | 8.48E-06 | | -0.0424638 | | | 0.02553 | | | 0.0962543 | |  |  |  |  |  |  |
| genus.Turicibacter.id.2162 | | rs11054680 | | T | C | | | -0.1047512 | | | 0.02269975 | | 2.31E-06 | | -0.0376286 | | | 0.030342 | | | 0.214916 | |  |  |  |  |  |  |
| genus.Turicibacter.id.2162 | | rs11649454 | | G | C | | | 0.0950891 | | | 0.020343331 | | 3.27E-06 | | 0.0295741 | | | 0.031803 | | | 0.352407 | |  |  |  |  |  |  |
| genus.Turicibacter.id.2162 | | rs11666533 | | C | T | | | -0.1116891 | | | 0.024843618 | | 7.37E-06 | | -0.0492865 | | | 0.042339 | | | 0.244392 | |  |  |  |  |  |  |
| genus.Turicibacter.id.2162 | | rs149744580 | | A | G | | | 0.1698827 | | | 0.031547837 | | 7.01E-08 | | 0.0847729 | | | 0.050845 | | | 0.0954597 | |  |  |  |  |  |  |
| genus.Turicibacter.id.2162 | | rs2221441 | | G | C | | | 0.0710364 | | | 0.015343033 | | 3.46E-06 | | 0.0060368 | | | 0.022956 | | | 0.792576 | |  |  |  |  |  |  |
| genus.Turicibacter.id.2162 | | rs2834977 | | T | C | | | -0.0959995 | | | 0.020826107 | | 3.96E-06 | | -0.0456963 | | | 0.031861 | | | 0.151505 | |  |  |  |  |  |  |
| genus.Turicibacter.id.2162 | | rs2952020 | | G | A | | | -0.0759019 | | | 0.016576375 | | 5.63E-06 | | -0.0024982 | | | 0.026635 | | | 0.925273 | |  |  |  |  |  |  |
| genus.Turicibacter.id.2162 | | rs3734633 | | G | A | | | -0.1209568 | | | 0.026830036 | | 5.32E-06 | | -0.000493 | | | 0.048808 | | | 0.991941 | |  |  |  |  |  |  |
| genus.Turicibacter.id.2162 | | rs4247078 | | C | G | | | 0.0710377 | | | 0.015522123 | | 5.46E-06 | | -0.0276843 | | | 0.023348 | | | 0.235723 | |  |  |  |  |  |  |
| genus.Turicibacter.id.2162 | | rs4869133 | | G | A | | | 0.1311861 | | | 0.027196968 | | 2.55E-06 | | -0.0066974 | | | 0.030083 | | | 0.823821 | |  |  |  |  |  |  |
| genus.Turicibacter.id.2162 | | rs55756211 | | T | C | | | -0.1151152 | | | 0.024070764 | | 2.81E-06 | | 0.068537 | | | 0.044095 | | | 0.120107 | |  |  |  |  |  |  |
| genus.Turicibacter.id.2162 | | rs61265175 | | G | C | | | -0.0858591 | | | 0.018577761 | | 4.14E-06 | | 0.0008029 | | | 0.029549 | | | 0.978323 | |  |  |  |  |  |  |
| genus.Turicibacter.id.2162 | | rs7199484 | | G | A | | | -0.0731428 | | | 0.016017166 | | 5.77E-06 | | 0.0116345 | | | 0.024742 | | | 0.638193 | |  |  |  |  |  |  |
| genus.Tyzzerella3.id.11335 | | rs10898797 | | C | T | | | 0.1223803 | | | 0.027468252 | | 8.85E-06 | | 0.052529 | | | 0.036434 | | | 0.149371 | |  |  |  |  |  |  |
| genus.Tyzzerella3.id.11335 | | rs112102233 | | A | G | | | -0.2163484 | | | 0.04775752 | | 6.18E-06 | | -0.00455 | | | 0.053955 | | | 0.932795 | |  |  |  |  |  |  |
| genus.Tyzzerella3.id.11335 | | rs1232220 | | G | T | | | -0.1438693 | | | 0.031828041 | | 7.91E-06 | | -0.0069076 | | | 0.037964 | | | 0.85562 | |  |  |  |  |  |  |
| genus.Tyzzerella3.id.11335 | | rs17706273 | | T | C | | | -0.1403684 | | | 0.027470793 | | 5.88E-07 | | 0.0134274 | | | 0.042531 | | | 0.75222 | |  |  |  |  |  |  |
| genus.Tyzzerella3.id.11335 | | rs17809157 | | A | T | | | -0.1638195 | | | 0.033638641 | | 1.54E-06 | | -0.0638627 | | | 0.036298 | | | 0.0785073 | |  |  |  |  |  |  |
| genus.Tyzzerella3.id.11335 | | rs191093 | | G | A | | | 0.1590081 | | | 0.035330566 | | 6.76E-06 | | 0.0147078 | | | 0.037325 | | | 0.693542 | |  |  |  |  |  |  |
| genus.Tyzzerella3.id.11335 | | rs4904512 | | T | C | | | -0.1171509 | | | 0.025030561 | | 3.09E-06 | | 0.0231744 | | | 0.033983 | | | 0.495276 | |  |  |  |  |  |  |
| genus.Tyzzerella3.id.11335 | | rs55799124 | | A | G | | | -0.1143501 | | | 0.023860417 | | 1.34E-06 | | -0.0088652 | | | 0.025995 | | | 0.733079 | |  |  |  |  |  |  |
| genus.Tyzzerella3.id.11335 | | rs67476743 | | T | G | | | 0.1321636 | | | 0.02220797 | | 3.74E-09 | | 0.0323595 | | | 0.025798 | | | 0.209718 | |  |  |  |  |  |  |
| genus.Tyzzerella3.id.11335 | | rs7019909 | | T | C | | | 0.1441555 | | | 0.030162645 | | 1.76E-06 | | 0.0096768 | | | 0.035041 | | | 0.782429 | |  |  |  |  |  |  |
| genus.Tyzzerella3.id.11335 | | rs7333521 | | T | C | | | -0.2071915 | | | 0.045312058 | | 4.88E-06 | | -0.0893917 | | | 0.064869 | | | 0.16819 | |  |  |  |  |  |  |
| genus.Tyzzerella3.id.11335 | | rs75091807 | | G | T | | | -0.1849662 | | | 0.038302914 | | 1.71E-06 | | 0.0032633 | | | 0.047572 | | | 0.94531 | |  |  |  |  |  |  |
| genus.Tyzzerella3.id.11335 | | rs7561370 | | T | C | | | 0.1313411 | | | 0.028629245 | | 1.52E-06 | | 0.0375392 | | | 0.031935 | | | 0.239794 | |  |  |  |  |  |  |
| genus.Veillonella.id.2198 | | rs12679709 | | C | G | | | -0.0793499 | | | 0.016481776 | | 1.78E-06 | | 0.0174534 | | | 0.02383 | | | 0.463908 | |  |  |  |  |  |  |
| genus.Veillonella.id.2198 | | rs1882878 | | A | G | | | -0.0768966 | | | 0.016390829 | | 2.98E-06 | | -0.0118799 | | | 0.025001 | | | 0.634667 | |  |  |  |  |  |  |
| genus.Veillonella.id.2198 | | rs2013594 | | T | C | | | -0.0720698 | | | 0.01551532 | | 3.42E-06 | | 0.0034392 | | | 0.023343 | | | 0.882872 | |  |  |  |  |  |  |
| genus.Veillonella.id.2198 | | rs62376424 | | C | T | | | -0.0762216 | | | 0.016349923 | | 3.65E-06 | | 0.001977 | | | 0.024922 | | | 0.936774 | |  |  |  |  |  |  |
| genus.Veillonella.id.2198 | | rs6656807 | | A | G | | | 0.0703062 | | | 0.015395397 | | 5.50E-06 | | -0.0213729 | | | 0.023778 | | | 0.368722 | |  |  |  |  |  |  |
| genus.Veillonella.id.2198 | | rs742016 | | A | G | | | -0.0688571 | | | 0.01497691 | | 4.66E-06 | | 0.0104126 | | | 0.024221 | | | 0.667272 | |  |  |  |  |  |  |
| genus.Veillonella.id.2198 | | rs7645873 | | A | T | | | 0.0761385 | | | 0.016422757 | | 3.12E-06 | | 0.015836 | | | 0.027156 | | | 0.559798 | |  |  |  |  |  |  |
| genus.Victivallis.id.2256 | | rs11899949 | | G | A | | | 0.1305678 | | | 0.027623889 | | 2.77E-06 | | 0.0079744 | | | 0.02475 | | | 0.747302 | |  |  |  |  |  |  |
| genus.Victivallis.id.2256 | | rs12512543 | | A | C | | | -0.1780361 | | | 0.037434441 | | 2.54E-06 | | -0.0452397 | | | 0.041479 | | | 0.275414 | |  |  |  |  |  |  |
| genus.Victivallis.id.2256 | | rs173120 | | T | C | | | 0.1338097 | | | 0.029012714 | | 7.65E-06 | | -0.0118198 | | | 0.029252 | | | 0.686163 | |  |  |  |  |  |  |
| genus.Victivallis.id.2256 | | rs1882775 | | A | G | | | -0.1382703 | | | 0.031272974 | | 8.73E-06 | | 0.0191663 | | | 0.029547 | | | 0.51655 | |  |  |  |  |  |  |
| genus.Victivallis.id.2256 | | rs2546432 | | T | C | | | -0.1108032 | | | 0.024965483 | | 9.93E-06 | | -0.0199719 | | | 0.022894 | | | 0.383017 | |  |  |  |  |  |  |
| genus.Victivallis.id.2256 | | rs342302 | | A | G | | | -0.152754 | | | 0.035157217 | | 8.16E-06 | | 0.0082216 | | | 0.033239 | | | 0.804638 | |  |  |  |  |  |  |
| genus.Victivallis.id.2256 | | rs4764863 | | G | A | | | 0.1215606 | | | 0.024605824 | | 8.22E-07 | | -0.0197194 | | | 0.023025 | | | 0.391765 | |  |  |  |  |  |  |
| genus.Victivallis.id.2256 | | rs4895919 | | T | C | | | -0.1169175 | | | 0.024751549 | | 2.75E-06 | | -0.0266907 | | | 0.022938 | | | 0.244574 | |  |  |  |  |  |  |
| genus.Victivallis.id.2256 | | rs56349194 | | A | G | | | -0.1585012 | | | 0.031523075 | | 6.26E-07 | | -0.0264225 | | | 0.034018 | | | 0.437328 | |  |  |  |  |  |  |
| genus.Victivallis.id.2256 | | rs592514 | | T | A | | | -0.1814489 | | | 0.039167751 | | 2.60E-06 | | -0.0490685 | | | 0.034365 | | | 0.153336 | |  |  |  |  |  |  |
| genus.Victivallis.id.2256 | | rs6445926 | | G | C | | | 0.1170307 | | | 0.024976353 | | 2.96E-06 | | -0.0018197 | | | 0.023184 | | | 0.93744 | |  |  |  |  |  |  |
| genus.Victivallis.id.2256 | | rs7374366 | | G | A | | | -0.1822739 | | | 0.040503861 | | 7.23E-06 | | 0.0064802 | | | 0.034932 | | | 0.852828 | |  |  |  |  |  |  |
| genus.Victivallis.id.2256 | | rs911666 | | T | C | | | -0.1185757 | | | 0.026314576 | | 7.65E-06 | | 0.0052309 | | | 0.024772 | | | 0.832758 | |  |  |  |  |  |  |

| **Table S2** The F-statistic data for the instrumental variables. |  |  |
| --- | --- | --- |
| **Bacterial taxa (exposure)** | **R_square** | **F_value** |
| genus..Clostridiuminnocuumgroup.id.14397 | 0.011401436 | 21.13870272 |
| genus..Eubacteriumbrachygroup.id.11296 | 0.012372309 | 20.87276009 |
| genus..Eubacteriumcoprostanoligenesgroup.id.11375 | 0.015472469 | 22.15420128 |
| genus..Eubacteriumeligensgroup.id.14372 | 0.011327969 | 21.00093289 |
| genus..Eubacteriumfissicatenagroup.id.14373 | 0.010371662 | 21.34500073 |
| genus..Eubacteriumhalliigroup.id.11338 | 0.016086244 | 21.39999049 |
| genus..Eubacteriumnodatumgroup.id.11297 | 0.012927489 | 21.82164687 |
| genus..Eubacteriumoxidoreducensgroup.id.11339 | 0.007120719 | 21.91339589 |
| genus..Eubacteriumrectalegroup.id.14374 | 0.013773487 | 21.32933762 |
| genus..Eubacteriumruminantiumgroup.id.11340 | 0.022244887 | 21.93673406 |
| genus..Eubacteriumventriosumgroup.id.11341 | 0.01904901 | 22.23830539 |
| genus..Eubacteriumxylanophilumgroup.id.14375 | 0.0141642 | 21.94308028 |
| genus..Ruminococcusgauvreauiigroup.id.11342 | 0.014369584 | 22.26589938 |
| genus..Ruminococcusgnavusgroup.id.14376 | 0.013051763 | 22.03419546 |
| genus..Ruminococcustorquesgroup.id.14377 | 0.01515308 | 21.68984889 |
| genus.Actinomyces.id.423 | 0.009016919 | 20.8491389 |
| genus.Adlercreutzia.id.812 | 0.013905617 | 21.53683669 |
| genus.Akkermansia.id.4037 | 0.015555493 | 22.27495657 |
| genus.Alistipes.id.968 | 0.014493285 | 20.73153982 |
| genus.Allisonella.id.2174 | 0.010889279 | 22.42198986 |
| genus.Alloprevotella.id.961 | 0.006652497 | 20.46283222 |
| genus.Anaerofilum.id.2053 | 0.013561359 | 22.90633092 |
| genus.Anaerostipes.id.1991 | 0.017449895 | 21.69537357 |
| genus.Anaerotruncus.id.2054 | 0.018011426 | 21.00478592 |
| genus.Bacteroides.id.918 | 0.013266177 | 22.40103914 |
| genus.Barnesiella.id.944 | 0.017647077 | 21.94493271 |
| genus.Bifidobacterium.id.436 | 0.03328195 | 28.66425158 |
| genus.Bilophila.id.3170 | 0.018321186 | 21.37276791 |
| genus.Blautia.id.1992 | 0.014058903 | 21.7776293 |
| genus.Butyricicoccus.id.2055 | 0.010180523 | 20.94758881 |
| genus.Butyricimonas.id.945 | 0.021200527 | 22.04598975 |
| genus.Butyrivibrio.id.1993 | 0.018961431 | 22.13408748 |
| genus.CandidatusSoleaferrea.id.11350 | 0.016861613 | 20.95142071 |
| genus.Catenibacterium.id.2153 | 0.004638721 | 21.36182822 |
| genus.ChristensenellaceaeR.7group.id.11283 | 0.010237375 | 21.06577965 |
| genus.Clostridiumsensustricto1.id.1873 | 0.008855588 | 20.47277245 |
| genus.Collinsella.id.815 | 0.014704977 | 21.0388697 |
| genus.Coprobacter.id.949 | 0.016027878 | 21.32107876 |
| genus.Coprococcus1.id.11301 | 0.016518799 | 21.98509593 |
| genus.Coprococcus2.id.11302 | 0.013732132 | 21.26440439 |
| genus.Coprococcus3.id.11303 | 0.012962685 | 21.88183722 |
| genus.DefluviitaleaceaeUCG011.id.11287 | 0.013432985 | 22.68654386 |
| genus.Desulfovibrio.id.3173 | 0.013666615 | 21.16154509 |
| genus.Dialister.id.2183 | 0.013348209 | 20.66185132 |
| genus.Dorea.id.1997 | 0.015065509 | 21.56258289 |
| genus.Eggerthella.id.819 | 0.01037691 | 21.35591414 |
| genus.Eisenbergiella.id.11304 | 0.013889352 | 21.51129084 |
| genus.Enterorhabdus.id.820 | 0.011382098 | 23.44843207 |
| genus.Erysipelatoclostridium.id.11381 | 0.020229141 | 22.25240132 |
| genus.ErysipelotrichaceaeUCG003.id.11384 | 0.020717285 | 21.5328457 |
| genus.Escherichia.Shigella.id.3504 | 0.017119189 | 21.27704687 |
| genus.Faecalibacterium.id.2057 | 0.015545709 | 22.26072469 |
| genus.FamilyXIIIAD3011group.id.11293 | 0.01768722 | 21.9957517 |
| genus.FamilyXIIIUCG001.id.11294 | 0.010535407 | 21.68557847 |
| genus.Flavonifractor.id.2059 | 0.011779929 | 21.84880955 |
| genus.Fusicatenibacter.id.11305 | 0.022993869 | 21.55691089 |
| genus.Gordonibacter.id.821 | 0.016554284 | 22.03311729 |
| genus.Haemophilus.id.3698 | 0.017478567 | 23.28518702 |
| genus.Holdemanella.id.11393 | 0.016794359 | 22.35810597 |
| genus.Holdemania.id.2157 | 0.020027581 | 22.02615071 |
| genus.Howardella.id.2000 | 0.011596151 | 21.50394823 |
| genus.Hungatella.id.11306 | 0.005564737 | 20.51896132 |
| genus.Intestinibacter.id.11345 | 0.017870008 | 22.22720202 |
| genus.Intestinimonas.id.2062 | 0.022336532 | 22.02917487 |
| genus.Lachnoclostridium.id.11308 | 0.017054198 | 21.19486947 |
| genus.Lachnospira.id.2004 | 0.008221318 | 21.70893344 |
| genus.LachnospiraceaeFCS020group.id.11314 | 0.02007486 | 22.07921279 |
| genus.LachnospiraceaeNC2004group.id.11316 | 0.011283431 | 20.91742183 |
| genus.LachnospiraceaeND3007group.id.11317 | 0.003420725 | 20.97923693 |
| genus.LachnospiraceaeNK4A136group.id.11319 | 0.018694062 | 21.81603649 |
| genus.LachnospiraceaeUCG001.id.11321 | 0.019000351 | 22.1804004 |
| genus.LachnospiraceaeUCG004.id.11324 | 0.016150296 | 21.48659831 |
| genus.LachnospiraceaeUCG008.id.11328 | 0.016661261 | 23.8852095 |
| genus.LachnospiraceaeUCG010.id.11330 | 0.014101615 | 21.84473697 |
| genus.Lactobacillus.id.1837 | 0.012037489 | 22.33233947 |
| genus.Lactococcus.id.1851 | 0.012592076 | 23.37434827 |
| genus.Marvinbryantia.id.2005 | 0.014233427 | 22.05187546 |
| genus.Methanobrevibacter.id.123 | 0.009129492 | 21.11182984 |
| genus.Odoribacter.id.952 | 0.010652144 | 21.92845206 |
| genus.Olsenella.id.822 | 0.011693985 | 21.68751827 |
| genus.Oscillibacter.id.2063 | 0.019180064 | 22.39429375 |
| genus.Oscillospira.id.2064 | 0.011676221 | 21.65418445 |
| genus.Oxalobacter.id.2978 | 0.01537092 | 23.84170717 |
| genus.Parabacteroides.id.954 | 0.009389827 | 21.71955703 |
| genus.Paraprevotella.id.962 | 0.015727601 | 22.52534802 |
| genus.Parasutterella.id.2892 | 0.019315783 | 22.55587827 |
| genus.Peptococcus.id.2037 | 0.022312506 | 23.22870262 |
| genus.Phascolarctobacterium.id.2168 | 0.013188131 | 22.26749093 |
| genus.Prevotella7.id.11182 | 0.01358614 | 21.03521998 |
| genus.Prevotella9.id.11183 | 0.021628552 | 21.31550045 |
| genus.RikenellaceaeRC9gutgroup.id.11191 | 0.016046265 | 21.34593669 |
| genus.Romboutsia.id.11347 | 0.017340972 | 21.55756088 |
| genus.Roseburia.id.2012 | 0.020724591 | 21.54059982 |
| genus.Ruminiclostridium5.id.11355 | 0.017565718 | 21.84195046 |
| genus.Ruminiclostridium6.id.11356 | 0.018223719 | 21.25695561 |
| genus.Ruminiclostridium9.id.11357 | 0.018883346 | 22.0411832 |
| genus.RuminococcaceaeNK4A214group.id.11358 | 0.020180945 | 22.19829324 |
| genus.RuminococcaceaeUCG002.id.11360 | 0.028196023 | 22.14138888 |
| genus.RuminococcaceaeUCG003.id.11361 | 0.01712352 | 22.80394845 |
| genus.RuminococcaceaeUCG004.id.11362 | 0.013963738 | 21.62812888 |
| genus.RuminococcaceaeUCG005.id.11363 | 0.019884792 | 21.86592639 |
| genus.RuminococcaceaeUCG009.id.11366 | 0.015599037 | 22.33829842 |
| genus.RuminococcaceaeUCG010.id.11367 | 0.009342044 | 21.60798813 |
| genus.RuminococcaceaeUCG011.id.11368 | 0.009906444 | 22.92649759 |
| genus.RuminococcaceaeUCG013.id.11370 | 0.016413865 | 21.84310612 |
| genus.RuminococcaceaeUCG014.id.11371 | 0.018292955 | 21.33922122 |
| genus.Ruminococcus1.id.11373 | 0.01644995 | 21.89193068 |
| genus.Ruminococcus2.id.11374 | 0.017278136 | 21.47807138 |
| genus.Sellimonas.id.14369 | 0.011960895 | 22.1885194 |
| genus.Senegalimassilia.id.11160 | 0.009248958 | 21.39067228 |
| genus.Slackia.id.825 | 0.010592134 | 21.80359357 |
| genus.Streptococcus.id.1853 | 0.019376065 | 22.62766212 |
| genus.Subdoligranulum.id.2070 | 0.016306499 | 21.69785894 |
| genus.Sutterella.id.2896 | 0.013755518 | 21.30112344 |
| genus.Terrisporobacter.id.11348 | 0.006905728 | 21.24717995 |
| genus.Turicibacter.id.2162 | 0.015554877 | 22.27406051 |
| genus.Tyzzerella3.id.11335 | 0.016297975 | 23.35578129 |
| genus.Veillonella.id.2198 | 0.008277229 | 21.85780222 |
| genus.Victivallis.id.2256 | 0.015267119 | 21.8556121 |

| **Table S3** The full result of MR estimates for the association between gut microbiota and puerperal sepsis. |  |  |  |  |
| --- | --- | --- | --- | --- |
| **Bacterial taxa (exposure)** | **MR method** | **No. of SNP** | **OR(95%CI)** | **P-value** |
| genus..Clostridiuminnocuumgroup.id.14397 | MR Egger | 8 | 1.25 (0.47- 3.33) | 0.67 |
| genus..Clostridiuminnocuumgroup.id.14397 | Weighted median | 8 | 1.03 (0.83- 1.26) | 0.81 |
| genus..Clostridiuminnocuumgroup.id.14397 | Inverse variance weighted | 8 | 0.96 (0.80- 1.16) | 0.69 |
| genus..Clostridiuminnocuumgroup.id.14397 | Simple mode | 8 | 1.07 (0.80- 1.42) | 0.67 |
| genus..Clostridiuminnocuumgroup.id.14397 | Weighted mode | 8 | 1.05 (0.80- 1.40) | 0.72 |
| genus..Eubacteriumbrachygroup.id.11296 | MR Egger | 10 | 0.82 (0.47- 1.43) | 0.5 |
| genus..Eubacteriumbrachygroup.id.11296 | Weighted median | 10 | 1.06 (0.89- 1.25) | 0.52 |
| genus..Eubacteriumbrachygroup.id.11296 | Inverse variance weighted | 10 | 1.02 (0.89- 1.17) | 0.79 |
| genus..Eubacteriumbrachygroup.id.11296 | Simple mode | 10 | 1.08 (0.83- 1.40) | 0.59 |
| genus..Eubacteriumbrachygroup.id.11296 | Weighted mode | 10 | 1.09 (0.85- 1.39) | 0.52 |
| genus..Eubacteriumcoprostanoligenesgroup.id.11375 | MR Egger | 12 | 0.46 (0.14- 1.53) | 0.23 |
| genus..Eubacteriumcoprostanoligenesgroup.id.11375 | Weighted median | 12 | 1.11 (0.77- 1.61) | 0.57 |
| genus..Eubacteriumcoprostanoligenesgroup.id.11375 | Inverse variance weighted | 12 | 0.89 (0.64- 1.25) | 0.51 |
| genus..Eubacteriumcoprostanoligenesgroup.id.11375 | Simple mode | 12 | 1.18 (0.69- 2.02) | 0.56 |
| genus..Eubacteriumcoprostanoligenesgroup.id.11375 | Weighted mode | 12 | 1.16 (0.70- 1.93) | 0.57 |
| genus..Eubacteriumeligensgroup.id.14372 | MR Egger | 7 | 1.17 (0.40- 3.47) | 0.79 |
| genus..Eubacteriumeligensgroup.id.14372 | Weighted median | 7 | 1.12 (0.77- 1.64) | 0.54 |
| genus..Eubacteriumeligensgroup.id.14372 | Inverse variance weighted | 7 | 1.11 (0.82- 1.51) | 0.5 |
| genus..Eubacteriumeligensgroup.id.14372 | Simple mode | 7 | 1.10 (0.60- 2.01) | 0.77 |
| genus..Eubacteriumeligensgroup.id.14372 | Weighted mode | 7 | 1.07 (0.60- 1.91) | 0.82 |
| genus..Eubacteriumfissicatenagroup.id.14373 | MR Egger | 9 | 1.38 (0.57- 3.32) | 0.5 |
| genus..Eubacteriumfissicatenagroup.id.14373 | Weighted median | 9 | 0.81 (0.65- 1.00) | 0.05 |
| genus..Eubacteriumfissicatenagroup.id.14373 | Inverse variance weighted | 9 | 0.92 (0.77- 1.08) | 0.31 |
| genus..Eubacteriumfissicatenagroup.id.14373 | Simple mode | 9 | 0.77 (0.54- 1.08) | 0.17 |
| genus..Eubacteriumfissicatenagroup.id.14373 | Weighted mode | 9 | 0.78 (0.55- 1.12) | 0.21 |
| genus..Eubacteriumhalliigroup.id.11338 | MR Egger | 14 | 1.32 (0.86- 2.02) | 0.22 |
| genus..Eubacteriumhalliigroup.id.11338 | Weighted median | 14 | 1.18 (0.90- 1.55) | 0.23 |
| genus..Eubacteriumhalliigroup.id.11338 | Inverse variance weighted | 14 | 1.07 (0.87- 1.31) | 0.52 |
| genus..Eubacteriumhalliigroup.id.11338 | Simple mode | 14 | 1.26 (0.83- 1.92) | 0.3 |
| genus..Eubacteriumhalliigroup.id.11338 | Weighted mode | 14 | 1.26 (0.87- 1.83) | 0.25 |
| genus..Eubacteriumnodatumgroup.id.11297 | MR Egger | 11 | 1.13 (0.66- 1.94) | 0.66 |
| genus..Eubacteriumnodatumgroup.id.11297 | Weighted median | 11 | 1.04 (0.88- 1.21) | 0.66 |
| genus..Eubacteriumnodatumgroup.id.11297 | Inverse variance weighted | 11 | 1.03 (0.92- 1.16) | 0.6 |
| genus..Eubacteriumnodatumgroup.id.11297 | Simple mode | 11 | 1.18 (0.92- 1.52) | 0.22 |
| genus..Eubacteriumnodatumgroup.id.11297 | Weighted mode | 11 | 1.15 (0.90- 1.48) | 0.3 |
| genus..Eubacteriumoxidoreducensgroup.id.11339 | MR Egger | 5 | 1.67 (0.74- 3.77) | 0.31 |
| genus..Eubacteriumoxidoreducensgroup.id.11339 | Weighted median | 5 | 1.11 (0.84- 1.46) | 0.48 |
| genus..Eubacteriumoxidoreducensgroup.id.11339 | Inverse variance weighted | 5 | 1.03 (0.83- 1.29) | 0.76 |
| genus..Eubacteriumoxidoreducensgroup.id.11339 | Simple mode | 5 | 1.20 (0.77- 1.87) | 0.46 |
| genus..Eubacteriumoxidoreducensgroup.id.11339 | Weighted mode | 5 | 1.18 (0.78- 1.80) | 0.47 |
| genus..Eubacteriumrectalegroup.id.14374 | MR Egger | 9 | 2.11 (0.52- 8.55) | 0.33 |
| genus..Eubacteriumrectalegroup.id.14374 | Weighted median | 9 | 1.02 (0.67- 1.55) | 0.93 |
| genus..Eubacteriumrectalegroup.id.14374 | Inverse variance weighted | 9 | 1.18 (0.82- 1.70) | 0.37 |
| genus..Eubacteriumrectalegroup.id.14374 | Simple mode | 9 | 0.88 (0.44- 1.76) | 0.73 |
| genus..Eubacteriumrectalegroup.id.14374 | Weighted mode | 9 | 0.92 (0.47- 1.82) | 0.82 |
| genus..Eubacteriumruminantiumgroup.id.11340 | MR Egger | 18 | 0.87 (0.56- 1.36) | 0.56 |
| genus..Eubacteriumruminantiumgroup.id.11340 | Weighted median | 18 | 1.10 (0.92- 1.31) | 0.28 |
| genus..Eubacteriumruminantiumgroup.id.11340 | Inverse variance weighted | 18 | 1.07 (0.93- 1.22) | 0.35 |
| genus..Eubacteriumruminantiumgroup.id.11340 | Simple mode | 18 | 1.11 (0.80- 1.53) | 0.53 |
| genus..Eubacteriumruminantiumgroup.id.11340 | Weighted mode | 18 | 1.08 (0.83- 1.41) | 0.57 |
| genus..Eubacteriumventriosumgroup.id.11341 | MR Egger | 15 | 0.57 (0.22- 1.48) | 0.27 |
| genus..Eubacteriumventriosumgroup.id.11341 | Weighted median | 15 | 0.86 (0.65- 1.15) | 0.31 |
| genus..Eubacteriumventriosumgroup.id.11341 | Inverse variance weighted | 15 | 0.90 (0.73- 1.11) | 0.32 |
| genus..Eubacteriumventriosumgroup.id.11341 | Simple mode | 15 | 0.84 (0.49- 1.44) | 0.54 |
| genus..Eubacteriumventriosumgroup.id.11341 | Weighted mode | 15 | 0.84 (0.51- 1.37) | 0.49 |
| genus..Eubacteriumxylanophilumgroup.id.14375 | MR Egger | 9 | 1.29 (0.64- 2.61) | 0.5 |
| genus..Eubacteriumxylanophilumgroup.id.14375 | Weighted median | 9 | 0.93 (0.68- 1.28) | 0.67 |
| genus..Eubacteriumxylanophilumgroup.id.14375 | Inverse variance weighted | 9 | 0.96 (0.76- 1.21) | 0.73 |
| genus..Eubacteriumxylanophilumgroup.id.14375 | Simple mode | 9 | 1.04 (0.64- 1.68) | 0.89 |
| genus..Eubacteriumxylanophilumgroup.id.14375 | Weighted mode | 9 | 0.99 (0.64- 1.53) | 0.97 |
| genus..Ruminococcusgauvreauiigroup.id.11342 | MR Egger | 11 | 0.69 (0.22- 2.13) | 0.54 |
| genus..Ruminococcusgauvreauiigroup.id.11342 | Weighted median | 11 | 1.12 (0.82- 1.53) | 0.49 |
| genus..Ruminococcusgauvreauiigroup.id.11342 | Inverse variance weighted | 11 | 0.92 (0.71- 1.20) | 0.56 |
| genus..Ruminococcusgauvreauiigroup.id.11342 | Simple mode | 11 | 1.14 (0.66- 1.98) | 0.65 |
| genus..Ruminococcusgauvreauiigroup.id.11342 | Weighted mode | 11 | 1.15 (0.70- 1.88) | 0.6 |
| genus..Ruminococcusgnavusgroup.id.14376 | MR Egger | 11 | 0.97 (0.41- 2.34) | 0.95 |
| genus..Ruminococcusgnavusgroup.id.14376 | Weighted median | 11 | 0.98 (0.78- 1.22) | 0.86 |
| genus..Ruminococcusgnavusgroup.id.14376 | Inverse variance weighted | 11 | 0.98 (0.82- 1.17) | 0.82 |
| genus..Ruminococcusgnavusgroup.id.14376 | Simple mode | 11 | 0.84 (0.55- 1.27) | 0.43 |
| genus..Ruminococcusgnavusgroup.id.14376 | Weighted mode | 11 | 0.94 (0.64- 1.37) | 0.75 |
| genus..Ruminococcustorquesgroup.id.14377 | MR Egger | 8 | 3.69 (0.72- 18.89) | 0.17 |
| genus..Ruminococcustorquesgroup.id.14377 | Weighted median | 8 | 0.82 (0.49- 1.34) | 0.42 |
| genus..Ruminococcustorquesgroup.id.14377 | Inverse variance weighted | 8 | 1.05 (0.61- 1.81) | 0.87 |
| genus..Ruminococcustorquesgroup.id.14377 | Simple mode | 8 | 0.59 (0.27- 1.30) | 0.23 |
| genus..Ruminococcustorquesgroup.id.14377 | Weighted mode | 8 | 0.66 (0.32- 1.35) | 0.29 |
| genus.Actinomyces.id.423 | MR Egger | 7 | 0.85 (0.49- 1.48) | 0.6 |
| genus.Actinomyces.id.423 | Weighted median | 7 | 0.94 (0.71- 1.24) | 0.64 |
| genus.Actinomyces.id.423 | Inverse variance weighted | 7 | 0.92 (0.75- 1.14) | 0.46 |
| genus.Actinomyces.id.423 | Simple mode | 7 | 0.97 (0.64- 1.49) | 0.91 |
| genus.Actinomyces.id.423 | Weighted mode | 7 | 0.98 (0.70- 1.36) | 0.9 |
| genus.Adlercreutzia.id.812 | MR Egger | 8 | 2.43 (0.81- 7.27) | 0.16 |
| genus.Adlercreutzia.id.812 | Weighted median | 8 | 1.09 (0.81- 1.47) | 0.57 |
| genus.Adlercreutzia.id.812 | Inverse variance weighted | 8 | 1.07 (0.82- 1.40) | 0.6 |
| genus.Adlercreutzia.id.812 | Simple mode | 8 | 1.08 (0.65- 1.81) | 0.77 |
| genus.Adlercreutzia.id.812 | Weighted mode | 8 | 1.08 (0.67- 1.73) | 0.77 |
| genus.Akkermansia.id.4037 | MR Egger | 12 | 0.98 (0.40- 2.39) | 0.96 |
| genus.Akkermansia.id.4037 | Weighted median | 12 | 0.95 (0.71- 1.27) | 0.72 |
| genus.Akkermansia.id.4037 | Inverse variance weighted | 12 | 0.99 (0.78- 1.26) | 0.94 |
| genus.Akkermansia.id.4037 | Simple mode | 12 | 0.82 (0.54- 1.25) | 0.38 |
| genus.Akkermansia.id.4037 | Weighted mode | 12 | 0.88 (0.59- 1.31) | 0.54 |
| genus.Alistipes.id.968 | MR Egger | 12 | 0.52 (0.14- 1.98) | 0.36 |
| genus.Alistipes.id.968 | Weighted median | 12 | 0.76 (0.54- 1.08) | 0.13 |
| genus.Alistipes.id.968 | Inverse variance weighted | 12 | 0.80 (0.60- 1.05) | 0.11 |
| genus.Alistipes.id.968 | Simple mode | 12 | 0.70 (0.39- 1.29) | 0.28 |
| genus.Alistipes.id.968 | Weighted mode | 12 | 0.69 (0.38- 1.28) | 0.27 |
| genus.Allisonella.id.2174 | MR Egger | 8 | 1.22 (0.50- 2.98) | 0.68 |
| genus.Allisonella.id.2174 | Weighted median | 8 | 0.89 (0.75- 1.06) | 0.2 |
| genus.Allisonella.id.2174 | Inverse variance weighted | 8 | 0.88 (0.77- 1.00) | 0.06 |
| genus.Allisonella.id.2174 | Simple mode | 8 | 0.80 (0.61- 1.04) | 0.14 |
| genus.Allisonella.id.2174 | Weighted mode | 8 | 0.92 (0.71- 1.19) | 0.55 |
| genus.Alloprevotella.id.961 | MR Egger | 5 | 0.32 (0.01- 7.54) | 0.53 |
| genus.Alloprevotella.id.961 | Weighted median | 5 | 0.85 (0.64- 1.13) | 0.26 |
| genus.Alloprevotella.id.961 | Inverse variance weighted | 5 | 0.98 (0.70- 1.38) | 0.9 |
| genus.Alloprevotella.id.961 | Simple mode | 5 | 0.76 (0.50- 1.15) | 0.27 |
| genus.Alloprevotella.id.961 | Weighted mode | 5 | 0.76 (0.54- 1.07) | 0.19 |
| genus.Anaerofilum.id.2053 | MR Egger | 10 | 0.65 (0.26- 1.65) | 0.39 |
| genus.Anaerofilum.id.2053 | Weighted median | 10 | 1.03 (0.84- 1.27) | 0.77 |
| genus.Anaerofilum.id.2053 | Inverse variance weighted | 10 | 1.04 (0.87- 1.23) | 0.69 |
| genus.Anaerofilum.id.2053 | Simple mode | 10 | 1.03 (0.68- 1.54) | 0.91 |
| genus.Anaerofilum.id.2053 | Weighted mode | 10 | 1.03 (0.69- 1.55) | 0.88 |
| genus.Anaerostipes.id.1991 | MR Egger | 13 | 0.84 (0.32- 2.23) | 0.73 |
| genus.Anaerostipes.id.1991 | Weighted median | 13 | 0.98 (0.68- 1.42) | 0.93 |
| genus.Anaerostipes.id.1991 | Inverse variance weighted | 13 | 1.09 (0.84- 1.41) | 0.53 |
| genus.Anaerostipes.id.1991 | Simple mode | 13 | 0.92 (0.52- 1.63) | 0.77 |
| genus.Anaerostipes.id.1991 | Weighted mode | 13 | 0.94 (0.55- 1.61) | 0.83 |
| genus.Anaerotruncus.id.2054 | MR Egger | 13 | 1.88 (0.90- 3.89) | 0.12 |
| genus.Anaerotruncus.id.2054 | Weighted median | 13 | 1.12 (0.78- 1.62) | 0.53 |
| genus.Anaerotruncus.id.2054 | Inverse variance weighted | 13 | 1.00 (0.76- 1.32) | 0.98 |
| genus.Anaerotruncus.id.2054 | Simple mode | 13 | 1.09 (0.62- 1.92) | 0.76 |
| genus.Anaerotruncus.id.2054 | Weighted mode | 13 | 1.17 (0.68- 1.99) | 0.59 |
| genus.Bacteroides.id.918 | MR Egger | 8 | 1.49 (0.30- 7.34) | 0.64 |
| genus.Bacteroides.id.918 | Weighted median | 8 | 1.18 (0.80- 1.76) | 0.41 |
| genus.Bacteroides.id.918 | Inverse variance weighted | 8 | 1.18 (0.87- 1.60) | 0.28 |
| genus.Bacteroides.id.918 | Simple mode | 8 | 1.18 (0.65- 2.12) | 0.6 |
| genus.Bacteroides.id.918 | Weighted mode | 8 | 1.18 (0.71- 1.97) | 0.55 |
| genus.Barnesiella.id.944 | MR Egger | 12 | 1.62 (0.63- 4.22) | 0.34 |
| genus.Barnesiella.id.944 | Weighted median | 12 | 1.13 (0.83- 1.54) | 0.44 |
| genus.Barnesiella.id.944 | Inverse variance weighted | 12 | 1.11 (0.88- 1.41) | 0.37 |
| genus.Barnesiella.id.944 | Simple mode | 12 | 1.13 (0.66- 1.92) | 0.66 |
| genus.Barnesiella.id.944 | Weighted mode | 12 | 1.13 (0.69- 1.85) | 0.63 |
| genus.Bifidobacterium.id.436 | MR Egger | 19 | 0.86 (0.55- 1.35) | 0.53 |
| genus.Bifidobacterium.id.436 | Weighted median | 19 | 0.98 (0.79- 1.22) | 0.85 |
| genus.Bifidobacterium.id.436 | Inverse variance weighted | 19 | 1.05 (0.90- 1.24) | 0.52 |
| genus.Bifidobacterium.id.436 | Simple mode | 19 | 1.00 (0.69- 1.45) | 0.99 |
| genus.Bifidobacterium.id.436 | Weighted mode | 19 | 1.00 (0.77- 1.29) | 0.98 |
| genus.Bilophila.id.3170 | MR Egger | 13 | 1.11 (0.37- 3.33) | 0.85 |
| genus.Bilophila.id.3170 | Weighted median | 13 | 0.96 (0.71- 1.30) | 0.8 |
| genus.Bilophila.id.3170 | Inverse variance weighted | 13 | 0.95 (0.76- 1.19) | 0.68 |
| genus.Bilophila.id.3170 | Simple mode | 13 | 0.98 (0.60- 1.59) | 0.93 |
| genus.Bilophila.id.3170 | Weighted mode | 13 | 0.96 (0.60- 1.55) | 0.88 |
| genus.Blautia.id.1992 | MR Egger | 12 | 0.81 (0.34- 1.93) | 0.64 |
| genus.Blautia.id.1992 | Weighted median | 12 | 0.86 (0.58- 1.26) | 0.44 |
| genus.Blautia.id.1992 | Inverse variance weighted | 12 | 0.95 (0.69- 1.32) | 0.76 |
| genus.Blautia.id.1992 | Simple mode | 12 | 0.79 (0.38- 1.67) | 0.55 |
| genus.Blautia.id.1992 | Weighted mode | 12 | 0.72 (0.34- 1.51) | 0.4 |
| genus.Butyricicoccus.id.2055 | MR Egger | 8 | 1.04 (0.61- 1.77) | 0.9 |
| genus.Butyricicoccus.id.2055 | Weighted median | 8 | 0.90 (0.63- 1.29) | 0.58 |
| genus.Butyricicoccus.id.2055 | Inverse variance weighted | 8 | 0.88 (0.67- 1.15) | 0.35 |
| genus.Butyricicoccus.id.2055 | Simple mode | 8 | 0.68 (0.39- 1.19) | 0.22 |
| genus.Butyricicoccus.id.2055 | Weighted mode | 8 | 0.95 (0.60- 1.50) | 0.83 |
| genus.Butyricimonas.id.945 | MR Egger | 13 | 0.54 (0.26- 1.12) | 0.12 |
| genus.Butyricimonas.id.945 | Weighted median | 13 | 0.84 (0.64- 1.10) | 0.21 |
| genus.Butyricimonas.id.945 | Inverse variance weighted | 13 | 0.82 (0.67- 1.01) | 0.07 |
| genus.Butyricimonas.id.945 | Simple mode | 13 | 0.84 (0.53- 1.32) | 0.46 |
| genus.Butyricimonas.id.945 | Weighted mode | 13 | 0.83 (0.51- 1.37) | 0.49 |
| genus.Butyrivibrio.id.1993 | MR Egger | 15 | 0.74 (0.48- 1.14) | 0.19 |
| genus.Butyrivibrio.id.1993 | Weighted median | 15 | 0.89 (0.78- 1.02) | 0.09 |
| genus.Butyrivibrio.id.1993 | Inverse variance weighted | 15 | 0.93 (0.84- 1.03) | 0.15 |
| genus.Butyrivibrio.id.1993 | Simple mode | 15 | 0.86 (0.70- 1.06) | 0.17 |
| genus.Butyrivibrio.id.1993 | Weighted mode | 15 | 0.86 (0.70- 1.06) | 0.18 |
| genus.CandidatusSoleaferrea.id.11350 | MR Egger | 9 | 1.90 (0.27- 13.57) | 0.54 |
| genus.CandidatusSoleaferrea.id.11350 | Weighted median | 9 | 1.18 (0.92- 1.51) | 0.2 |
| genus.CandidatusSoleaferrea.id.11350 | Inverse variance weighted | 9 | 1.02 (0.85- 1.22) | 0.86 |
| genus.CandidatusSoleaferrea.id.11350 | Simple mode | 9 | 1.21 (0.81- 1.82) | 0.38 |
| genus.CandidatusSoleaferrea.id.11350 | Weighted mode | 9 | 1.21 (0.84- 1.75) | 0.33 |
| genus.Catenibacterium.id.2153 | MR Egger | 4 | 0.38 (0.03- 4.62) | 0.53 |
| genus.Catenibacterium.id.2153 | Weighted median | 4 | 0.98 (0.78- 1.24) | 0.89 |
| genus.Catenibacterium.id.2153 | Inverse variance weighted | 4 | 0.99 (0.81- 1.21) | 0.92 |
| genus.Catenibacterium.id.2153 | Simple mode | 4 | 0.92 (0.67- 1.27) | 0.65 |
| genus.Catenibacterium.id.2153 | Weighted mode | 4 | 0.93 (0.67- 1.27) | 0.66 |
| genus.ChristensenellaceaeR.7group.id.11283 | MR Egger | 8 | 1.60 (0.52- 4.95) | 0.45 |
| genus.ChristensenellaceaeR.7group.id.11283 | Weighted median | 8 | 1.10 (0.70- 1.70) | 0.69 |
| genus.ChristensenellaceaeR.7group.id.11283 | Inverse variance weighted | 8 | 1.19 (0.86- 1.64) | 0.29 |
| genus.ChristensenellaceaeR.7group.id.11283 | Simple mode | 8 | 0.93 (0.48- 1.81) | 0.83 |
| genus.ChristensenellaceaeR.7group.id.11283 | Weighted mode | 8 | 0.92 (0.45- 1.87) | 0.83 |
| genus.Clostridiumsensustricto1.id.1873 | MR Egger | 6 | 1.34 (0.65- 2.75) | 0.48 |
| genus.Clostridiumsensustricto1.id.1873 | Weighted median | 6 | 1.04 (0.72- 1.51) | 0.83 |
| genus.Clostridiumsensustricto1.id.1873 | Inverse variance weighted | 6 | 1.07 (0.82- 1.40) | 0.62 |
| genus.Clostridiumsensustricto1.id.1873 | Simple mode | 6 | 1.04 (0.63- 1.72) | 0.87 |
| genus.Clostridiumsensustricto1.id.1873 | Weighted mode | 6 | 1.04 (0.66- 1.65) | 0.86 |
| genus.Collinsella.id.815 | MR Egger | 10 | 2.63 (0.89- 7.77) | 0.12 |
| genus.Collinsella.id.815 | Weighted median | 10 | 0.95 (0.65- 1.39) | 0.8 |
| genus.Collinsella.id.815 | Inverse variance weighted | 10 | 1.20 (0.91- 1.58) | 0.2 |
| genus.Collinsella.id.815 | Simple mode | 10 | 0.88 (0.47- 1.66) | 0.71 |
| genus.Collinsella.id.815 | Weighted mode | 10 | 0.87 (0.48- 1.59) | 0.67 |
| genus.Coprobacter.id.949 | MR Egger | 11 | 1.58 (0.79- 3.16) | 0.23 |
| genus.Coprobacter.id.949 | Weighted median | 11 | 0.98 (0.77- 1.26) | 0.9 |
| genus.Coprobacter.id.949 | Inverse variance weighted | 11 | 1.05 (0.88- 1.26) | 0.59 |
| genus.Coprobacter.id.949 | Simple mode | 11 | 0.85 (0.53- 1.36) | 0.51 |
| genus.Coprobacter.id.949 | Weighted mode | 11 | 0.86 (0.55- 1.34) | 0.52 |
| genus.Coprococcus1.id.11301 | MR Egger | 12 | 0.90 (0.49- 1.66) | 0.74 |
| genus.Coprococcus1.id.11301 | Weighted median | 12 | 0.90 (0.66- 1.25) | 0.54 |
| genus.Coprococcus1.id.11301 | Inverse variance weighted | 12 | 0.83 (0.66- 1.04) | 0.11 |
| genus.Coprococcus1.id.11301 | Simple mode | 12 | 0.92 (0.52- 1.60) | 0.76 |
| genus.Coprococcus1.id.11301 | Weighted mode | 12 | 1.02 (0.65- 1.59) | 0.93 |
| genus.Coprococcus2.id.11302 | MR Egger | 9 | 0.95 (0.24- 3.75) | 0.94 |
| genus.Coprococcus2.id.11302 | Weighted median | 9 | 0.78 (0.56- 1.09) | 0.14 |
| genus.Coprococcus2.id.11302 | Inverse variance weighted | 9 | 0.79 (0.62- 1.01) | 0.06 |
| genus.Coprococcus2.id.11302 | Simple mode | 9 | 0.65 (0.38- 1.09) | 0.14 |
| genus.Coprococcus2.id.11302 | Weighted mode | 9 | 0.68 (0.40- 1.16) | 0.2 |
| genus.Coprococcus3.id.11303 | MR Egger | 10 | 3.60 (0.65- 19.88) | 0.18 |
| genus.Coprococcus3.id.11303 | Weighted median | 10 | 1.29 (0.91- 1.84) | 0.15 |
| genus.Coprococcus3.id.11303 | Inverse variance weighted | 10 | 1.20 (0.91- 1.58) | 0.19 |
| genus.Coprococcus3.id.11303 | Simple mode | 10 | 1.32 (0.76- 2.31) | 0.35 |
| genus.Coprococcus3.id.11303 | Weighted mode | 10 | 1.32 (0.78- 2.22) | 0.33 |
| genus.DefluviitaleaceaeUCG011.id.11287 | MR Egger | 10 | 1.74 (0.73- 4.17) | 0.25 |
| genus.DefluviitaleaceaeUCG011.id.11287 | Weighted median | 10 | 1.19 (0.89- 1.59) | 0.25 |
| genus.DefluviitaleaceaeUCG011.id.11287 | Inverse variance weighted | 10 | 1.15 (0.91- 1.45) | 0.23 |
| genus.DefluviitaleaceaeUCG011.id.11287 | Simple mode | 10 | 1.43 (0.88- 2.32) | 0.18 |
| genus.DefluviitaleaceaeUCG011.id.11287 | Weighted mode | 10 | 1.40 (0.85- 2.32) | 0.22 |
| genus.Desulfovibrio.id.3173 | MR Egger | 11 | 1.53 (0.84- 2.79) | 0.19 |
| genus.Desulfovibrio.id.3173 | Weighted median | 11 | 1.10 (0.83- 1.44) | 0.51 |
| genus.Desulfovibrio.id.3173 | Inverse variance weighted | 11 | 1.03 (0.84- 1.25) | 0.8 |
| genus.Desulfovibrio.id.3173 | Simple mode | 11 | 0.79 (0.50- 1.25) | 0.33 |
| genus.Desulfovibrio.id.3173 | Weighted mode | 11 | 1.17 (0.78- 1.75) | 0.47 |
| genus.Dialister.id.2183 | MR Egger | 11 | 0.44 (0.18- 1.10) | 0.11 |
| genus.Dialister.id.2183 | Weighted median | 11 | 0.94 (0.68- 1.28) | 0.69 |
| genus.Dialister.id.2183 | Inverse variance weighted | 11 | 0.87 (0.69- 1.09) | 0.22 |
| genus.Dialister.id.2183 | Simple mode | 11 | 0.96 (0.56- 1.66) | 0.89 |
| genus.Dialister.id.2183 | Weighted mode | 11 | 0.95 (0.55- 1.63) | 0.86 |
| genus.Dorea.id.1997 | MR Egger | 11 | 1.42 (0.78- 2.59) | 0.28 |
| genus.Dorea.id.1997 | Weighted median | 11 | 1.11 (0.79- 1.57) | 0.53 |
| genus.Dorea.id.1997 | Inverse variance weighted | 11 | 1.12 (0.88- 1.44) | 0.36 |
| genus.Dorea.id.1997 | Simple mode | 11 | 1.08 (0.67- 1.74) | 0.77 |
| genus.Dorea.id.1997 | Weighted mode | 11 | 1.06 (0.69- 1.64) | 0.79 |
| genus.Eggerthella.id.819 | MR Egger | 9 | 0.54 (0.26- 1.13) | 0.15 |
| genus.Eggerthella.id.819 | Weighted median | 9 | 1.06 (0.84- 1.34) | 0.6 |
| genus.Eggerthella.id.819 | Inverse variance weighted | 9 | 1.08 (0.92- 1.27) | 0.36 |
| genus.Eggerthella.id.819 | Simple mode | 9 | 1.10 (0.74- 1.64) | 0.64 |
| genus.Eggerthella.id.819 | Weighted mode | 9 | 1.09 (0.74- 1.61) | 0.68 |
| genus.Eisenbergiella.id.11304 | MR Egger | 11 | 0.75 (0.21- 2.65) | 0.67 |
| genus.Eisenbergiella.id.11304 | Weighted median | 11 | 1.22 (0.98- 1.53) | 0.08 |
| genus.Eisenbergiella.id.11304 | Inverse variance weighted | 11 | 1.19 (1.01- 1.41) | 3.80E-02 |
| genus.Eisenbergiella.id.11304 | Simple mode | 11 | 1.44 (0.98- 2.12) | 0.09 |
| genus.Eisenbergiella.id.11304 | Weighted mode | 11 | 1.44 (0.98- 2.11) | 0.09 |
| genus.Enterorhabdus.id.820 | MR Egger | 6 | 1.62 (0.85- 3.07) | 0.21 |
| genus.Enterorhabdus.id.820 | Weighted median | 6 | 0.87 (0.62- 1.21) | 0.4 |
| genus.Enterorhabdus.id.820 | Inverse variance weighted | 6 | 0.85 (0.64- 1.13) | 0.26 |
| genus.Enterorhabdus.id.820 | Simple mode | 6 | 0.81 (0.51- 1.29) | 0.42 |
| genus.Enterorhabdus.id.820 | Weighted mode | 6 | 1.08 (0.73- 1.60) | 0.71 |
| genus.Erysipelatoclostridium.id.11381 | MR Egger | 16 | 1.94 (0.90- 4.16) | 0.11 |
| genus.Erysipelatoclostridium.id.11381 | Weighted median | 16 | 1.05 (0.83- 1.34) | 0.67 |
| genus.Erysipelatoclostridium.id.11381 | Inverse variance weighted | 16 | 1.05 (0.85- 1.28) | 0.66 |
| genus.Erysipelatoclostridium.id.11381 | Simple mode | 16 | 1.23 (0.79- 1.91) | 0.38 |
| genus.Erysipelatoclostridium.id.11381 | Weighted mode | 16 | 1.19 (0.80- 1.78) | 0.41 |
| genus.ErysipelotrichaceaeUCG003.id.11384 | MR Egger | 17 | 0.96 (0.57- 1.62) | 0.88 |
| genus.ErysipelotrichaceaeUCG003.id.11384 | Weighted median | 17 | 1.05 (0.82- 1.35) | 0.7 |
| genus.ErysipelotrichaceaeUCG003.id.11384 | Inverse variance weighted | 17 | 1.09 (0.90- 1.31) | 0.38 |
| genus.ErysipelotrichaceaeUCG003.id.11384 | Simple mode | 17 | 0.99 (0.64- 1.54) | 0.97 |
| genus.ErysipelotrichaceaeUCG003.id.11384 | Weighted mode | 17 | 1.00 (0.65- 1.53) | 1 |
| genus.Escherichia.Shigella.id.3504 | MR Egger | 10 | 2.91 (1.20- 7.07) | 4.60E-02 |
| genus.Escherichia.Shigella.id.3504 | Weighted median | 10 | 1.04 (0.72- 1.51) | 0.82 |
| genus.Escherichia.Shigella.id.3504 | Inverse variance weighted | 10 | 1.01 (0.71- 1.44) | 0.97 |
| genus.Escherichia.Shigella.id.3504 | Simple mode | 10 | 0.99 (0.53- 1.86) | 0.97 |
| genus.Escherichia.Shigella.id.3504 | Weighted mode | 10 | 1.08 (0.61- 1.93) | 0.79 |
| genus.Faecalibacterium.id.2057 | MR Egger | 10 | 1.22 (0.69- 2.13) | 0.51 |
| genus.Faecalibacterium.id.2057 | Weighted median | 10 | 0.88 (0.63- 1.23) | 0.45 |
| genus.Faecalibacterium.id.2057 | Inverse variance weighted | 10 | 1.05 (0.79- 1.38) | 0.75 |
| genus.Faecalibacterium.id.2057 | Simple mode | 10 | 0.77 (0.40- 1.49) | 0.46 |
| genus.Faecalibacterium.id.2057 | Weighted mode | 10 | 0.80 (0.53- 1.22) | 0.33 |
| genus.FamilyXIIIAD3011group.id.11293 | MR Egger | 13 | 1.46 (0.41- 5.15) | 0.57 |
| genus.FamilyXIIIAD3011group.id.11293 | Weighted median | 13 | 1.03 (0.75- 1.43) | 0.84 |
| genus.FamilyXIIIAD3011group.id.11293 | Inverse variance weighted | 13 | 0.97 (0.75- 1.26) | 0.81 |
| genus.FamilyXIIIAD3011group.id.11293 | Simple mode | 13 | 1.15 (0.65- 2.03) | 0.65 |
| genus.FamilyXIIIAD3011group.id.11293 | Weighted mode | 13 | 1.12 (0.65- 1.91) | 0.7 |
| genus.FamilyXIIIUCG001.id.11294 | MR Egger | 8 | 1.30 (0.58- 2.89) | 0.55 |
| genus.FamilyXIIIUCG001.id.11294 | Weighted median | 8 | 0.92 (0.65- 1.31) | 0.65 |
| genus.FamilyXIIIUCG001.id.11294 | Inverse variance weighted | 8 | 0.99 (0.76- 1.28) | 0.91 |
| genus.FamilyXIIIUCG001.id.11294 | Simple mode | 8 | 0.89 (0.54- 1.47) | 0.66 |
| genus.FamilyXIIIUCG001.id.11294 | Weighted mode | 8 | 0.90 (0.56- 1.46) | 0.68 |
| genus.Flavonifractor.id.2059 | MR Egger | 6 | 0.60 (0.18- 1.97) | 0.45 |
| genus.Flavonifractor.id.2059 | Weighted median | 6 | 0.92 (0.64- 1.31) | 0.64 |
| genus.Flavonifractor.id.2059 | Inverse variance weighted | 6 | 0.92 (0.69- 1.23) | 0.59 |
| genus.Flavonifractor.id.2059 | Simple mode | 6 | 0.90 (0.51- 1.56) | 0.71 |
| genus.Flavonifractor.id.2059 | Weighted mode | 6 | 0.91 (0.52- 1.58) | 0.74 |
| genus.Fusicatenibacter.id.11305 | MR Egger | 19 | 1.11 (0.52- 2.41) | 0.79 |
| genus.Fusicatenibacter.id.11305 | Weighted median | 19 | 0.90 (0.68- 1.18) | 0.43 |
| genus.Fusicatenibacter.id.11305 | Inverse variance weighted | 19 | 0.86 (0.70- 1.06) | 0.15 |
| genus.Fusicatenibacter.id.11305 | Simple mode | 19 | 0.94 (0.58- 1.52) | 0.8 |
| genus.Fusicatenibacter.id.11305 | Weighted mode | 19 | 0.91 (0.59- 1.40) | 0.66 |
| genus.Gordonibacter.id.821 | MR Egger | 11 | 0.75 (0.43- 1.31) | 0.34 |
| genus.Gordonibacter.id.821 | Weighted median | 11 | 0.96 (0.81- 1.14) | 0.63 |
| genus.Gordonibacter.id.821 | Inverse variance weighted | 11 | 0.94 (0.83- 1.07) | 0.36 |
| genus.Gordonibacter.id.821 | Simple mode | 11 | 0.94 (0.73- 1.23) | 0.68 |
| genus.Gordonibacter.id.821 | Weighted mode | 11 | 0.95 (0.72- 1.25) | 0.71 |
| genus.Haemophilus.id.3698 | MR Egger | 9 | 1.07 (0.71- 1.62) | 0.74 |
| genus.Haemophilus.id.3698 | Weighted median | 9 | 1.01 (0.79- 1.29) | 0.94 |
| genus.Haemophilus.id.3698 | Inverse variance weighted | 9 | 0.96 (0.80- 1.16) | 0.7 |
| genus.Haemophilus.id.3698 | Simple mode | 9 | 1.06 (0.73- 1.53) | 0.77 |
| genus.Haemophilus.id.3698 | Weighted mode | 9 | 1.06 (0.78- 1.44) | 0.72 |
| genus.Holdemanella.id.11393 | MR Egger | 11 | 1.12 (0.71- 1.78) | 0.63 |
| genus.Holdemanella.id.11393 | Weighted median | 11 | 1.09 (0.87- 1.35) | 0.46 |
| genus.Holdemanella.id.11393 | Inverse variance weighted | 11 | 1.06 (0.90- 1.24) | 0.48 |
| genus.Holdemanella.id.11393 | Simple mode | 11 | 1.20 (0.80- 1.81) | 0.39 |
| genus.Holdemanella.id.11393 | Weighted mode | 11 | 1.22 (0.83- 1.79) | 0.34 |
| genus.Holdemania.id.2157 | MR Egger | 14 | 0.97 (0.59- 1.62) | 0.92 |
| genus.Holdemania.id.2157 | Weighted median | 14 | 1.01 (0.80- 1.28) | 0.95 |
| genus.Holdemania.id.2157 | Inverse variance weighted | 14 | 1.05 (0.88- 1.24) | 0.6 |
| genus.Holdemania.id.2157 | Simple mode | 14 | 1.30 (0.84- 2.01) | 0.25 |
| genus.Holdemania.id.2157 | Weighted mode | 14 | 0.90 (0.62- 1.31) | 0.6 |
| genus.Howardella.id.2000 | MR Egger | 9 | 0.84 (0.33- 2.12) | 0.72 |
| genus.Howardella.id.2000 | Weighted median | 9 | 1.06 (0.88- 1.29) | 0.53 |
| genus.Howardella.id.2000 | Inverse variance weighted | 9 | 1.04 (0.83- 1.30) | 0.74 |
| genus.Howardella.id.2000 | Simple mode | 9 | 1.29 (0.96- 1.74) | 0.12 |
| genus.Howardella.id.2000 | Weighted mode | 9 | 1.15 (0.89- 1.48) | 0.31 |
| genus.Hungatella.id.11306 | MR Egger | 5 | 0.28 (0.09- 0.94) | 0.13 |
| genus.Hungatella.id.11306 | Weighted median | 5 | 0.91 (0.70- 1.19) | 0.49 |
| genus.Hungatella.id.11306 | Inverse variance weighted | 5 | 0.94 (0.77- 1.14) | 0.52 |
| genus.Hungatella.id.11306 | Simple mode | 5 | 0.87 (0.60- 1.26) | 0.51 |
| genus.Hungatella.id.11306 | Weighted mode | 5 | 0.84 (0.58- 1.23) | 0.42 |
| genus.Intestinibacter.id.11345 | MR Egger | 15 | 1.91 (1.04- 3.52) | 0.06 |
| genus.Intestinibacter.id.11345 | Weighted median | 15 | 1.21 (0.93- 1.58) | 0.15 |
| genus.Intestinibacter.id.11345 | Inverse variance weighted | 15 | 1.16 (0.97- 1.41) | 0.11 |
| genus.Intestinibacter.id.11345 | Simple mode | 15 | 1.26 (0.82- 1.94) | 0.31 |
| genus.Intestinibacter.id.11345 | Weighted mode | 15 | 1.26 (0.83- 1.90) | 0.29 |
| genus.Intestinimonas.id.2062 | MR Egger | 16 | 1.66 (0.98- 2.80) | 0.08 |
| genus.Intestinimonas.id.2062 | Weighted median | 16 | 1.20 (0.94- 1.54) | 0.15 |
| genus.Intestinimonas.id.2062 | Inverse variance weighted | 16 | 1.20 (0.99- 1.45) | 0.06 |
| genus.Intestinimonas.id.2062 | Simple mode | 16 | 1.42 (0.86- 2.35) | 0.19 |
| genus.Intestinimonas.id.2062 | Weighted mode | 16 | 1.42 (0.88- 2.31) | 0.18 |
| genus.Lachnoclostridium.id.11308 | MR Egger | 13 | 1.06 (0.36- 3.16) | 0.92 |
| genus.Lachnoclostridium.id.11308 | Weighted median | 13 | 0.93 (0.65- 1.34) | 0.71 |
| genus.Lachnoclostridium.id.11308 | Inverse variance weighted | 13 | 0.86 (0.63- 1.17) | 0.34 |
| genus.Lachnoclostridium.id.11308 | Simple mode | 13 | 0.63 (0.31- 1.29) | 0.23 |
| genus.Lachnoclostridium.id.11308 | Weighted mode | 13 | 1.33 (0.69- 2.56) | 0.42 |
| genus.Lachnospira.id.2004 | MR Egger | 6 | 0.19 (0.02- 1.74) | 0.22 |
| genus.Lachnospira.id.2004 | Weighted median | 6 | 0.82 (0.52- 1.29) | 0.39 |
| genus.Lachnospira.id.2004 | Inverse variance weighted | 6 | 0.79 (0.54- 1.15) | 0.22 |
| genus.Lachnospira.id.2004 | Simple mode | 6 | 1.01 (0.49- 2.09) | 0.98 |
| genus.Lachnospira.id.2004 | Weighted mode | 6 | 1.01 (0.49- 2.07) | 0.97 |
| genus.LachnospiraceaeFCS020group.id.11314 | MR Egger | 13 | 0.93 (0.53- 1.64) | 0.81 |
| genus.LachnospiraceaeFCS020group.id.11314 | Weighted median | 13 | 0.84 (0.63- 1.11) | 0.22 |
| genus.LachnospiraceaeFCS020group.id.11314 | Inverse variance weighted | 13 | 0.81 (0.66- 1.00) | 4.70E-02 |
| genus.LachnospiraceaeFCS020group.id.11314 | Simple mode | 13 | 0.74 (0.45- 1.20) | 0.24 |
| genus.LachnospiraceaeFCS020group.id.11314 | Weighted mode | 13 | 0.78 (0.50- 1.23) | 0.31 |
| genus.LachnospiraceaeNC2004group.id.11316 | MR Egger | 9 | 0.59 (0.29- 1.22) | 0.2 |
| genus.LachnospiraceaeNC2004group.id.11316 | Weighted median | 9 | 0.93 (0.74- 1.17) | 0.54 |
| genus.LachnospiraceaeNC2004group.id.11316 | Inverse variance weighted | 9 | 0.89 (0.74- 1.06) | 0.2 |
| genus.LachnospiraceaeNC2004group.id.11316 | Simple mode | 9 | 1.01 (0.71- 1.45) | 0.94 |
| genus.LachnospiraceaeNC2004group.id.11316 | Weighted mode | 9 | 1.01 (0.71- 1.42) | 0.97 |
| genus.LachnospiraceaeND3007group.id.11317 | MR Egger | 3 | 2.14 (0.00-10327.68) | 0.89 |
| genus.LachnospiraceaeND3007group.id.11317 | Weighted median | 3 | 1.41 (0.76- 2.61) | 0.27 |
| genus.LachnospiraceaeND3007group.id.11317 | Inverse variance weighted | 3 | 1.31 (0.79- 2.16) | 0.3 |
| genus.LachnospiraceaeND3007group.id.11317 | Simple mode | 3 | 1.46 (0.71- 3.00) | 0.41 |
| genus.LachnospiraceaeND3007group.id.11317 | Weighted mode | 3 | 1.47 (0.70- 3.06) | 0.41 |
| genus.LachnospiraceaeNK4A136group.id.11319 | MR Egger | 15 | 0.65 (0.44- 0.95) | 4.50E-02 |
| genus.LachnospiraceaeNK4A136group.id.11319 | Weighted median | 15 | 0.76 (0.57- 1.03) | 0.07 |
| genus.LachnospiraceaeNK4A136group.id.11319 | Inverse variance weighted | 15 | 0.80 (0.66- 0.97) | 2.40E-02 |
| genus.LachnospiraceaeNK4A136group.id.11319 | Simple mode | 15 | 0.78 (0.47- 1.27) | 0.33 |
| genus.LachnospiraceaeNK4A136group.id.11319 | Weighted mode | 15 | 0.76 (0.55- 1.06) | 0.13 |
| genus.LachnospiraceaeUCG001.id.11321 | MR Egger | 13 | 0.99 (0.42- 2.36) | 0.99 |
| genus.LachnospiraceaeUCG001.id.11321 | Weighted median | 13 | 1.21 (0.94- 1.56) | 0.13 |
| genus.LachnospiraceaeUCG001.id.11321 | Inverse variance weighted | 13 | 1.10 (0.90- 1.33) | 0.35 |
| genus.LachnospiraceaeUCG001.id.11321 | Simple mode | 13 | 1.20 (0.81- 1.76) | 0.38 |
| genus.LachnospiraceaeUCG001.id.11321 | Weighted mode | 13 | 1.20 (0.82- 1.74) | 0.36 |
| genus.LachnospiraceaeUCG004.id.11324 | MR Egger | 13 | 1.78 (0.54- 5.80) | 0.36 |
| genus.LachnospiraceaeUCG004.id.11324 | Weighted median | 13 | 0.79 (0.55- 1.13) | 0.2 |
| genus.LachnospiraceaeUCG004.id.11324 | Inverse variance weighted | 13 | 0.83 (0.62- 1.10) | 0.19 |
| genus.LachnospiraceaeUCG004.id.11324 | Simple mode | 13 | 1.32 (0.65- 2.69) | 0.45 |
| genus.LachnospiraceaeUCG004.id.11324 | Weighted mode | 13 | 0.69 (0.36- 1.34) | 0.3 |
| genus.LachnospiraceaeUCG008.id.11328 | MR Egger | 12 | 0.74 (0.34- 1.63) | 0.47 |
| genus.LachnospiraceaeUCG008.id.11328 | Weighted median | 12 | 0.98 (0.80- 1.18) | 0.8 |
| genus.LachnospiraceaeUCG008.id.11328 | Inverse variance weighted | 12 | 1.02 (0.87- 1.19) | 0.82 |
| genus.LachnospiraceaeUCG008.id.11328 | Simple mode | 12 | 0.97 (0.69- 1.36) | 0.85 |
| genus.LachnospiraceaeUCG008.id.11328 | Weighted mode | 12 | 0.97 (0.70- 1.34) | 0.86 |
| genus.LachnospiraceaeUCG010.id.11330 | MR Egger | 10 | 0.55 (0.26- 1.20) | 0.17 |
| genus.LachnospiraceaeUCG010.id.11330 | Weighted median | 10 | 0.75 (0.54- 1.05) | 0.09 |
| genus.LachnospiraceaeUCG010.id.11330 | Inverse variance weighted | 10 | 0.81 (0.63- 1.04) | 0.09 |
| genus.LachnospiraceaeUCG010.id.11330 | Simple mode | 10 | 0.66 (0.37- 1.16) | 0.18 |
| genus.LachnospiraceaeUCG010.id.11330 | Weighted mode | 10 | 0.67 (0.39- 1.15) | 0.18 |
| genus.Lactobacillus.id.1837 | MR Egger | 8 | 1.17 (0.76- 1.83) | 0.5 |
| genus.Lactobacillus.id.1837 | Weighted median | 8 | 1.05 (0.83- 1.32) | 0.7 |
| genus.Lactobacillus.id.1837 | Inverse variance weighted | 8 | 1.00 (0.84- 1.19) | 0.98 |
| genus.Lactobacillus.id.1837 | Simple mode | 8 | 1.14 (0.80- 1.61) | 0.49 |
| genus.Lactobacillus.id.1837 | Weighted mode | 8 | 1.08 (0.80- 1.44) | 0.63 |
| genus.Lactococcus.id.1851 | MR Egger | 8 | 0.64 (0.31- 1.31) | 0.27 |
| genus.Lactococcus.id.1851 | Weighted median | 8 | 1.17 (0.95- 1.43) | 0.14 |
| genus.Lactococcus.id.1851 | Inverse variance weighted | 8 | 1.14 (0.98- 1.33) | 0.1 |
| genus.Lactococcus.id.1851 | Simple mode | 8 | 1.30 (0.90- 1.88) | 0.21 |
| genus.Lactococcus.id.1851 | Weighted mode | 8 | 1.32 (0.92- 1.91) | 0.18 |
| genus.Marvinbryantia.id.2005 | MR Egger | 11 | 0.58 (0.23- 1.43) | 0.27 |
| genus.Marvinbryantia.id.2005 | Weighted median | 11 | 1.41 (1.02- 1.96) | 3.90E-02 |
| genus.Marvinbryantia.id.2005 | Inverse variance weighted | 11 | 1.38 (1.05- 1.82) | 2.00E-02 |
| genus.Marvinbryantia.id.2005 | Simple mode | 11 | 1.38 (0.82- 2.32) | 0.26 |
| genus.Marvinbryantia.id.2005 | Weighted mode | 11 | 1.47 (0.92- 2.34) | 0.13 |
| genus.Methanobrevibacter.id.123 | MR Egger | 6 | 1.36 (0.70- 2.66) | 0.42 |
| genus.Methanobrevibacter.id.123 | Weighted median | 6 | 1.03 (0.82- 1.30) | 0.77 |
| genus.Methanobrevibacter.id.123 | Inverse variance weighted | 6 | 1.11 (0.92- 1.32) | 0.27 |
| genus.Methanobrevibacter.id.123 | Simple mode | 6 | 0.96 (0.68- 1.36) | 0.83 |
| genus.Methanobrevibacter.id.123 | Weighted mode | 6 | 0.95 (0.66- 1.37) | 0.79 |
| genus.Odoribacter.id.952 | MR Egger | 7 | 1.46 (0.36- 5.92) | 0.62 |
| genus.Odoribacter.id.952 | Weighted median | 7 | 1.14 (0.75- 1.74) | 0.53 |
| genus.Odoribacter.id.952 | Inverse variance weighted | 7 | 0.94 (0.62- 1.44) | 0.78 |
| genus.Odoribacter.id.952 | Simple mode | 7 | 1.25 (0.66- 2.36) | 0.52 |
| genus.Odoribacter.id.952 | Weighted mode | 7 | 1.25 (0.70- 2.22) | 0.48 |
| genus.Olsenella.id.822 | MR Egger | 10 | 0.73 (0.49- 1.10) | 0.17 |
| genus.Olsenella.id.822 | Weighted median | 10 | 0.97 (0.83- 1.14) | 0.71 |
| genus.Olsenella.id.822 | Inverse variance weighted | 10 | 0.96 (0.85- 1.09) | 0.57 |
| genus.Olsenella.id.822 | Simple mode | 10 | 1.02 (0.81- 1.29) | 0.85 |
| genus.Olsenella.id.822 | Weighted mode | 10 | 0.94 (0.76- 1.17) | 0.62 |
| genus.Oscillibacter.id.2063 | MR Egger | 13 | 1.02 (0.53- 1.96) | 0.96 |
| genus.Oscillibacter.id.2063 | Weighted median | 13 | 0.95 (0.75- 1.21) | 0.7 |
| genus.Oscillibacter.id.2063 | Inverse variance weighted | 13 | 0.93 (0.79- 1.11) | 0.43 |
| genus.Oscillibacter.id.2063 | Simple mode | 13 | 1.01 (0.67- 1.53) | 0.96 |
| genus.Oscillibacter.id.2063 | Weighted mode | 13 | 1.01 (0.68- 1.50) | 0.97 |
| genus.Oscillospira.id.2064 | MR Egger | 9 | 1.33 (0.39- 4.56) | 0.66 |
| genus.Oscillospira.id.2064 | Weighted median | 9 | 1.01 (0.74- 1.39) | 0.93 |
| genus.Oscillospira.id.2064 | Inverse variance weighted | 9 | 0.90 (0.69- 1.17) | 0.42 |
| genus.Oscillospira.id.2064 | Simple mode | 9 | 1.07 (0.65- 1.74) | 0.8 |
| genus.Oscillospira.id.2064 | Weighted mode | 9 | 1.06 (0.67- 1.69) | 0.8 |
| genus.Oxalobacter.id.2978 | MR Egger | 11 | 1.24 (0.67- 2.31) | 0.52 |
| genus.Oxalobacter.id.2978 | Weighted median | 11 | 1.16 (0.98- 1.38) | 0.09 |
| genus.Oxalobacter.id.2978 | Inverse variance weighted | 11 | 1.09 (0.95- 1.24) | 0.2 |
| genus.Oxalobacter.id.2978 | Simple mode | 11 | 1.19 (0.91- 1.55) | 0.23 |
| genus.Oxalobacter.id.2978 | Weighted mode | 11 | 1.18 (0.91- 1.54) | 0.24 |
| genus.Parabacteroides.id.954 | MR Egger | 5 | 0.45 (0.04- 4.53) | 0.54 |
| genus.Parabacteroides.id.954 | Weighted median | 5 | 0.65 (0.41- 1.04) | 0.07 |
| genus.Parabacteroides.id.954 | Inverse variance weighted | 5 | 0.70 (0.48- 1.01) | 0.06 |
| genus.Parabacteroides.id.954 | Simple mode | 5 | 0.59 (0.32- 1.08) | 0.16 |
| genus.Parabacteroides.id.954 | Weighted mode | 5 | 0.59 (0.31- 1.12) | 0.18 |
| genus.Paraprevotella.id.962 | MR Egger | 13 | 0.84 (0.49- 1.47) | 0.56 |
| genus.Paraprevotella.id.962 | Weighted median | 13 | 1.08 (0.88- 1.32) | 0.45 |
| genus.Paraprevotella.id.962 | Inverse variance weighted | 13 | 1.07 (0.93- 1.24) | 0.35 |
| genus.Paraprevotella.id.962 | Simple mode | 13 | 1.17 (0.79- 1.73) | 0.45 |
| genus.Paraprevotella.id.962 | Weighted mode | 13 | 1.17 (0.81- 1.70) | 0.43 |
| genus.Parasutterella.id.2892 | MR Egger | 14 | 0.81 (0.50- 1.32) | 0.42 |
| genus.Parasutterella.id.2892 | Weighted median | 14 | 1.01 (0.80- 1.28) | 0.95 |
| genus.Parasutterella.id.2892 | Inverse variance weighted | 14 | 1.03 (0.87- 1.23) | 0.7 |
| genus.Parasutterella.id.2892 | Simple mode | 14 | 1.01 (0.69- 1.50) | 0.94 |
| genus.Parasutterella.id.2892 | Weighted mode | 14 | 0.97 (0.67- 1.39) | 0.86 |
| genus.Peptococcus.id.2037 | MR Egger | 14 | 1.32 (0.79- 2.22) | 0.31 |
| genus.Peptococcus.id.2037 | Weighted median | 14 | 0.98 (0.81- 1.19) | 0.85 |
| genus.Peptococcus.id.2037 | Inverse variance weighted | 14 | 0.96 (0.84- 1.09) | 0.52 |
| genus.Peptococcus.id.2037 | Simple mode | 14 | 1.06 (0.76- 1.49) | 0.73 |
| genus.Peptococcus.id.2037 | Weighted mode | 14 | 1.04 (0.76- 1.40) | 0.82 |
| genus.Phascolarctobacterium.id.2168 | MR Egger | 9 | 0.69 (0.24- 1.98) | 0.51 |
| genus.Phascolarctobacterium.id.2168 | Weighted median | 9 | 0.93 (0.68- 1.28) | 0.66 |
| genus.Phascolarctobacterium.id.2168 | Inverse variance weighted | 9 | 1.05 (0.83- 1.33) | 0.69 |
| genus.Phascolarctobacterium.id.2168 | Simple mode | 9 | 0.87 (0.53- 1.41) | 0.58 |
| genus.Phascolarctobacterium.id.2168 | Weighted mode | 9 | 0.86 (0.52- 1.43) | 0.58 |
| genus.Prevotella7.id.11182 | MR Egger | 11 | 0.96 (0.46- 1.99) | 0.92 |
| genus.Prevotella7.id.11182 | Weighted median | 11 | 0.91 (0.77- 1.07) | 0.23 |
| genus.Prevotella7.id.11182 | Inverse variance weighted | 11 | 0.93 (0.82- 1.05) | 0.25 |
| genus.Prevotella7.id.11182 | Simple mode | 11 | 0.83 (0.63- 1.08) | 0.2 |
| genus.Prevotella7.id.11182 | Weighted mode | 11 | 0.83 (0.64- 1.07) | 0.19 |
| genus.Prevotella9.id.11183 | MR Egger | 15 | 0.78 (0.49- 1.24) | 0.31 |
| genus.Prevotella9.id.11183 | Weighted median | 15 | 0.95 (0.76- 1.20) | 0.68 |
| genus.Prevotella9.id.11183 | Inverse variance weighted | 15 | 0.94 (0.80- 1.10) | 0.45 |
| genus.Prevotella9.id.11183 | Simple mode | 15 | 1.14 (0.78- 1.69) | 0.51 |
| genus.Prevotella9.id.11183 | Weighted mode | 15 | 1.10 (0.77- 1.57) | 0.6 |
| genus.RikenellaceaeRC9gutgroup.id.11191 | MR Egger | 12 | 0.84 (0.41- 1.69) | 0.63 |
| genus.RikenellaceaeRC9gutgroup.id.11191 | Weighted median | 12 | 1.07 (0.92- 1.24) | 0.4 |
| genus.RikenellaceaeRC9gutgroup.id.11191 | Inverse variance weighted | 12 | 1.05 (0.94- 1.18) | 0.4 |
| genus.RikenellaceaeRC9gutgroup.id.11191 | Simple mode | 12 | 1.13 (0.88- 1.44) | 0.36 |
| genus.RikenellaceaeRC9gutgroup.id.11191 | Weighted mode | 12 | 1.12 (0.87- 1.45) | 0.4 |
| genus.Romboutsia.id.11347 | MR Egger | 14 | 1.19 (0.49- 2.91) | 0.71 |
| genus.Romboutsia.id.11347 | Weighted median | 14 | 0.98 (0.70- 1.36) | 0.89 |
| genus.Romboutsia.id.11347 | Inverse variance weighted | 14 | 0.92 (0.69- 1.24) | 0.6 |
| genus.Romboutsia.id.11347 | Simple mode | 14 | 1.11 (0.57- 2.16) | 0.77 |
| genus.Romboutsia.id.11347 | Weighted mode | 14 | 0.96 (0.53- 1.75) | 0.89 |
| genus.Roseburia.id.2012 | MR Egger | 14 | 0.66 (0.32- 1.39) | 0.3 |
| genus.Roseburia.id.2012 | Weighted median | 14 | 0.93 (0.67- 1.27) | 0.64 |
| genus.Roseburia.id.2012 | Inverse variance weighted | 14 | 1.09 (0.85- 1.39) | 0.51 |
| genus.Roseburia.id.2012 | Simple mode | 14 | 0.86 (0.54- 1.38) | 0.54 |
| genus.Roseburia.id.2012 | Weighted mode | 14 | 0.85 (0.54- 1.34) | 0.49 |
| genus.Ruminiclostridium5.id.11355 | MR Egger | 11 | 0.62 (0.20- 1.88) | 0.42 |
| genus.Ruminiclostridium5.id.11355 | Weighted median | 11 | 0.82 (0.57- 1.17) | 0.28 |
| genus.Ruminiclostridium5.id.11355 | Inverse variance weighted | 11 | 0.87 (0.67- 1.14) | 0.32 |
| genus.Ruminiclostridium5.id.11355 | Simple mode | 11 | 0.81 (0.47- 1.38) | 0.46 |
| genus.Ruminiclostridium5.id.11355 | Weighted mode | 11 | 0.80 (0.46- 1.39) | 0.45 |
| genus.Ruminiclostridium6.id.11356 | MR Egger | 15 | 0.97 (0.59- 1.62) | 0.92 |
| genus.Ruminiclostridium6.id.11356 | Weighted median | 15 | 1.03 (0.78- 1.36) | 0.84 |
| genus.Ruminiclostridium6.id.11356 | Inverse variance weighted | 15 | 1.05 (0.85- 1.29) | 0.66 |
| genus.Ruminiclostridium6.id.11356 | Simple mode | 15 | 1.07 (0.68- 1.67) | 0.77 |
| genus.Ruminiclostridium6.id.11356 | Weighted mode | 15 | 0.99 (0.65- 1.51) | 0.98 |
| genus.Ruminiclostridium9.id.11357 | MR Egger | 9 | 0.81 (0.18- 3.59) | 0.78 |
| genus.Ruminiclostridium9.id.11357 | Weighted median | 9 | 1.12 (0.77- 1.64) | 0.55 |
| genus.Ruminiclostridium9.id.11357 | Inverse variance weighted | 9 | 1.08 (0.81- 1.46) | 0.59 |
| genus.Ruminiclostridium9.id.11357 | Simple mode | 9 | 1.11 (0.63- 1.94) | 0.73 |
| genus.Ruminiclostridium9.id.11357 | Weighted mode | 9 | 1.11 (0.63- 1.96) | 0.74 |
| genus.RuminococcaceaeNK4A214group.id.11358 | MR Egger | 14 | 2.16 (1.04- 4.48) | 0.06 |
| genus.RuminococcaceaeNK4A214group.id.11358 | Weighted median | 14 | 1.24 (0.90- 1.71) | 0.19 |
| genus.RuminococcaceaeNK4A214group.id.11358 | Inverse variance weighted | 14 | 1.33 (1.07- 1.67) | 1.10E-02 |
| genus.RuminococcaceaeNK4A214group.id.11358 | Simple mode | 14 | 1.08 (0.64- 1.83) | 0.77 |
| genus.RuminococcaceaeNK4A214group.id.11358 | Weighted mode | 14 | 1.19 (0.78- 1.82) | 0.44 |
| genus.RuminococcaceaeUCG002.id.11360 | MR Egger | 20 | 1.02 (0.61- 1.69) | 0.94 |
| genus.RuminococcaceaeUCG002.id.11360 | Weighted median | 20 | 1.16 (0.88- 1.52) | 0.29 |
| genus.RuminococcaceaeUCG002.id.11360 | Inverse variance weighted | 20 | 1.07 (0.89- 1.30) | 0.46 |
| genus.RuminococcaceaeUCG002.id.11360 | Simple mode | 20 | 1.38 (0.88- 2.17) | 0.17 |
| genus.RuminococcaceaeUCG002.id.11360 | Weighted mode | 20 | 1.27 (0.89- 1.81) | 0.2 |
| genus.RuminococcaceaeUCG003.id.11361 | MR Egger | 12 | 0.96 (0.47- 1.95) | 0.91 |
| genus.RuminococcaceaeUCG003.id.11361 | Weighted median | 12 | 1.04 (0.79- 1.37) | 0.78 |
| genus.RuminococcaceaeUCG003.id.11361 | Inverse variance weighted | 12 | 1.10 (0.88- 1.37) | 0.4 |
| genus.RuminococcaceaeUCG003.id.11361 | Simple mode | 12 | 1.02 (0.64- 1.63) | 0.94 |
| genus.RuminococcaceaeUCG003.id.11361 | Weighted mode | 12 | 1.04 (0.68- 1.58) | 0.87 |
| genus.RuminococcaceaeUCG004.id.11362 | MR Egger | 11 | 1.50 (0.50- 4.49) | 0.49 |
| genus.RuminococcaceaeUCG004.id.11362 | Weighted median | 11 | 1.10 (0.84- 1.43) | 0.49 |
| genus.RuminococcaceaeUCG004.id.11362 | Inverse variance weighted | 11 | 1.13 (0.93- 1.38) | 0.22 |
| genus.RuminococcaceaeUCG004.id.11362 | Simple mode | 11 | 1.17 (0.76- 1.80) | 0.49 |
| genus.RuminococcaceaeUCG004.id.11362 | Weighted mode | 11 | 1.18 (0.78- 1.77) | 0.45 |
| genus.RuminococcaceaeUCG005.id.11363 | MR Egger | 14 | 0.79 (0.43- 1.43) | 0.45 |
| genus.RuminococcaceaeUCG005.id.11363 | Weighted median | 14 | 1.06 (0.78- 1.43) | 0.72 |
| genus.RuminococcaceaeUCG005.id.11363 | Inverse variance weighted | 14 | 1.01 (0.81- 1.26) | 0.92 |
| genus.RuminococcaceaeUCG005.id.11363 | Simple mode | 14 | 0.86 (0.53- 1.40) | 0.56 |
| genus.RuminococcaceaeUCG005.id.11363 | Weighted mode | 14 | 1.09 (0.69- 1.70) | 0.72 |
| genus.RuminococcaceaeUCG009.id.11366 | MR Egger | 11 | 1.07 (0.55- 2.07) | 0.84 |
| genus.RuminococcaceaeUCG009.id.11366 | Weighted median | 11 | 1.11 (0.88- 1.41) | 0.39 |
| genus.RuminococcaceaeUCG009.id.11366 | Inverse variance weighted | 11 | 1.07 (0.91- 1.28) | 0.41 |
| genus.RuminococcaceaeUCG009.id.11366 | Simple mode | 11 | 1.11 (0.76- 1.62) | 0.61 |
| genus.RuminococcaceaeUCG009.id.11366 | Weighted mode | 11 | 1.08 (0.75- 1.55) | 0.69 |
| genus.RuminococcaceaeUCG010.id.11367 | MR Egger | 6 | 0.97 (0.35- 2.74) | 0.96 |
| genus.RuminococcaceaeUCG010.id.11367 | Weighted median | 6 | 1.19 (0.81- 1.74) | 0.38 |
| genus.RuminococcaceaeUCG010.id.11367 | Inverse variance weighted | 6 | 1.09 (0.78- 1.54) | 0.6 |
| genus.RuminococcaceaeUCG010.id.11367 | Simple mode | 6 | 1.34 (0.81- 2.22) | 0.3 |
| genus.RuminococcaceaeUCG010.id.11367 | Weighted mode | 6 | 1.20 (0.77- 1.87) | 0.46 |
| genus.RuminococcaceaeUCG011.id.11368 | MR Egger | 8 | 0.74 (0.30- 1.85) | 0.54 |
| genus.RuminococcaceaeUCG011.id.11368 | Weighted median | 8 | 1.09 (0.88- 1.36) | 0.41 |
| genus.RuminococcaceaeUCG011.id.11368 | Inverse variance weighted | 8 | 1.07 (0.90- 1.28) | 0.44 |
| genus.RuminococcaceaeUCG011.id.11368 | Simple mode | 8 | 1.31 (0.88- 1.96) | 0.23 |
| genus.RuminococcaceaeUCG011.id.11368 | Weighted mode | 8 | 0.85 (0.57- 1.28) | 0.47 |
| genus.RuminococcaceaeUCG013.id.11370 | MR Egger | 11 | 0.55 (0.27- 1.11) | 0.13 |
| genus.RuminococcaceaeUCG013.id.11370 | Weighted median | 11 | 0.83 (0.60- 1.14) | 0.25 |
| genus.RuminococcaceaeUCG013.id.11370 | Inverse variance weighted | 11 | 0.86 (0.67- 1.09) | 0.21 |
| genus.RuminococcaceaeUCG013.id.11370 | Simple mode | 11 | 0.83 (0.49- 1.41) | 0.51 |
| genus.RuminococcaceaeUCG013.id.11370 | Weighted mode | 11 | 0.85 (0.51- 1.43) | 0.56 |
| genus.RuminococcaceaeUCG014.id.11371 | MR Egger | 10 | 0.98 (0.41- 2.30) | 0.96 |
| genus.RuminococcaceaeUCG014.id.11371 | Weighted median | 10 | 0.90 (0.64- 1.27) | 0.55 |
| genus.RuminococcaceaeUCG014.id.11371 | Inverse variance weighted | 10 | 0.92 (0.64- 1.30) | 0.62 |
| genus.RuminococcaceaeUCG014.id.11371 | Simple mode | 10 | 1.03 (0.50- 2.13) | 0.93 |
| genus.RuminococcaceaeUCG014.id.11371 | Weighted mode | 10 | 0.91 (0.63- 1.30) | 0.61 |
| genus.Ruminococcus1.id.11373 | MR Egger | 10 | 1.58 (0.82- 3.04) | 0.21 |
| genus.Ruminococcus1.id.11373 | Weighted median | 10 | 1.02 (0.74- 1.41) | 0.89 |
| genus.Ruminococcus1.id.11373 | Inverse variance weighted | 10 | 0.96 (0.75- 1.22) | 0.73 |
| genus.Ruminococcus1.id.11373 | Simple mode | 10 | 1.14 (0.69- 1.87) | 0.63 |
| genus.Ruminococcus1.id.11373 | Weighted mode | 10 | 1.12 (0.74- 1.70) | 0.61 |
| genus.Ruminococcus2.id.11374 | MR Egger | 15 | 0.91 (0.57- 1.45) | 0.7 |
| genus.Ruminococcus2.id.11374 | Weighted median | 15 | 0.88 (0.68- 1.15) | 0.36 |
| genus.Ruminococcus2.id.11374 | Inverse variance weighted | 15 | 0.94 (0.78- 1.13) | 0.52 |
| genus.Ruminococcus2.id.11374 | Simple mode | 15 | 0.84 (0.56- 1.25) | 0.4 |
| genus.Ruminococcus2.id.11374 | Weighted mode | 15 | 0.87 (0.63- 1.20) | 0.41 |
| genus.Sellimonas.id.14369 | MR Egger | 9 | 0.74 (0.38- 1.44) | 0.41 |
| genus.Sellimonas.id.14369 | Weighted median | 9 | 0.87 (0.74- 1.03) | 0.1 |
| genus.Sellimonas.id.14369 | Inverse variance weighted | 9 | 0.90 (0.80- 1.02) | 0.09 |
| genus.Sellimonas.id.14369 | Simple mode | 9 | 0.87 (0.68- 1.11) | 0.3 |
| genus.Sellimonas.id.14369 | Weighted mode | 9 | 0.87 (0.66- 1.15) | 0.36 |
| genus.Senegalimassilia.id.11160 | MR Egger | 5 | 0.92 (0.24- 3.53) | 0.91 |
| genus.Senegalimassilia.id.11160 | Weighted median | 5 | 0.99 (0.69- 1.42) | 0.95 |
| genus.Senegalimassilia.id.11160 | Inverse variance weighted | 5 | 1.01 (0.75- 1.37) | 0.94 |
| genus.Senegalimassilia.id.11160 | Simple mode | 5 | 0.96 (0.55- 1.68) | 0.9 |
| genus.Senegalimassilia.id.11160 | Weighted mode | 5 | 0.98 (0.59- 1.65) | 0.96 |
| genus.Slackia.id.825 | MR Egger | 6 | 1.49 (0.17- 13.33) | 0.74 |
| genus.Slackia.id.825 | Weighted median | 6 | 1.05 (0.77- 1.44) | 0.74 |
| genus.Slackia.id.825 | Inverse variance weighted | 6 | 1.04 (0.77- 1.41) | 0.81 |
| genus.Slackia.id.825 | Simple mode | 6 | 1.07 (0.66- 1.76) | 0.78 |
| genus.Slackia.id.825 | Weighted mode | 6 | 1.05 (0.66- 1.67) | 0.85 |
| genus.Streptococcus.id.1853 | MR Egger | 14 | 1.72 (0.69- 4.31) | 0.27 |
| genus.Streptococcus.id.1853 | Weighted median | 14 | 1.18 (0.84- 1.64) | 0.34 |
| genus.Streptococcus.id.1853 | Inverse variance weighted | 14 | 1.08 (0.84- 1.38) | 0.54 |
| genus.Streptococcus.id.1853 | Simple mode | 14 | 1.33 (0.68- 2.60) | 0.42 |
| genus.Streptococcus.id.1853 | Weighted mode | 14 | 1.36 (0.75- 2.46) | 0.33 |
| genus.Subdoligranulum.id.2070 | MR Egger | 11 | 1.02 (0.50- 2.05) | 0.96 |
| genus.Subdoligranulum.id.2070 | Weighted median | 11 | 0.86 (0.62- 1.21) | 0.38 |
| genus.Subdoligranulum.id.2070 | Inverse variance weighted | 11 | 0.98 (0.76- 1.27) | 0.88 |
| genus.Subdoligranulum.id.2070 | Simple mode | 11 | 0.78 (0.45- 1.36) | 0.4 |
| genus.Subdoligranulum.id.2070 | Weighted mode | 11 | 0.77 (0.47- 1.26) | 0.32 |
| genus.Sutterella.id.2896 | MR Egger | 12 | 0.75 (0.29- 1.95) | 0.57 |
| genus.Sutterella.id.2896 | Weighted median | 12 | 1.05 (0.77- 1.44) | 0.75 |
| genus.Sutterella.id.2896 | Inverse variance weighted | 12 | 1.08 (0.86- 1.35) | 0.51 |
| genus.Sutterella.id.2896 | Simple mode | 12 | 0.98 (0.61- 1.57) | 0.94 |
| genus.Sutterella.id.2896 | Weighted mode | 12 | 0.99 (0.61- 1.59) | 0.96 |
| genus.Terrisporobacter.id.11348 | MR Egger | 5 | 1.72 (0.44- 6.74) | 0.49 |
| genus.Terrisporobacter.id.11348 | Weighted median | 5 | 1.13 (0.77- 1.67) | 0.53 |
| genus.Terrisporobacter.id.11348 | Inverse variance weighted | 5 | 0.81 (0.51- 1.30) | 0.38 |
| genus.Terrisporobacter.id.11348 | Simple mode | 5 | 1.20 (0.71- 2.04) | 0.53 |
| genus.Terrisporobacter.id.11348 | Weighted mode | 5 | 1.18 (0.76- 1.84) | 0.5 |
| genus.Turicibacter.id.2162 | MR Egger | 9 | 1.51 (0.59- 3.84) | 0.42 |
| genus.Turicibacter.id.2162 | Weighted median | 9 | 1.03 (0.77- 1.37) | 0.87 |
| genus.Turicibacter.id.2162 | Inverse variance weighted | 9 | 1.13 (0.91- 1.42) | 0.26 |
| genus.Turicibacter.id.2162 | Simple mode | 9 | 1.44 (0.88- 2.36) | 0.18 |
| genus.Turicibacter.id.2162 | Weighted mode | 9 | 1.01 (0.63- 1.61) | 0.97 |
| genus.Tyzzerella3.id.11335 | MR Egger | 12 | 1.11 (0.50- 2.45) | 0.81 |
| genus.Tyzzerella3.id.11335 | Weighted median | 12 | 1.08 (0.89- 1.30) | 0.43 |
| genus.Tyzzerella3.id.11335 | Inverse variance weighted | 12 | 1.13 (0.98- 1.30) | 0.11 |
| genus.Tyzzerella3.id.11335 | Simple mode | 12 | 1.05 (0.79- 1.39) | 0.77 |
| genus.Tyzzerella3.id.11335 | Weighted mode | 12 | 1.06 (0.80- 1.41) | 0.67 |
| genus.Veillonella.id.2198 | MR Egger | 5 | 16.06 (0.02-13800.46) | 0.48 |
| genus.Veillonella.id.2198 | Weighted median | 5 | 0.96 (0.67- 1.36) | 0.8 |
| genus.Veillonella.id.2198 | Inverse variance weighted | 5 | 0.93 (0.70- 1.25) | 0.64 |
| genus.Veillonella.id.2198 | Simple mode | 5 | 0.94 (0.58- 1.51) | 0.8 |
| genus.Veillonella.id.2198 | Weighted mode | 5 | 0.94 (0.58- 1.53) | 0.83 |
| genus.Victivallis.id.2256 | MR Egger | 11 | 1.02 (0.46- 2.25) | 0.96 |
| genus.Victivallis.id.2256 | Weighted median | 11 | 0.97 (0.82- 1.14) | 0.68 |
| genus.Victivallis.id.2256 | Inverse variance weighted | 11 | 1.03 (0.91- 1.16) | 0.64 |
| genus.Victivallis.id.2256 | Simple mode | 11 | 0.93 (0.72- 1.21) | 0.62 |
| genus.Victivallis.id.2256 | Weighted mode | 11 | 0.94 (0.72- 1.21) | 0.62 |

| **Table S4** The heterogeneity of the gut microbiota instrumental variables. | | |  |  |
| --- | --- | --- | --- | --- |
| **Bacterial taxa (exposure)** | **Method** | **Cochran's Q** | **DF** | **P-value** |
| genus..Clostridiuminnocuumgroup.id.14397 | MR Egger | 10.49413526 | 6 | 0.105326607 |
| genus..Clostridiuminnocuumgroup.id.14397 | Inverse variance weighted | 10.98989666 | 7 | 0.139060331 |
| genus..Eubacteriumbrachygroup.id.11296 | MR Egger | 5.068606993 | 8 | 0.750218792 |
| genus..Eubacteriumbrachygroup.id.11296 | Inverse variance weighted | 5.69612288 | 9 | 0.76990407 |
| genus..Eubacteriumcoprostanoligenesgroup.id.11375 | MR Egger | 15.65993263 | 10 | 0.109789852 |
| genus..Eubacteriumcoprostanoligenesgroup.id.11375 | Inverse variance weighted | 17.66535502 | 11 | 0.089677461 |
| genus..Eubacteriumeligensgroup.id.14372 | MR Egger | 3.003164003 | 5 | 0.699498009 |
| genus..Eubacteriumeligensgroup.id.14372 | Inverse variance weighted | 3.013216047 | 6 | 0.807186258 |
| genus..Eubacteriumfissicatenagroup.id.14373 | MR Egger | 9.299279678 | 7 | 0.231877177 |
| genus..Eubacteriumfissicatenagroup.id.14373 | Inverse variance weighted | 10.43514762 | 8 | 0.235802014 |
| genus..Eubacteriumhalliigroup.id.11338 | MR Egger | 5.338171204 | 12 | 0.945719936 |
| genus..Eubacteriumhalliigroup.id.11338 | Inverse variance weighted | 6.578309276 | 13 | 0.922556972 |
| genus..Eubacteriumnodatumgroup.id.11297 | MR Egger | 5.008719212 | 9 | 0.833547523 |
| genus..Eubacteriumnodatumgroup.id.11297 | Inverse variance weighted | 5.132638593 | 10 | 0.882143215 |
| genus..Eubacteriumoxidoreducensgroup.id.11339 | MR Egger | 1.839784409 | 3 | 0.606315906 |
| genus..Eubacteriumoxidoreducensgroup.id.11339 | Inverse variance weighted | 3.271255821 | 4 | 0.513499378 |
| genus..Eubacteriumrectalegroup.id.14374 | MR Egger | 11.55052371 | 7 | 0.116343082 |
| genus..Eubacteriumrectalegroup.id.14374 | Inverse variance weighted | 12.71157835 | 8 | 0.122166135 |
| genus..Eubacteriumruminantiumgroup.id.11340 | MR Egger | 14.2050535 | 16 | 0.58344086 |
| genus..Eubacteriumruminantiumgroup.id.11340 | Inverse variance weighted | 15.05473641 | 17 | 0.591538928 |
| genus..Eubacteriumventriosumgroup.id.11341 | MR Egger | 7.510856513 | 13 | 0.8739446 |
| genus..Eubacteriumventriosumgroup.id.11341 | Inverse variance weighted | 8.43398321 | 14 | 0.865514439 |
| genus..Eubacteriumxylanophilumgroup.id.14375 | MR Egger | 4.472531085 | 7 | 0.724022422 |
| genus..Eubacteriumxylanophilumgroup.id.14375 | Inverse variance weighted | 5.258370157 | 8 | 0.729637909 |
| genus..Ruminococcusgauvreauiigroup.id.11342 | MR Egger | 13.09967553 | 9 | 0.158147678 |
| genus..Ruminococcusgauvreauiigroup.id.11342 | Inverse variance weighted | 13.49803556 | 10 | 0.197142872 |
| genus..Ruminococcusgnavusgroup.id.14376 | MR Egger | 11.59208016 | 9 | 0.237294515 |
| genus..Ruminococcusgnavusgroup.id.14376 | Inverse variance weighted | 11.5924448 | 10 | 0.313257939 |
| genus..Ruminococcustorquesgroup.id.14377 | MR Egger | 13.00943202 | 6 | 0.04288641 |
| genus..Ruminococcustorquesgroup.id.14377 | Inverse variance weighted | 18.49345102 | 7 | 0.009931335 |
| genus.Actinomyces.id.423 | MR Egger | 5.865939255 | 5 | 0.319482984 |
| genus.Actinomyces.id.423 | Inverse variance weighted | 5.982015832 | 6 | 0.4252077 |
| genus.Adlercreutzia.id.812 | MR Egger | 7.273478775 | 6 | 0.296294895 |
| genus.Adlercreutzia.id.812 | Inverse variance weighted | 10.01018556 | 7 | 0.187996996 |
| genus.Akkermansia.id.4037 | MR Egger | 15.40283286 | 10 | 0.118050888 |
| genus.Akkermansia.id.4037 | Inverse variance weighted | 15.40404779 | 11 | 0.16473643 |
| genus.Alistipes.id.968 | MR Egger | 3.922486974 | 10 | 0.950775979 |
| genus.Alistipes.id.968 | Inverse variance weighted | 4.324560295 | 11 | 0.959443678 |
| genus.Allisonella.id.2174 | MR Egger | 3.005905882 | 6 | 0.808105215 |
| genus.Allisonella.id.2174 | Inverse variance weighted | 3.537161859 | 7 | 0.831273809 |
| genus.Alloprevotella.id.961 | MR Egger | 14.25715226 | 3 | 0.002575221 |
| genus.Alloprevotella.id.961 | Inverse variance weighted | 16.58455129 | 4 | 0.002327194 |
| genus.Anaerofilum.id.2053 | MR Egger | 10.11218968 | 8 | 0.257239832 |
| genus.Anaerofilum.id.2053 | Inverse variance weighted | 11.36609289 | 9 | 0.251447207 |
| genus.Anaerostipes.id.1991 | MR Egger | 12.81740809 | 11 | 0.305426925 |
| genus.Anaerostipes.id.1991 | Inverse variance weighted | 13.15788649 | 12 | 0.357670076 |
| genus.Anaerotruncus.id.2054 | MR Egger | 11.99701279 | 11 | 0.363867802 |
| genus.Anaerotruncus.id.2054 | Inverse variance weighted | 15.48196178 | 12 | 0.216131077 |
| genus.Bacteroides.id.918 | MR Egger | 2.199212222 | 6 | 0.900495593 |
| genus.Bacteroides.id.918 | Inverse variance weighted | 2.282290017 | 7 | 0.9425803 |
| genus.Barnesiella.id.944 | MR Egger | 7.90857776 | 10 | 0.637766589 |
| genus.Barnesiella.id.944 | Inverse variance weighted | 8.551033288 | 11 | 0.663256829 |
| genus.Bifidobacterium.id.436 | MR Egger | 18.09294609 | 17 | 0.382999972 |
| genus.Bifidobacterium.id.436 | Inverse variance weighted | 19.03671556 | 18 | 0.389565818 |
| genus.Bilophila.id.3170 | MR Egger | 8.595927308 | 11 | 0.659134296 |
| genus.Bilophila.id.3170 | Inverse variance weighted | 8.67461849 | 12 | 0.730442313 |
| genus.Blautia.id.1992 | MR Egger | 16.69493577 | 10 | 0.081392757 |
| genus.Blautia.id.1992 | Inverse variance weighted | 16.96724981 | 11 | 0.10884807 |
| genus.Butyricicoccus.id.2055 | MR Egger | 3.952221516 | 6 | 0.683142221 |
| genus.Butyricicoccus.id.2055 | Inverse variance weighted | 4.457630252 | 7 | 0.725813119 |
| genus.Butyricimonas.id.945 | MR Egger | 4.935323251 | 11 | 0.934258295 |
| genus.Butyricimonas.id.945 | Inverse variance weighted | 6.358276932 | 12 | 0.896955699 |
| genus.Butyrivibrio.id.1993 | MR Egger | 9.135532454 | 13 | 0.762632042 |
| genus.Butyrivibrio.id.1993 | Inverse variance weighted | 10.26563431 | 14 | 0.742516112 |
| genus.CandidatusSoleaferrea.id.11350 | MR Egger | 8.464136744 | 7 | 0.293449243 |
| genus.CandidatusSoleaferrea.id.11350 | Inverse variance weighted | 8.936924783 | 8 | 0.347644849 |
| genus.Catenibacterium.id.2153 | MR Egger | 0.131938566 | 2 | 0.93615962 |
| genus.Catenibacterium.id.2153 | Inverse variance weighted | 0.691269749 | 3 | 0.875255446 |
| genus.ChristensenellaceaeR.7group.id.11283 | MR Egger | 4.065959939 | 6 | 0.667750512 |
| genus.ChristensenellaceaeR.7group.id.11283 | Inverse variance weighted | 4.349719754 | 7 | 0.73872973 |
| genus.Clostridiumsensustricto1.id.1873 | MR Egger | 3.248939349 | 4 | 0.517062859 |
| genus.Clostridiumsensustricto1.id.1873 | Inverse variance weighted | 3.670176036 | 5 | 0.597808408 |
| genus.Collinsella.id.815 | MR Egger | 6.583474898 | 8 | 0.582164172 |
| genus.Collinsella.id.815 | Inverse variance weighted | 8.740607632 | 9 | 0.461554825 |
| genus.Coprobacter.id.949 | MR Egger | 10.25138456 | 9 | 0.330520274 |
| genus.Coprobacter.id.949 | Inverse variance weighted | 11.86198953 | 10 | 0.294399246 |
| genus.Coprococcus1.id.11301 | MR Egger | 11.10474574 | 10 | 0.349415815 |
| genus.Coprococcus1.id.11301 | Inverse variance weighted | 11.19340892 | 11 | 0.427205318 |
| genus.Coprococcus2.id.11302 | MR Egger | 7.793147172 | 7 | 0.351187046 |
| genus.Coprococcus2.id.11302 | Inverse variance weighted | 7.868030728 | 8 | 0.446466453 |
| genus.Coprococcus3.id.11303 | MR Egger | 3.745720147 | 8 | 0.879305911 |
| genus.Coprococcus3.id.11303 | Inverse variance weighted | 5.371932497 | 9 | 0.800756029 |
| genus.DefluviitaleaceaeUCG011.id.11287 | MR Egger | 10.71938668 | 8 | 0.218111609 |
| genus.DefluviitaleaceaeUCG011.id.11287 | Inverse variance weighted | 11.94112878 | 9 | 0.216648572 |
| genus.Desulfovibrio.id.3173 | MR Egger | 6.505900166 | 9 | 0.688410363 |
| genus.Desulfovibrio.id.3173 | Inverse variance weighted | 8.454794686 | 10 | 0.584503953 |
| genus.Dialister.id.2183 | MR Egger | 8.317222622 | 9 | 0.502520873 |
| genus.Dialister.id.2183 | Inverse variance weighted | 10.54795926 | 10 | 0.393801974 |
| genus.Dorea.id.1997 | MR Egger | 7.955928665 | 9 | 0.5385936 |
| genus.Dorea.id.1997 | Inverse variance weighted | 8.667750247 | 10 | 0.563898582 |
| genus.Eggerthella.id.819 | MR Egger | 4.129015248 | 7 | 0.764803282 |
| genus.Eggerthella.id.819 | Inverse variance weighted | 7.67462633 | 8 | 0.46588128 |
| genus.Eisenbergiella.id.11304 | MR Egger | 11.16346501 | 9 | 0.264670505 |
| genus.Eisenbergiella.id.11304 | Inverse variance weighted | 11.81432232 | 10 | 0.297675456 |
| genus.Enterorhabdus.id.820 | MR Egger | 2.098634685 | 4 | 0.717623222 |
| genus.Enterorhabdus.id.820 | Inverse variance weighted | 6.622755536 | 5 | 0.250241586 |
| genus.Erysipelatoclostridium.id.11381 | MR Egger | 18.88240798 | 14 | 0.169491326 |
| genus.Erysipelatoclostridium.id.11381 | Inverse variance weighted | 22.46049972 | 15 | 0.096285638 |
| genus.ErysipelotrichaceaeUCG003.id.11384 | MR Egger | 9.501125331 | 15 | 0.849893301 |
| genus.ErysipelotrichaceaeUCG003.id.11384 | Inverse variance weighted | 9.746507551 | 16 | 0.879526674 |
| genus.Escherichia.Shigella.id.3504 | MR Egger | 10.54550446 | 8 | 0.228804341 |
| genus.Escherichia.Shigella.id.3504 | Inverse variance weighted | 18.63053725 | 9 | 0.028524192 |
| genus.Faecalibacterium.id.2057 | MR Egger | 13.8500964 | 8 | 0.085757244 |
| genus.Faecalibacterium.id.2057 | Inverse variance weighted | 14.49837377 | 9 | 0.105668763 |
| genus.FamilyXIIIAD3011group.id.11293 | MR Egger | 15.71617864 | 11 | 0.151998988 |
| genus.FamilyXIIIAD3011group.id.11293 | Inverse variance weighted | 16.32571377 | 12 | 0.176769052 |
| genus.FamilyXIIIUCG001.id.11294 | MR Egger | 5.378398208 | 6 | 0.496274016 |
| genus.FamilyXIIIUCG001.id.11294 | Inverse variance weighted | 5.880660029 | 7 | 0.553751292 |
| genus.Flavonifractor.id.2059 | MR Egger | 3.223221703 | 4 | 0.521188428 |
| genus.Flavonifractor.id.2059 | Inverse variance weighted | 3.764309065 | 5 | 0.583823574 |
| genus.Fusicatenibacter.id.11305 | MR Egger | 16.54936013 | 17 | 0.485277911 |
| genus.Fusicatenibacter.id.11305 | Inverse variance weighted | 17.01518225 | 18 | 0.522061439 |
| genus.Gordonibacter.id.821 | MR Egger | 10.35373 | 9 | 0.322614879 |
| genus.Gordonibacter.id.821 | Inverse variance weighted | 11.13512704 | 10 | 0.347087774 |
| genus.Haemophilus.id.3698 | MR Egger | 3.983158873 | 7 | 0.781715127 |
| genus.Haemophilus.id.3698 | Inverse variance weighted | 4.314329118 | 8 | 0.827708671 |
| genus.Holdemanella.id.11393 | MR Egger | 7.302305815 | 9 | 0.605676886 |
| genus.Holdemanella.id.11393 | Inverse variance weighted | 7.369141184 | 10 | 0.690196364 |
| genus.Holdemania.id.2157 | MR Egger | 8.981185483 | 12 | 0.704536602 |
| genus.Holdemania.id.2157 | Inverse variance weighted | 9.067867395 | 13 | 0.767798706 |
| genus.Howardella.id.2000 | MR Egger | 22.74962388 | 7 | 0.0018844 |
| genus.Howardella.id.2000 | Inverse variance weighted | 23.45103879 | 8 | 0.002830952 |
| genus.Hungatella.id.11306 | MR Egger | 0.143386519 | 3 | 0.986165088 |
| genus.Hungatella.id.11306 | Inverse variance weighted | 4.067464835 | 4 | 0.396952467 |
| genus.Intestinibacter.id.11345 | MR Egger | 5.898274018 | 13 | 0.949776893 |
| genus.Intestinibacter.id.11345 | Inverse variance weighted | 8.695001562 | 14 | 0.850078565 |
| genus.Intestinimonas.id.2062 | MR Egger | 14.9534742 | 14 | 0.381342537 |
| genus.Intestinimonas.id.2062 | Inverse variance weighted | 16.71861652 | 15 | 0.335961918 |
| genus.Lachnoclostridium.id.11308 | MR Egger | 19.19734068 | 11 | 0.057641866 |
| genus.Lachnoclostridium.id.11308 | Inverse variance weighted | 19.46329914 | 12 | 0.077944335 |
| genus.Lachnospira.id.2004 | MR Egger | 1.222977767 | 4 | 0.874301183 |
| genus.Lachnospira.id.2004 | Inverse variance weighted | 2.851604945 | 5 | 0.72285109 |
| genus.LachnospiraceaeFCS020group.id.11314 | MR Egger | 8.79954114 | 11 | 0.640389542 |
| genus.LachnospiraceaeFCS020group.id.11314 | Inverse variance weighted | 9.056288419 | 12 | 0.698115279 |
| genus.LachnospiraceaeNC2004group.id.11316 | MR Egger | 1.859729248 | 7 | 0.967194385 |
| genus.LachnospiraceaeNC2004group.id.11316 | Inverse variance weighted | 3.149965178 | 8 | 0.924596729 |
| genus.LachnospiraceaeND3007group.id.11317 | MR Egger | 0.389570953 | 1 | 0.532525013 |
| genus.LachnospiraceaeND3007group.id.11317 | Inverse variance weighted | 0.402579816 | 2 | 0.817675346 |
| genus.LachnospiraceaeNK4A136group.id.11319 | MR Egger | 11.19837123 | 13 | 0.594203548 |
| genus.LachnospiraceaeNK4A136group.id.11319 | Inverse variance weighted | 12.78560183 | 14 | 0.543470789 |
| genus.LachnospiraceaeUCG001.id.11321 | MR Egger | 13.97410013 | 11 | 0.23442994 |
| genus.LachnospiraceaeUCG001.id.11321 | Inverse variance weighted | 14.04017497 | 12 | 0.298150135 |
| genus.LachnospiraceaeUCG004.id.11324 | MR Egger | 15.35985221 | 11 | 0.166608238 |
| genus.LachnospiraceaeUCG004.id.11324 | Inverse variance weighted | 17.72607246 | 12 | 0.124264577 |
| genus.LachnospiraceaeUCG008.id.11328 | MR Egger | 3.951145192 | 10 | 0.949523992 |
| genus.LachnospiraceaeUCG008.id.11328 | Inverse variance weighted | 4.602279724 | 11 | 0.948889721 |
| genus.LachnospiraceaeUCG010.id.11330 | MR Egger | 5.87441613 | 8 | 0.661296794 |
| genus.LachnospiraceaeUCG010.id.11330 | Inverse variance weighted | 6.897499495 | 9 | 0.647790392 |
| genus.Lactobacillus.id.1837 | MR Egger | 4.904098934 | 6 | 0.556170811 |
| genus.Lactobacillus.id.1837 | Inverse variance weighted | 5.48585713 | 7 | 0.600890313 |
| genus.Lactococcus.id.1851 | MR Egger | 3.022210983 | 6 | 0.806053999 |
| genus.Lactococcus.id.1851 | Inverse variance weighted | 5.685829321 | 7 | 0.576874139 |
| genus.Marvinbryantia.id.2005 | MR Egger | 9.945040482 | 9 | 0.354952819 |
| genus.Marvinbryantia.id.2005 | Inverse variance weighted | 14.20198441 | 10 | 0.16397624 |
| genus.Methanobrevibacter.id.123 | MR Egger | 3.512385711 | 4 | 0.475997571 |
| genus.Methanobrevibacter.id.123 | Inverse variance weighted | 3.91406065 | 5 | 0.56185439 |
| genus.Odoribacter.id.952 | MR Egger | 11.14001434 | 5 | 0.048673506 |
| genus.Odoribacter.id.952 | Inverse variance weighted | 12.08789366 | 6 | 0.060036484 |
| genus.Olsenella.id.822 | MR Egger | 4.801437762 | 8 | 0.778572641 |
| genus.Olsenella.id.822 | Inverse variance weighted | 6.783234147 | 9 | 0.659676202 |
| genus.Oscillibacter.id.2063 | MR Egger | 9.662959057 | 11 | 0.560934617 |
| genus.Oscillibacter.id.2063 | Inverse variance weighted | 9.737595759 | 12 | 0.638968967 |
| genus.Oscillospira.id.2064 | MR Egger | 11.15831582 | 7 | 0.131860894 |
| genus.Oscillospira.id.2064 | Inverse variance weighted | 11.83417647 | 8 | 0.158757287 |
| genus.Oxalobacter.id.2978 | MR Egger | 7.113626132 | 9 | 0.625290483 |
| genus.Oxalobacter.id.2978 | Inverse variance weighted | 7.286476224 | 10 | 0.698151257 |
| genus.Parabacteroides.id.954 | MR Egger | 1.265340004 | 3 | 0.73737942 |
| genus.Parabacteroides.id.954 | Inverse variance weighted | 1.410863033 | 4 | 0.842304788 |
| genus.Paraprevotella.id.962 | MR Egger | 9.560470769 | 11 | 0.570301391 |
| genus.Paraprevotella.id.962 | Inverse variance weighted | 10.33071207 | 12 | 0.586972061 |
| genus.Parasutterella.id.2892 | MR Egger | 7.043596391 | 12 | 0.85471911 |
| genus.Parasutterella.id.2892 | Inverse variance weighted | 8.113856743 | 13 | 0.836104358 |
| genus.Peptococcus.id.2037 | MR Egger | 9.835822319 | 12 | 0.630361306 |
| genus.Peptococcus.id.2037 | Inverse variance weighted | 11.42523136 | 13 | 0.575240761 |
| genus.Phascolarctobacterium.id.2168 | MR Egger | 3.915782649 | 7 | 0.789424949 |
| genus.Phascolarctobacterium.id.2168 | Inverse variance weighted | 4.56648283 | 8 | 0.802745445 |
| genus.Prevotella7.id.11182 | MR Egger | 6.095523704 | 9 | 0.73032111 |
| genus.Prevotella7.id.11182 | Inverse variance weighted | 6.104069341 | 10 | 0.806445282 |
| genus.Prevotella9.id.11183 | MR Egger | 12.86031905 | 13 | 0.458658084 |
| genus.Prevotella9.id.11183 | Inverse variance weighted | 13.58302256 | 14 | 0.481215124 |
| genus.RikenellaceaeRC9gutgroup.id.11191 | MR Egger | 6.234086387 | 10 | 0.795227773 |
| genus.RikenellaceaeRC9gutgroup.id.11191 | Inverse variance weighted | 6.645930442 | 11 | 0.82698943 |
| genus.Romboutsia.id.11347 | MR Egger | 25.13498676 | 12 | 0.014196009 |
| genus.Romboutsia.id.11347 | Inverse variance weighted | 25.84798851 | 13 | 0.017817476 |
| genus.Roseburia.id.2012 | MR Egger | 11.72000448 | 12 | 0.468420415 |
| genus.Roseburia.id.2012 | Inverse variance weighted | 13.64212201 | 13 | 0.399521333 |
| genus.Ruminiclostridium5.id.11355 | MR Egger | 8.636618543 | 9 | 0.471470438 |
| genus.Ruminiclostridium5.id.11355 | Inverse variance weighted | 9.027710495 | 10 | 0.529475785 |
| genus.Ruminiclostridium6.id.11356 | MR Egger | 6.897503557 | 13 | 0.907356001 |
| genus.Ruminiclostridium6.id.11356 | Inverse variance weighted | 6.98899297 | 14 | 0.935135381 |
| genus.Ruminiclostridium9.id.11357 | MR Egger | 2.758053197 | 7 | 0.906446168 |
| genus.Ruminiclostridium9.id.11357 | Inverse variance weighted | 2.916576483 | 8 | 0.939483146 |
| genus.RuminococcaceaeNK4A214group.id.11358 | MR Egger | 11.8795383 | 12 | 0.455402008 |
| genus.RuminococcaceaeNK4A214group.id.11358 | Inverse variance weighted | 13.72028876 | 13 | 0.393833414 |
| genus.RuminococcaceaeUCG002.id.11360 | MR Egger | 21.9316594 | 18 | 0.23503338 |
| genus.RuminococcaceaeUCG002.id.11360 | Inverse variance weighted | 21.9945319 | 19 | 0.284528483 |
| genus.RuminococcaceaeUCG003.id.11361 | MR Egger | 3.536320297 | 10 | 0.965850727 |
| genus.RuminococcaceaeUCG003.id.11361 | Inverse variance weighted | 3.695512692 | 11 | 0.977992976 |
| genus.RuminococcaceaeUCG004.id.11362 | MR Egger | 6.108538602 | 9 | 0.729008056 |
| genus.RuminococcaceaeUCG004.id.11362 | Inverse variance weighted | 6.371943366 | 10 | 0.783106445 |
| genus.RuminococcaceaeUCG005.id.11363 | MR Egger | 14.08890559 | 12 | 0.295067003 |
| genus.RuminococcaceaeUCG005.id.11363 | Inverse variance weighted | 14.99913161 | 13 | 0.307406989 |
| genus.RuminococcaceaeUCG009.id.11366 | MR Egger | 7.716313429 | 9 | 0.562968388 |
| genus.RuminococcaceaeUCG009.id.11366 | Inverse variance weighted | 7.716427421 | 10 | 0.656513666 |
| genus.RuminococcaceaeUCG010.id.11367 | MR Egger | 6.909912279 | 4 | 0.140726228 |
| genus.RuminococcaceaeUCG010.id.11367 | Inverse variance weighted | 7.008307228 | 5 | 0.220023222 |
| genus.RuminococcaceaeUCG011.id.11368 | MR Egger | 10.57323621 | 6 | 0.102496059 |
| genus.RuminococcaceaeUCG011.id.11368 | Inverse variance weighted | 11.73066797 | 7 | 0.109771182 |
| genus.RuminococcaceaeUCG013.id.11370 | MR Egger | 7.386361113 | 9 | 0.596961046 |
| genus.RuminococcaceaeUCG013.id.11370 | Inverse variance weighted | 9.144656898 | 10 | 0.518432434 |
| genus.RuminococcaceaeUCG014.id.11371 | MR Egger | 21.9113662 | 8 | 0.005082734 |
| genus.RuminococcaceaeUCG014.id.11371 | Inverse variance weighted | 21.98828565 | 9 | 0.008916178 |
| genus.Ruminococcus1.id.11373 | MR Egger | 5.018250584 | 8 | 0.755623715 |
| genus.Ruminococcus1.id.11373 | Inverse variance weighted | 7.651406346 | 9 | 0.569621235 |
| genus.Ruminococcus2.id.11374 | MR Egger | 13.68001634 | 13 | 0.396758456 |
| genus.Ruminococcus2.id.11374 | Inverse variance weighted | 13.70312658 | 14 | 0.472054499 |
| genus.Sellimonas.id.14369 | MR Egger | 4.217329613 | 7 | 0.754432674 |
| genus.Sellimonas.id.14369 | Inverse variance weighted | 4.549440754 | 8 | 0.804466575 |
| genus.Senegalimassilia.id.11160 | MR Egger | 4.981605339 | 3 | 0.173149058 |
| genus.Senegalimassilia.id.11160 | Inverse variance weighted | 5.018817872 | 4 | 0.285372108 |
| genus.Slackia.id.825 | MR Egger | 8.884681162 | 4 | 0.064047431 |
| genus.Slackia.id.825 | Inverse variance weighted | 9.126482478 | 5 | 0.104124308 |
| genus.Streptococcus.id.1853 | MR Egger | 13.68266114 | 12 | 0.321430293 |
| genus.Streptococcus.id.1853 | Inverse variance weighted | 14.91549423 | 13 | 0.312657972 |
| genus.Subdoligranulum.id.2070 | MR Egger | 11.23771214 | 9 | 0.259766316 |
| genus.Subdoligranulum.id.2070 | Inverse variance weighted | 11.25256531 | 10 | 0.338182443 |
| genus.Sutterella.id.2896 | MR Egger | 6.323539408 | 10 | 0.787387971 |
| genus.Sutterella.id.2896 | Inverse variance weighted | 6.906897213 | 11 | 0.80657224 |
| genus.Terrisporobacter.id.11348 | MR Egger | 9.314799478 | 3 | 0.025385437 |
| genus.Terrisporobacter.id.11348 | Inverse variance weighted | 13.38826734 | 4 | 0.009526523 |
| genus.Turicibacter.id.2162 | MR Egger | 8.557317599 | 7 | 0.286019351 |
| genus.Turicibacter.id.2162 | Inverse variance weighted | 9.020027807 | 8 | 0.340609252 |
| genus.Tyzzerella3.id.11335 | MR Egger | 5.26132366 | 10 | 0.873051647 |
| genus.Tyzzerella3.id.11335 | Inverse variance weighted | 5.263329215 | 11 | 0.917745868 |
| genus.Veillonella.id.2198 | MR Egger | 0.349177125 | 3 | 0.950529088 |
| genus.Veillonella.id.2198 | Inverse variance weighted | 1.031614436 | 4 | 0.904964915 |
| genus.Victivallis.id.2256 | MR Egger | 5.244120124 | 9 | 0.812529565 |
| genus.Victivallis.id.2256 | Inverse variance weighted | 5.244744591 | 10 | 0.87424058 |

**Table S5** The directional horizontal pleiotropy when assessed by intercept terms in MR Egger regression and MR-PRESSO between gut microbiota and puerperal sepsis.

| **Bacterial taxa (exposure)** | **MR Egger regression** | | | **MR-PRESSO** |
| --- | --- | --- | --- | --- |
|  | **Egger_intercept** | **SE** | **P-value** | **Global test P-value** |
| genus..Clostridiuminnocuumgroup.id.14397 | -0.03602317 | 0.067661763 | 0.613578106 | 0.164 |
| genus..Eubacteriumbrachygroup.id.11296 | 0.028213999 | 0.035616586 | 0.451116451 | 0.784 |
| genus..Eubacteriumcoprostanoligenesgroup.id.11375 | 0.043823835 | 0.038725978 | 0.284193274 | 0.104 |
| genus..Eubacteriumeligensgroup.id.14372 | -0.00407454 | 0.040639743 | 0.924034017 | 0.81 |
| genus..Eubacteriumfissicatenagroup.id.14373 | -0.05359815 | 0.057964426 | 0.385902757 | 0.286 |
| genus..Eubacteriumhalliigroup.id.11338 | -0.01965785 | 0.017652289 | 0.287253618 | 0.929 |
| genus..Eubacteriumnodatumgroup.id.11297 | -0.01395661 | 0.039647002 | 0.732923837 | 0.875 |
| genus..Eubacteriumoxidoreducensgroup.id.11339 | -0.05361574 | 0.044812685 | 0.317458778 | 0.562 |
| genus..Eubacteriumrectalegroup.id.14374 | -0.03705091 | 0.044169698 | 0.429275476 | 0.145 |
| genus..Eubacteriumruminantiumgroup.id.11340 | 0.020088967 | 0.02179361 | 0.370333262 | 0.628 |
| genus..Eubacteriumventriosumgroup.id.11341 | 0.034891176 | 0.036314908 | 0.35418607 | 0.874 |
| genus..Eubacteriumxylanophilumgroup.id.14375 | -0.02603052 | 0.029364056 | 0.404785672 | 0.743 |
| genus..Ruminococcusgauvreauiigroup.id.11342 | 0.021938986 | 0.041936066 | 0.613502294 | 0.216 |
| genus..Ruminococcusgnavusgroup.id.14376 | 0.000824894 | 0.049025609 | 0.986942706 | 0.332 |
| genus..Ruminococcustorquesgroup.id.14377 | -0.0848644 | 0.053361695 | 0.162856603 | 0.018 |
| genus.Actinomyces.id.423 | 0.009997994 | 0.031785155 | 0.765806072 | 0.478 |
| genus.Adlercreutzia.id.812 | -0.07418634 | 0.049374795 | 0.183650327 | 0.223 |
| genus.Akkermansia.id.4037 | 0.000982546 | 0.0349846 | 0.978146853 | 0.198 |
| genus.Alistipes.id.968 | 0.025738365 | 0.040590867 | 0.540253336 | 0.953 |
| genus.Allisonella.id.2174 | -0.0470619 | 0.064568035 | 0.493535583 | 0.86 |
| genus.Alloprevotella.id.961 | 0.156056642 | 0.222998837 | 0.534430635 | 0.008 |
| genus.Anaerofilum.id.2053 | 0.051865619 | 0.052074507 | 0.34842319 | 0.27 |
| genus.Anaerostipes.id.1991 | 0.017104541 | 0.031642471 | 0.599587704 | 0.369 |
| genus.Anaerotruncus.id.2054 | -0.04510591 | 0.025233382 | 0.10139885 | 0.204 |
| genus.Bacteroides.id.918 | -0.01493358 | 0.051810938 | 0.782866846 | 0.949 |
| genus.Barnesiella.id.944 | -0.02895948 | 0.036130104 | 0.441452694 | 0.663 |
| genus.Bifidobacterium.id.436 | 0.017048417 | 0.018104281 | 0.359547382 | 0.438 |
| genus.Bilophila.id.3170 | -0.01137078 | 0.040534729 | 0.784284622 | 0.75 |
| genus.Blautia.id.1992 | 0.012564472 | 0.031110115 | 0.694809122 | 0.113 |
| genus.Butyricicoccus.id.2055 | -0.01662282 | 0.023382096 | 0.503818305 | 0.734 |
| genus.Butyricimonas.id.945 | 0.038067136 | 0.031912058 | 0.258013243 | 0.914 |
| genus.Butyrivibrio.id.1993 | 0.033407534 | 0.031425748 | 0.307102791 | 0.765 |
| genus.CandidatusSoleaferrea.id.11350 | -0.05854152 | 0.093620951 | 0.551603908 | 0.358 |
| genus.Catenibacterium.id.2153 | 0.121834603 | 0.162905646 | 0.532511134 | 0.897 |
| genus.ChristensenellaceaeR.7group.id.11283 | -0.02282878 | 0.042855568 | 0.613389015 | 0.743 |
| genus.Clostridiumsensustricto1.id.1873 | -0.02309566 | 0.03558502 | 0.551702698 | 0.559 |
| genus.Collinsella.id.815 | -0.05691105 | 0.03874879 | 0.180099582 | 0.507 |
| genus.Coprobacter.id.949 | -0.04466206 | 0.03755902 | 0.264819714 | 0.309 |
| genus.Coprococcus1.id.11301 | -0.00648732 | 0.022958752 | 0.783273093 | 0.443 |
| genus.Coprococcus2.id.11302 | -0.01369154 | 0.052791787 | 0.802827225 | 0.479 |
| genus.Coprococcus3.id.11303 | -0.06469985 | 0.050735816 | 0.238011956 | 0.817 |
| genus.DefluviitaleaceaeUCG011.id.11287 | -0.04162144 | 0.043588034 | 0.367596536 | 0.238 |
| genus.Desulfovibrio.id.3173 | -0.04022154 | 0.028811408 | 0.196182477 | 0.601 |
| genus.Dialister.id.2183 | 0.051393292 | 0.034409811 | 0.169494108 | 0.423 |
| genus.Dorea.id.1997 | -0.01904288 | 0.022570809 | 0.420697937 | 0.597 |
| genus.Eggerthella.id.819 | 0.077413589 | 0.041112289 | 0.10171958 | 0.46 |
| genus.Eisenbergiella.id.11304 | 0.048661793 | 0.067177442 | 0.487236911 | 0.335 |
| genus.Enterorhabdus.id.820 | -0.09019406 | 0.042404393 | 0.10054689 | 0.253 |
| genus.Erysipelatoclostridium.id.11381 | -0.05110406 | 0.031375766 | 0.125647371 | 0.095 |
| genus.ErysipelotrichaceaeUCG003.id.11384 | 0.011590627 | 0.023398358 | 0.627523549 | 0.876 |
| genus.Escherichia.Shigella.id.3504 | -0.08341963 | 0.033683411 | 0.038315435 | 0.027 |
| genus.Faecalibacterium.id.2057 | -0.01815963 | 0.029676178 | 0.557573051 | 0.113 |
| genus.FamilyXIIIAD3011group.id.11293 | -0.03387859 | 0.051868434 | 0.527066821 | 0.196 |
| genus.FamilyXIIIUCG001.id.11294 | -0.02435796 | 0.034369705 | 0.505097902 | 0.579 |
| genus.Flavonifractor.id.2059 | 0.035733268 | 0.048577934 | 0.502785415 | 0.573 |
| genus.Fusicatenibacter.id.11305 | -0.01777859 | 0.026048779 | 0.504110295 | 0.555 |
| genus.Gordonibacter.id.821 | 0.035495893 | 0.043069457 | 0.431150271 | 0.352 |
| genus.Haemophilus.id.3698 | -0.01426357 | 0.024785786 | 0.582988383 | 0.83 |
| genus.Holdemanella.id.11393 | -0.00659933 | 0.025526823 | 0.8018167 | 0.656 |
| genus.Holdemania.id.2157 | 0.007333042 | 0.024906916 | 0.773469527 | 0.769 |
| genus.Howardella.id.2000 | 0.034062569 | 0.073320935 | 0.656344321 | 0.007 |
| genus.Hungatella.id.11306 | 0.157107698 | 0.079310124 | 0.141929249 | 0.448 |
| genus.Intestinibacter.id.11345 | -0.0415968 | 0.024873385 | 0.118337666 | 0.865 |
| genus.Intestinimonas.id.2062 | -0.02947991 | 0.02293207 | 0.219464943 | 0.331 |
| genus.Lachnoclostridium.id.11308 | -0.01454577 | 0.037260957 | 0.703712495 | 0.074 |
| genus.Lachnospira.id.2004 | 0.085659308 | 0.06712182 | 0.270956395 | 0.702 |
| genus.LachnospiraceaeFCS020group.id.11314 | -0.0107065 | 0.02112977 | 0.622366139 | 0.736 |
| genus.LachnospiraceaeNC2004group.id.11316 | 0.048261014 | 0.042487568 | 0.293393342 | 0.927 |
| genus.LachnospiraceaeND3007group.id.11317 | -0.02901291 | 0.254373337 | 0.927701864 | NA |
| genus.LachnospiraceaeNK4A136group.id.11319 | 0.018219189 | 0.014461357 | 0.229870326 | 0.582 |
| genus.LachnospiraceaeUCG001.id.11321 | 0.009178509 | 0.040245714 | 0.823782141 | 0.32 |
| genus.LachnospiraceaeUCG004.id.11324 | -0.05014083 | 0.038517767 | 0.219600054 | 0.123 |
| genus.LachnospiraceaeUCG008.id.11328 | 0.032337403 | 0.040074653 | 0.438477898 | 0.944 |
| genus.LachnospiraceaeUCG010.id.11330 | 0.030356814 | 0.030012397 | 0.341399482 | 0.68 |
| genus.Lactobacillus.id.1837 | -0.01992378 | 0.026121647 | 0.474532708 | 0.655 |
| genus.Lactococcus.id.1851 | 0.081646758 | 0.050026826 | 0.153787293 | 0.553 |
| genus.Marvinbryantia.id.2005 | 0.074452925 | 0.037932793 | 0.081283137 | 0.199 |
| genus.Methanobrevibacter.id.123 | -0.03103663 | 0.048970795 | 0.560656672 | 0.553 |
| genus.Odoribacter.id.952 | -0.03434038 | 0.052648548 | 0.543027637 | 0.093 |
| genus.Olsenella.id.822 | 0.039454549 | 0.028026416 | 0.196850776 | 0.703 |
| genus.Oscillibacter.id.2063 | -0.00851644 | 0.031173237 | 0.789762788 | 0.654 |
| genus.Oscillospira.id.2064 | -0.03833933 | 0.058879785 | 0.535734863 | 0.173 |
| genus.Oxalobacter.id.2978 | -0.01963134 | 0.04721883 | 0.687329027 | 0.733 |
| genus.Parabacteroides.id.954 | 0.039057579 | 0.102385735 | 0.728265933 | 0.868 |
| genus.Paraprevotella.id.962 | 0.026332474 | 0.030003939 | 0.398905616 | 0.577 |
| genus.Parasutterella.id.2892 | 0.020880124 | 0.020183122 | 0.32128584 | 0.843 |
| genus.Peptococcus.id.2037 | -0.04418611 | 0.035048376 | 0.231372265 | 0.606 |
| genus.Phascolarctobacterium.id.2168 | 0.036399016 | 0.045123126 | 0.446398961 | 0.807 |
| genus.Prevotella7.id.11182 | -0.00477765 | 0.051682305 | 0.928371417 | 0.821 |
| genus.Prevotella9.id.11183 | 0.019955066 | 0.023473243 | 0.410642637 | 0.48 |
| genus.RikenellaceaeRC9gutgroup.id.11191 | 0.03137445 | 0.048888839 | 0.535467562 | 0.828 |
| genus.Romboutsia.id.11347 | -0.02148045 | 0.036816864 | 0.570400159 | 0.012 |
| genus.Roseburia.id.2012 | 0.037117995 | 0.026772846 | 0.190842275 | 0.407 |
| genus.Ruminiclostridium5.id.11355 | 0.022287663 | 0.035638965 | 0.547253639 | 0.569 |
| genus.Ruminiclostridium6.id.11356 | 0.006788633 | 0.022443827 | 0.767077393 | 0.932 |
| genus.Ruminiclostridium9.id.11357 | 0.019157724 | 0.048116872 | 0.702383672 | 0.928 |
| genus.RuminococcaceaeNK4A214group.id.11358 | -0.03725786 | 0.027461257 | 0.19983761 | 0.44 |
| genus.RuminococcaceaeUCG002.id.11360 | 0.004527081 | 0.019929087 | 0.822859739 | 0.302 |
| genus.RuminococcaceaeUCG003.id.11361 | 0.010729848 | 0.026892577 | 0.698288415 | 0.978 |
| genus.RuminococcaceaeUCG004.id.11362 | -0.02411532 | 0.046987372 | 0.620150761 | 0.796 |
| genus.RuminococcaceaeUCG005.id.11363 | 0.022496298 | 0.025549619 | 0.395883615 | 0.31 |
| genus.RuminococcaceaeUCG009.id.11366 | 0.000352149 | 0.032982948 | 0.99171432 | 0.666 |
| genus.RuminococcaceaeUCG010.id.11367 | 0.008945231 | 0.037481021 | 0.823097544 | 0.274 |
| genus.RuminococcaceaeUCG011.id.11368 | 0.050293128 | 0.062056788 | 0.448631661 | 0.117 |
| genus.RuminococcaceaeUCG013.id.11370 | 0.036666395 | 0.027651726 | 0.217503222 | 0.507 |
| genus.RuminococcaceaeUCG014.id.11371 | -0.0062313 | 0.037183491 | 0.871070671 | 0.014 |
| genus.Ruminococcus1.id.11373 | -0.04181946 | 0.025771525 | 0.143310136 | 0.612 |
| genus.Ruminococcus2.id.11374 | 0.002806156 | 0.018935696 | 0.884463642 | 0.505 |
| genus.Sellimonas.id.14369 | 0.027469372 | 0.047665813 | 0.582465691 | 0.793 |
| genus.Senegalimassilia.id.11160 | 0.009310669 | 0.062195692 | 0.890499425 | 0.319 |
| genus.Slackia.id.825 | -0.03662509 | 0.111004438 | 0.757999439 | 0.132 |
| genus.Streptococcus.id.1853 | -0.03880786 | 0.037321771 | 0.318922353 | 0.303 |
| genus.Subdoligranulum.id.2070 | -0.00284557 | 0.02609019 | 0.915542694 | 0.346 |
| genus.Sutterella.id.2896 | 0.02497054 | 0.032693426 | 0.462638992 | 0.815 |
| genus.Terrisporobacter.id.11348 | -0.07884498 | 0.068836385 | 0.335135618 | 0.031 |
| genus.Turicibacter.id.2162 | -0.03058834 | 0.049718855 | 0.557868427 | 0.369 |
| genus.Tyzzerella3.id.11335 | 0.002568973 | 0.057364378 | 0.965161601 | 0.93 |
| genus.Veillonella.id.2198 | -0.20736771 | 0.25102082 | 0.469316948 | 0.905 |
| genus.Victivallis.id.2256 | 0.001351602 | 0.054087112 | 0.980608762 | 0.867 |
