# Supplementary figures and images for: Causal relationship between gut microbiota and puerperal sepsis: a 2-sample Mendelian randomization study
Source: Front Microbiol. 2024 Jun 12;15:1407324. doi: 10.3389/fmicb.2024.1407324 (PMC11203603; doi:10.3389/fmicb.2024.1407324)

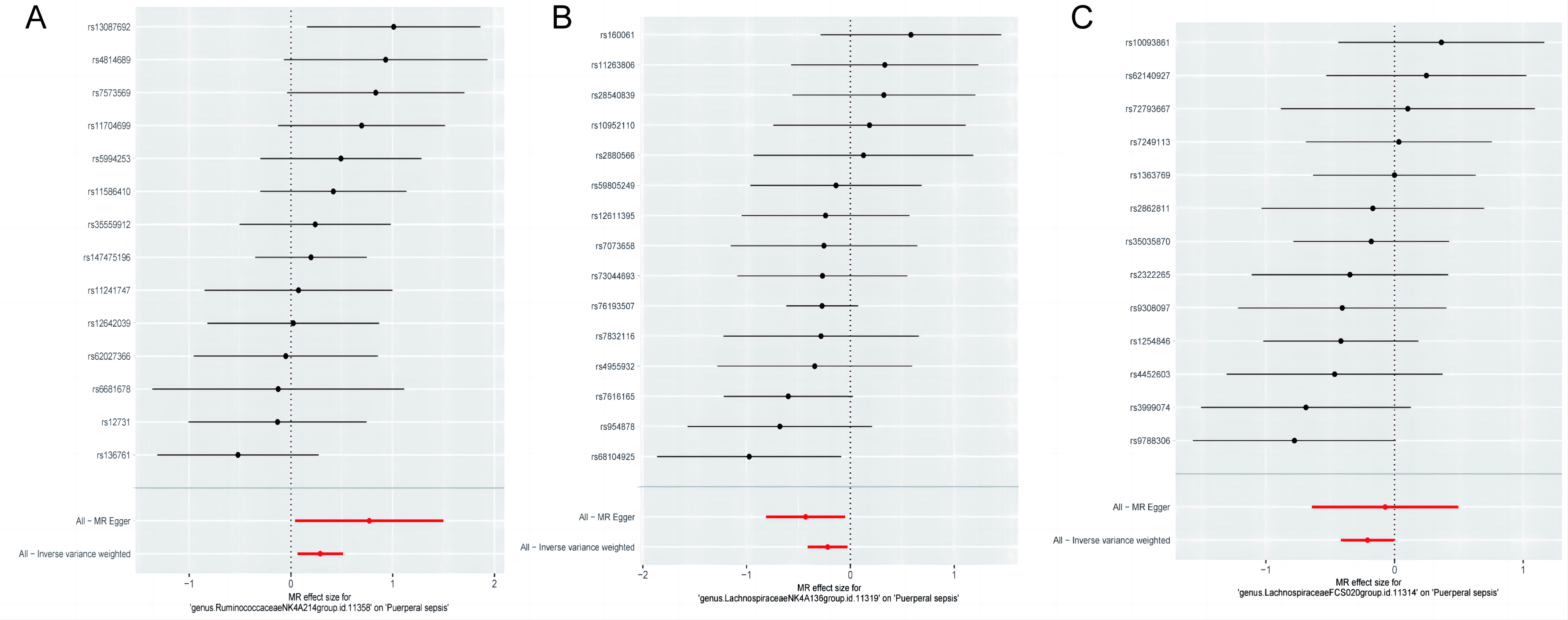

Supplement: Supplementary file 1 [file Data_Sheet_1.zip › Supplementary Figure 1.jpg]

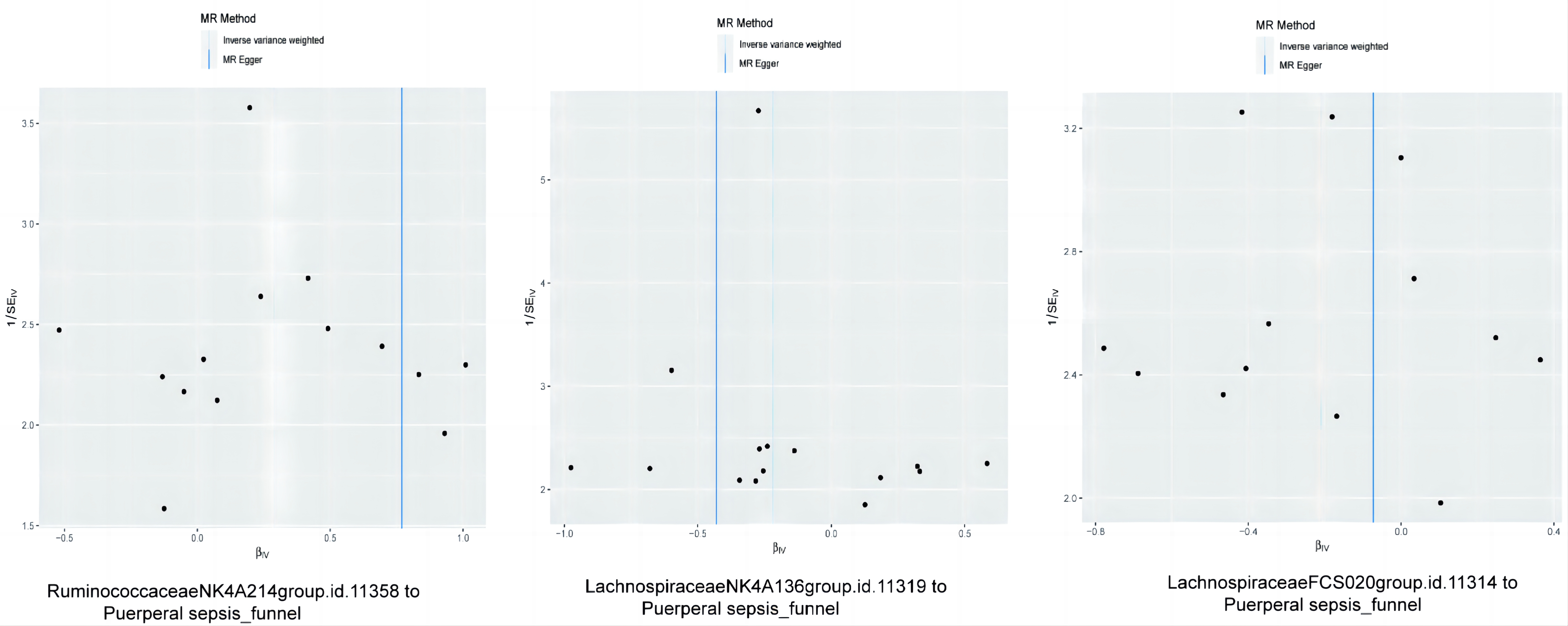

Supplement: Supplementary file 1 [file Data_Sheet_1.zip › Supplementary Figure 2.jpg]

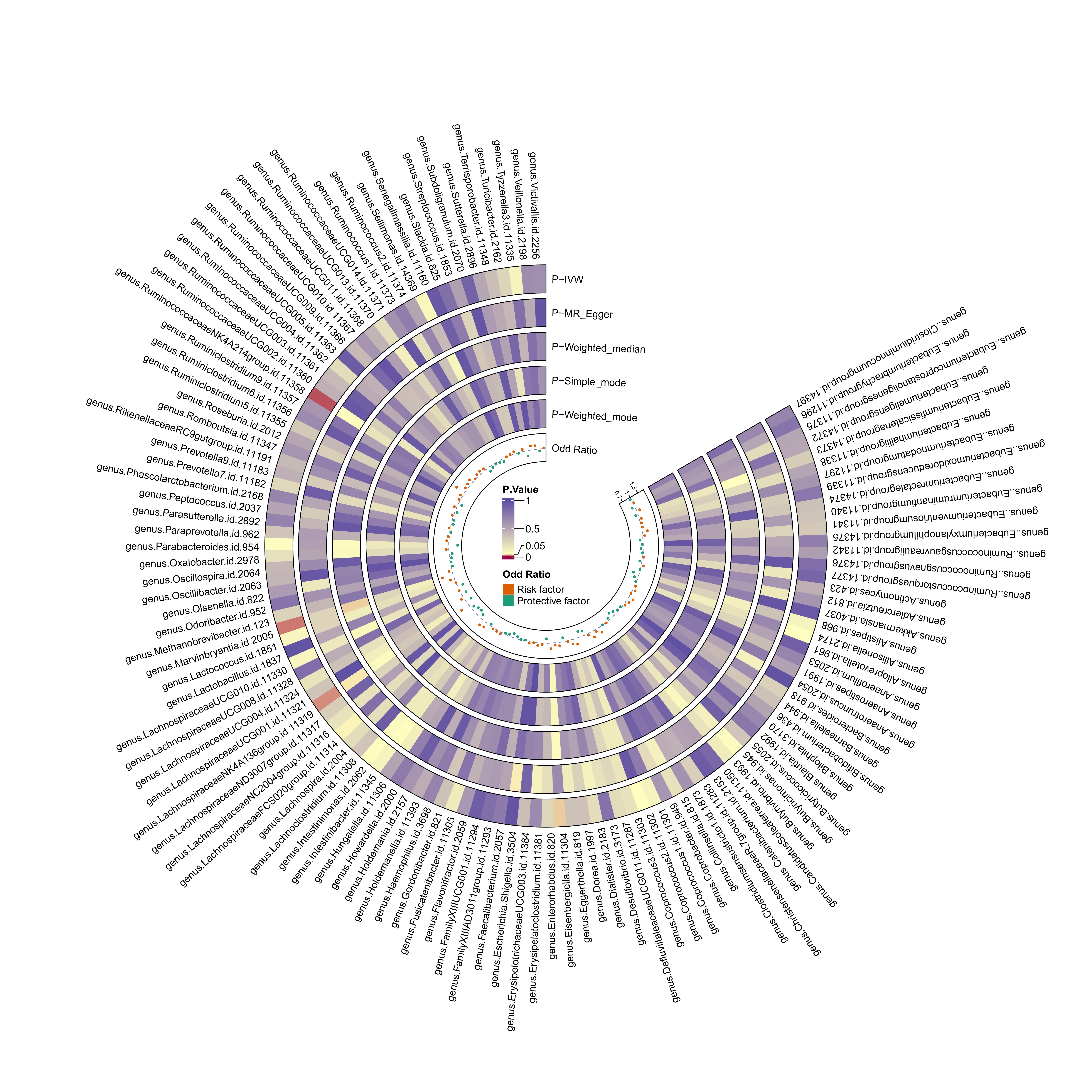

Supplement: Supplementary file 1 [file Data_Sheet_1.zip › Supplementary Figure 3.jpg]
